# Synthesis, *in silico* modelling, and *in vitro* biological evaluation of substituted pyrazole derivatives as potential anti-skin cancer, anti-tyrosinase, and antioxidant agents

**DOI:** 10.1080/14756366.2023.2205042

**Published:** 2023-05-15

**Authors:** Samuel T. Boateng, Tithi Roy, Kara Torrey, Uchechi Owunna, Sergette Banang-Mbeumi, David Basnet, Eleonora Niedda, Alexis D. Alexander, Denzel El Hage, Siriki Atchimnaidu, Bolni Marius Nagalo, Dinesh Aryal, Ann Findley, Navindra P. Seeram, Tatiana Efimova, Mario Sechi, Ronald A. Hill, Hang Ma, Jean Christopher Chamcheu, Siva Murru

**Affiliations:** a School of Basic Pharmaceutical and Toxicological Sciences, College of Pharmacy, University of Louisiana at Monroe, Monroe, LA, USA; b Department of Biomedical and Pharmaceutical Sciences, College of Pharmacy, Bioactive Botanical Research Laboratory, University of Rhode Island, Kingston, RI, USA; c School of Sciences, College of Arts, Education and Sciences, University of Louisiana at Monroe, Monroe, LA, USA; d School of Nursing and Allied Health Sciences, Louisiana Delta Community College, Monroe, LA, USA; e Department of Medicine, Surgery and Pharmacy, University of Sassari, Sassari, Italy; f Department of Pathology, University of Arkansas for Medical Sciences (UAMS), Little Rock, AR, USA; g The Winthrop P. Rockefeller Cancer Institute, UAMS, Little Rock, AR, USA; h Department of Biomedical Affairs and Research, Edward Via College of Osteopathic Medicine, Monroe, LA, USA; i Department of Biomedical Engineering, Northwestern University, Chicago, IL, USA

**Keywords:** Antitumor agents, apoptosis, tyrosinase inhibition, antioxidant, molecular docking and ADMET

## Abstract

Twenty-five azole compounds (**P1**–**P25**) were synthesised using regioselective base-metal catalysed and microwave-assisted approaches, fully characterised by high-resolution mass spectrometry (HRMS), nuclear magnetic resonance (NMR), and infrared spectra (IR) analyses, and evaluated for anticancer, anti-tyrosinase, and anti-oxidant activities *in silico* and *in vitro*. **P25** exhibited potent anticancer activity against cells of four skin cancer (SC) lines, with selectivity for melanoma (A375, SK-Mel-28) or non-melanoma (A431, SCC-12) SC cells over non-cancerous HaCaT-keratinocytes. Clonogenic, scratch-wound, and immunoblotting assay data were consistent with anti-proliferative results, expression profiling therewith implicating intrinsic and extrinsic apoptosis activation. In a mushroom tyrosinase inhibition assay, **P14** was most potent among the compounds (half-maximal inhibitory concentration where 50% of cells are dead, IC_50_ 15.9 μM), with activity greater than arbutin and kojic acid. Also, **P6** exhibited noteworthy free radical-scavenging activity. Furthermore, *in silico* docking and absorption, distribution, metabolism, excretion, and toxicity (ADMET) simulations predicted prominent-phenotypic actives to engage diverse cancer/hyperpigmentation-related targets with relatively high affinities. Altogether, promising early-stage hits were identified – some with multiple activities – warranting further hit-to-lead optimisation chemistry with further biological evaluations, towards identifying new skin-cancer and skin-pigmentation renormalising agents.

## Graphical Abstract

**Figure:**
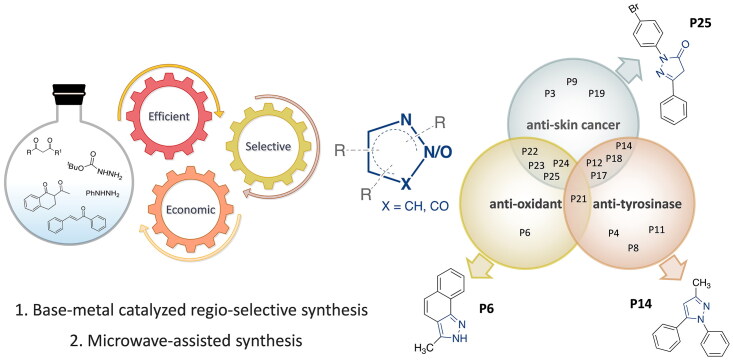

## Introduction

Cancer is the second-leading cause of death after cardiovascular diseases, and skin cancer (SC) is one of the most prevalent cancer types globally, with incidence and resultant mortality expected to keep growing at alarming rates^1^^,^^2^. Non-melanoma SCs, including squamous cell carcinomas (SCCs) and basal cell carcinomas (BCCs), and melanomas affect nearly 3 million and more than 120 000 people globally each year, respectively^3^. The still less-than-complete restoration of the ozone layer allows increased amounts of UVB radiation to reach the Earth’s surface, which is a significant risk factor for developing cutaneous cancers^4–6^. Despite surgical tumour excision being among the most effective SC treatment approaches, such interventions often lead to disfigurement, requiring additional costly skin grafts to cover resultant defects^7–11^. Moreover, targeting proliferation and inducing apoptosis in proliferating cancer cells are effective approaches in cancer therapy^12–15^, and significant strides have been made recently towards a better understanding of the mechanisms that trigger and sustain SCs, thus revealing new possibilities for intervention.

Tyrosinase (also known as polyphenol oxidase, PPO) is a rate-limiting binuclear copper-containing metalloenzyme that enables the biosynthesis of melanin polymers. These polymers are high-molecular-weight pigments known for protecting the skin against the deleterious effects of UV rays^16–19^. Specifically, tyrosinase catalyses the first two steps in melanogenesis (the process of melanin synthesis). It converts l-tyrosine, viz. hydroxylation to 3,4-dihydroxyphenylalanine (l-DOPA) and effects subsequent oxidation of l-DOPA to DOPA-quinone, which spontaneously polymerises to form melanin in the melanosomes of melanocytes^18–27^. The phosphorylated isomers of l-DOPA or l-tyrosine are vital regulatory molecules of melanogenesis within melanocytes localised in the skin epidermal basal layer and hair follicles. In the skin, every melanocyte is bound to over 30 keratinocytes (acting as melanin-recipient cells) to distribute the synthesised melanin and dictate the colour of human skin, eye (pigmented retina), and hair^19^^,^^27–31^. Diverse extrinsic and intrinsic factors such as UV exposure, α-melanocyte-stimulating hormone (α-MSH), melanocortin 1 receptor (MC1R), and pathogenic mutations modulate melanogenesis and aberrant proliferation of melanocytes. Altered melanin production, and accumulation or deficiency of melanocytes and melanin, can lead to various dermatoses and oculo-cutaneous pigmentation disorders such as albinism, age-related spots, chloasma, freckles, post-inflammatory hyperpigmentation, melasma, lentigo, malignant melanoma, and oculocutaneous albinism type 1 (OCA1)^24^^,^^30^^,^^32–39^.

Pharmacological modulation of tyrosinase-dependent melanogenesis is a potential therapeutic approach for combatting melanoma as well as other pigmentation-related disorders^40–44^. A number of naturally occurring tyrosinase inhibitors, including arbutin, azelaic acid, hydroquinone, kojic acid, and various phenolics, can reduce excessive melanin production to improve skin tone and treat dermatoses^30^^,^^34^^,^^40^^,^^45–47^. However, due to safety concerns related to serious adverse effects, only a few are currently in clinical/cosmeceutical use^48^. This dearth of options provides the impetus to create and develop new anti-tyrosinase agents that are prospectively safer and more efficacious alternatives for treating pigment-related dermatological disorders^38^^,^^49^^,^^50^. Compounds built on an array of natural or synthetic scaffolds, including various azoles, chalcones, flavonoids, stilbenes, and tropolones, have been reported to not only exhibit tyrosinase inhibitory activities^26^^,^^38^^,^^50–52^, but also to provide various therapeutic augmentations, such as antioxidant activity^53^^,^^54^, and anti-inflammatory and anticancer actions^55–57^.

In cancers and other skin disorders such as hyperpigmentation, increased generation of reactive oxygen species (ROS) and free radicals, as well as severe perturbations of cellular redox balance, cause enhanced growth, genetic instability, and adaptive responses that promote the maintenance of malignant phenotypes by cancerous cells^58^. Also, hyperpigmentation increases hydrogen peroxide (H_2_O_2_) and ROS levels, resulting in elevated oxidative stress load on melanocytes. Thus, antioxidant or ROS buffering in disease conditions has long been a significant drug discovery and development focus^59^.

**Figure 1.**
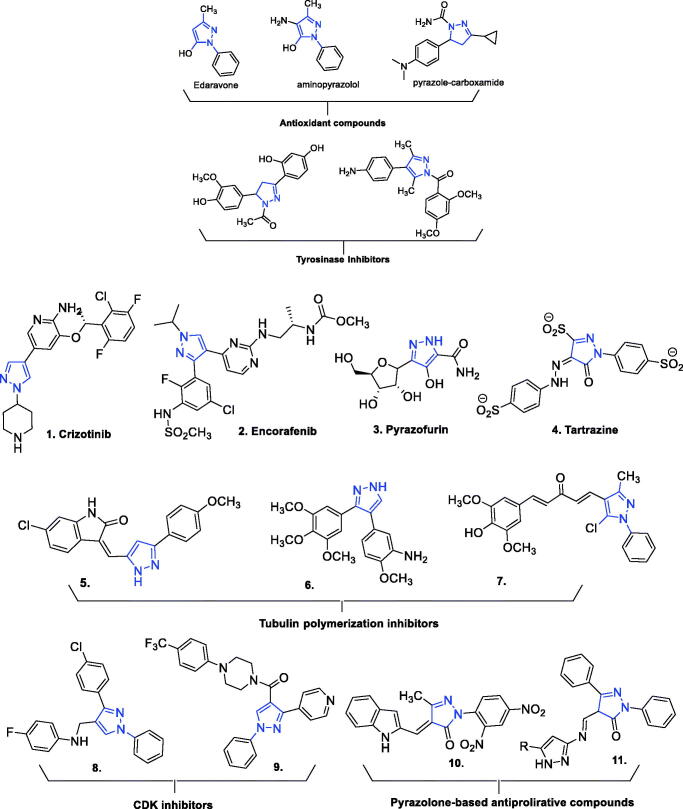
Molecular structures of previously reported antioxidant, antityrosinase, and anticancer compounds incorporating pyrazole or pyrazolone substructures.

Numerous small-molecule compounds incorporating a pyrazole substructure exhibit one or more biological activities among a wide array thereof. Of medicinal relevance, these activities include analgesic, anticancer, antibacterial, antifungal, anti-inflammatory, antiviral, antidiabetic, antitubercular, antidepressant, anticonvulsant, antipyretic, anxiolytic, antimalarial, immunosuppressive, and antioxidant (reviewed in refs.^60–67^) The large number of structurally diverse compounds elaborated from a pyrazole core includes representatives exhibiting anticancer activities of wide-ranging character and demonstrated or apparent efficacies against cancers appearing in almost every human organ or tissue, including lung, brain, colon, kidney, prostate, pancreas, and blood^68^^,^^69^. Exemplary market-approved pyrazole-based anti-cancer drugs include crizotinib, encorafenib, pyrazofurin, and tartrazine. Various other pyrazole and pyrazolone derivatives have also exhibited antiproliferative activities mediated through their interactions with various cancer related targets, or directly (Figure 1)^65^^,^^70–75^.

**Figure 2.**
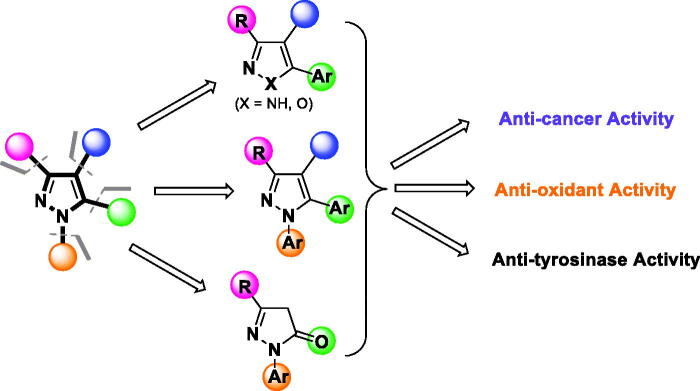
Structural design of biologically relevant substituted azoles and the proposed activity studies.

Given the broad spectrum of activities exhibited by elaborated pyrazoles, this moiety arguably merits “privileged structure” status, offering a versatile core chemotype in drug design and for compounds to be included in physical and virtual libraries for screening-based discovery^76–79^. The pyrazole moiety has four different sites (Figure 2) at which a wide array of substituents can be introduced to achieve a desired pharmacological activity, target selectivity, and other attributes needed for a biochemical/pharmacological tool or clinically/commercially useful agent through hit-to-lead and lead optimisation.

Well-construed *in vitro* screening studies hold promise for expediting discovery-to-development translation, as such studies can often quickly provide crucial information as to whether an early-stage test agent should be discarded or further pursued. This impact has now been seen in projects addressing unmet medical need for numerous human health problems, including cancers, inflammation, and pigmentation anomalies^80^^,^^81^.

As noted above, available treatments still have serious limitations. Beyond safety and intrinsic efficacy, for agents applied dermally, low drug penetrability into the stratum corneum and diseased skin lesions^14^^,^^82^^,^^83^ can compromise the attainment of therapeutic levels at sites-of-action^84^. Compensating with higher applied concentrations can, for many available agents, result in skin irritation, or severe local or even systemic side effects and other safety concerns^85–88^. As part of our effort to discover new synthetic scaffold-based entities with low toxicity, enhanced efficacy, and improved biopharmaceutic profiles against cutaneous cancers, pigmentation disorders, and various other dermal pathologies, we recently synthesised 1,3-diarylpyrazolones having potent suppressive activity against non-small-cell lung cancer cells^89^.

**Figure 3.**
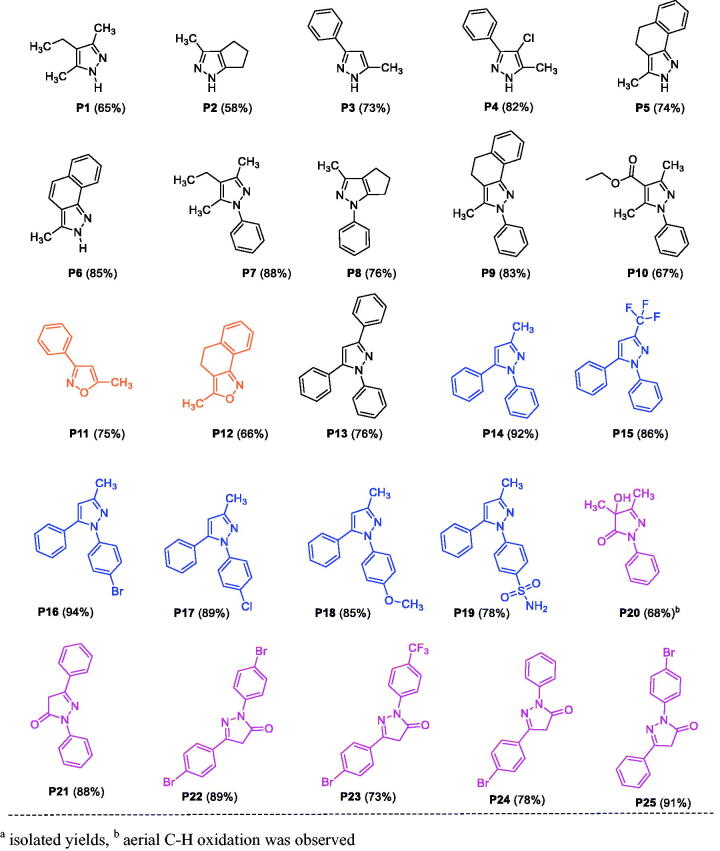
List of substituted pyrazole, isoxazole, and pyrazolone derivatives^a^. ^a^Isolated yields and ^b^aerial C–H oxidation was observed.

In the present work, we synthesised various pyrazoles and isoxazoles, and one set of pyrazolones, to evaluate their anti-tyrosinase, antioxidant and anticancer activities (**P1**–**P25**, Figure 3), by selecting suitable reaction partners with the primary goal of introducing a variety of substituents with diverse electronic and steric properties. A few of these compounds exhibited relatively potent anti-tyrosinase and anti-SC activities *in vitro*. We further explored the mode-of-action of the most-active broad-spectrum compound **P25**, and characterised alterations in physicochemical characteristics across the array of compounds tested (structure–property relationships). Additionally, *in silico* studies were carried out for targets predictions, including docking simulations to predict interactions with identified targets as well as an established non-human target for structurally similar compounds (i.e. mushroom tyrosinase), and predictive assessment of ADMET (absorption, distribution, metabolism, excretion, and toxicity) characteristics.

## Results and discussion

### Chemistry

**Scheme 1.**
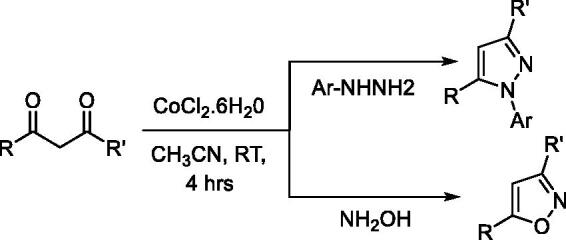
Co-catalysed synthesis of pyrazoles and isoxazoles.

The wide range of biological activities associated with pyrazoles has made them popular synthetic targets. Thus efforts continue to develop new and more versatile synthetic approaches, resulting in expansions of such approaches as: cyclocondensation of hydrazines with carbonyl systems; dipolar cycloadditions of diazo compounds with alkynes; and the reaction of α,β-unsaturated aldehydes and ketones with hydrazines^67^. Some of the emergent procedures involve expensive transition-metal catalysts^90–92^, stoichiometric amounts of harsh reagents (i.e. LiHMDS, *t*BuOLi, *t*BuOK, *n*-BuLi, HCl, and H_2_SO_4_)^93^^,^^94^, or specialised reagents^95^^,^^96^. Cyclocondensation of 1,3-dicarbonyl compounds with substituted hydrazines at relatively higher temperatures offers a direct approach to access substituted pyrazoles. However, unsymmetrical 1,3-dicarbonyl synthon usage often results in the formation of regioisomeric pyrazoles, which are difficult to separate using chromatography^97–100^. Therefore, it is important to develop efficient, selective, and economically viable synthetic methods with potential for large-scale synthesis. Along these lines, we have developed two synthetic approaches for substituting pyrazoles using unsymmetrical or symmetrical dicarbonyl synthons. The first approach entails base metal-catalysed regioselective synthesis of *N*-arylpyrazoles and isoxazoles from unsymmetrical dicarbonyl derivatives. The second approach is based on microwave-assisted synthesis of pyrazoles and pyrazolones. To establish a room-temperature cobalt-catalysed approach for the regioselective synthesis of *N*-arylpyrazoles and isoxazoles, a set of base metal (cobalt and nickel) catalysts and solvents were screened for a reaction between phenylhydrazine and benzoylacetone. Among the tested catalysts and solvents, Co(II)Cl_2_·6H_2_O in acetonitrile produced a quantitative yield of *N*-phenylpyrazole (**P14**) with >99% regioisomeric selectivity. The same reaction conditions afforded other *N*-arylpyrazoles (**P8**, **P9**, **P15**–**P19)** and isoxazoles (**P11**–**P12)** with good yields (Table S6). Structural diversity was achieved by introducing a variety of substituents on the pyrazole ring through the variation of diketone and arylhydrazine synthons (Scheme 1).

**Scheme 2.**
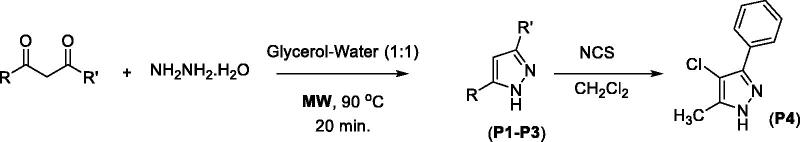
Microwave-assisted synthesis of pyrazoles.

The microwave-assisted approach was found to be more efficient for pyrazoles **P1**–**P6** as compared to the cobalt-catalysed approach^89^. In fact, microwave irradiation facilitates the polarisation of the substrate molecules, accelerating the reactions and providing higher product yields than conventional heating procedures. In organic synthesis, microwave irradiation is considered an ecofriendly route. The microwave-assisted reaction of hydrazine with 1,3-diketones produced the pyrazole compounds **P1**–**P3** in good yields. Moreover, pyrazole **P3** was converted to the corresponding chloropyrazole (**P4**) via chlorination using *N*-chlorosuccinimide^101^ (Scheme 2).

**Scheme 3.**
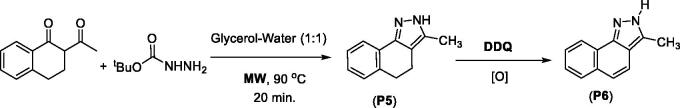
Microwave-assisted synthesis of tricyclic pyrazole **P5** and benznidazole **P6**.

Tricyclic pyrazole **P5** was synthesised from α-tetralone using *tert*-butylcarbazate as a source of hydrazine (Scheme 3); **P5** was further converted to a fully aromatised indazole benznidazole (**P6)** using 2,3-dichloro-5,6-dicyano-1,4-benzoquinone (DDQ) DDQ as an oxidising agent^102^.

**Scheme 4.**
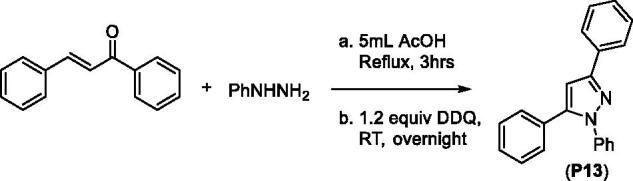
Synthesis of 1,3,5-triphenylpyrazoline from chalcone.

1,3,5-Triphenylpyrazole (**P13**) was obtained from a two-step synthesis via cyclocondensation of chalcone and phenylhydrazine under acid-catalysed reaction conditions followed by Pd-catalysed ring-oxidation^103^ (Scheme 4).

A set of 1,3-disubstituted pyrazolones (**P20**–**P25**) were synthesised from β-ketoesters and phenylhydrazine using our recently reported procedure^89^. The pyrazolone **P20** is a mono-aryl pyrazolone, whereas **P21**–**P25** are diaryl pyrazolones. Moreover, **P22** and **P23** have para-substitutions on both benzene rings.

### Tyrosinase inhibitory activities of pyrazoles, isoxazoles, and 3-pyrazolones P1–P25

**Figure 4.**
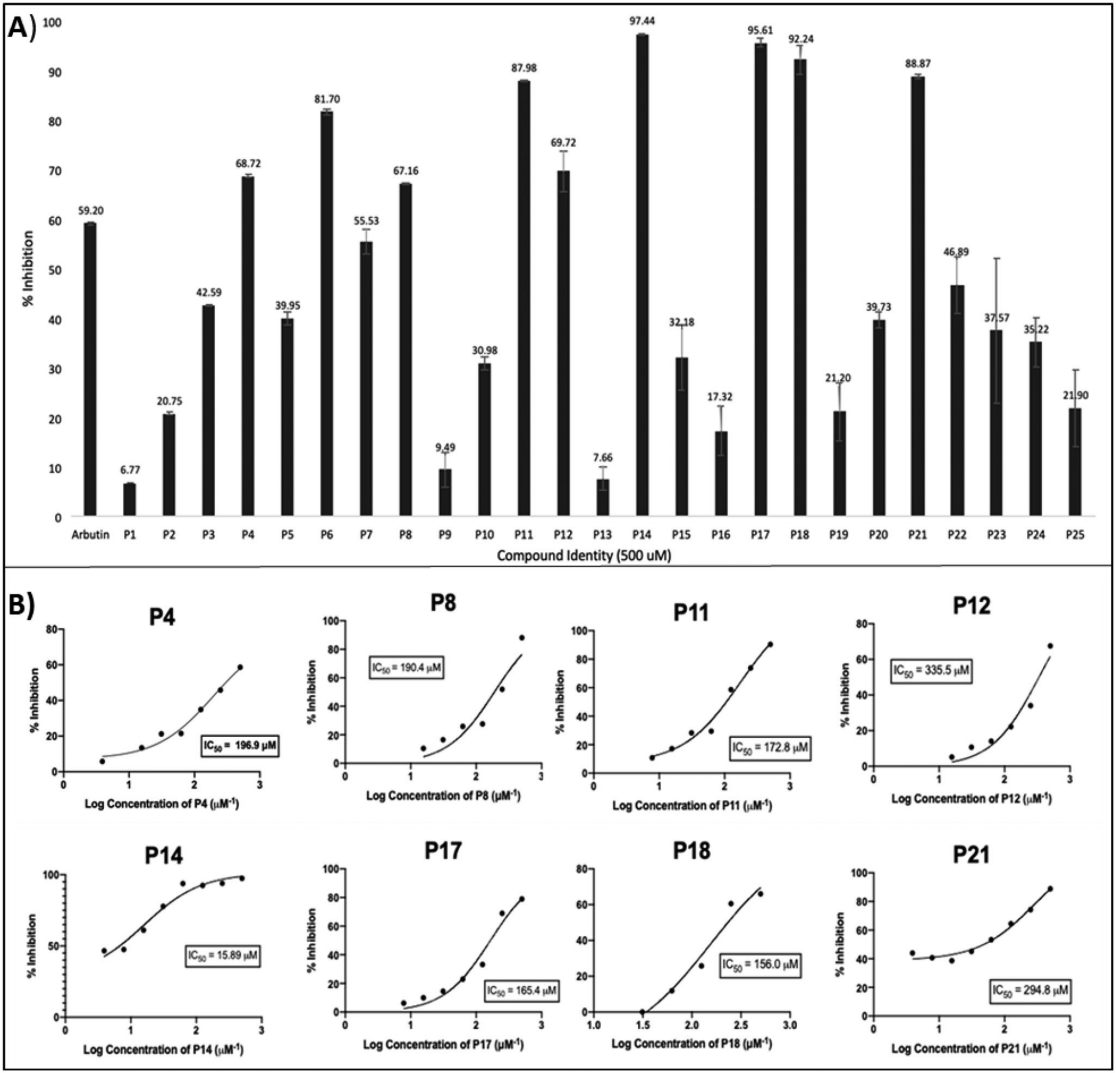
The mushroom tyrosinase inhibition screen of pyrazoles, pyrazolones, and isoxazoles **P1**–**P25** identified several relatively active tyrosinase inhibitors (**P4**, **P8**, **P11**, **P12**, **P14**, **P17**, **P18**, and **P21**). (A) Initial single-concentration tyrosinase inhibition screening for derivatives **P1**–**P25**, with arbutin as a positive control. Ten compounds at a fixed concentration of 500 µM showed comparable or better percentage activity than arbutin. Data are expressed as mean ± SD from at least six independent experiments performed in triplicate. (B) Response vs. concentration curves for the above-listed potent pyrazole and pyrazolone compounds, with calculated 50% inhibitory activity (IC_50_) values. Data are expressed as mean ± SD from at least six independent experiments performed in quadruplicate.

Due to the lack of an available high-resolution crystal structure of human tyrosinase, a mushroom tyrosinase with a highly similar active site protein sequence^41–43^^,^^104^ was adopted for preliminary assessment of tyrosinase inhibition of the test compounds. The initial screening of all test compounds at 500 µM identified 10 compounds exhibiting promising tyrosinase inhibitory activity (cf. Figure 4), including **P4** (68.7 ± 0.4%), **P6** (81.7 ± 0.7%), **P8** (67.2 ± 0.2%), **P11** (88.0 ± 0.3%), **P12** (69.7 ± 5.0%), **P14** (97.4 ± 0.2%), **P17** (95.6 ± 1.1%), **P18** (92.2 ± 3.7%), and **P21** (88.9 ± 0.4%). Inhibition was greater than for arbutin (59 ± 0.4%), which was used as a positive control. These compounds were further tested at various concentrations, from which half-maximal inhibitory concentration where 50% of cells are dead (IC_50_) values could be calculated (Figure 4(B), Table S1). Eight of the 25 synthesised compounds (**P4**, **P8**, **P11**, **P12**, **P14**, **P17**, **P18**, and **P21**) exhibited potent-to-moderate tyrosinase inhibitory activities, with estimated IC_50_ values ranging from 16 µM to 340 µM, among which **P14** exhibited the highest tyrosinase-inhibiting potency. Notably, the molecular weight of 11 of the evaluated compounds is relatively low, at only 200 or less (see Tables S2 and S3), including **P4**, **P8**, **P11**, and **P12** among the compounds listed above. For all other tested compounds, the estimated IC_50_ value was either greater than 500 µM, or no activity was discerned (“not active” (N.A.)) (Table S1). The pyrazole derivative **P14** (IC_50_ 15.9 ± 1.2 µM), one of several relatively close analogues of celecoxib tested, was the most potent tyrosinase inhibitor (Figure 4(B)), exhibiting activity greater than the positive comparators arbutin (**IC_50_** 91 µM) and kojic acid (**IC_50_** 31 µM) (Table S1).

Over the years, the mechanism-of-action of tyrosinase inhibitors has been experimentally linked to one of five main modes: competitive inhibition; reverse conversion (of ortho-dopaquinone to l-DOPA, i.e. a shift to reductase mode); non-specific enzyme inactivation (NE); specific, irreversible enzyme inactivation (SIE), in some instances more specifically entailing “suicide inhibition”; and specific reversible enzyme (SRE) inactivation (pseudo-irreversible inhibition)^27^. Moreover, an extremely subtle relationship exists between antioxidant defence systems and tyrosinase inhibition^105^. However, **P14** (15.9 ± 1.2 µM), the most potent tested anti-tyrosinase compound, was inactive in the DPPH antioxidant assay. It is worth mentioning that the screening of both tyrosinase inhibition and antioxidant activity in prospective studies requires further exploration.

**Figure 5.**
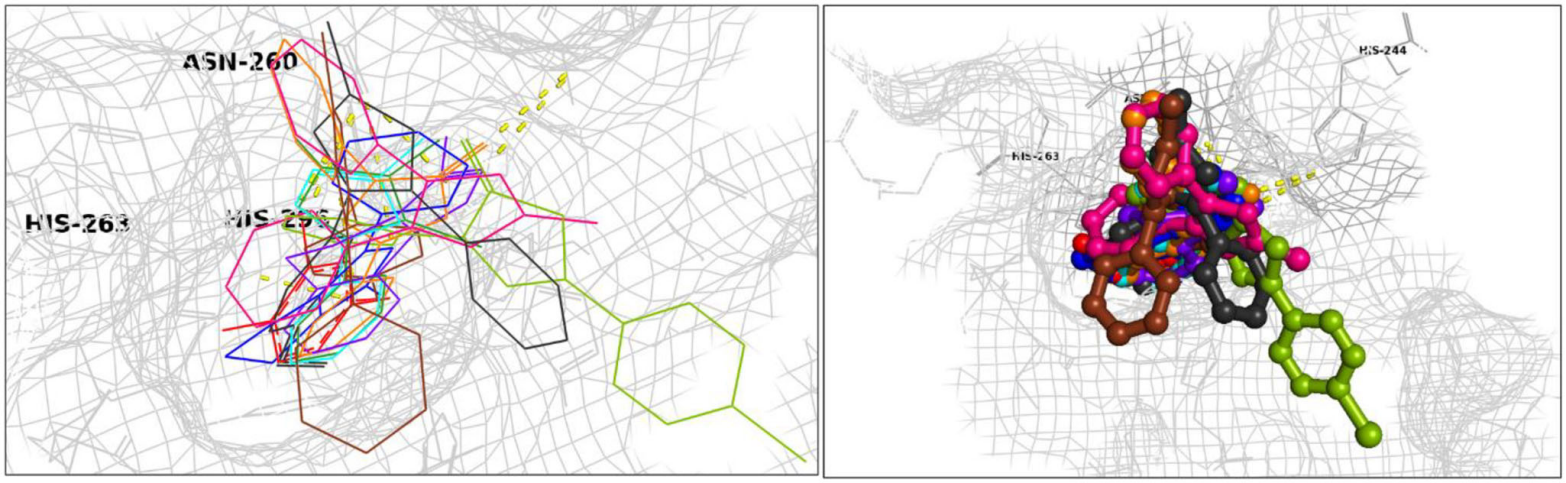
Docking interactions and poses between compounds (**P4**, **P8**, **P11**, **P12**, **P14**, **P17**, **P18**, **P21**, **P25**, and kojic acid) and the target mushroom tyrosinase protein structure adopted from PDB 2Y9X. The target protein is highlighted in line format with light grey colour (A, B). The key interacting binding site amino acids are shown in black colour, while several hydrogen bonds are observed between functional groups of particular compounds and the enzyme amino acids, as summarised in Table 1, with interaction distances ranging from 2.16 Å to 3.26 Å. *Left:* Ligands in line and stick representation. *Right:* Ligands in ball and stick representation. Binding positions are proximal to the two copper ions (Cu^2+^) (not shown) in the active site.

Exploiting the availability of a high-quality X-ray structure of an inhibitor-bound tyrosinase (more specifically, a tyrosinase from *Agaricus bisporus* in deoxy-form, in complex with an unidentified lectin-like subunit, and with the inhibitor tropolone bound in its catalytic site; PDB ID 2Y9X), we carried out docking simulations to predict binding poses and specific molecular recognition interactions of **P1**–**P25** within the catalytic pocket. Based on binding energies estimated with these simulations (cf. Refs.^106^^,^^107^) the eight most active of the synthesised compounds (**P4**, **P8**, **P11**, **P12**, **P14**, **P17**, **P18**, and **P21**) are predicted to bind with comparable or higher affinity than the inhibitor bound in the complex for which the PDB structure (2y9x) was obtained (tropolone: −5.90 kcal/mol) as well as one of our positive control ligands (kojic acid: −5.80 kcal/mol) ligands (Table 1). Detailed scrutiny of binding poses for the ligands predicted to bind with the highest affinity (Figure 5) indicates involvement of diverse interactions, including conventional *H-*bonds and various pi bonds at distances ranging 2.16 Å and 3.96 Å, as well as hydrophobic interactions. The comparative analysis is predicted by the simulation to exhibit relatively high binding affinities, as shown in Table 1.

**Table 1.** Docking interaction energies for the active tyrosinase inhibitory compounds.

Compd. ID	2Y9X Energy values (kcal/mol)	H bond forming residues at 2y9x	pi–pi stacked or edge-face (T-shaped)
NL tropolone	–5.90	HIS60, VAL217	
CL kojic acid	–5.80	HIS296, HIS 259, HIS 263, ASN260	His263
**P4**	–6.50	ASN260	His263, Ser282
**P8**	–6.70	ASN 260	His263
**P11**	–6.10	ASN260	His263, Ser282
**P12**	–7.20		His263, His259, Ser282
**P14**	–6.00	–	Phe264
**P17**	–6.40		Phe264
**P18**	–6.70		His244
**P21**	–6.70	HIS244	Phe264, His263

NL: natural ligand; CL: clinical ligand.

From an SAR standpoint, eight of the 25 test compounds showed moderate to potent tyrosinase inhibitory activity (≥70% inhibition). The 3-methyl-1,5-diaryl pyrazoles **P14**, **P17**, and **P18** exhibited considerably greater potencies as compared to other tested compounds, which may be attributed to aryl substituents on positions 1 and 5 of the pyrazole moiety, and suggests these positions of the pyrazole ring as well as the nature of the substituent may be critical for the activity of the compound (see Table S1). Although **P14**, **P17**, and **P18** showed similar % inhibition, **P14** exhibited a substantially lower IC_50_ value (15.9 ± 1.2 µM), perhaps suggestive that the presence of para-substitutions on the *N*1-phenyl may not be favourable for binding. Moreover, compound **P6 (**a benznidazole), **P11** and **P12** (both isoxazoles), and **P21** (a pyrazolone) displayed good to moderate anti-tyrosinase activities, although clearly, **P6** and **P12** are not overtly similar structurally. On the other hand (cf. Figure 2), the activities of pyrazoles **P3** and **P5** were significantly increased upon isosteric replacement of their pyrazole ring moiety with isoxazole (**P11** and **P12**, respectively). Unlike other pyrazolones tested, **P21** lacks substituents at the para-position of the benzene rings, suggesting that this might have accounted for the observed effect.

### *In vitro* assessment of antiproliferative activity and preliminary structure–activity relationships

The antiproliferative effects of all the synthesised compounds (**P1**–**P25**), alongside cisplatin and celecoxib (serving as positive controls), were investigated in cultured human cutaneous melanoma (A375 and SK-MEL-28) and cutaneous SCC (A431 and SCC-12) cell lines by 3-(4,5-dimethylthiazol-2-yl)-2,5-diphenyl tetrazolium bromide (MTT) assay. HaCaT immortalised keratinocytes were used as control noncancerous cells. As described earlier^108^^,^^109^, antiproliferative and cytotoxic activities were assessed for all the indicated five human skin-derived cell lines.

Some of the test compounds exhibited antiproliferative activities of varying degrees against the different human SC cell lines, comparing to their effect on noncancerous control cells (Table 2). Twelve compounds (**P3**, **P9**, **P12**, **P14**, **P16**, **P17**, **P18**, **P19**, **P22**, **P23**, **P24**, and **P25**) exhibited anti-SC activity against at least one among the spectrum of cancer cell lines tested, with markedly differing potencies. The most active compound, **P25**, displayed good-to-moderate potency across all four SC cell lines (A431: IC_50_ = 3.7 µM, SKMEL-28: IC_50_ = 7.6 µM, SCC-12: IC_50_ = 12.2 µM, and A375: IC_50_ = 14.3 µM). Importantly, **P25** was also selectively active against cancer cells (A431 and SCC-12) vs. noncancerous control (HaCaT) cells, albeit with modest selectivity indexes (SIs) of only about 8.2 and 2.5, respectively. These SI values are, however, better than for cisplatin (SI of 0.8 and 0.4), a clinical anticancer agent with numerous side-effects^110^. Of potential interest for downstream investigation, **P25** exhibited better potency against non-melanoma A431 cells than SCC-12 cells (IC_50_ 3.7 µM vs. 12.2 µM, respectively). The next-most-active compound, **P22**, exhibited only moderate to marginal activities across the non-melanoma (A431: IC_50_ 13 µM and SCC-12: IC_50_ 19 µM) and melanoma (A375: IC_50_ 29 µM and SK-MEL-28: IC_50_ 31 µM) lines. Compounds **P18** and **P23** exhibited modest activities with some observed selectivity for cancerous over noncancerous cells and between cancerous lines.

**Table 2.** Cytotoxicity of pyrazole (celecoxib analogs), isoxazole, pyrazolone, and positive control compounds **P1**–**P25** (structures, Figure 3) against cells of human cutaneous melanoma and non-melanoma skin cancer lines relative to standard control noncancerous immortalised HaCaT cells.

Compd. ID	Cell lines, IC_50_ values in μM, and selectivity index (SI)				
	Melanoma		Non-melanoma		Control
	A375	SKMEL-28	GFP-A431	SCC-12	HaCaT
**P1**	47.7 ± 4.2	42.8 ± 0.3	41.6 ± 0.8	257.3 ± 18.4	60.5 ± 3.9
**P2**	54.3 ± 1.6	42.6 ± 1.5	43.9 ± 2.1	245.6 ± 37.3	44.2 ± 1.1
**P3**	54.8 ± 2.7	59.1 ± 4.6	27.2 ± 0.9 (1.6)	546.5 ± 20.7 (0.1)	42.1 ± 3.4
**P4**	58.4 ± 4.5	62.3 ± 2.7	70.8 ± 6.2	215.8 ± 13.4	76.1 ± 0.0
**P5**	65.3 ± 4.2	130.9 ± 2.4	42.1 ± 3.1 (6.3)	77.6 ± 1.7 (3.4)	265.4 ± 4.3
**P6**	113.5 ± 3.3	47.4 ± 1.2	69.8 ± 7.5 (2.1)	49.9 ± 3.4 (3.0)	147.2 ± 0.8
**P7**	53.3 ± 4.2	46.0 ± 1.0	47.8 ± 2.0	43.0 ± 2.1	59.2 ± 3.5
**P8**	55.3 ± 3.8	180.9 ± 4.7	72.2 ± 5.2	120.0 ± 8.5	44.5 ± 1.4
**P9**	45.2 ± 2.0	40.8 ± 1.0	28.0 ± 3.6 (1.6)	28.8 ± 1.4 (1.5)	43.6 ± 1.4
**P10**	55.3 ± 2.5	43.7 ± 2.7	195.5 ± 3.8	49.1 ± 2.0	68.0 ± 1.2
**P11**	57.8 ± 6.5	34.5 ± 1.2	43.7 ± 2.7 (2.6)	44.6 ± 1.3 (2.6)	115.5 ± 10.1
**P12**	73.5 ± 3.8	24.0 ± 2.0	55.5 ± 3.5	32.6 ± 2.1	32.6 ± 2.4
**P13**	93.4 ± 6.4	153.5 ± 2.9	181.4 ± 4.7	52.8 ± 2.2	91.6 ± 4.1
**P14**	25.8 ± 2.7	33.3 ± 1.7	17.8 ± 0.5 (2.0)	42.5 ± 2.8 (0.8)	35.1 ± 2.8
**P15**	58.5 ± 3.4	36.0 ± 3.9	42.9 ± 1.0	98.0 ± 5.5	53.7 ± 3.1
**P16**	46.0 ± 1.0	39.1 ± 2.3	21.0 ± 1.3 (2.6)	19.6 ± 2.0 (2.8)	54.2 ± 3.6
**P17**	44.5 ± 3.0	34.2 ± 3.9	19.7 ± 0.8 (3.7)	23.4 ± 1.7 (3.1)	72.4 ± 6.0
**P18**	48.0 ± 2.1	19.3 ± 2.4	25.9 ± 0.9 (2.0)	21.9 ± 0.2 (2.3)	49.6 ± 1.5
**P19**	47.1 ± 4.2	40.5 ± 3.0	7.6 ± 0.6 (7.5)	97.9 ± 5.6 (0.6)	57.0 ± 1.3
**P20**	109.8 ± 15.4	50.0 ± 4.3	119.6 ± 2.6	57.3 ± 0.2	47.7 ± 2.7
**P21**	67.8 ± 2.3	42.7 ± 4.0	58.6 ± 2.3	37.8 ± 3.2	50.1 ± 6.5
**P22**	28.7 ± 1.2	31.3 ± 2.4	12.6 ± 1.6 (3.8)	18.7 ± 1.4 (2.6)	47.6 ± 1.6
**P23**	31.2 ± 1.5	27.4 ± 2.3	23.9 ± 1.0 (1.7)	21.7 ± 1.9 (1.9)	40.5 ± 1.5
**P24**	56.4 ± 1.7	54.1 ± 1.9	18.6 ± 0.9 (3.3)	140.8 ± 3.9 (0.4)	60.4 ± 3.2
**P25**	14.3 ± 0.9	7.6 ± 0.6	3.7 ± 0.5 (8.2)	12.2 ± 0.6 (2.5)	30.5 ± 2.0
Celecoxib^a^	26.8 ± 2.7	11.4 ± 2.4	7.4 ± 0.6 (6.7)	44.1 ± 1.1 (1.1)	49.8 ± 8.2
Cisplatin^b^	1.49 ± 0.44	14.2 ± 0.24	7.7 ± 0.3 (0.4)	4.4 ± 0.2 (0.8)	3.4 ± 0.4

SI: a selectivity index was calculated as the IC_50_ value in the respective normal cells (HaCaT keratinocytes) divided by the IC_50_ for the specified cancer cell lines. IC_50_: expresses the compound concentration capable of inhibiting the maximal measured cell proliferation (assessed as cell viability of the cultured cells) by 50%.

^a^Celecoxib and ^b^cisplatin (a known anticancer agent) served as positive controls. Data are expressed as mean ± SD from the response vs. concentration curves of at least six independent experiments carried out in quadruplicate.

The anticancer activities of nonsteroidal anti-inflammatory cyclooxygenase inhibitors (“NSAIDs”), including aspirin (modestly COX1-selective) and celecoxib (a moderately COX2-selective pyrazole), prompted us to synthesise several celecoxib analogues and test their activities alongside the parent molecule in our panel of cutaneous cancerous cell lines^111–114^. Celecoxib exhibited modest to marginal activity against the three cancerous cell lines tested (non-melanoma A431: IC_50_ 7.4 µM, SI 6.7; melanoma SK-MEL-28: IC_50_ 11.4 µM, SI 4.4 and A375: IC_50_ 27 µM, SI 1.9). Amongst the celecoxib analogues (**P14**–**P19**), **P15** was the only inactive compound, while the others showed marginal to moderate potencies. Compound **P19** was reasonably selective with modest potency against non-melanoma A431 cells (IC_50_ 7.6 µM, SI 7.5), values comparable to those obtained for celecoxib (vide supra) and cisplatin (IC_50_ 7.7 µM, but with no selectivity (SI 0.8)). However, **P19** exhibited poor activity against cells of the other nonmelanoma line (SCC-12), with essentially no selectivity, and also poor activity against cells of the two melanoma lines (A375 and SK-MEL-28). Marginal activity was observed for the remaining compounds, in most instances with poor selectivity against cancerous vs. control cells. However, **P5** and **P6** showed some selectivity against non-melanoma vs. non-cancerous control HaCaT cells.

From a screening stage structure–activity relationships (SARs) standpoint, compounds of greatest interest are pyrazoles that are celecoxib analogues, maintaining (**P15**) or replacing (**P14** and **P16**–**P19**) the trifluoromethyl (CF_3_, powerfully electron-withdrawing) at position 3 of the pyrazole heterocycle with a methyl group (CH_3_; moderately electron-donating), and with omission (**P14** and **P15**), substitution (**P16**, **P17**, and **P18**), or maintenance (**P19**) of the sulphonamide (SO_2_NH_2_) moiety. Only **P25** exhibited activity and selectivity of some interest among the four pyrazolones. Still, no clear SAR conclusions can be drawn from these results, and non-analogous binding poses might easily be a confounding contributor. Moreover, it should be noted that the effects of various substituents on lipophilicity and, thus, cell penetrability must be considered in future studies. Given that no clear SAR emerged from this initial series of anti-proliferative screens, more insight into the relationship between the structural patterns and the activities of these compounds was sought by exploring their anti-tyrosinase (above) and antioxidant potentials, given that multi-component action might also confound interpretation of phenotypic results at this stage.

### Colony formation, scratch wound healing, and apoptotic machinery investigations for phenotypic assessment of the most-active broad-spectrum anti-SC agent, pyrazolone P25

**Figure 6.**
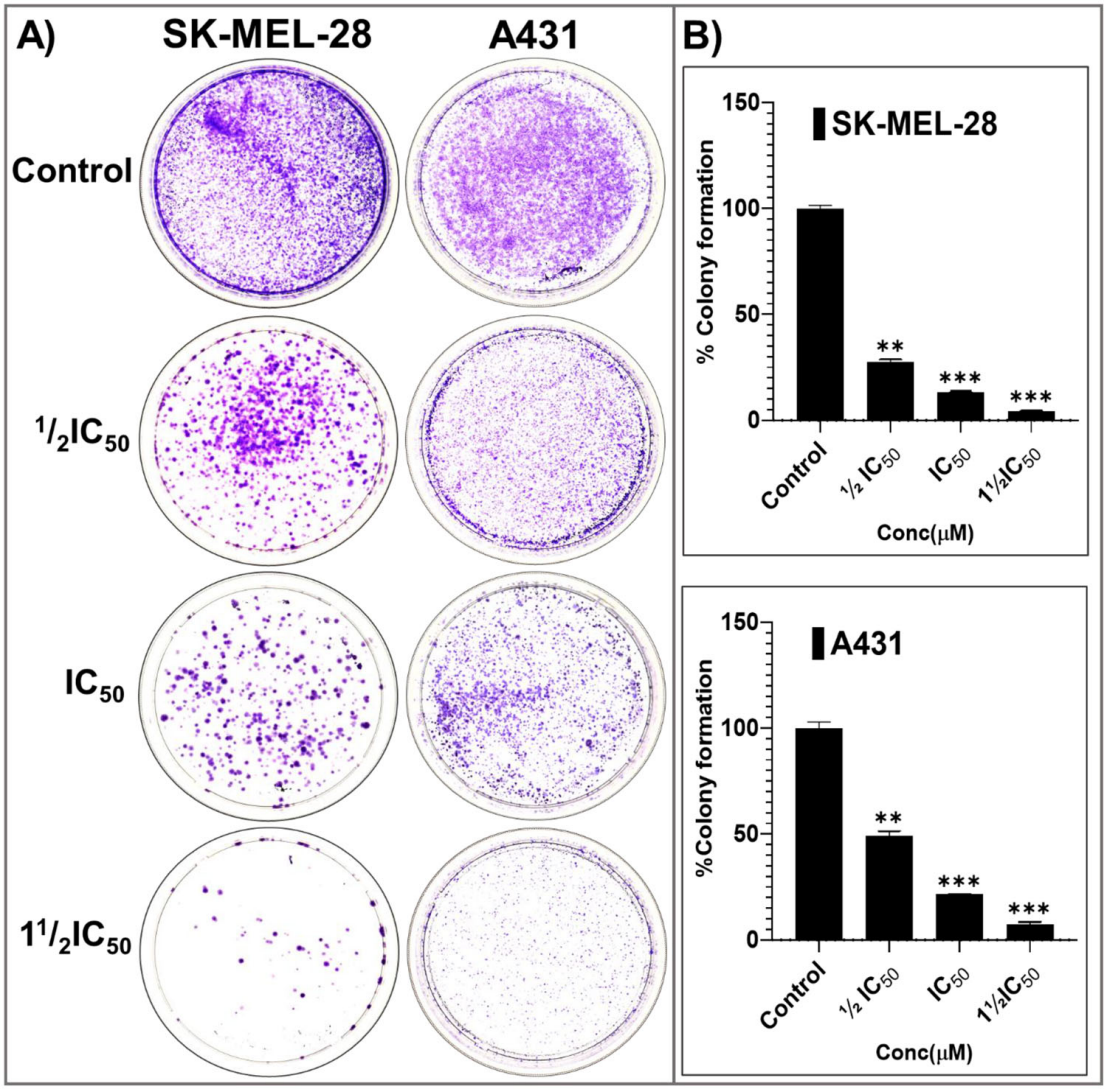
The potent broad-spectrum anticancer hit compound P25 significantly inhibits colony formation capacity in A431 (A) and SK-MEL-28 (B) cells after 14 days. The percentage decrease in colony formation was concentration-dependent (0, ½IC_50_, IC_50_, and 1½IC_50_ obtained from the antiproliferation assay) and was comparable in A431 (C, D) and SK-MEL-28 (E, F) cells. The data expressed in the bar graphs represent the mean ± SD in the **P25-**treated group expressed as a percentage relative to the untreated control group. Data are from three independent experiments performed in quadruplicate. Statistical significance was assessed using one-way ANOVA and Tukey’s multiple comparison tests; **p < 0.01, and ***p < 0.001 were considered significant.

**Figure 7.**
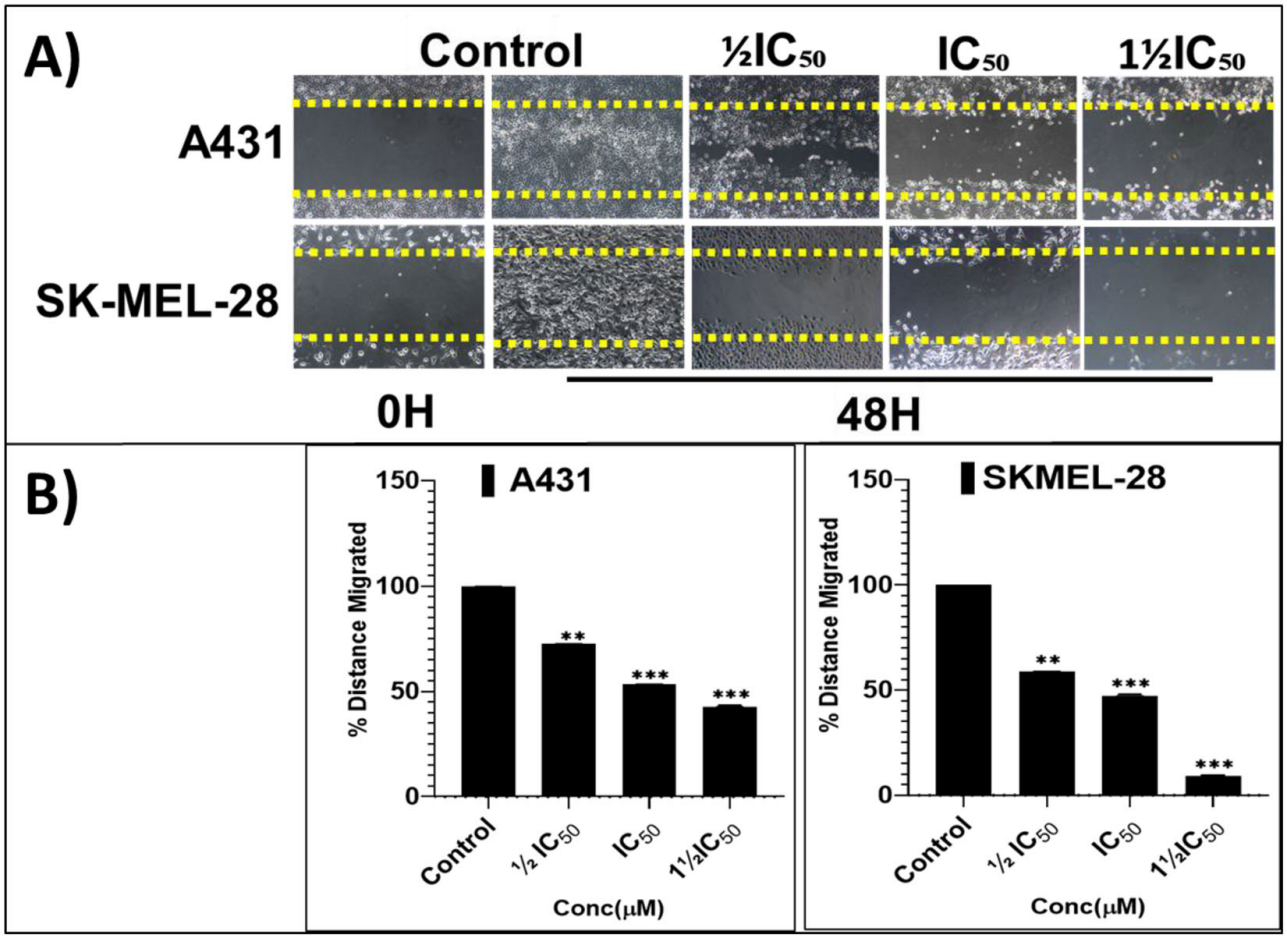
The screening hit compound P25 potently and dose-dependently (0, ½IC_50_, IC_50_, and 1½IC_50_) inhibits the migration of A431 (A; top panel) and SKMEL-28 (A; bottom panel) cells into the cell-free scratched-wounded areas of a confluent cell monolayer. This suppression of A431 and SKMEL-28 cells scratch wound closure in a concentration-dependent manner was significant. The bar graphs (B) represent the mean ± SD of covered scratch-wound area values (expressed as the migrated distance from 0-time point of scratch) after 48 h. They are expressed as a percentage of wound area at 0 h filled by migrated cells by the end of the experiment, vs. the same indicator for untreated control cells, from three independent experiments conducted in triplicate. Statistical significance was assessed using one-way ANOVA and Tukey’s multiple comparison tests; **p < 0.01, and ***p < 0.001 were considered significant.

From the cell proliferation and viability experiments, the most-active and somewhat-selective broad-spectrum compound, pyrazolone **P25**, was selected for further characterising mode(s)-of-action phenotypically, in an attempt to identify molecular-level contributors to, and determinates of, efficacy, potency, and all-important cancer-directed selectivity. Towards these ends, anti-proliferative effects on colony formation, inhibition of scratch wound healing, and apoptosis-promoting effects were assessed. Long-term (14-days) treatment with various concentrations of **P25** (0, ½IC_50_, IC_50_, and 1½IC_50_) revealed significant concentration-dependent reductions in the relative percentage of colonies formed by A431 and SKMEL-28 cells, as compared to untreated controls (Figure 6). Further, a scratch-wound assay was carried out to scrutinise the inhibitory effects of **P25** on the migration of A431 and SK-MEL-28 cells into cell-free scratch-wounded areas after 48-h treatments. **P25** significantly and dose-dependently decreased the scratch wound areas in both A431 (non-melanoma) and SK-MEL-28 (melanoma) cultures as compared to untreated control cells (Figure 7).

Apoptosis is an adaptive cellular mechanism that ensures replicating cells to maintain the integrity of their sourced DNA. Apoptosis is often impaired or dysfunctional in cancerous cells; consequently, the ability of a compound to restore normalcy of apoptotic triggering (and other programmed cell death pathways) constitutes an essential aspect in the screening of potential anticancer agents, and is a key prospective source of targeting selectivity (i.e. cancerous vs. non-cancerous cells and tissue)^115^. As **P25** significantly decreased cell viability and colony formation, apoptotic mechanisms of cytotoxicity were investigated by assessing the potential of this compound to modulate the intrinsic and extrinsic apoptosis pathways and function in A431 and SK-MEL-28 cell lines. The key apoptosis-related markers assessed (by western blot analysis) included the pro- and cleaved forms of caspase-3 (a member of the executioner apoptotic caspase family along with caspases 6 and 7) and caspase-9 (a member of the initiator apoptotic caspase family along with caspases 8 and 10), as well as cleaved vs. uncleaved poly(ADP-ribose) polymerase (PARP). Particular changes in these markers of pathway function are often positive indicators that a compound could serve for the successful development of an anticancer agent (more commonly, serving as a lead for further optimisation)^116^^,^^117^.

**Figure 8.**
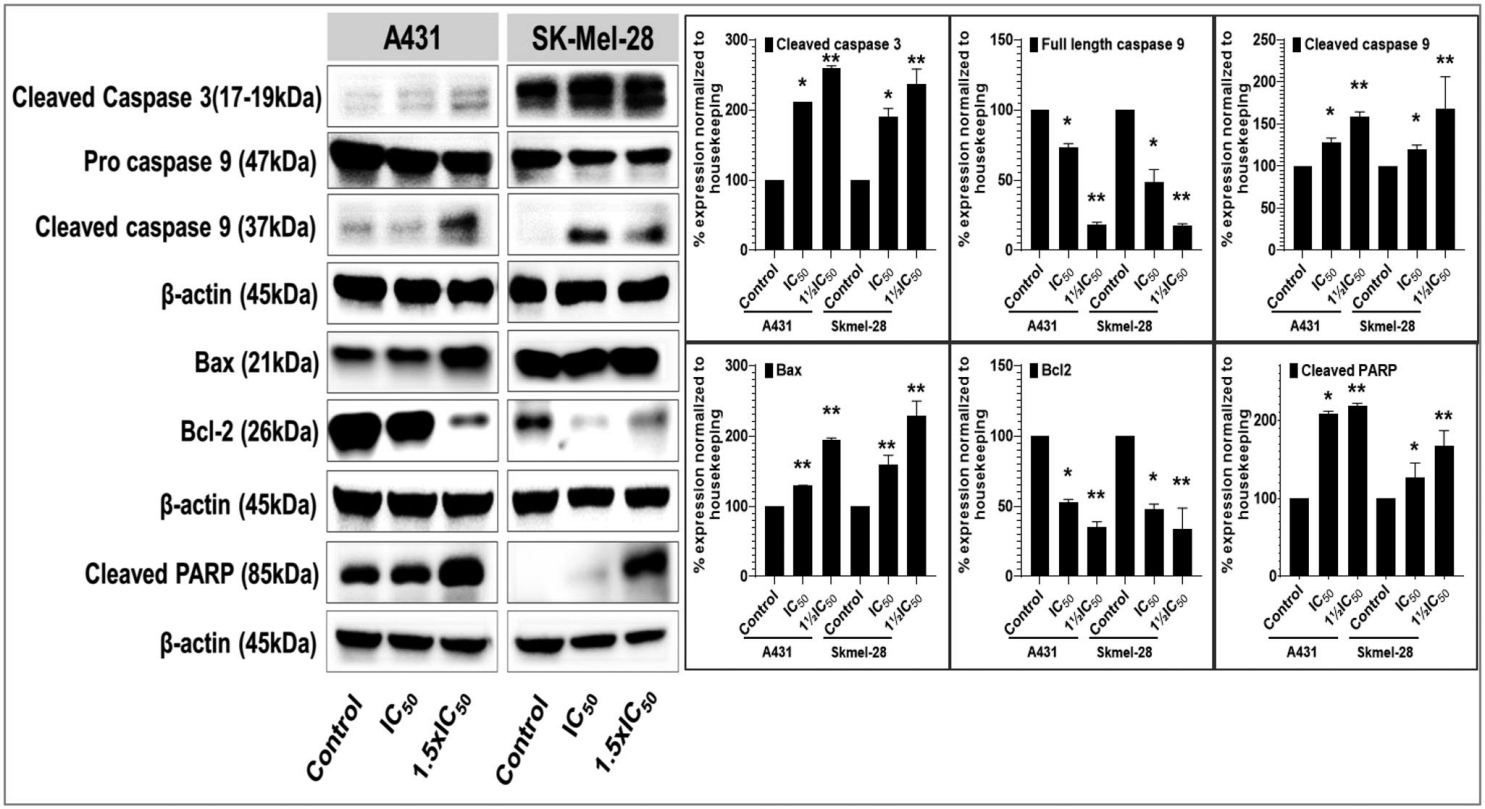
Compound P25 induces apoptosis by activating the extrinsic and intrinsic apoptotic pathways in cutaneous melanoma (SK-Mel-28) and non-melanoma (A431) cancer cells in culture. The blots (left) document a concentration-dependent effect (0, ½IC_50_, IC_50_, and 1½IC_50_; IC_50_ refers to the cell viability result, cf. Table 2) of protein expression levels of markers of apoptosis, including pro- and cleaved caspase-3, and caspase-9, and of cleaved PARP, after 48 h of treatment. The data shown are representative immunoblots from three independent experiments with similar results. β-actin was used as a loading control. At right, protein levels as normalised (mean ± SD of relative quantitative density values) are expressed as a percent increase or decrease vs. untreated cells (control) in the bar graphs. Statistical significance was assessed using one-way ANOVA and Bonferroni’s multiple comparison tests; *p < 0.05 and **p < 0.01, were considered significant.

Treatment of A431 and SK-MEL-28 cells with **P25** resulted in significant and concentration-dependent activation of apoptosis, as evidenced by substantially increased levels of activated caspase-3, and caspase-9, and of cleaved PARP, as compared with untreated controls (Figure 8). Additionally, the involvement of the mitochondria, specifically with respect to intrinsic apoptotic pathways, was observed from the dose-dependent amplified expression of pro-apoptotic Bax with corresponding decreases in anti-apoptotic Bcl-2 protein levels in both A431 and SK-MEL-28 **P25**-treated cells as compared with untreated controls^118^. Therefore, these data strongly support that **P25** induces the apoptosis of A431 and SKMEL-28 SC cells via the intrinsic mitochondrial apoptotic pathways associated with PARP activation (extrinsic pathways).

### Antioxidant activity as assessed by DPPH radical scavenging

In cancers, increased ROS generation and severe perturbation of cellular redox balance have been associated with enhanced tumour growth, genetic instability, and adaptive responses that promote malignant phenotypes in cancerous cells^59^. However, antioxidant or ROS buffering as a component activity of a therapeutic agent for treating certain chronic skin diseases, notably those involving inflammation or cancers, may confer greater efficacy^119^.

**Figure 9.**
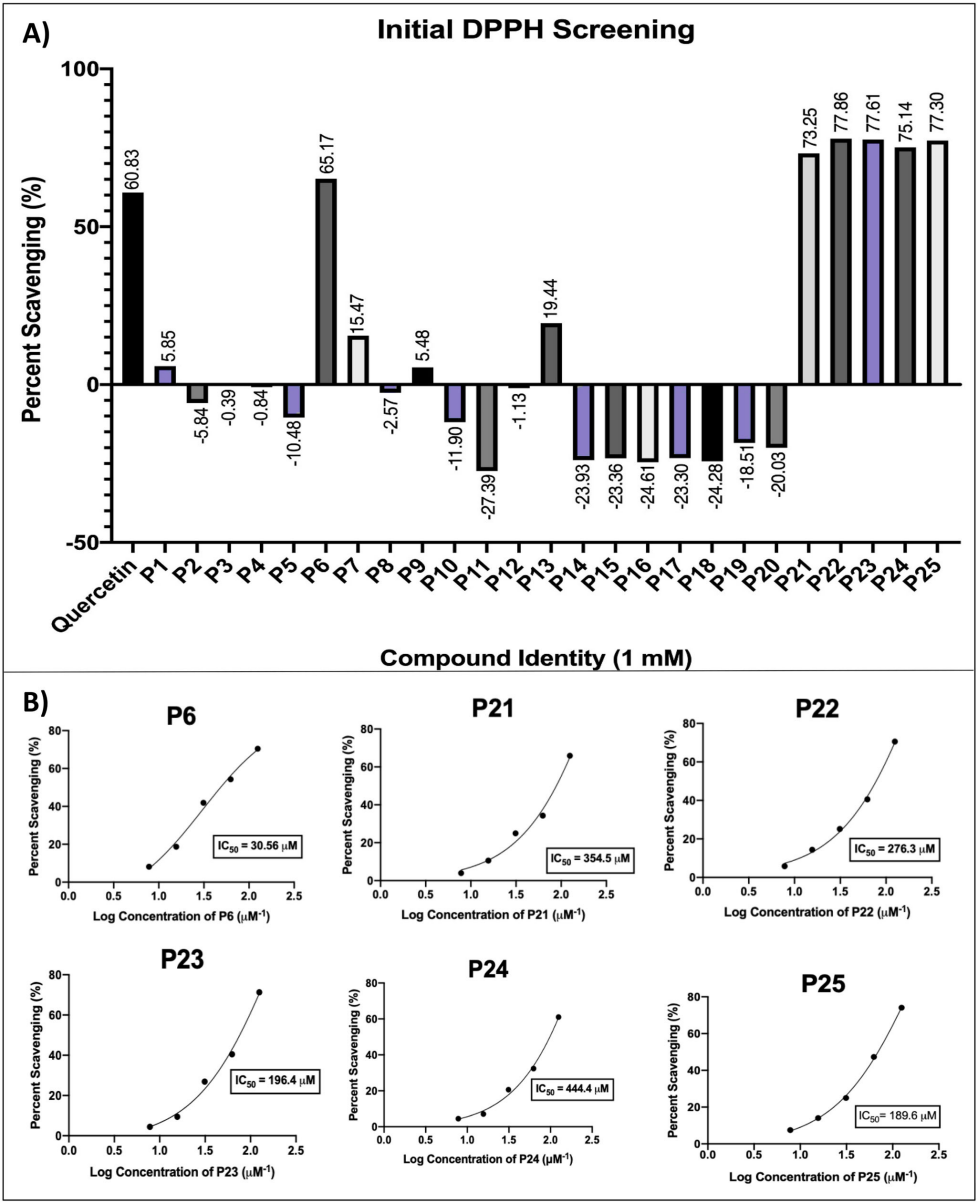
The 2,2-diphenyl-1-picrylhydrazyl (DPPH) anti-oxidant (radical scavenging) activity of compounds **P1**–**P25** identifies modest to moderately potent antioxidant activity of one tricyclic pyrazole (a naphtho[1,2-*c*] pyrazole) and several pyrazolones. (A) Concentration-dependent radical scavenging activities, compared to quercetin as a positive control. (B) IC_50_ values for appreciably active compounds. Data are expressed as mean ± SD from at least six independent experiments performed in quadruplicate.

The antioxidant potential of the test compounds was explored via the DPPH assay, a rapid, simple, inexpensive, and widely used method to measure the ability of compounds to act as free-radical scavengers or hydrogen donors. Initial screening of all 25 compounds at 1 mM revealed compounds **P6**, **P21**, **P22**, **P23**, **P24**, and **P25** to be the most active, with a scavenging effect ranging from 65 to 75%, comparing favourably to quercetin as a positive control (60%; Figure 9). All of other compounds exhibited lesser scavenging activities than quercetin. The activities of all compounds were further investigated as a function of concentration, with further comparisons to the known antioxidants ascorbic acid and butylated hydroxytoluene (BHT). The six compounds listed above exhibited antioxidant IC_50_ values ranging from 31 to 440 µM (Figure 9; Table S4), whereas the activities of the remaining 19 compounds were negligible. The tricyclic naphtho[1,2-*c*]pyrazole **P6** (IC_50_ 30.6 ± 2.1 µM) was the most potent, exhibiting antioxidant activity comparable to that of ascorbic acid (IC_50_ 25.2 ± 1.4 µM), and far better than of BHT (IC_50_ 285 ± 7 µM). From a preliminary SAR perspective, except for **P6** (pyrazole), all of the other active compounds showing appreciable antioxidant activities were pyrazolones, which are able to undergo keto-enol and imine-enamine tautomerism. Among the three possible tautomers, rapid exchange can occur between OH and NH tautomers, versus a slow equilibrium involving the CH (parent pyrazolone) tautomer. Possibly the NH (enamine) and OH (enol) tautomeric forms are responsible for the antioxidant effect of pyrazolones **P21**–**P25**, via sequential electron-transfer proton-transfer (SETPT), hydrogen atom transfer (HAT) or sequential proton-loss electron-transfer mechanisms (SPLETM)^85^. Compound **P20**, the only pyrazolone of those tested found to exhibit no appreciable antioxidant activity, includes a C5-alkyl rather than aryl substituent and a quaternary C4 ring carbon. This observation suggests that the presence of 2,5-diaryl substitution and/or absence of a quaternary carbon in the pyrazolone ring is essential for retaining antioxidant activity, as exemplified in previous reports^119–121^. The tricyclic **P6** is the only pyrazole that also emerged as a free-radical scavenger, and was the most active of all compounds assessed in this assay. Its high antioxidant activity is likely due to its free N–H moiety in conjunction with its highly conjugated fused aromatic rings, which can readily support one or more antioxidant mechanisms (i.e. SETPT, HAT, and SPLETM, vide supra). The inactivity of **P5** in this assay, despite its close structural analogy to **P6**, is concordant with these assertions.

### *In silico* target(s) prediction, molecular docking, and biopharmaceutic properties prediction

*In silico* ligand-based target prediction (so-called “target fishing”) is increasingly proving its suitability in predicting pertinent protein targets underlying observed phenotypic activities^122^. Accordingly, we used the SwissTargetPrediction server, which exploits molecular similarities between test compounds and large, curated libraries of compounds with known and well-document activities (≥280 000 active compounds, and ≥440 000 documented interactions^123^) and generates data suggesting the probability that a particular molecule will interact with a given protein as a target^123–126^. Of the thousands of included targets, all 25 of the synthesised compounds (**P1**–**P25**) displayed molecular interaction similarities with only 11 molecular targets, including four kinases (mitogen-activated protein kinase 14 (MAPK14/p38α), vascular endothelial growth factor receptor 1 (VEGFR1), tyrosine-protein kinase FLT3, and tyrosine protein kinase JAK1), two oxidoreductases (COX-1 and COX-2), and four other targets including alpha-2B adrenergic receptor (α_2b_AR), carbonic anhydrase II, a cytosolic heat shock protein isoform (Hsp90-α), and cytochrome P450s (aggregate), as well as tyrosinase (cf. Table 3 and discourse above).

**Table 3.** Predicted binding energies of compounds to molecular targets, as compared to their crystal-structure-native and commercial ligands. The bold values are the significant binding affinities.

Ligands	Kinases				Cyclooxygenases		Other targets				
	MAPK 14/p38α	VEGFR1	Tyrosine protein kinase FLT3	JAK1	COX-1	COX-2	Alpha-2B AR	Carbonic anhydrase II	HSP 90α	Cytochrome P450	Tyrosinase
**Native**	**–13.4**	**–10.7**	**–7.7**	**–6.4**	**–10.6**	**10.3**	**–8.5**	**–10.4**	**–7.2**	**–9.6**	**–5.90**
**Clinical**	PH–797804 **–8.2**	Sorafenib **–10.7**	Gilteritinib –**7.7**	Tofacitinib **–8.6**	Celecoxib **–10.0**	Celecoxib **–11.0**	Imiloxan **–7.8**	Dorzolamide **–7.2**	Geldanamycin **–7.6**	Clarithromycin **–10.2**	**Kojic acid** **–5.80**
**1**	–5.7	–5.6	–5.0	–5.1	–5.4	–5.6	–5.9	–4.9	–5.2	–5.8	–5.40
**2**	–6.5	–6.2	–5.8	–5.6	–5.8	–5.9	–6.3	–5.3	–5.0	–5.9	–6.10
**3**	–7.6	–7.5	–7.1	–6.6	–6.8	–7.2	–7.3	–6.4	–6.2	–7.4	–6.10
**4**	**–8.1**	–7.5	–7.2	–6.6	–7.1	–7.1	–7.8	–6.3	–6.3	–7.8	**–6.50**
**5**	**–9.1**	–7.5	**–8.6**	**–8.0**	–7.7	**–8.9**	**–8.0**	–7.1	–7.0	**–8.3**	–7.00
**6**	**–9.1**	**–8.0**	**–8.6**	**–8.2**	–7.9	**–8.7**	**–8.2**	–7.1	–6.9	**–8.7**	–7.00
**7**	**–8.5**	**–8.0**	**–7.8**	–6.7	–7.6	–7.2	**–8.5**	–6.6	–6.4	–7.1	–6.20
**8**	**–9.1**	–7.9	**–8.5**	–7.6	–7.8	**–8.0**	**–8.5**	–7.2	–7.1	**–8.5**	**–6.70**
**9**	**–10.3**	**–8.8**	**–8.7**	**–9.1**	**–10.2**	**–10.1**	**–8.5**	**–9.3**	**–8.2**	**–9.9**	–6.70
**10**	**–8.1**	–6.9	**–7.7**	–6.9	–7.8	–7.7	**–8.0**	**–7.9**	–6.6	–7.7	–5.70
**11**	–7.7	–7.5	–7.2	–6.6	–6.8	–7.2	–7.4	–6.4	–6.1	–7.4	**–6.10**
**12**	**–9.2**	–7.5	**–8.9**	–7.8	–7.8	**–8.7**	**–8.1**	–7.4	–7.6	**–8.3**	**–7.20**
**13**	**–9.4**	–7.9	**–10.2**	**–8.3**	**–9.4**	**–10.0**	–5.6	**–8.7**	**–8.4**	**–9.1**	–6.40
**14**	**–9.5**	–6.3	**–8.9**	**–8.3**	**–8.8**	**–8.5**	–7.9	**–7.9**	–7.2	**–8.4**	**–6.00**
**15**	**–8.5**	–6.9	**–10.1**	**–8.7**	**–9.5**	**–9.5**	**–9.2**	**–8.5**	–7.1	–**8.7**	–6.0
**16**	**–9.0**	–7.0	–**8.8**	**–8.5**	**–9.5**	**–9.1**	–7.6	–7.5	–7.4	**–8.3**	–6.50
**17**	**–9.1**	–6.7	**–9.2**	**–8.5**	**–9.6**	**–9.2**	**–8.1**	**–8.0**	–7.4	**–8.1**	**–6.40**
**18**	**–9.8**	–7.0	**–8.9**	**–8.4**	**–9.6**	**–9.0**	–7.9	**–8.1**	–7.5	**–8.0**	–6.70
**19**	**–9.0**	–7.5	**–8.5**	**–9.1**	**–9.1**	**–10.5**	–6.4	**–8.2**	–7.2	**–8.9**	–6.40
**20**	**–8.1**	**–8.0**	–7.6	–6.5	–7.5	–7.6	**–8.0**	–6.7	–6.4	–7.5	–6.50
**21**	**–9.2**	**–9.3**	**–8.6**	–7.7	**–8.9**	**–9.3**	–7.2	**–8.5**	–7.6	**–8.5**	**–6.70**
**22**	–7.6	**–10.2**	**–8.9**	**–8.3**	–7.8	**–8.6**	–4.1	–7.5	–6.9	**–8.0**	–5.80
**23**	**–8.3**	**–11.3**	**–10.3**	**–9.2**	**–8.2**	**–9.3**	–4.1	**–8.3**	–7.7	**–9.5**	–6.50
**24**	**–8.6**	**–9.7**	**–8.8**	**–8.0**	**–9.0**	**–8.9**	–5.9	**–7.9**	–7.4	**–8.6**	**–6.20**
**25**	**–8.5**	**–9.8**	**–8.7**	**–8.0**	**–8.8**	**–9.0**	–4.8	–7.7	–7.8	**–8.9**	**–6.40**

We next employed the molecular docking simulation protocols of the AutoDock Vina suite to estimate the binding affinities in parallel with those of well-documented high-affinity ligands. We identified prospective targets for several of these compounds through a specific reverse screening protocol. Docking simulations were carried out for all 25 compounds against the 11 protein targets identified by SwissTargetPrediction (MAPK14, VEGFR1, FLT3, JAK1, COX-1/2, α_2b_AR, carbonic anhydrase II, Hsp90α, cytochrome P450s, and tyrosinase). Results were scrutinised with special attention to the most-active hits from the cell proliferation and tyrosinase inhibition assays. The docking protocol was validated by re-docking the co-crystallised (native) standard ligand within the catalytic domain of the specifically selected target protein. We observed that the re-docked pose of the co-crystallised ligand retained the experimental binding conformation with an RMSD value of less than 1 Å. Key interactions essential for inhibiting the identified protein targets were considered screening parameters along with the obtained docking score for each hit against respective target proteins. Compounds were predicted to have appreciable binding affinities to only selected macromolecular targets (Table 3). Briefly, for the substituted pyrazoles **P1**–**P3** (**P2** being an aliphatic-ring-fused bicyclic) and **P11** (an isoxazole), no significant interactions with any of the identified targets were predicted. For pyrazole **P4**, only a single but considerably selective interaction was predicted, namely with the MAPK14/p38α kinase, a rather remarkable finding given the small size of this molecule. Compounds **P5**–**P8**, **P10**, **P14**, **P16**, and **P18**, which are substituted or comparable ring-fused pyrazoles, and substituted pyrazolones **P20**, **P21**, **P24**, and **P25**, displayed appreciable interactions with multiple targets were predicted, including the four kinases (MAPK14, VEGFR1, FLT3, and JAK1), two oxidoreductases (COX-1 and COX-2), one adrenergic receptor (α_2b_AR), and cytochrome P450). A number of the other compounds (**P9**, **P13**, and **P15**–**P19**, which are substituted pyrazole analogues of celecoxib, as well as the substituted pyrazolones **P22** and **P23)** were predicted to be relatively more promiscuous (less-selective). Among the predictions are good-to-strong binding interactions with VEGFR1 (**P23**), FLT3 (**P13**, **P15**, **P17**, and **P23**), JAK1 (**P9**, **P19**, and **P23**), and α_2b_AR (**P15**), as well as a stronger interaction (but with less selectivity) to the cytosolic Hsp90α for two pyrazoles (**P9** and **P13**); only these two pyrazoles were predicted to interact significantly with Hsp90α. Importantly, while no meaningful interactions were predicted for any of the identified targets for one of the isoxazoles (**P11**), for the other isoxazole (**P12**) significant interactions with the MAPK14, FLT3, one oxidoreductase (COX-2), α_2b_AR and the cytochrome P450s were predicted. Here, it seems worth noting that in the cytotoxicity assay, compound **P14** (A375) exhibited an IC_50_ comparable to that of celecoxib, whereas compound **P19** (A431) exerted no discernable activity against cells of SCC-12 or SK-MEL-28 lines, in contrast to celecoxib.

Overexpression of heat shock protein 90 (Hsp90) is common in various types of cancer, including cutaneous cancers^127–130^, as well as in chronic skin defects such as inflammation and burns^131–134^. Carbonic anhydrase II elevation has been reported in mycosis fungoides^135^, in adenomas, in glandular tumours (sweat, sebaceous, and major salivary glands), and in various tumours of the skin^136–139^. Cytochrome P450 enzyme systems are involved in drug biotransformation, and as such, are especially noteworthy for their involvement in numerous deleterious drug–drug interactions^140^^,^^141^. Particular P450 isoforms (isozymes; CYPs) are also known to be significant players in various inflammatory conditions, notably through altering aryl hydrocarbon receptor^142^ and retinoid-X-receptor (RxR) gene products expression^143^ and in other aspects of function in the human epidermis constitutively or under drug treatments^140–144^. Modulation of VEGFR1 expression has been shown to contribute to the pathogenesis of skin inflammation and cancer, including melanoma, placing VEGFR1 prominently among important dermal therapeutic targets^145–149^. Alterations of MAPK14 expression and associated pathways have been tied (directly and by targeted modulation) to SCs including cutaneous malignant melanoma, as well as to skin inflammation^150–157^. Dysregulation of Janus kinase 1, a protein tyrosine kinase (JAK1), is known to be a potential contributor to cutaneous inflammation and carcinogenesis, and its targeting can lead to disease resolution^158–162^. Alpha-2B adrenergic receptor modulation has been shown to mitigate skin cell migration, glaucoma, and other human ailments^163–165^. Deregulation of FLT3 has been implicated in disorders involving cutaneous inflammation, such as psoriasis, unwanted hair pigmentation changes, and cancers including melanoma and leukemias^166–170^.

Finally, using the *in silico* SwissADME and ProTox II drug-likeness (DL) properties and organ toxicity prediction analyses, we observed that all these hit compounds’ violations of DL rules, physicochemical properties, ADME parameters, and DL rules fall within the defined and acceptable parameters for a good drug candidate (cf. Tables S2, S3, and S5). The predictions concerning P-glycoprotein (P-gp) and P450 family member inhibition varied across individual compound structures, and any compounds moving directly forward into further preclinical assessment would require assessment in suitable physical assessments.

## Discussion and conclusions

The central goal of our work here is to introduce new small molecule scaffolds into the development pipelines for SC or pigmentation disorders. Towards furthering this aim, we devised and reports herein two modified approaches for the synthesis of various pyrazoles, isoxazoles, and pyrazolones. We engaged microwave-assisted methods for synthesising unsubstituted pyrazoles (**P1**–**P6**) and pyrazolones (**P20**–**P25**), and a catalytic regioselective synthesis for making substituted pyrazoles (**P7**–**P10**, **P14**–**P19**) and isoxazoles (**P11**, **P12**) in good to high yields. These compounds were characterised and identified as a series of lead compounds with varying degrees of anti-tyrosinase, antioxidant, and anticancer activities. Melanocytes with dysregulated tyrosinase-associated function leading to excessive production of melanin have been implicated in the development of various pigmentary dermatoses, including age-related spots, chloasma, freckles, melasma, post-inflammatory hyperpigmentation, and malignant melanoma^24^^,^^32–36^^,^^39^. Moreover, disease-causing mutations in the tyrosinase gene cause melanin deficiency in OCA1, an autosomal recessive disorder characterised by the lack of eye, hair, and skin pigmentation^30^. Therefore, genetic or pharmacological modulation of melanogenesis is a potential therapeutic approach for melanoma and various skin pigmentation disorders^40–44^. Furthermore, tyrosinase inhibition aimed at reducing melanogenesis is relevant in the agroindustry for the preservation of fruit via mitigating browning, interfering with insect moulting (pesticidal), and preventing deleterious adhesion of marine organisms^171^. Clearly, suitably designed tyrosinase inhibitors would be of prospectively high value for an array of cosmetics, skin health, and agro-industry uses^49^.

Cancers of the skin are in aggregate among the most prevalent of cancer types with respect to tissue-of-origin, with about 9500 diagnoses per day in the U.S. alone^172–174^. Available treatments are hampered by drug resistance, side-effects, low bioavailability, or high cost. Several of the compounds characterised in our studies exhibited noteworthy anti-skin-cancer activities, some superior to cisplatin’s (ranging up to eightfold greater), with better SI for cancerous over non-cancerous cells. The most-active broad-spectrum anticancer compound, **P25**, suppressed cell migration and colony formation in the A431 and SK-MEL-28 cell lines *in vitro*, likely through modulation of certain parallel pathways significantly impinging on the induction of apoptosis, as evidenced by altered apoptosis-related marker proteins, namely marked increases in cleaved caspases 3 and 9, and cleaved PARP, as well as a pronounced increase in the Bax/Bcl-2 ratio.

**Figure 10.**
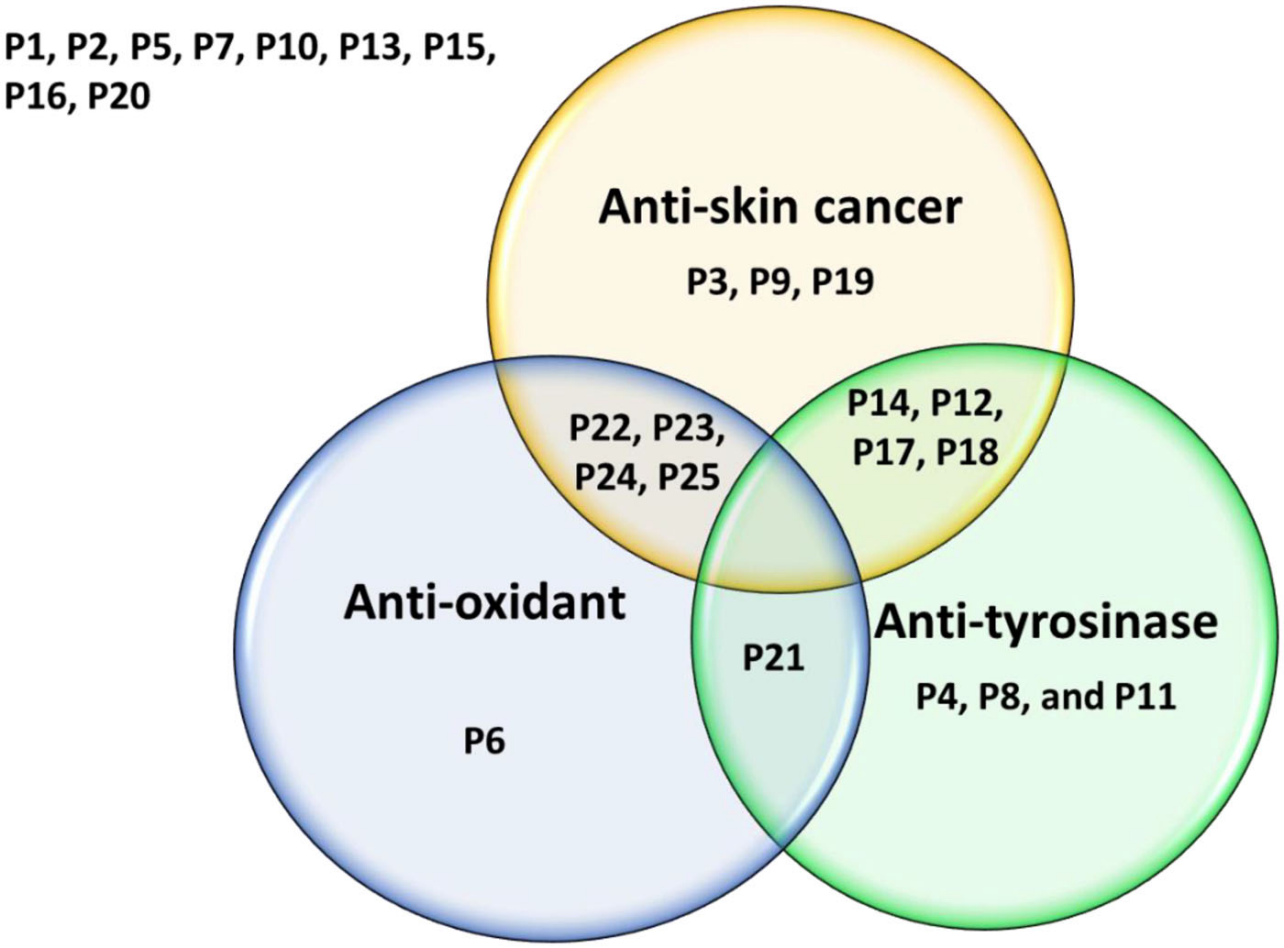
The Venn diagram summarises the anti-proliferative (anti-skin cancer), antioxidant, and tyrosinase inhibitory effects of the 25 compounds. Dual-acting compounds were identified with noteworthy anti-proliferative and antioxidant activity (**P22**, **P23**, **P24**, and **P25**); anti-proliferative and anti-tyrosinase activity (**P12**, **P14**, **P17**, and **P18**); and antioxidant and anti-tyrosinase activity (**P21**). Some tested compounds exhibited only a single noteworthy activity: antioxidant (**P6**); anti-tyrosinase (**P4**, **P8**, and **P19**), and/or anti-skin-cancer (**P3**, **P9**, and **P19**).

Several of the evaluated compounds also showed dual anti-tyrosinase and antioxidant, anticancer and antioxidant, or potent anti-tyrosinase with mild anticancer properties (Venn diagram, Figure 10). The anti-tyrosinase, anti-proliferative, antioxidant, anti-migratory, and pro-apoptotic effects observed among these structurally analogous compounds suggest that further elaboration and optimisation efforts could ultimately provide high-value clinical candidates, possibly with advantageous, synergistic dual-targeting attributes. To further understand the underlying molecular mechanism-of-action of these compounds, *in silico* simulations and analyses identified that a number of the compounds are predicted to bind to a relatively small number of targets highly relevant to cancer, inflammation, and pigmentation disorders. It is noteworthy that many of these identified targets including enzymes, kinases, proteases, and receptors have been shown to play important roles in carcinogenesis and SC progression and inflammation. Moreover, targets such as lipoxygenases are well known to catalyse the formation of corresponding hydroperoxides from polyunsaturated fatty acids such as linoleic acid and arachidonic acid. They are expressed in immune, epithelial, and tumour cells that display a variety of physiological functions, including inflammation, skin disorder, and tumorigenesis^175–177^. In fact, SK-MEL-28, one of a series of melanoma cell lines established from patient-derived tumour samples used in this study, all believed to play an essential role in skin carcinogenesis or treatment. Therefore, further studies are warranted to assess the validity of these predictions in suitably construed physical experiments. Regardless, the data amassed in this study will be helpful in further attempts to optimise the activity of the hit molecules and the determination of their putative drug targets and potential mechanisms of action. The fact that pharmacological modulation of several of these dysregulated targets and receptors appeared to induce apoptotic death of tumorigenic epidermal cells without affecting the viability of non-transformed epidermal cells^178^ suggests that suitable multicomponent manipulation of various signalling pathways via multiple targets and mechanisms can be a promising strategy for the small-molecule-based management of some types of cancers, chronic inflammatory maladies, and pigmentation disorders.

Altogether, our results offer up promising prototypes for further studies towards identifying single, in-combination, or adjuvant agents for the control of melanoma and NMSCs, and tyrosinase-related pigmentary disorders.

## Materials and experimental methods

### Chemistry

#### Synthesis and characterisation of pyrazole derivatives

*General information*: All the chemicals were purchased from Sigma-Aldrich (St. Louis, MO) and TCI (Portland, OR). All the reagents were commercial grade, and solvents were purified according to established procedures. Organic extracts were dried over anhydrous sodium sulphate. After completion of the reactions, solvents were removed in a rotary evaporator under reduced pressure using a Buchi Rotavapor equipped with a vacuum controller and vacuum pump. Silica gel (60–120 mesh size) was used for column chromatography. Reactions were monitored by thin-layer chromatography (TLC) on silica gel precoated aluminium cards with fluorescent indicator visualisable at 254 nm (0.25 mm). Developed plates were visualised by a Spectroline ENF 260C/FE UV apparatus. Nuclear magnetic resonance (NMR) spectra were recorded on a JEOL 400 MHz FT spectrometer in the indicated solvent. Chemical shifts are expressed in *δ* units (ppm) from tetramethylsilane (TMS) as the internal standard for ^1^H NMR (400 MHz) and for ^13^C NMR (100 MHz). Infrared spectra (IR) were run on a SpectrumOne FT-ATR spectrophotometer (Perkin Elmer, Waltham, MA). Band position and absorption ranges are given in cm^−1^. Gas chromatography–mass spectrometry (GC–MS) analysis was carried out on an Agilent GC-MS (7890A-5975C VL MSD) system.

Microwave-assisted reactions were performed with a Discover S-Class (CEM) single-mode microwave reactor, where the instrument settings were controlled with PC-running Synergy 1.4 software. All experiments were carried out in microwave reaction vials using a stirring bar (length 20 mm, diameter 6 mm). Stirring, temperature, irradiation power, PowerMAX (*in situ* cooling during the microwave irradiation), ramp, and hold times were set as indicated. The temperature of the reaction was monitored by a built-in infrared sensor. After completion of the reaction, the mixture was cooled to 25 °C via air-jet cooling. Most products were purified using column chromatography, and some were isolated in pure form through recrystallisation. All the synthesised compounds were characterised by NMR, GC–MS, and HRMS analysis.

### General synthesis procedures

#### Microwave assisted synthesis of pyrazoles P1, P2, P3, and P5

A 10 mL microwave reaction vial, equipped with a stir bar, charged with hydrazine hydrate (1 mmol), 1,3-diketone (1 mmol), 1:1 water and glycerol (2 mL) and added potassium carbonate was mixed by stirring the solution at room temperature. The vial was then sealed with a Teflon-lined silicone cap and placed in the microwave synthesiser cavity. Microwave irradiation of 200 W was used, and the temperature ramped from 25 °C to 80 °C. Once the temperature reached 80 °C, the reaction continued for 10–20 min at the same temperature while stirring. Upon completion, the reaction mixture was cooled to room temperature, extracted with ethyl acetate (EtOAc), and evaporated to collect the solid product mixture, which was either recrystallised or purified by column chromatography to obtain a pure product.

#### Synthesis of 4-chloro-5-methyl-3-phenyl-1H-pyrazole (P4)^101^

To a solution of 5-methyl-3-phenyl-1H-pyrazole (**P3**) (1 mmol) in acetonitrile (5 mL), *N*-chlorosuccinimide (2 mmol) was added, and the solution stirred continuously at 40 °C for 3 h. After completion, the acetonitrile solvent was removed, and the mixture was washed with saturated sodium thiosulphate (2 × 20 mL) and water (20 mL), and the product was extracted with EtOAc solvent.

#### Synthesis of pyrazole P6^102^

We used a slightly modified method developed by Hilt et al. To the solution of **P5** (1 mmol) in 5 mL of acetonitrile, DDQ (1.2 mmol) was added, and the mixture was stirred continuously at room temperature for 5 h. After filtration through a short pad of silica, the crude product was purified by column chromatography to yield pure pyrazole **P6**.

#### Cobalt(II)-catalysed synthesis of pyrazoles, isoxazoles (P7–P12 and P14–P19)

To a solution of cobalt(II) chloride hexahydrate and arylhydrazine/hydroxylamine hydrochloride (1 mmol) in acetonitrile, a 1,3-diketone (1 mmol) was added, and the mixture was stirred at room temperature. The reaction was monitored using TLC and GC–MS analysis. After completion, the reaction mixture was filtered through celite and washed with EtOAc. Products were purified by recrystallisation or column chromatography using EtOAc and hexane mixture as eluent.

#### Microwave-assisted synthesis of 1,3-diarylpyrazolones (P20–P25)^89^

A 10 mL microwave reaction vial equipped with a stir bar was charged with the substituted β-ketoester (2 mmol), the appropriate arylhydrazine (2 mmol), glycerol (1 mL), and water (1 mL). The vial was sealed with a Teflon-lined silicone cap and placed in the microwave reactor. Microwave irradiation of 300 W was used, and the temperature ramped from room temperature to 100 °C. The reaction continued for 20 min at the same temperature while stirring. After completion, the reaction mixture was cooled to room temperature, extracted with EtOAc, dried with sodium sulphate, and the solvent was removed under vacuum to extract the crude product. When the crude product was a solid, it was recrystallised using acetonitrile solvent to obtain the pure product in crystalline form. In some cases, the products were purified by column chromatography using hexane–EtOAc as eluent.

#### Synthesis of 1,3,5-triphenyl pyrazole (P13)

We initially synthesised a chalcone from benzaldehyde and acetophenone under microwave reaction conditions that we had developed previously^179^. The isolated chalcone was further converted to 1,3,5-triphenyl pyrazole via cyclocondensation with phenyl hydrazine, followed by an oxidation reaction using Pd/C as the oxidising agent. We have slightly modified the procedure reported by Hayashi and coworkers for the oxidation step^103^. To the solution of chalcone (1 mmol) in 5 mL of acetic acid, phenyl hydrazine (1.2 mmol) was added and refluxed for 3 h. The pyrazoline product was extracted using EtOAc used directly for the next step. To the solution of pyrazoline in dichloromethane, 272 mg (1.2 mmol) of 2,3-dichloro-5,6-dicyano-1,4-benzoquinone (DDQ) was added and stirred overnight at room temperature. The final product was then extracted with EtOAc (40 mL × 3). After usual work-up, the obtained residue was column chromatographed on silica-gel to afford 1,3,5-triphenylpyrazole (**P13**).

#### Structures assignment based on ^1^H and ^13^C NMR, IR, GC–MS, and HRMS analysis

All the structures of synthesised azoles were confirmed by ^1^H NMR, ^13^C NMR, IR, GC–MS, and HRMS (ESI-MS) analysis.

*4-Ethyl-3,5-dimethyl-1H-pyrazole (****P1****)*^180^: ^1^H NMR (400 MHz, CDCl_3_): 9.96 (brs, 1H), 2.34 (q, 2H, *J* = 7.3 Hz), 2.20 (s, 6H), and 1.04 (t, 3H, *J* = 7.3 Hz). ^13^C NMR (100 MHz; CDCl_3_): 141.5, 117.2, 16.3, 15.2, 10.8, and 10.7. IR (KBr) cm^−1^: 3202, 3154, 3092, 2962, 2927, 1665, 1592, 1451, 1419, 1301, 1159, 1007, and 796. High-resolution mass spectrometry (HRMS) (ESI) calcd. for C_7_H_12_N_2_ (M + H^+^), 125.1073; found, 125.1073.

*3-Methyl-1,4,5,6-tetrahydrocyclopenta[c]pyrazole (****P2****)*^181^: ^1^H NMR (400 MHz, CDCl_3_): 10.14 (brs, 1H), 2.67 (t, 2H, *J* = 6.9 Hz), 2.52 (t, 2H, *J* = 6.9 Hz), 2.38–2.43 (m, 2H), and 2.20 (s, 3H). ^13^C NMR (100 MHz; CDCl_3_): 159.9, 133.9, 122.9, 30.6, 24.6, 22.3, and 10.7. HRMS (ESI) calcd. for C_7_H_10_N_2_ (M + H^+^), 123.0917; found, 123.0919.

*5-Methyl-3-phenyl-1H-pyrazole (****P3****)*^182^: ^1^H NMR (400 MHz, CDCl_3_): 10.90 (brs, 1H), 7.74 (d, 2H, *J* = 7.2 Hz), 7.40 (t, 2H, *J* = 7.2 Hz), 7.33 (t, 2H, *J* = 7.2 Hz), 6.37 (s, 1H), and 2.33 (s, 3H). ^13^C NMR (100 MHz; CDCl_3_): 149.9, 143.2, 132.6, 128.7, 127.8, 125.8, 102.1, and 11.6. HRMS (ESI) calcd. for C_10_H_10_N_2_ (M + H^+^), 159.0917; found, 159.0915.

*4-Chloro-5-methyl-3-phenyl-1H-pyrazole (****P4****):* m.p.: 119–120 °C. ^1^H NMR (400 MHz, CDCl_3_): 10.93 (brs, 1H), 7.73 (d, 2H, *J* = 8.2 Hz), 7.33–7.38 (m, 3H), and 2.10 (s, 3H). ^13^C NMR (100 MHz; CDCl_3_): 144.1, 141.7, 130.4, 128.7, 128.6, 127.3, 106.8, and 10.0. HRMS (ESI) calcd. for C_10_H_9_ClN_2_ (M + H^+^), 193.0527; found, 193.0524.

*3-Methyl-4,5-dihydro-2H-benzo[g]indazole (****P5****)*: m.p.: 137–138 °C. ^1^H NMR (400 MHz, CDCl_3_): 10.5 (brs, 1H), 7.72–7.73 (m, 1H), 7.16–7.23 (m, 3H), 2.92 (t, 2H, *J* = 7.3 Hz), 2.63 (t, 2H, *J* = 7.3 Hz), and 2.22 (s, 3H). ^13^C NMR (100 MHz; CDCl_3_): 146.2, 137.7, 136.7, 129.1, 128.4, 127.6, 126.8, 122.3, 113.8, 29.8, 18.6, and 10.1. HRMS (ESI) calcd. for C_12_H_12_N_2_ (M + H^+^), 185.1073; found, 185.1074.

*3-Methyl-2H-benzo[g]indazole (****P6****)*^183^: ^1^H NMR (400 MHz, CDCl_3_): 8.17–8.20 (m, 1H), 7.87–7.90 (m, 1H), 7.50–7.53 (m, 2H), 7.39 (d, 1H, *J* = 8.7 Hz), 7.38 (d, 1H, *J* = 8.7 Hz), and 2.50 (s, 3H). ^13^C NMR (100 MHz; CDCl_3_): 143.2, 138.8, 132.6, 128.7, 126.5, 126.2, 121.8, 121.6, 120.5, 118.42, 118.39, and 11.9. HRMS (ESI) calcd. for C_12_H_10_N_2_ (M + H^+^), 183.0917; found, 183.0923.

*4-Ethyl-3,5-dimethyl-1-phenyl-1H-pyrazole (****P7****)*: m.p.: 69–70 °C. ^1^H NMR (400 MHz, CDCl_3_): 7.38–7.44 (m, 4H), 7.30–7.34 (m, 1H), 2.40 (q, 2H, *J* = 7.8 Hz), 2.28 (s, 3H), 2.22 (s, 3H), and 1.11 (t, 3H, *J* = 7.8 Hz). ^13^C NMR (100 MHz; CDCl_3_): 162.8, 147.2, 139.4, 136.2, 129.2, 127.5, 124.8, 119.9, 16.7, 15.1, 11.6, and 10.7. HRMS (ESI) calcd. for C_13_H_16_N_2_ (M + H^+^), 201.1386; found, 201.1391.

*3-Methyl-1-phenyl-1,4,5,6-tetrahydrocyclopenta[c]pyrazole (****P8****)*^184^: m.p.: 51–52 °C. ^1^H NMR (400 MHz, CDCl_3_): 7.56 (d, 2H, *J* = 7.3 Hz), 7.35 (t, 2H, *J* = 7.3 Hz), 7.15 (t, 1H, *J* = 7.3 Hz), 2.92 (m, 2H), 2.55 (t, 4H, *J* = 4.1 Hz), and 2.25 (s, 3H). ^13^C NMR (100 MHz; CDCl_3_): 149.0, 143.9, 140.4, 129.3, 128.4, 125.1, 118.6, 31.0, 26.9, 22.4, and 12.8. HRMS (ESI) calcd. for C_13_H_14_N_2_ (M + H^+^), 199.1230; found, 199.1228.

*3-Methyl-2-phenyl-4,5-dihydro-2H-benzo[g]indazole (****P9****)*^185^: ^1^H NMR (400 MHz, CDCl_3_): 7.34–7.39 (m, 2H), 7.33 (t, 2H, *J* = 7.2 Hz), 7.27 (d, 1H, *J* = 7.6 Hz), 7.16 (d, 1H, *J* = 7.2 Hz), 7.01 (t, 1H, *J* = 7.6 Hz), 6.87 (t, 1H, *J* = 7.6 Hz), 6.73 (d, 1H, *J* = 7.6 Hz), 2.87 (t, 2H, *J* = 7.6 Hz), 2.55 (t, 2H, *J* = 7.6 Hz), and 2.21 (s, 3H). ^13^C NMR (100 MHz; CDCl_3_): 146.0, 140.9, 138.1, 137.2, 129.2, 128.6, 127.8, 127.3, 127.2, 126.3, 125.6, 123.1, 118.6, 30.7, 19.3, and 11.8. HRMS (ESI) calcd. for C_18_H_16_N_2_ (M + H^+^), 261.1386; found, 261.1383.

*Ethyl 3,5-dimethyl-1-phenyl-1H-pyrazole-4-carboxylate (****P10****)*^186^: ^1^H NMR (400 MHz, CDCl_3_): 7.36–7.39 (m, 2H), 7.28–7.32 (m, 3H), 4.22 (q, 2H, *J* = 7.2 Hz), 2.42 (s, 3H), 2.41 (s, 3H), and 1.28 (t, 3H, *J* = 7.2 Hz). ^13^C NMR (100 MHz; CDCl_3_): 164.5, 151.4, 144.4, 138.8, 129.2, 128.4, 125.6, 110.8, 59.7, 14.4, 14.3, and 12.6. HRMS (ESI) calcd. for C_14_H_16_N_2_O_2_ (M + H^+^), 245.1285; found, 245.1285.

*5-Methyl-3-phenylisoxazole (****P11****)*^187^: ^1^H NMR (400 MHz, CDCl_3_): 7.69–7.72 (m, 2H), 7.36–7.40 (m, 3H), 6.31 (s, 1H), and 2.30 (s, 3H). ^13^C NMR (100 MHz; CDCl_3_): 169.7, 160.4, 130.1, 129.0, 127.6, 125.8, 100.3, and 11.6. HRMS (ESI) calcd. for C_10_H_9_NO (M + H^+^), 160.0757; found, 160.0756.

*3-Methyl-4,5-dihydronaphtho[1,2-c]isoxazole (****P12****)*: m.p.: 56–57 °C. ^1^H NMR (400 MHz, CDCl_3_): 7.53–7.55 (m, 1H), 7.17–7.20 (m, 3H), 2.92 (t, 2H, *J* = 7.8 Hz), 2.56 (t, 2H, *J* = 7.8 Hz), and 2.20 (s, 3H). ^13^C NMR (100 MHz; CDCl_3_): 164.9, 157.9, 136.4, 129.4, 128.4, 127.0, 125.4, 121.7, 112.4, 28.9, 17.8, and 10.0. HRMS (ESI) calcd. for C_12_H_11_NO (M + H^+^), 186.0913; found, 186.0920.

*1,3,5-Triphenyl-1H-pyrazole (****P13****)*^188^: m.p.: 136–137 °C. ^1^H NMR (400 MHz, CDCl_3_): 7.85 (d, 2H, *J* = 7.6 Hz), 7.35 (t, 2H, *J* = 7.6 Hz), 7.17–7.31 (m, 11H), and 6.75 (s, 1H). ^13^C NMR (100 MHz; CDCl_3_): 152.0, 144.5, 140.1, 133.0, 130.6, 128.9, 128.8, 128.7, 128.5, 128.4, 128.1, 127.5, 125.9, 125.4, and 105.3. GC–MS calcd. for C_21_H_16_N_2_, 296.3; found, 296.1.

*3-Methyl-1,5-diphenyl-1H-pyrazole (****P14****)*^188^: ^1^H NMR (400 MHz, CDCl_3_): 7.24–7.31 (m, 8H), 7.19–7.21 (m, 2H), 6.31 (s, 1H), and 2.39 (s, 3H). ^13^C NMR (100 MHz; CDCl_3_): 149.4, 144.0, 139.7, 130.4, 129.0, 128.7, 128.5, 128.4, 127.5, 125.3, 107.8, and 13.5. HRMS (ESI) calcd. for C_16_H_14_N_2_ (M + H^+^), 235.1230; found, 235.1229.

*1,5-Diphenyl-3-(trifluoromethyl)-1H-pyrazole (****P15****)*^189^: m.p.: 90–91 °C. ^1^H NMR (400 MHz, CDCl_3_): 7.35–7.38 (m, 8H), 7.24–7.28 (m, 2H), and 6.78 (s, 1H). ^13^C NMR (100 MHz; CDCl_3_): 144.7, 143.4 (q, C-F coupling, *J* = 31 Hz), 139.2, 129.2, 129.1, 129.0, 128.8, 128.7, 128.5, 125.5, and 105.6. HRMS (ESI) calcd. for C_16_H_11_F_3_N_2_ (M + H^+^), 289.0947; found, 289.0940.

*1-(4-Bromophenyl)-3-methyl-5-phenyl-1H-pyrazole (****P16****)*^190^: m.p.: 85–86 °C. ^1^H NMR (400 MHz, CDCl_3_): 7.44–7.42 (d, 2H, *J* = 6.8 Hz), 7.29–7.33 (m, 3H), 7.15–7.18 (m, 4H), 6.34 (s, 1H), and 2.44 (s, 3H). ^13^C NMR (100 MHz; CDCl_3_): 149.1, 145.5, 136.1, 132.7, 129.9, 129.2, 128.9, 128.4, 127.3, 123.1, 108.4, and 12.8. HRMS (ESI) calcd. for C_16_H_13_BrN_2_ (M + H^+^), 313.0335; found, 313.0327.

*1-(4-Chlorophenyl)-3-methyl-5-phenyl-1H-pyrazole (****P17****)*^190^: m.p.: 102–103 °C. ^1^H NMR (400 MHz, CDCl_3_): 7.27–7.29 (m, 3H), 7.24–7.26 (m, 2H), 7.17–7.19 (m, 4H), 6.28 (s, 1H), and 2.36 (s, 3H). ^13^C NMR (100 MHz; CDCl_3_): 149.9, 144.2, 138.4, 133.1, 130.4, 129.3, 128.9, 128.8, 128.7, 126.4, 108.4, and 13.6. HRMS (ESI) calcd. for C_16_H_13_ClN_2_ (M + H^+^), 269.0840; found, 269.0838.

*1-(4-Methoxyphenyl)-3-methyl-5-phenyl-1H-pyrazole (****P18****)*^190^: m.p.: 107–108 °C. ^1^H NMR (400 MHz, CDCl_3_): 7.24–7.26 (m, 3H), 7.16–7.18 (m, 4H), 6.80 (d, 2H, *J* = 7.4 Hz), 6.29 (s, 1H), 3.76 (s, 3H), and 2.38 (s, 3H). ^13^C NMR (100 MHz; CDCl_3_): 159.1, 148.9, 144.3, 132.7, 130.3, 128.8, 128.7, 128.5, 126.9, 114.4, 107.4, 55.7, and 13.5. HRMS (ESI) calcd. for C_17_H_16_N_2_O (M + H^+^), 265.1335; found, 265.1330.

*4-(3-Methyl-5-phenyl-1H-pyrazol-1-yl)benzenesulfonamide (****P19****)*^191^: m.p.: 201–203 °C. ^1^H NMR (400 MHz, CDCl_3_): 7.84 (d, 2H, *J* = 7.6 Hz), 7.41 (d, 2H, *J* = 7.6 Hz), 7.36–7.38 (m, 3H), 7.23–7.25 (m, 2H), 6.36 (s, 1H), 4.98 (brs, 2H), and 2.41 (s, 3H). ^13^C NMR (100 MHz; CDCl_3_): 150.8, 144.1, 143.4, 139.7, 130.2, 128.8, 128.7, 127.3, 124.6, 109.4, and 13.6. HRMS (ESI) calcd. for C_16_H_15_N_3_O_2_S (M + H^+^), 314.0958; found, 314.0952.

Compounds **P20**–**P25** were prepared according to our previous report^89^.

### Biological evaluation assays

#### Antibodies, chemicals, and reagents

Bovine serum albumin (BSA), dimethyl sulphoxide (DMSO), 4′,6-diamidino-2-phenylindole (DAPI-#DUO82040), MTT dye (3-(4,5-dimethylthiazol-2-yl)-2,5-diphenyltetrazolium bromide, 98% TLC) *in situ* mounting media, 4-hydroxyphenyl β-d-glucopyranoside (arbutin), mushroom tyrosinase (EC 1.14.18.1), and l-tyrosine were purchased from Sigma-Aldrich Chemical Company (St. Louis, MO) and MP Biomedicals (Irvine, CA). The antibodies for immunoblotting and immunocytochemistry, including Bcl-2 (2876S), Bax (2772S), caspase-3 (9662S), caspases 9 (#9502), PARP antibody #9542, mTOR, β-actin (13E5), and horseradish peroxidase (HRP)-conjugated anti-mouse and anti-rabbit secondary antibodies, were all obtained from Cell Signaling Technologies (Beverly, MA). Antibody to peIF4E (Ser209) (76256) was from Abcam (Cambridge, UK). A 2% (w/v) aqueous solution of gentian violet and crystal violet were from Ricca Chemical Company (Arlington, TX). Radioimmunoprecipitation assay (RIPA) buffer and Pierce Bicinchoninic Acid (BCA™) protein assay kit were purchased from Thermo Fisher Scientific (Rockford, IL). Mini-protean precast Tris-Glycine Gels (TGX) were from Bio-Rad (Bio-Rad Laboratories Inc., Hercules, CA). The SuperSignal™ West Pico PLUS Chemiluminescent substrate detection system was from Thermo Fisher Scientific (Waltham, MA). Dulbecco’s modified Eagle’s medium (DMEM) and Roswell Park Memorial Institute Medium (RPMI 1640) were from Corning (Corning, Manassas, VA). Cascade Biologics^®^ PMA-free Human Melanocyte Growth Supplement (HMGS-2, # S0165), and Medium 254 (# M-254-500) were purchased from Gibco™ Invitrogen Cell culture (Thermo Fisher Scientific, Rockford, IL). Epi-Life^®^ Growth Medium with 60 µM calcium and Cascade Biologist human keratinocyte growth supplement (HKGS) 100X (S-001-5) were from Life Technologies (Grand Island, NY). The quick coating solution (cAP-01) was purchased from Angio-Proteomie (Boston, MA). The Dulbecco’s phosphate-buffered saline (DPBS), phosphate-buffered saline (PBS) 1X, defined trypsin inhibitor (DTI) 1X (R007-100), trypsin EDTA 0.25%, 1X(R25200-072), trypsin neutraliser (TN) 1X (R-002-100), penicillin–streptomycin (Pen Strep, 15140-122) (PEST) 100X were purchased from Gibco, Thermo Fisher Scientific (Rockford, IL), and HKGS 100X (S-001-5) were procured from VWR Corporation (Missouri, TX). Quercetin and 5-hydroxy-2-(hydroxymethyl)-4*H*-pyran-4-one (kojic acid) were purchased from Cayman Chemical (Ann Arbor, MI). USDA-approved Origin Fetal Bovine Serum (FBS) was from VWR Seradigm Life Science (Missouri, TX). The organic solvents, including ethanol (6183-10) and methanol (BDH 1135-4LP), were acquired from Macron Chemicals (Radnor, PA) and VWR (Missouri, TX), respectively.

#### Cell lines, cell cultures, cytotoxicity, and viability assessment

Human-derived GFP-expressing melanoma A375 and epidermoid carcinoma A431 cell lines were purchased from Angio-Proteomie (Boston, MA). Human melanoma carcinoma SK-MEL-28 and human immortalised keratinocytes (HaCaT) cell lines were acquired from American Type Culture Collection (ATCC; Manassas, VA). Human cutaneous SCC cell line SCC-12^192^ was generously provided by Dr. James G. Rheinwald to Dr. Tatiana Efimova. Except SK-MEL-28, which was cultured in an RPMI-1640 medium supplemented with 5% FBS, all other human immortalised cell lines were grown and maintained in DMEM containing varying percentages of FBS; 5% FBS for A431, SCC-12, and HaCaT lines, and 10% FBS for A375. All culture media were routinely supplemented with 1% PEST 100X (100 U/mL penicillin, 100 μg/mL streptomycin), and cells were cultured in incubators maintained at 37 °C under an atmosphere of 95% humidity, 20% O_2_, and 5% CO_2_. The growth media of incubated cells were changed every 2–3 days until they attained 70–80% confluence, after which they were sub-cultured for experiments and re-passaging. DMSO was used as the vehicle to prepare a 10 mM stock solution of the test compounds. Control cells were treated with the vehicle (DMSO) at concentrations of 0.01–0.2%, which did not affect cell viability.

Stock solutions (10 mM) were prepared by dissolving each compound to be screened in DMSO. From these stock solutions, escalating concentrations were prepared using the corresponding cell growth media as a diluent, and the cells (65–75% confluent) were treated with (0–40 µM) or without the drug in sext- to octuplicate and incubated for 48 h. All treatment protocols and controls were prepared as previously described^109^.

The potency and cytotoxicity of the test compounds against two cutaneous melanoma (A375 and SK-MEL-28) and two non-melanoma (A431 and SCC-12) SC cell lines in addition to a control immortalised normal human epidermal keratinocytes (HaCaT) cell line were assessed by MTT assay as previously described^109^. The results from increasing concentrations of each test and control (cisplatin and celecoxib) agent were analysed using the zero drug control group as the relative comparator and are expressed as a percentile according to the following equation: ((absorbance in treatment group/absorbance in control) × 100%), and data were computed as earlier described^109^.

#### Colony formation assay

SCC-12 and SK-MEL-28 cells were seeded in T25 flasks in triplicate and allowed to adapt for 24 h before treatment with different concentrations of P25 (0, ½IC_50_, IC_50_, and 1½IC_50_) for 48 h. Cells were then trypsinised, and 1000 viable cells from each treated group were seeded on 10 cm^2^ plates in 10 mL of the medium, and incubated for an additional 14 days before fixation and crystal violet staining, imaging, and colonies assessment as previously described^109^^,^^116^. The plates were scanned, and the area of clones was quantified with the free software Image J (Bethesda, MD) (https://imagej.nih.gov/ij/).

#### *In vitro* wound healing assay

The scratch wound healing assay was performed to evaluate compound **P25**’s ability to limit cell migration and scratch wound closure following our previously described protocol^109^. Briefly, cells were seeded in wells of a 24-well plate and incubated to 100% confluent monolayer, following which a sterile 200 µL Gilson pipette tip was used to scratch a straight line across the centre. Cells were washed gently to remove debris and detached cells and were treated (or not) with increasing concentrations (0, ½IC_50_, IC_50_, and 1½IC_50_) of **P25**. The wounded and re-epithelialisation areas were imaged at 0 h, 24 h, and 48 h using an inverted phase-contrast Olympus IX51 microscope (Olympus America Inc., Center Valley, PA) attached to an Infinity 2 digital camera. Images were processed with an Infinity analysis software version 6.5.6 (Teledyne Lumenera, Ottawa, Canada) and analysed with Adobe Photoshop. The ImageJ software (NIH, Bethesda, MD) was used to measure the migratory area of each treatment group, with results expressed as remaining percentage of unfilled wound surface at 0 h^108^.

#### Immunoblotting

Immunoblotting was performed according to standard procedures of the Bio-Rad electrophoresis system (Bio-Rad, Hercules, CA) as described earlier^109^. Briefly, protein extracts were obtained by lysing cells in RIPA buffer containing protease inhibitors, and were resolved by 4–12% polyacrylamide ready mini-PROTEAN TGX gel electrophoresis with sodium dodecyl sulphate (SDS) running buffer. Gels were electrotransferred to 0.45 μm nitrocellulose membranes (Bio-Rad, Hercules, CA) in Tris-glycine (TG) buffer using a Trans-Blot Turbo Transfer Pack according to manufacturer’s instructions. Membranes were incubated for 1 h in 5–7% non-fat powdered milk dissolved in TBST (Tris-buffered saline containing 0.05% Tween 20) or 5% BSA for phosphorylated proteins, to saturate nonspecific binding sites, followed by an overnight incubation (4 °C) with primary antibodies. Membranes were washed 5× with TBST and treated with suitable HRP-conjugated secondary antibodies (Amersham Life Science Inc., Arlington Height, IL) for another 2 h at room temperature, followed by additional wash steps and chemiluminescence-based detection with the Amersham ECL Prime detection reagent kit and the Bio-Rad Quantity One ChemiDoc™ detection and imaging analysis software systems as described earlier^108^^,^^109^.

#### Antioxidant activity by the DPPH free radical scavenging assay

The antioxidant potential of the synthesised derivatives was determined based on their ability to scavenge the 2,2′-diphenyl-1-picrylhydrazyl (DPPH) radical as previously reported^193^, and the assays were conducted in a 96-well format. An initial screen was done of all 25 derivatives at a concentration of 1 mM. Compounds exhibiting greater than 50% scavenging at 1 mM were then serial-diluted using 100 µL aliquots (ranging from 0.125 mM to 7.8 µM). Methanol was used as the diluent, and 100 µL of DPPH (200 µM; Sigma-Aldrich, St. Louis, MO) was added to each well. Absorbance was determined after 30 min at 517 nm, using a microplate reader (SpectraMax M2, Molecular Devices Corp., Sunnyvale, CA, operated by SoftmaxPro v.4.6 software). Experimentally, it has been determined that absorbance constantly decreases and stabilises after preparing the sample of test compounds. The scavenging capacity (SC) was calculated as SC% = ((Δ*A*_control_ – Δ*A*_sample_)/Δ*A*_control_) × 100%, where Δ*A*_control_ was the absorbance of the reagent blank, and Δ*A*_sample_ was the absorbance of the test samples. The control contained all reagents except the compounds, and all tests were performed in triplicate. Quercetin served as a positive control. The DPPH methanolic solution (1 mL) mixed with DMSO (5 µL) served as a negative control. Antioxidant activity was evaluated by calculating the IC_50_ values denoting the concentration (in µM) of the sample required to scavenge 50% of formed DPPH free radicals, based on absorbance measurements (Table S4).

#### *In vitro* mushroom tyrosinase inhibition assay

The anti-tyrosinase potential of all the synthesised derivatives was determined by an enzymatic tyrosinase inhibition assay based on their ability to inhibit the substrate, l-tyrosine, from binding to the tyrosinase enzyme^194^. Briefly, mushroom tyrosinase, l-tyrosine, and positive control, arbutin and the more potent kojic acid (a fungal secondary metabolite used as a skin whitening agent) were used in the tyrosinase inhibition assays, which were performed in a 96-well microplate format using a SpectraMax M2 microplate reader, operated by SoftmaxPro v.4.6 software (Molecular Devices, Sunnyvale, CA). Test samples were dissolved in DMSO at a concentration of 10 mM and then diluted to different concentrations with phosphate buffer (0.1 M, pH 6.8). Each well contained 40 µL of the sample with 80 µL of phosphate buffer solution (0.1 M, pH 6.8), 40 µL of tyrosinase enzyme (100 units/mL), and 40 µL l-tyrosine (2.5 mM). The mixture was incubated for 30 min at room temperature, and absorbance was measured at 490 nm. Each sample was accompanied by a sample blank containing all components except tyrosinase. Arbutin and kojic acid were used as the positive controls. The results were compared with a negative control consisting of 0.1 M PBS in place of the sample. The percentage of tyrosinase inhibition was calculated as follows: ((Δ*A*_control_ – Δ*A*_sample_)/Δ*A*_control_) × 100. IC_50_ values denoted the sample concentration required to inhibit 50% tyrosinase activity (Table S1).

### *In silico* computational screening studies

#### In silico target(s) fishing, physicochemical and drug-likeness properties predictions, and bioavailability (absorbability) predictions

The SwissTargetPrediction database (http://www.swisstargetprediction.ch/) web server, which predicts the targets of bioactive molecules based on a combination of two-dimensional (2D) and 3D similarity measures with known ligands^123^^,^^195^, was employed to screen and estimate the putative macromolecular targets of all 25 derivatives synthesised. Briefly, each compound’s canonical Simplified Molecular Input-Line Entry System (SMILES) structure formats were individually converted using the ChemDraw tool before importing into the SwissTargetPrediction network database. After the SMILES structures were uploaded to the database, with the species set as “Homo sapiens,” a report of probable matched targets data was obtained. The 3D structures of the target proteins used for molecular docking were selected from the available Protein Data Bank (PDB). During target prediction, compounds possessing the highest probability to regulate identified targets >88.8% probability, and the specific targets were selected based on the predicted score value, and all PDB codes are provided in the master excel sheet as supplementary data.

#### *In silico* molecular docking simulation methodology

Molecular docking simulations were used in this study to predict the binding interactions between ligands and their protein targets and the ligand conformations and orientations (poses) in the functionally relevant sites of target proteins^196^. Using this technique, a molecule is docked into the ligand-binding domain of the target protein associated with the potential activity to identify a specific ligand that can fit within a specific protein ligand-binding domain^197^. The credibility of the connection between the identified targets and the molecules screened was evaluated by docking each compound with the predicted target protein. The molecular docking data were evaluated by selecting the native and clinical ligands of the studied targets and docking them as the positive longitudinal control. First, the SMILES structures of native (commercially available natural or clinically functional) ligands used in the study were retrieved from the PubChem network database (https://pubchem.ncbi.nlm.nih.gov/)^198^, compounds to be screened, and the SMILES structures for those and for the synthesised compounds to be screened were converted into 3D conformations predicted using OpenBabel software (v2.2.1) (https://pyrx.sourceforge.io/). A cross-docking protocol with numbers associated with each selected protein^199^^,^^200^ was used. The 3D crystallographic structure of the protein targets, including the mushroom tyrosinase structure (PDB ID: 2Y9X; resolution: 1.33 Å), was retrieved from the RCSB PDB web (https://www.rcsb.org/)^201^, and retrieved proteins (receptors) were in complex with other hetero-atoms and water molecules. PyMOL was used to process the downloaded PDB structures further by removing water molecules and heteroatoms (e.g. chloride or sodium ions), and separating the structure of the protein and structure-native ligand (inhibitor, for enzymes) from the complex. The separated native ligand was considered as a positive test control for the docking simulations. Target and ligand structures were then converted to PDBQT files using AutoDock Tools (ADT) from the open-source software suite MGL tools 1.5.6^202^ (Molecular Graphics Laboratory package from The Scripps Research Institute, http://autodock.scripps.edu/). During processing (to prepare a file suitable for docking), polar hydrogen was added to the separated protein structure before converting it to the PDBQT file. There was no need to add polar hydrogen to any ligands, but the torsion angle of each ligand was defined. While OpenBabel software (v2.2.1) (https://pyrx.sourceforge.io/) was used for converting the SMILES format of all the ligands to generate their 3D conformations in PDB format, the program Raccoon^203^. Once the torsion angle was found, it was converted and saved in PDBQT file format and was later used for docking as the ligand.

The grid box was set centred in Cartesian coordinate (11.25–19.88*x*-, 10.88–23.62*y*-, and 11.0018.36*z*) of the docking site, defined according to the pre-complexed ligand (native ligand) of the PDB structures for each target after removing water molecules and hetero atoms for each of the native-ligand-bound:target complex, with a default grid point spacing of 0.375 Å. After preparing all the ligands and targets, the binding affinities were predicted using the graphic user interface (GUI) AutoDock Vina software (v.1.2.0.)^204^ in Linux OS. After the initial docking simulation, 10 other conformations of the docked ligands were obtained, and each ligand with the minimum binding energy and RMSD value was chosen. The ligand:target complexes (ligand–protein interactions) were then visualised using PyMOL^205^ and processed using Discovery Studio (version 3.5, Dassault Systèmes BIOVIA, San Diego, CA). As an example, tyrosinase docking was similar to all other docking simulations. In brief, prior to docking of the test ligands, tropolone, the co-crystallised ligand in the PDB structure, was removed and redocked to the active site. The obtained binding mode was compared with the co-crystallised configuration by determining the root-mean-square deviation (RMSD) as a means of assessing the robustness and validity of the methodology. It was confirmed that the binding pose for tropolone was comparable to that of the native configuration of the PDB structure for the ligand:target complex (RMSD: 1.33 Å).

Predicted binding energies were taken as an index for evaluating potential for a compound to bind specifically to one of the identified targets with appreciable and pharmacologically significant affinity. Values ≤ −8 were considered to be predictive of high-affinity binding, irrespective of the calculated value for the native and/or clinical ligands. The low binding energy (< −8.00 kcal/mol) indicates good binding strength, sufficient to move forward with the predicted target for each derivative.

#### *In silico* predictions for drug-likeness, ADME/pharmacokinetics, and toxicity

The SwissADME server was used for the computational predictions of absorption, distribution, metabolism, and excretion (ADME) and DL properties of each of the newly synthesised compounds (**P1**–**P25**) as described earlier^206^^,^^207^. Gastrointestinal absorption (GA), DL, and other parameters such as oral bioavailability were considered to define the bioactive compounds^123^^,^^206^. The SwissADME online server predictor program (Swiss Institute of Bioinformatics, Écublens, Switzerland) reports various druglikeness indices, including molecular weight, number of H-bond donors and acceptors, total polar surface area (TPSA), number of rotatable bonds, and aggregate Lipinski Rule-of-Fives assessment, as well as further predictions of partition coefficient (as mi*log*P) and even general pharmacokinetic characteristics^123^^,^^206^^,^^208^. For toxicity prediction, the web-tool Pro Tox II (https://tox-new.charite.de/protox_II) was employed for as described earlier^209^^,^^210^. Herein, each ligand was assessed in silico with respect to predicted hepatotoxicity, carcinogenicity, immunotoxicity, cytotoxicity, or mutagenicity, particular organ toxicities, and other endpoints of toxicological interest and prospective concern; this tool even generates LD50 predictions. Data generated with this tool are included with the supplementary data provided in the master Excel spreadsheet.

### Statistical analysis

All statistical analyses were performed using GraphPad Prism Software version 9.1 (GraphPad Prism Inc., San Diego, CA) as previously described^109^, and quantitative and grouped data were expressed as means ± standard deviation (SD) or ± standard error of the mean (SEM) from at least three independent experiments. The significance was analysed by one-way ANOVA, Student’s *t*-test, or ANOVA with Turkey’s or Bonferroni’s multiple comparison *post hoc* tests for the difference between two or more groups. Statistical significance is denoted as: *p values <0.05, **p values < 0.01, ***p values <0.001, and ****p values <0.0001.

## Supplementary Material

Supplemental Material

## Data Availability

The data presented in this study are available at reasonable request from the corresponding authors.
