# Supplementary material for: Synthesis, in silico modelling, and in vitro biological evaluation of substituted pyrazole derivatives as potential anti-skin cancer, anti-tyrosinase, and antioxidant agents
Source: J Enzyme Inhib Med Chem. 2023 May 15;38(1):2205042. doi: 10.1080/14756366.2023.2205042 (PMC10187093; doi:10.1080/14756366.2023.2205042)
Supplement: Supplemental Material [file IENZ_A_2205042_SM9701.pdf]

Supplementary Information

Synthesis, *In-Silico* Modelling and *In-Vitro* Biological Evaluation of Substituted Pyrazole Derivatives as Potential Anti-Skin Cancer, Anti-Tyrosinase, and Antioxidant Agents

Samuel T. Boateng[a, ‡], Tithi Roy[a, ‡], Kara Torrey[b, ‡], Uchechi Owunna[c], Sergette Banang-Mbeumi[a,d], David Basnet[c], Eleonora Niedda[e], Alexis D. Alexander[a], Denzel El Hage[c], Siriki Atchimnaidu[c], Bolni Marius Nagalo[f,g], Dinesh Aryal[a, h], Ann Findley[c], Navindra P. Seeram[b], Tatiana Efimova[,i], Mario Sechi[e], Ronald A. Hill[a], Hang Ma[b], Jean Christopher Chamcheu[a,*], Siva Murru[c,*]

[a.] School of Basic Pharmaceutical and Toxicological Sciences, College of Pharmacy, University of Louisiana at Monroe, Monroe, LA 71209-0497, USA;
[b.] Bioactive Botanical Research Laboratory, Department of Biomedical and Pharmaceutical Sciences, College of Pharmacy, University of Rhode Island, Kingston, RI 02881, USA;
[c.] School of Sciences, College of Arts, Education and Sciences, University of Louisiana at Monroe, Monroe, LA 71209-0497, USA;
[d.] School of Nursing and Allied Health Sciences, Louisiana Delta Community College, Monroe, LA 71203, USA.
[e] Department of Medicine, Surgery and Pharmacy, University of Sassari, 07100 Sassari, Italy;
[f.] Department of Pathology, University of Arkansas for Medical Sciences (UAMS), Little Rock, AR, USA
[g.] The Winthrop P. Rockefeller Cancer Institute, UAMS, Little Rock, AR, USA
[h.] Edward Via College of Osteopathic Medicine, Department of Biomedical Affairs and Research, 4408 Bon Aire Drive, Monroe, LA 71203
[i.] Department of Biomedical Engineering, Northwestern University, Chicago, IL 60611, USA

Running Title: Identification of potent anticancer, antioxidant and anti-tyrosinase pyrazole derivatives

[‡] These authors contributed equally to this work.

*** Corresponding authors:**

Dr. Jean Christopher Chamcheu School of Basic Pharmaceutical and Toxicological Sciences, College of Pharmacy, University of Louisiana - Monroe, 800 Bienville Drive, Room 362, Monroe, LA 71209-0497, USA. Phone: 318-342-6820, Fax: (318) 342 1737; Email: chamcheu@ulm.edu; (ORCID https://orcid.org/0000-0002-3927-5664)

Dr. Siva Murru, School of Sciences, College of Arts, Education and Sciences, University of Louisiana at Monroe, Monroe, LA 71209-0497, USA; *E-mail:* murru@ulm.edu; Phone: 318-342-7966; ORCID https://orcid.org/0000-0002-1956-3079.

Table of Contents Pages

1. **Table S1**: The IC_{50} data from the mushroom tyrosinase inhibitory assay screening of P1-P25. 3
2. **Table S2**: Physicochemical properties of P1-P25. 4
3. **Table S3**: Physicochemical, pharmacokinetics (SwissADME), and toxicological properties of synthesized compounds. 5-6
4. **Table S4**: The IC_{50} data from the dose dependent DPPH antioxidant activity screening of selected active compounds. 7
5. **Table S5**: Computational prediction of some pharmacokinetic properties of P1-P25 8-9
6. **Table S6**: Catalysts and solvents screening for the synthesis of azole compounds. 10
7. ^{1}H- and ^{13}C-NMR, and High-Resolution Mass Spectra. 11-96

Table S1. The summary IC_{50} values of mushroom tyrosinase inhibition assay of pyrazoles, pyrazolones, isoxazoles and positive control drugs P1-P25 > 500 = IC_{50} Value is greater than 500 μM, and therefore not biologically relevant.

**Sample Identity**	**IC_{50} Value** (μM)
P1	> 500
P2	> 500
P3	> 500
P4	196.9
P5	> 500
P6	> 500
P7	> 500
P8	190.4
P9	> 500
P10	> 500
P11	172.8
P12	335.5
P13	> 500
P14	15.9
P15	> 500
P16	> 500
P17	165.4
P18	156.0
P19	> 500
P20	> 500
P21	294.8
P22	> 500
P23	> 500
P24	> 500
P25	> 500
Arbutin	91
Kojic acid	31

Table S2. Physicochemical properties of pyrazoles, pyrazolones, and isoxazoles P1-25

Compounds	MW	#Heavy atoms	#Aromatic heavy atoms	Fraction Csp3	#Rotatable bonds		#H-bond acceptors	#H-bond donors	MR
**P1**	124.18	9	5	0.57	1		1	1	38.29
**P2**	122.17	9	5	0.57	0		1	1	36.18
**P3**	158.2	12	6	0.2	1		2	0	57.74
**P4**	192.64	13	11	0.1	1		1	1	54
**P5**	184.24	14	11	0.25	0		1	1	56.81
**P6**	182.22	14	13	0.08	0		1	1	58.57
**P7**	200.28	15	11	0.31	2		1	0	63.27
**P8**	198.26	15	11	0.31	1		1	0	61.16
**P9**	260.33	20	17	0.17	1		1	0	81.78
**P10**	244.29	18	11	0.29	4		3	0	69.58
**P11**	159.18	12	11	0.1	1		2	0	46.91
**P12**	185.22	14	11	0.25	0		2	0	54.72
**P13**	296.37	23	23	0	3		1	0	94.44
**P14**	234.3	18	17	0.06	2		1	0	73.97
**P15**	288.27	21	17	0.06	3		4	0	74
**P16**	313.19	19	17	0.06	2		1	0	81.67
**P17**	268.74	19	17	0.06	2		1	0	78.98
**P18**	264.32	20	17	0.12	3		2	0	80.46
**P19**	313.37	22	17	0.06	3		4	1	84.96
**P20**	188.23	14	6	0.27	1		2	0	63.1
**P21**	236.27	18	12	0.07	2		2	0	77.98
**P22**	394.06	20	12	0.07	2		2	0	93.37
**P23**	383.16	23	12	0.12	3		5	0	90.68
**P24**	315.16	19	12	0.07	2		2	0	85.67
**P25**	315.16	19	12	0.07	2		2	0	85.67

Table S3: Physicochemical, pharmacokinetics (SwissADME), and toxicologic properties of the synthesized compounds.

			Lipophilicity						Drug-likeness (number of violations)					Water Solubility		Pharmacokinetics	
**Comp.**	**MW** g/mol	TPSA	ilogP	Xlog P3	Wlog P	Mlog P	Silicos -IT logP	Consensus logP	Lipinski	Ghose	Veber	Egan	Muegge	ESOL Log S	ESOL Class	Log K_p (cm/s)	F
**1**	124.18	28.68	1.32	1.81	1.59	1.08	2.55	1.67	0	2	0	0	1	-2.1	Soluble	-5.77	0.55
**2**	122.17	28.68	1.23	1.3	1.21	1.08	2.65	1.49	0	3	0	0	1	-1.83	Very soluble	-6.12	0.55
**3**	158.2	24.72	1.69	1.76	1.49	1.81	3.79	2.11	0	1	0	0	1	-2.23	Soluble	-6.02	0.55
**4**	192.64	28.68	1.75	2.91	3.04	2.33	3.66	2.74	0	0	0	0	1	-3.43	Soluble	-5.41	0.55
**5**	184.24	28.68	1.64	2.57	2.48	2.34	3.7	2.55	0	0	0	0	1	-3.18	Soluble	-5.6	0.55
**6**	182.22	28.68	1.63	3.2	3.02	2.45	3.53	2.77	0	0	0	0	1	-3.67	Soluble	-5.14	0.55
**7**	200.28	17.82	2.74	3.49	3.05	2.88	3.27	3.09	1	0	0	0	0	-3.69	Soluble	-5.04	0.55
**8**	198.26	17.82	2.56	2.98	2.67	2.88	3.13	2.84	0	0	0	0	1	-3.42	Soluble	-5.39	0.55
**9**	260.33	17.82	3.07	4.25	3.95	3.89	4.19	3.87	0	0	0	0	0	-4.69	Moderate	-4.87	0.55
**10**	244.29	44.12	2.99	3.13	2.67	2.41	2.69	2.78	0	0	0	0	0	-3.51	Soluble	-5.57	0.55
**11**	159.18	26.03	2.22	2.43	2.65	1.77	2.93	2.4	0	1	0	0	1	-2.97	Soluble	-5.55	0.55
**12**	185.22	26.03	2.39	2.73	2.75	2.34	3.57	2.76	0	0	0	0	1	-3.29	Soluble	-5.49	0.55
**13**	296.37	17.82	3.4	5.22	5.21	4.35	4.58	4.55	1	0	0	0	1	-5.51	Moderate	-4.4	0.55
**14**	234.3	17.82	2.91	3.95	3.85	3.41	3.55	3.53	0	0	0	0	0	-4.35	Moderate	-4.92	0.55
**15**	288.27	17.82	2.94	4.47	5.71	4.04	4.02	4.24	0	1	0	0	0	-4.84	Moderate	-4.88	0.55
**16**	313.19	17.82	3.3	4.64	4.61	4.04	4.19	4.16	0	0	0	0	0	-5.24	Moderate	-4.92	0.55
**17**	268.74	17.82	3.18	4.58	4.5	3.92	4.16	4.07	0	0	0	0	0	-4.92	Moderate	-4.69	0.55
**18**	264.32	27.05	3.23	3.92	3.86	3.04	3.55	3.52	0	0	0	0	0	-4.38	Moderate	-5.13	0.55
**19**	313.37	86.36	2.21	2.52	3.58	1.78	1.58	2.33	0	0	0	0	0	-3.74	Soluble	-6.42	0.55
**20**	188.23	32.67	2.3	1.84	1.28	1.89	2.24	1.91	0	0	0	0	1	-2.42	Soluble	-6.14	0.55
**21**	236.27	32.67	2.55	2.93	2.07	2.73	3.27	2.71	1	0	0	0	0	-3.51	Soluble	-5.66	0.55
**22**	394.06	32.67	3.3	4.31	3.59	3.99	4.57	3.95	0	0	0	0	0	-5.31	Moderate	-5.64	0.55

**23**	383.16	32.67	3.19	4.5	5	4.22	4.91	4.37	1	0	0	0	0	-5.24	Moderate	-5.44	0.55
**24**	315.16	32.67	2.9	3.62	2.83	3.37	3.92	3.33	0	0	0	0	0	-4.41	Moderate	-5.65	0.55
**25**	315.16	32.67	2.96	3.62	2.83	3.37	3.92	3.34	0	0	0	0	0	-4.41	Moderate	-5.65	0.55

Molecular weight (MW), LogP values (an indicator of lipophilicity), aqueous solubility parameters (ESOL), an indicator of skin permeation (Log K_p), and bioavailability score (F) HBA: number of hydrogen bond acceptor; HBD: number of hydrogen bond donor, LogP: partition coefficient; LogS: lipophilicity of water; PSA: total polar surface area; RO5: Lipinski's rule of 5

Table S4. Summary IC_{50} data of the DPPH antioxidant activity screening of pyrazoles, pyrazolones and isoxazoles drugs P1-P25. N.A. = No Activity, > 1000 = IC_{50} Value is greater than 1000 μM, and therefore not biologically relevant.

**Sample Identity**	**IC_{50} Value (**μM)
P1	> 1000
P2	N.A.
P3	N.A.
P4	N.A.
P5	N.A.
P6	30.56
P7	> 1000
P8	N.A.
P9	>1000
P10	N.A.
P11	N.A.
P12	N.A.
P13	>1000
P14	N.A.
P15	N.A.
P16	N.A.
P17	N.A.
P18	N.A.
P19	N.A.
P20	N.A.
P21	354.5
P22	276.3
P23	196.4
P24	444.4
P25	189.6

Table S5. Computational prediction of some pharmacokinetic properties of the pyrazoles, pyrazolones, and isoxazoles P1-P25.

Compound	GI absorption	BBB permeant	Pgp substrate	CYP1A2 inhibitor	CYP2C19 inhibitor	CYP2C9 inhibitor	CYP2D6 inhibitor	CYP3A4 inhibitor
P1	High	Yes	No	No	No	No	No	No
P2	High	Yes	Yes	No	No	No	No	No
P3	High	Yes	No	Yes	No	No	No	No
P4	High	Yes	No	Yes	No	No	No	No
P5	High	Yes	Yes	Yes	No	No	No	No
P6	High	Yes	Yes	Yes	No	No	No	No
P7	High	Yes	No	Yes	Yes	No	Yes	No
P8	High	Yes	Yes	Yes	No	No	No	No
P9	High	Yes	Yes	Yes	Yes	No	Yes	No
P10	High	Yes	No	Yes	Yes	Yes	No	No
P11	High	Yes	No	Yes	No	No	No	No
P12	High	Yes	Yes	Yes	No	No	No	No
P13	High	Yes	Yes	Yes	Yes	No	Yes	Yes
P4	High	Yes	No	Yes	Yes	No	Yes	No
P15	High	No	No	Yes	Yes	No	Yes	No
P16	High	Yes	No	Yes	Yes	Yes	Yes	No
P17	High	Yes	No	Yes	Yes	Yes	Yes	No
P18	High	Yes	No	Yes	Yes	Yes	Yes	Yes
P19	High	No	No	Yes	No	No	No	Yes
P20	High	Yes	No	Yes	No	No	No	No
P21	High	Yes	No	Yes	Yes	No	No	No
P22	High	Yes	No	Yes	Yes	Yes	No	No
P23	High	Yes	No	Yes	Yes	Yes	No	No
P24	High	Yes	No	Yes	Yes	Yes	No	No
P25	High	Yes	No	Yes	Yes	Yes	No	No

Table S6. Screening of Base-metal catalysts with different solvents

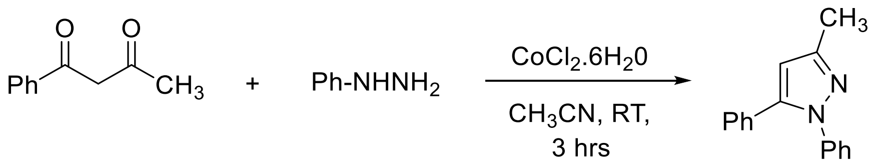

**S. No.**	**Catalyst**	**Solvent**	**% Yield**
1	$Ni^{II}Cl_2$	CH_3CN	53
2	$Ni^{II}(OTf)_2$	CH_3CN	43
3	$Ni^{II}(acac)_2$	CH_3CN	69
4	$Co^{II}Cl_2.6H_2O$	CH_3CN	~100
5	$Co^{II}SO_4$	CH_3CN	94
6	$Cu^{II}(BF_4)_2.H_2O$	CH_3CN	90
7	$Cu^{II}(OTf)_2$	CH_3CN	94
8	No catalyst	CH_3CN	11
9	$Co^{II}Cl_2.6H_2O$	CH_2Cl_2	94
10	$Co^{II}Cl_2.6H_2O$	Dioxane	86
11	$Co^{II}Cl_2.6H_2O$	DMF	60
12	$Co^{II}Cl_2.6H_2O$	Methanol	>99

^{1}H- and ^{13}C-NMR, and Mass Spectra of the Products P1-P19. We confirmed the products P20-P25 by comparing the data we reported earlier (ref#79 in the manuscript).

4-ethyl-3,5-dimethyl-1H-pyrazole (P1): ^{1}H-NMR ($CDCl_3$)

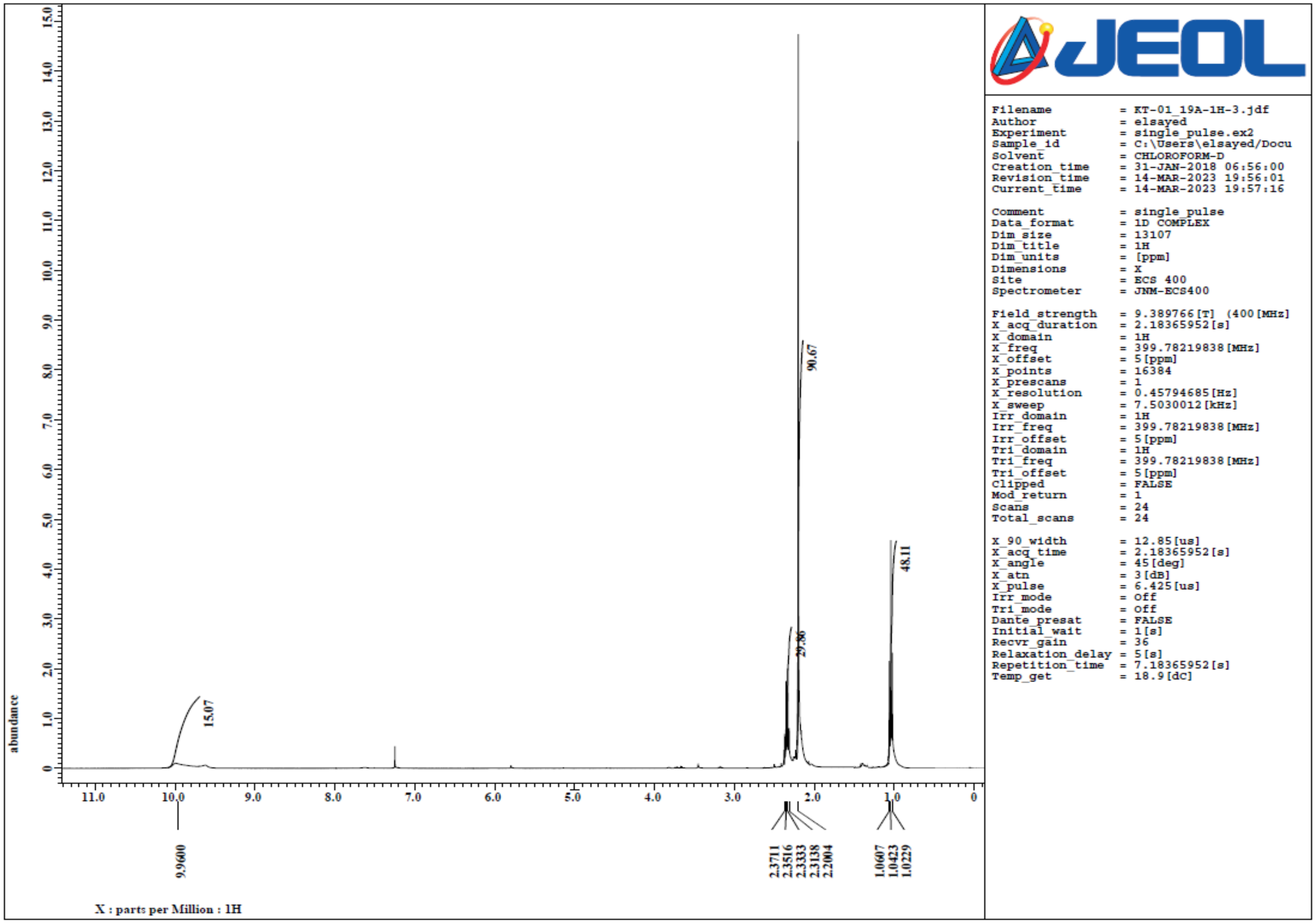

4-ethyl-3,5-dimethyl-1H-pyrazole (P1): ^{13}C-NMR ($CDCl_3$)

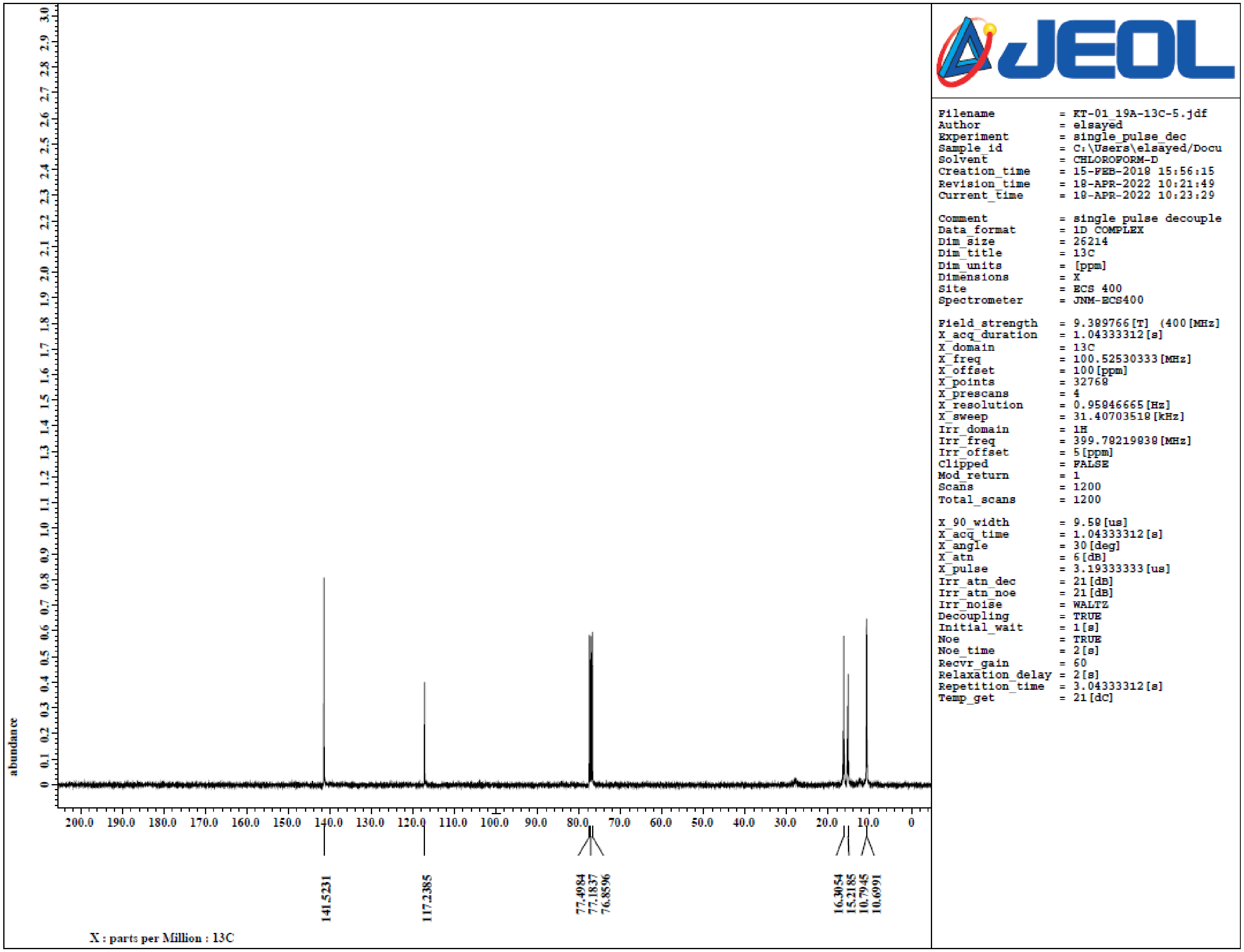

4-ethyl-3,5-dimethyl-1H-pyrazole (P1): ESI-MS

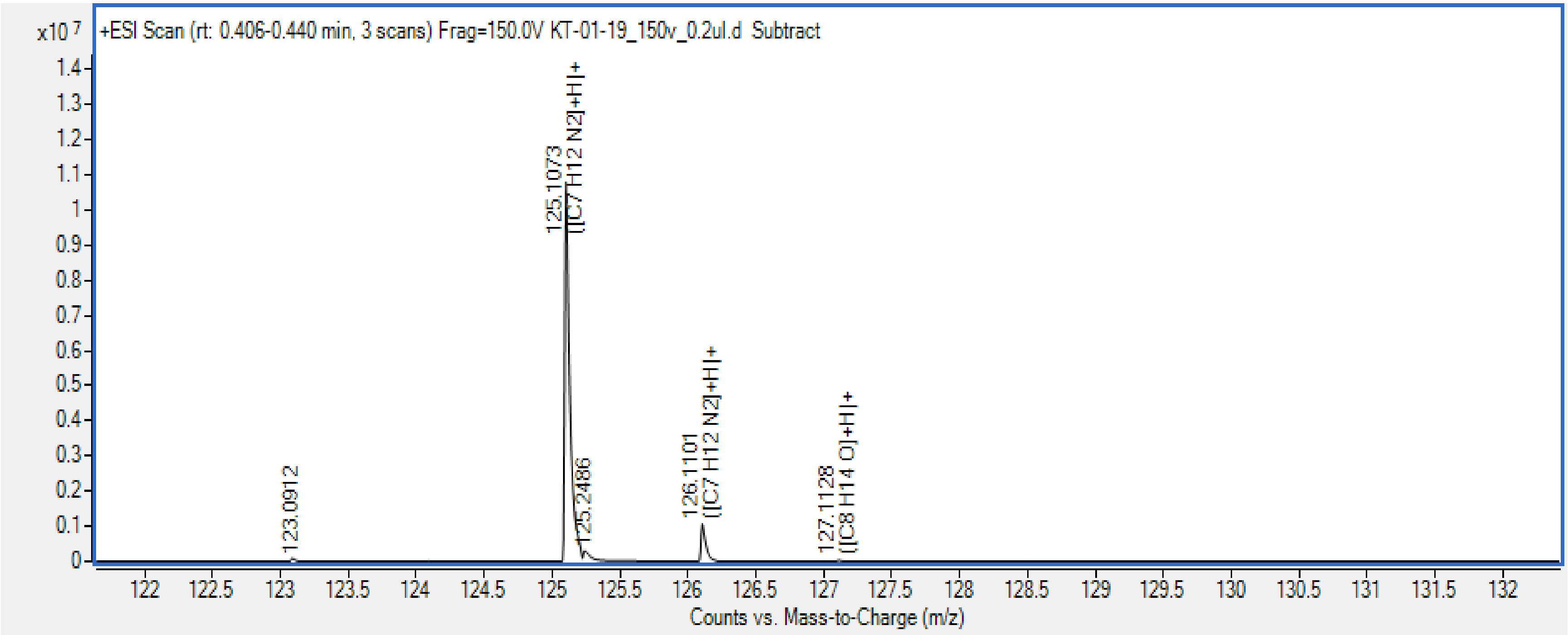

Formula,	Species,	m/z,	Score,	Diff (abs. ppm),	Mass
C7 H12 N2,	(M+H)+,	125.1073,	87.02,	0.29,	124.1

3-methyl-1,4,5,6-tetrahydrocyclopenta[c]pyrazole (P2): ^{1}H-NMR ($CDCl_3$)

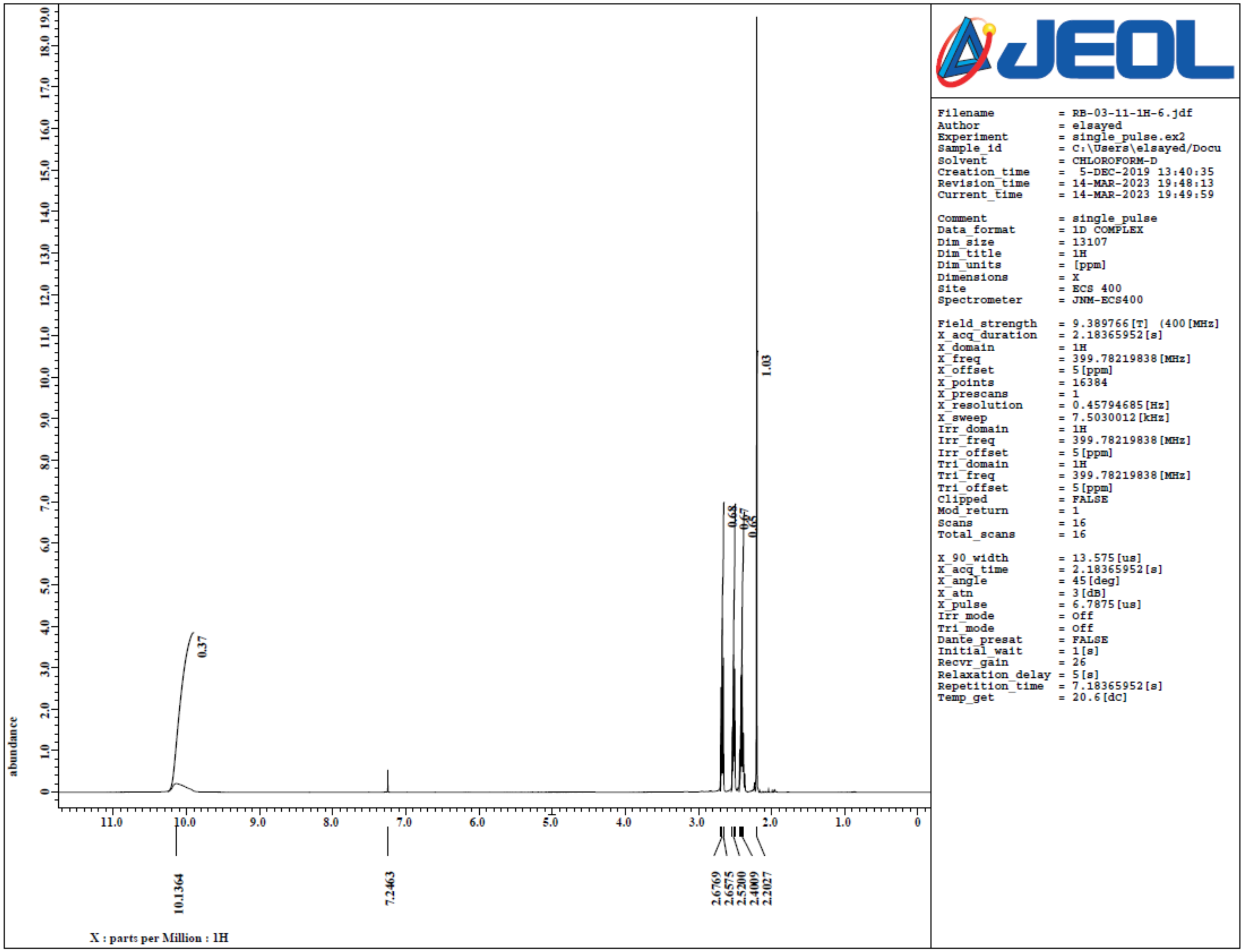

3-methyl-1,4,5,6-tetrahydrocyclopenta[c]pyrazole (P2): ^{1}H-NMR ($CDCl_3$) _ Expanded Aliphatic Region

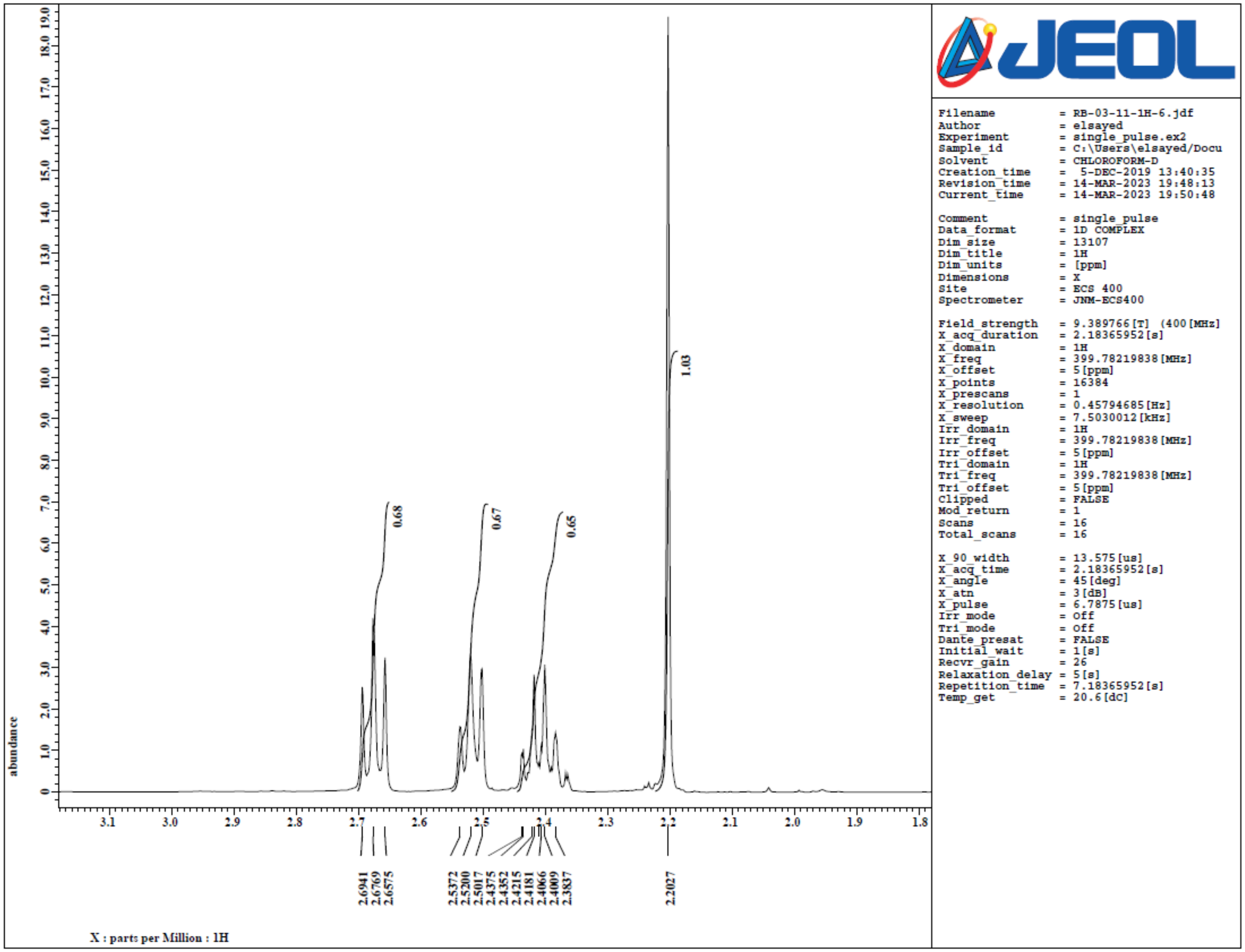

3-methyl-1,4,5,6-tetrahydrocyclopenta[c]pyrazole (P2): ^{13}C-NMR ($CDCl_3$)

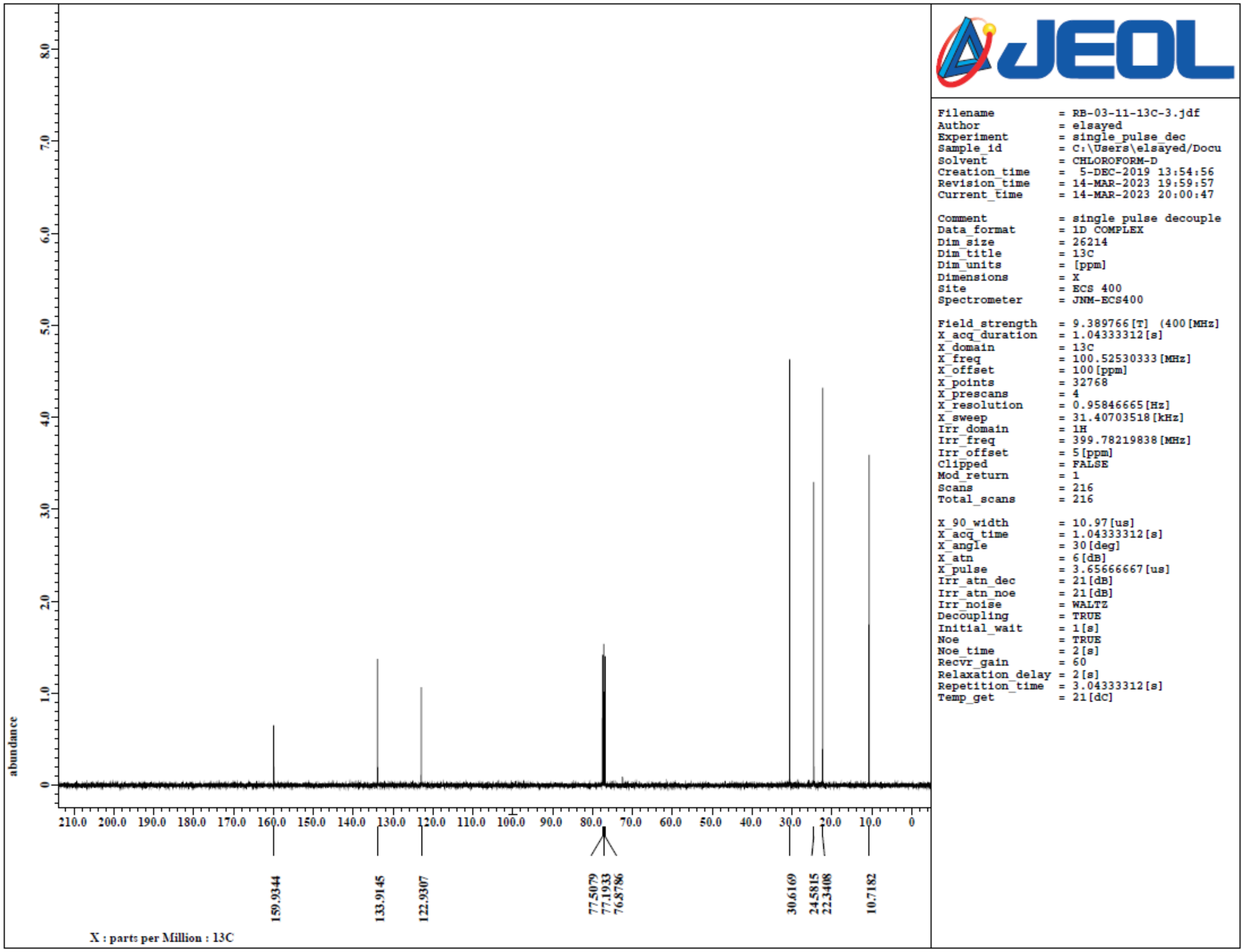

3-methyl-1,4,5,6-tetrahydrocyclopenta[c]pyrazole (P2): ESI-MS

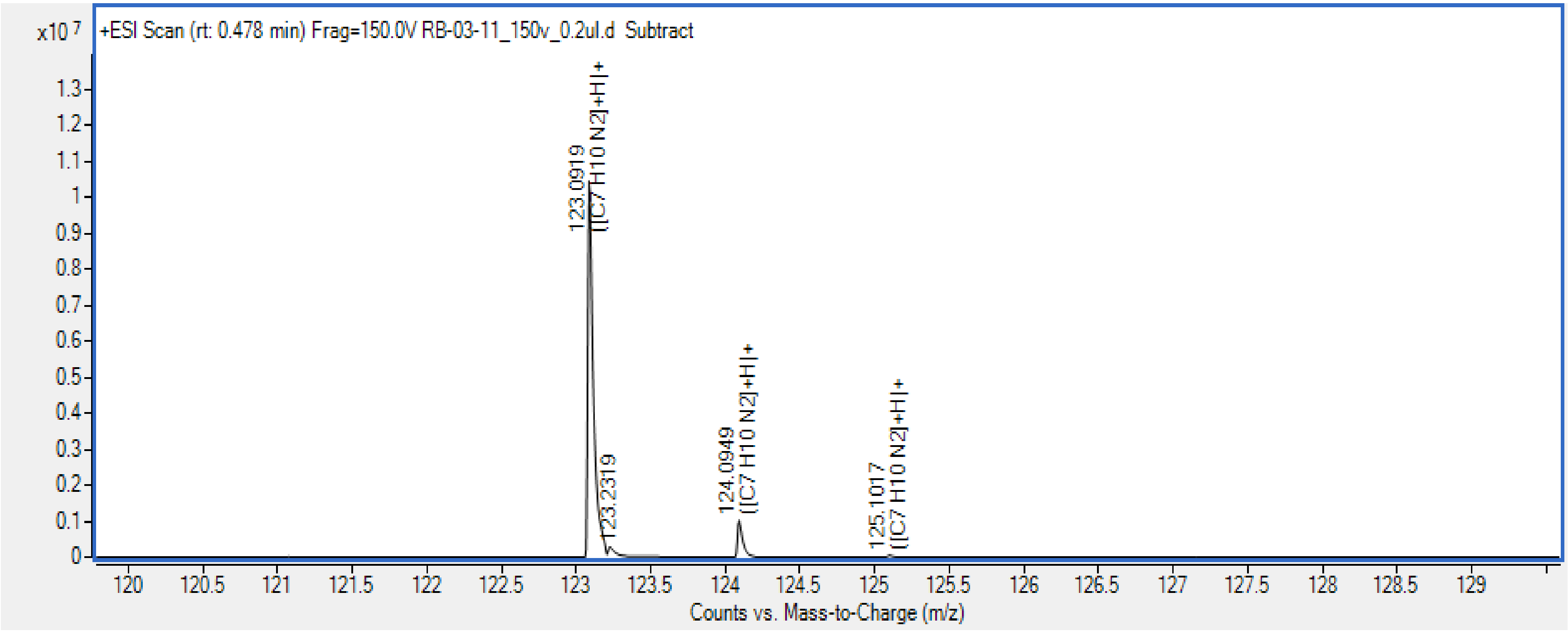

Formula,	Species,	m/z,	Score,	Diff (abs. ppm),	Mass
C7 H10 N2,	(M+H)+,	123.0919,	99.11,	1.85,	122.0846

5-methyl-3-phenyl-1H-pyrazole (P3): ^{1}H-NMR ($CDCl_3$)

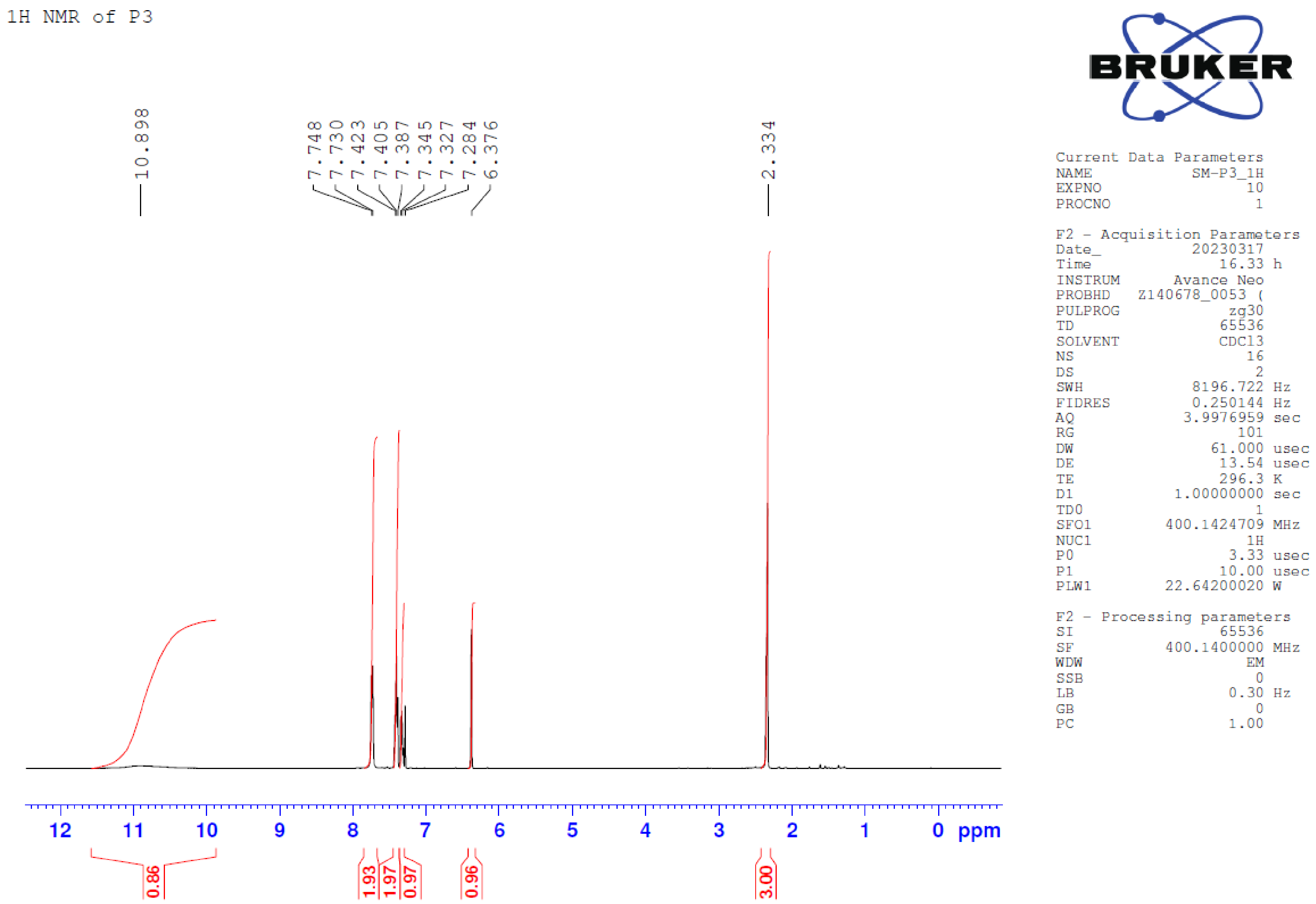

5-methyl-3-phenyl-1H-pyrazole (P3): ^{1}H-NMR ($CDCl_3$) _ Expanded Aromatic Region

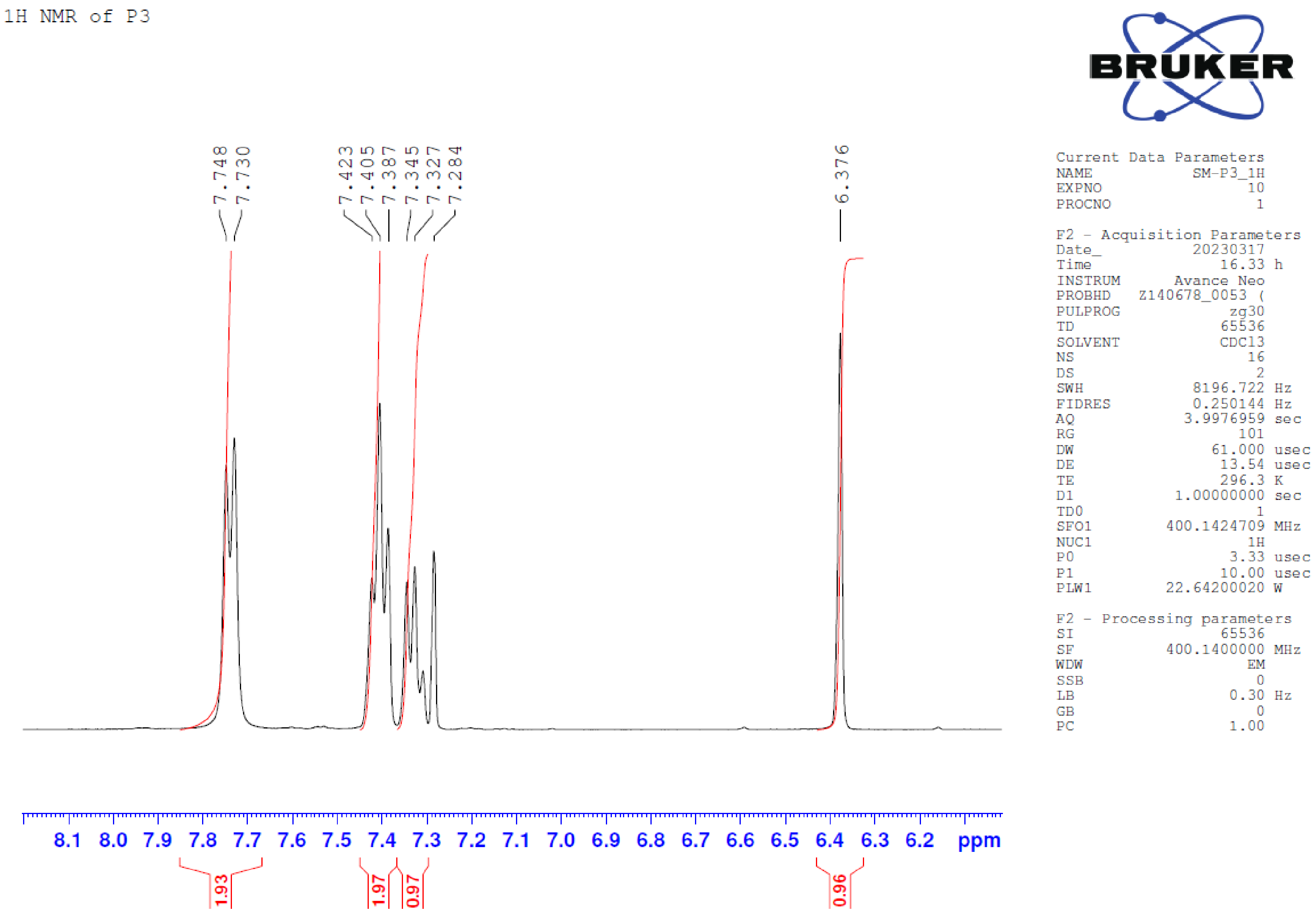

5-methyl-3-phenyl-1H-pyrazole (P3): ^{13}C-NMR ($CDCl_3$)

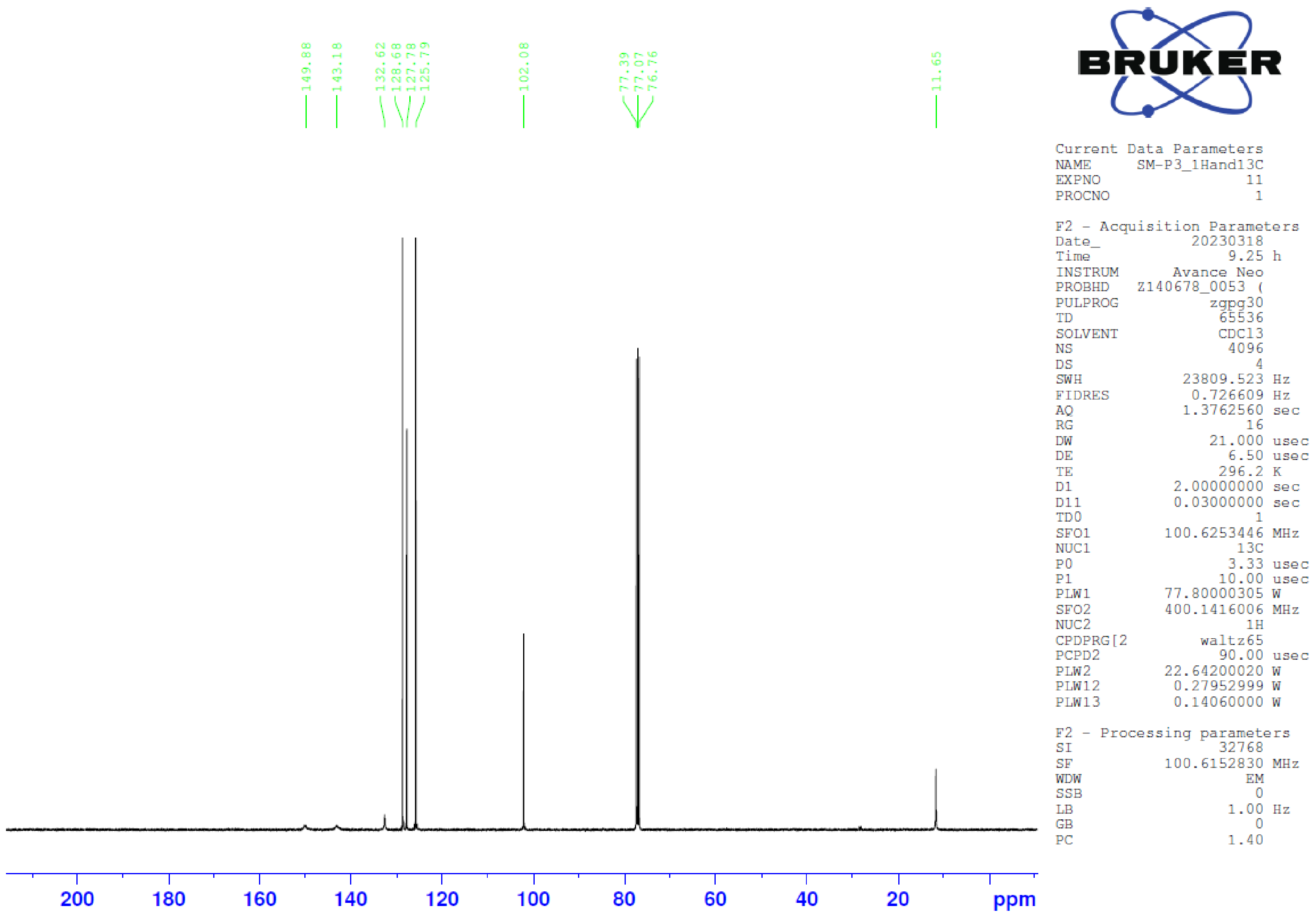

5-methyl-3-phenyl-1H-pyrazole (P3): ESI-MS

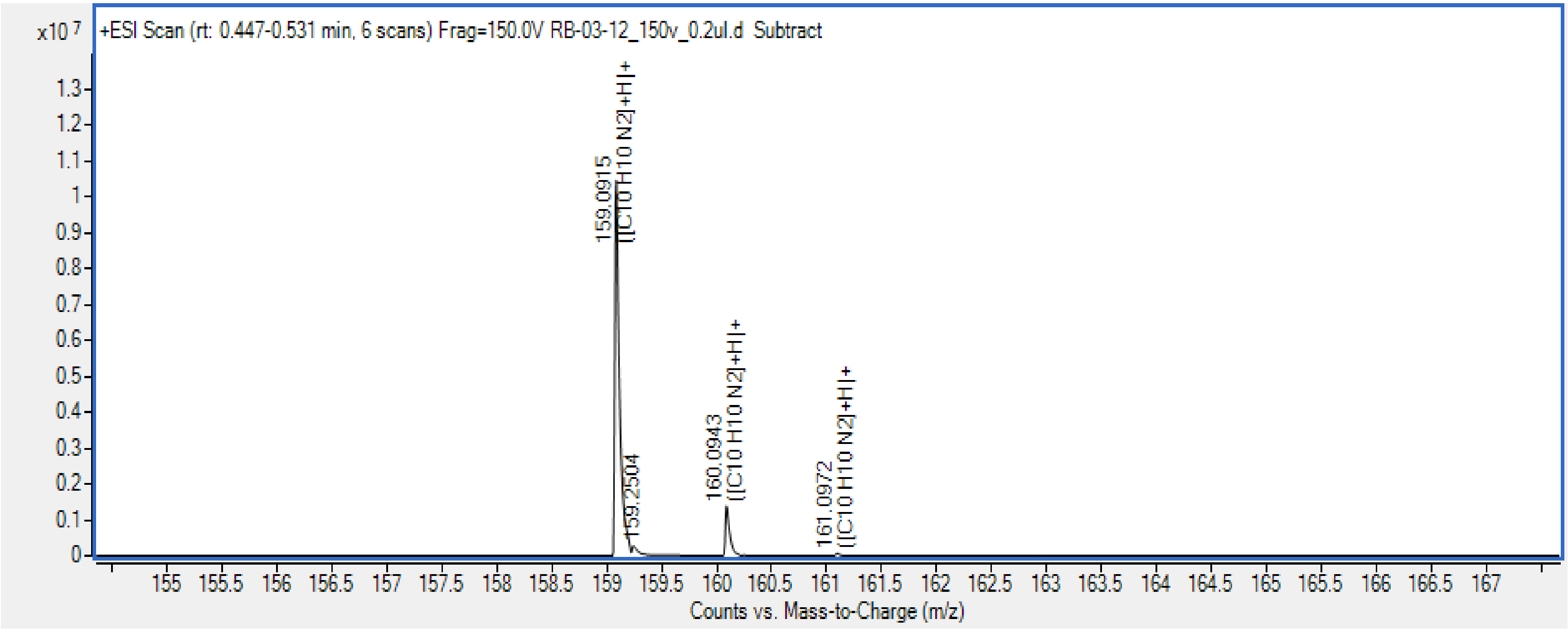

Formula,	Species,	m/z,	Score,	Diff (abs. ppm),	Mass
C10 H10 N2,	(M+H)+,	159.0915,	98.7,	1.37,	158.0842

4-chloro-5-methyl-3-phenyl-1H-pyrazole (P4): ^{1}H-NMR ($CDCl_3$)

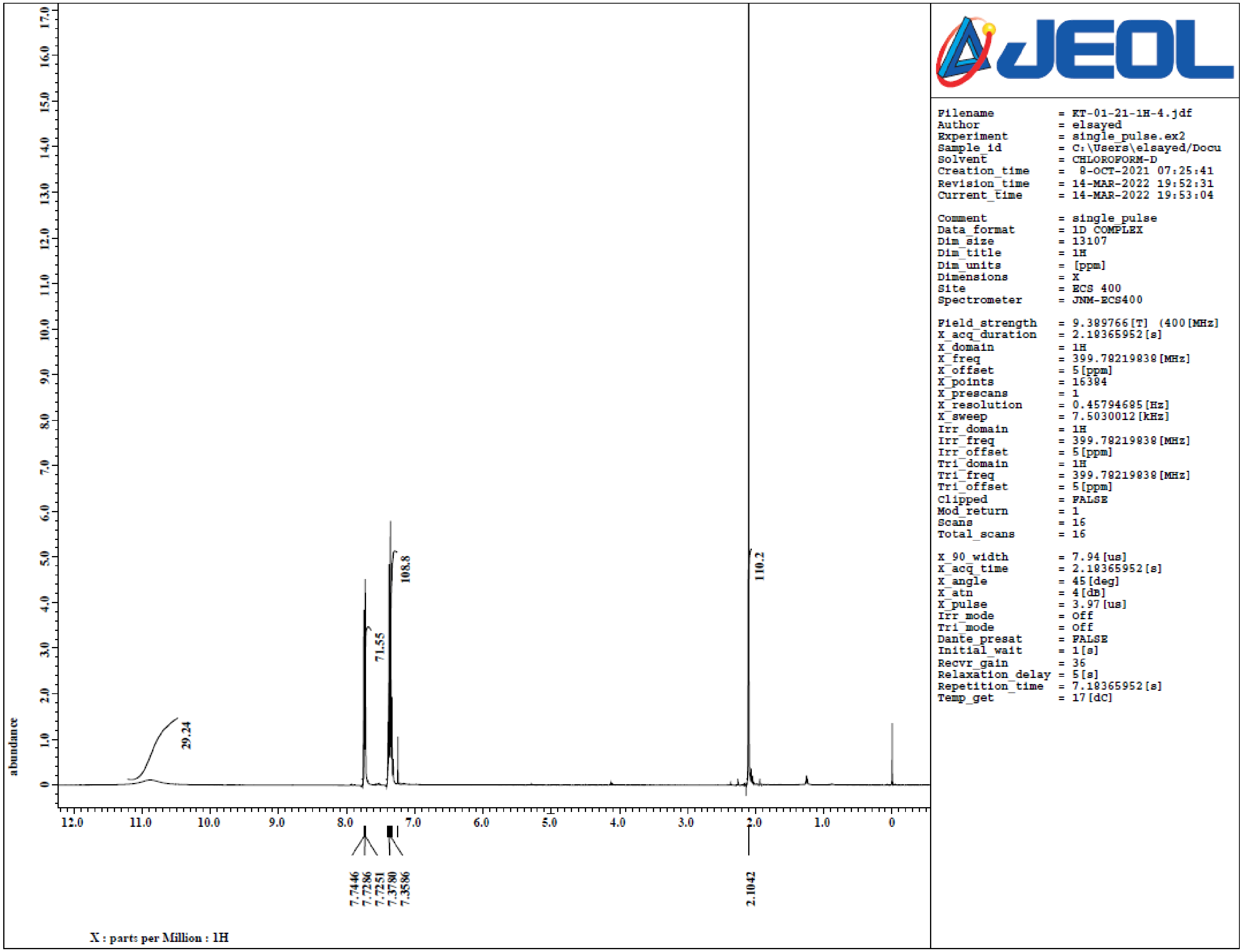

4-chloro-5-methyl-3-phenyl-1H-pyrazole (P4): ^{1}H-NMR ($CDCl_3$) _ Expanded Aromatic Region

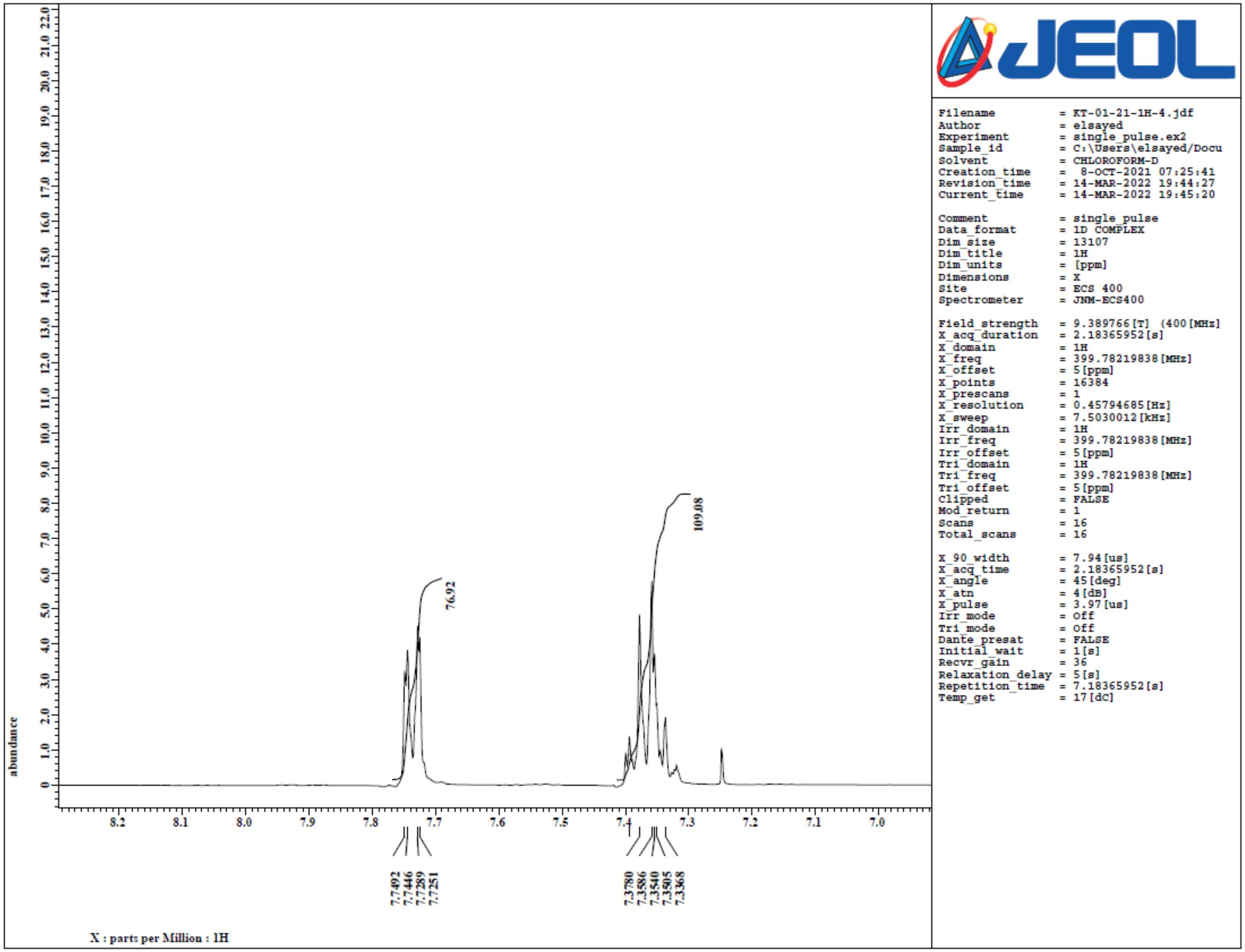

4-chloro-5-methyl-3-phenyl-1H-pyrazole (P4): ^{13}C-NMR ($CDCl_3$)

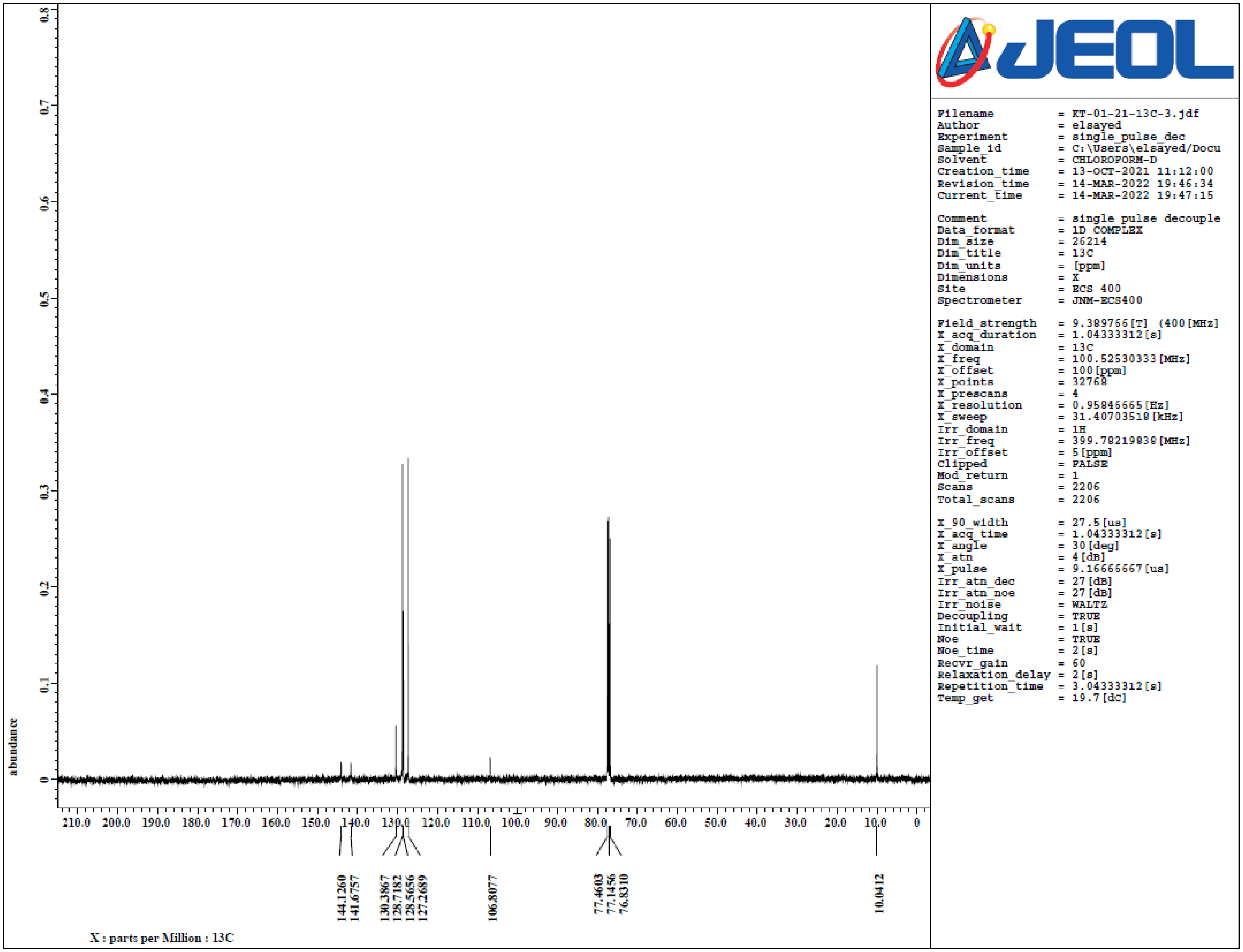

4-chloro-5-methyl-3-phenyl-1H-pyrazole (P4): ESI-MS

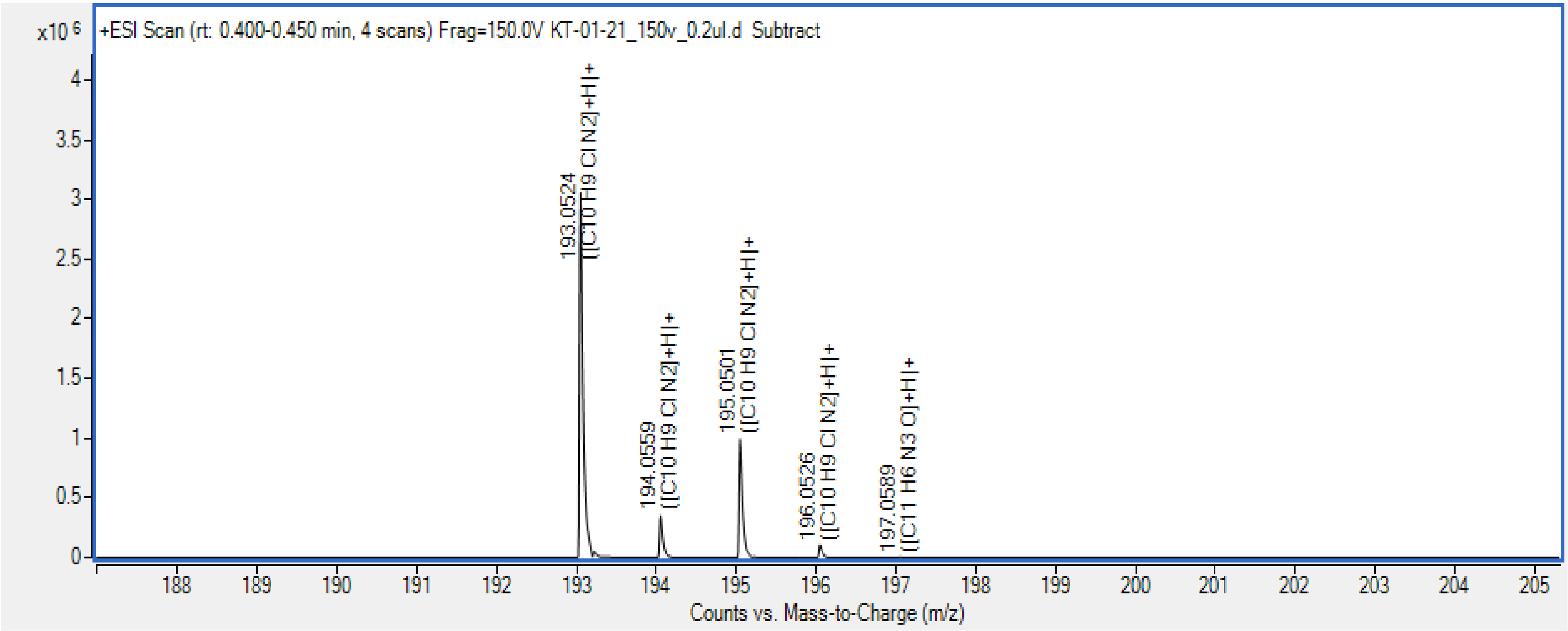

Formula,	Species,	m/z,	Score,	Diff (abs. ppm),	Mass
C10 H9 Cl N2,	(M+H)+,	193.0524,	99.25,	0.82,	192.0453

3-methyl-4,5-dihydro-2H-benzo[g]indazole (P5): ^{1}H-NMR ($CDCl_3$)

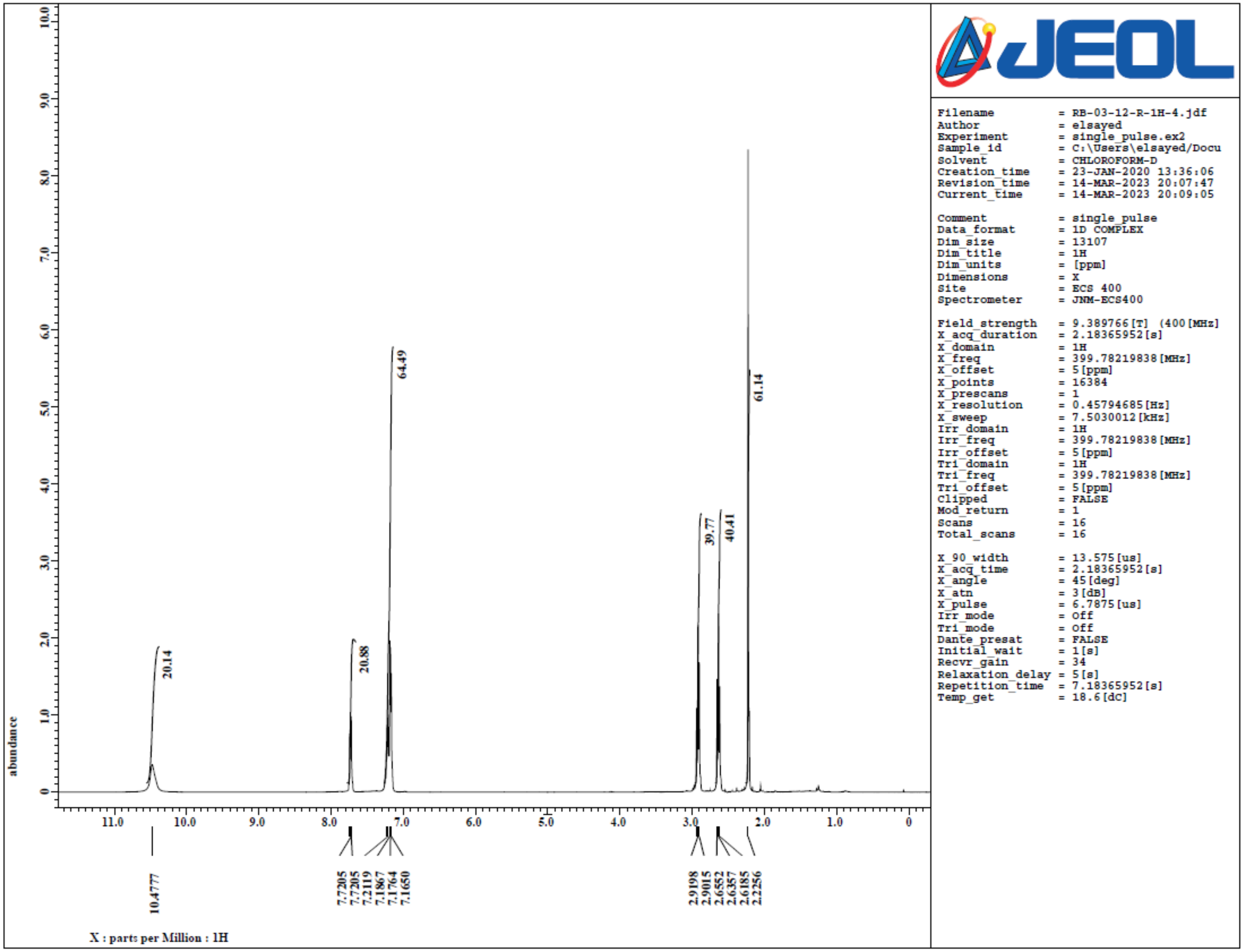

3-methyl-4,5-dihydro-2H-benzo[g]indazole (P5): ^{1}H-NMR ($CDCl_3$) _ Expanded Aromatic Region

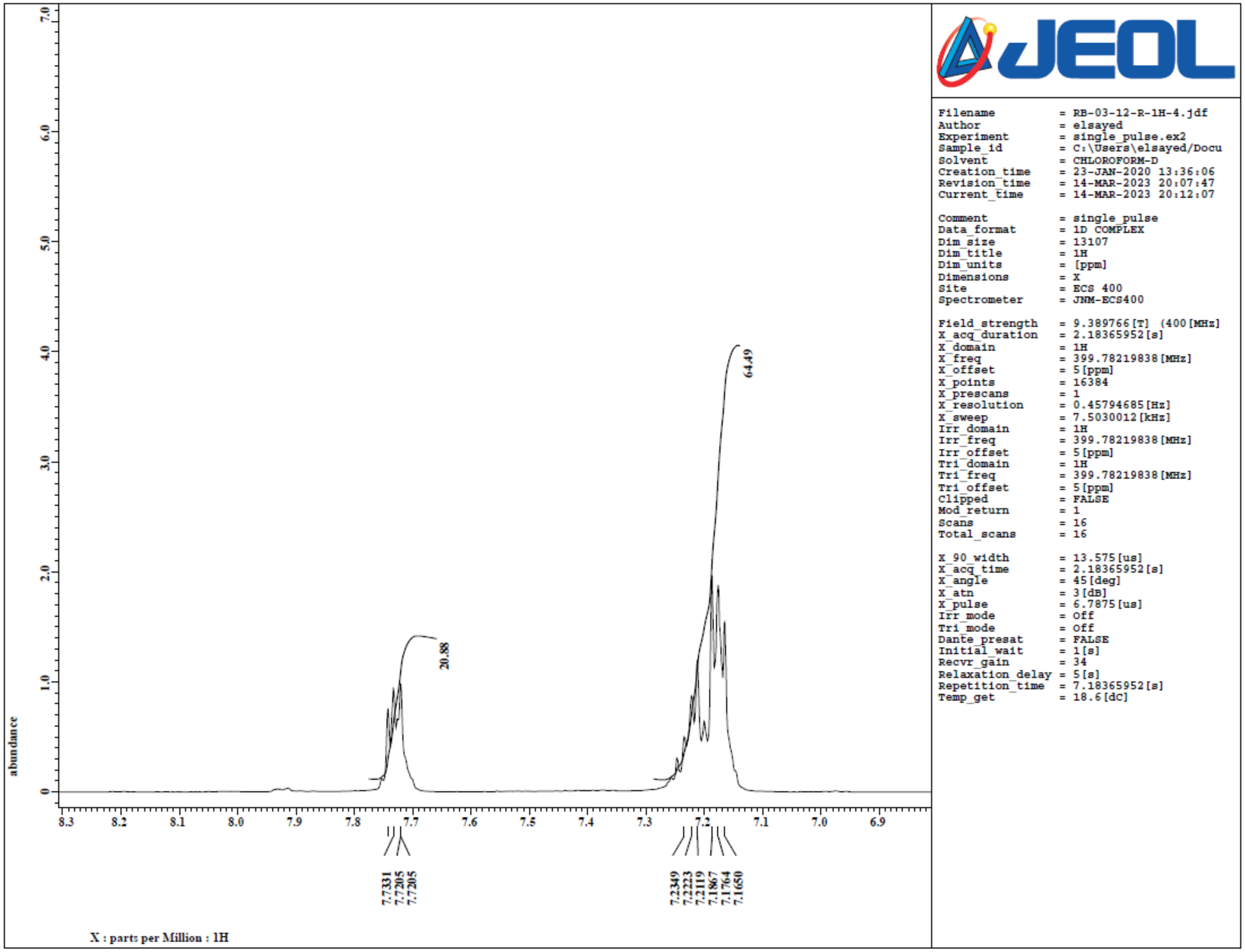

3-methyl-4,5-dihydro-2H-benzo[g]indazole (P5): ¹H-NMR ($CDCl_3$) _ Expanded Aliphatic Region

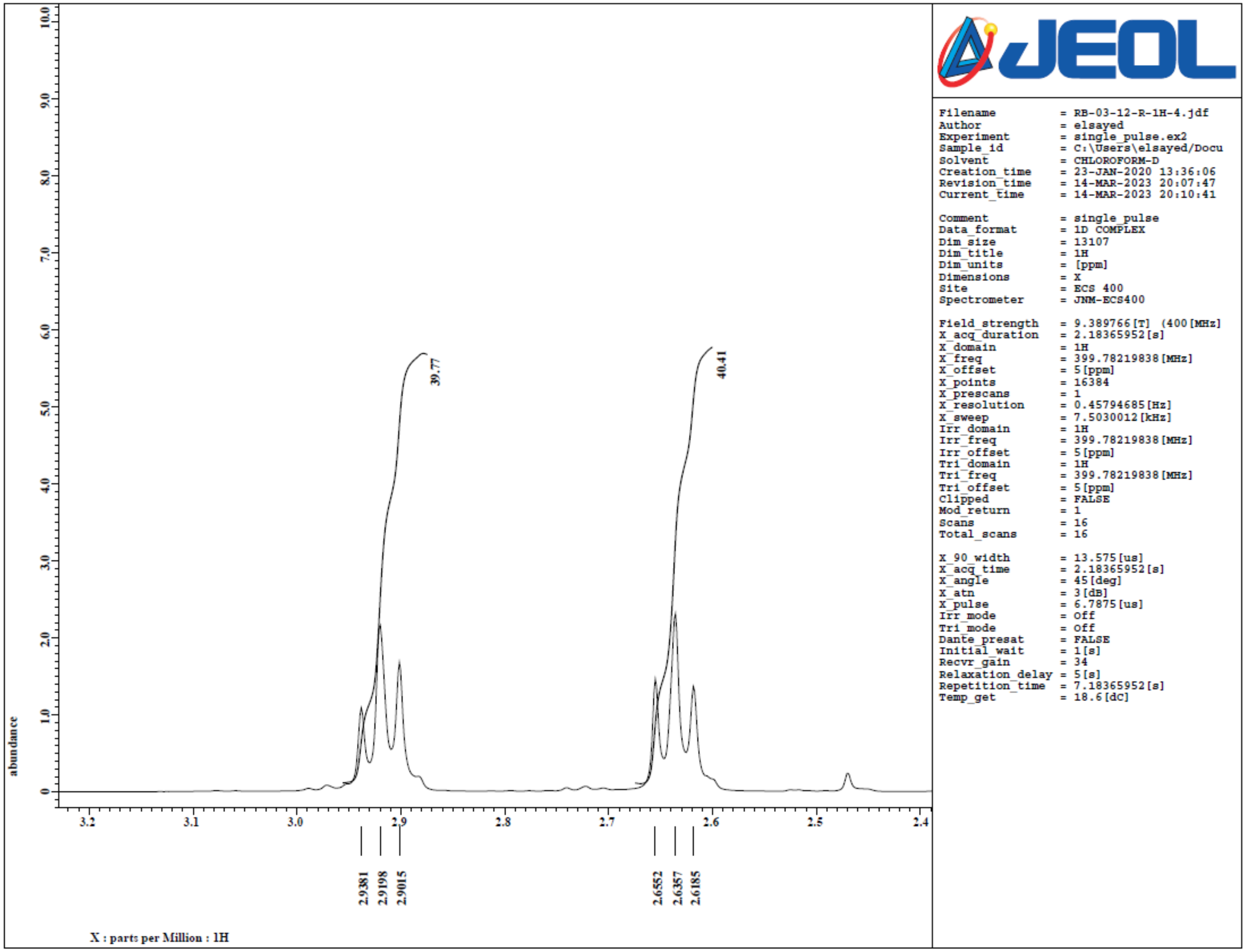

3-methyl-4,5-dihydro-2H-benzo[g]indazole (P5): ^{13}C-NMR ($CDCl_3$)

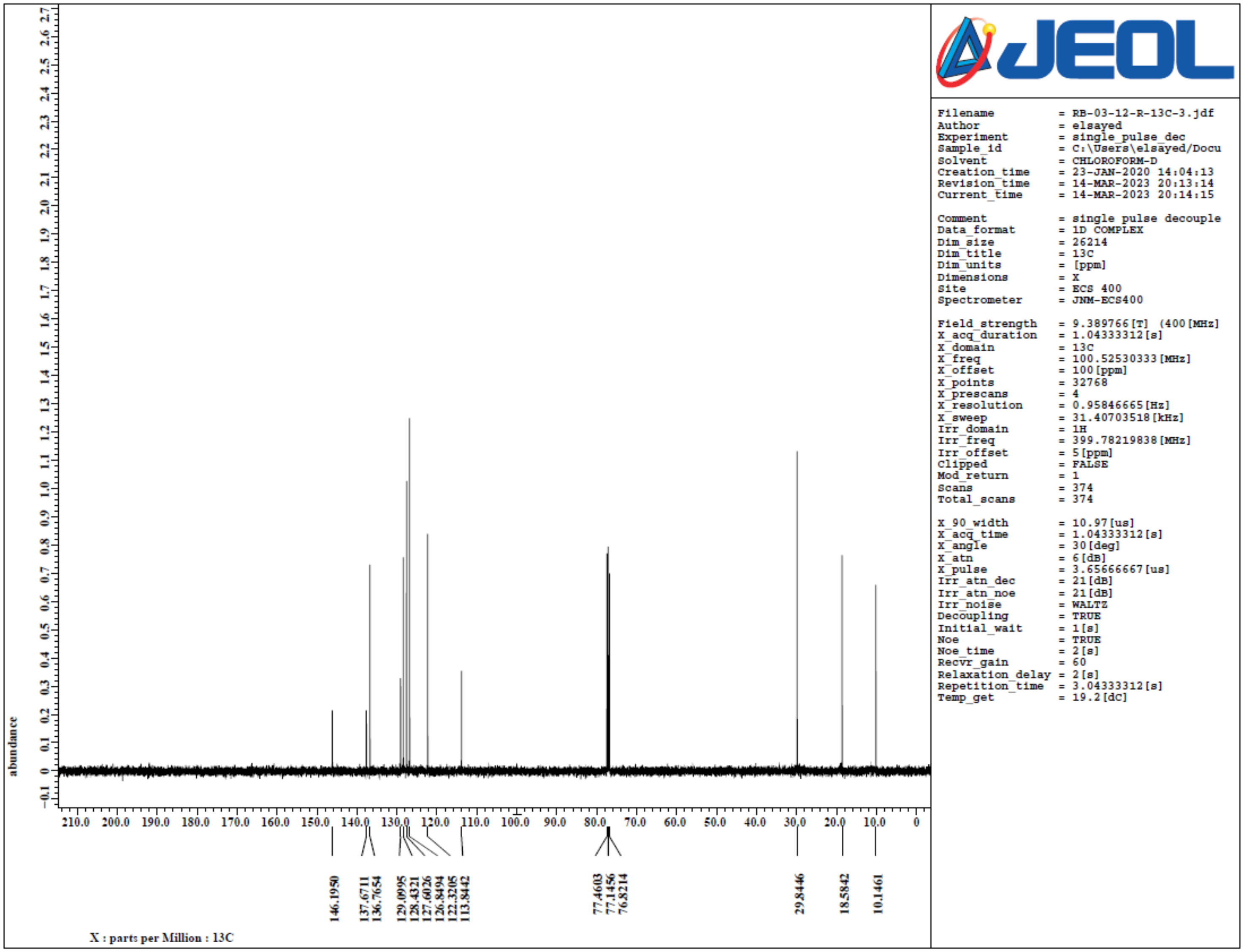

3-methyl-4,5-dihydro-2H-benzo[g]indazole (P5): ESI-MS

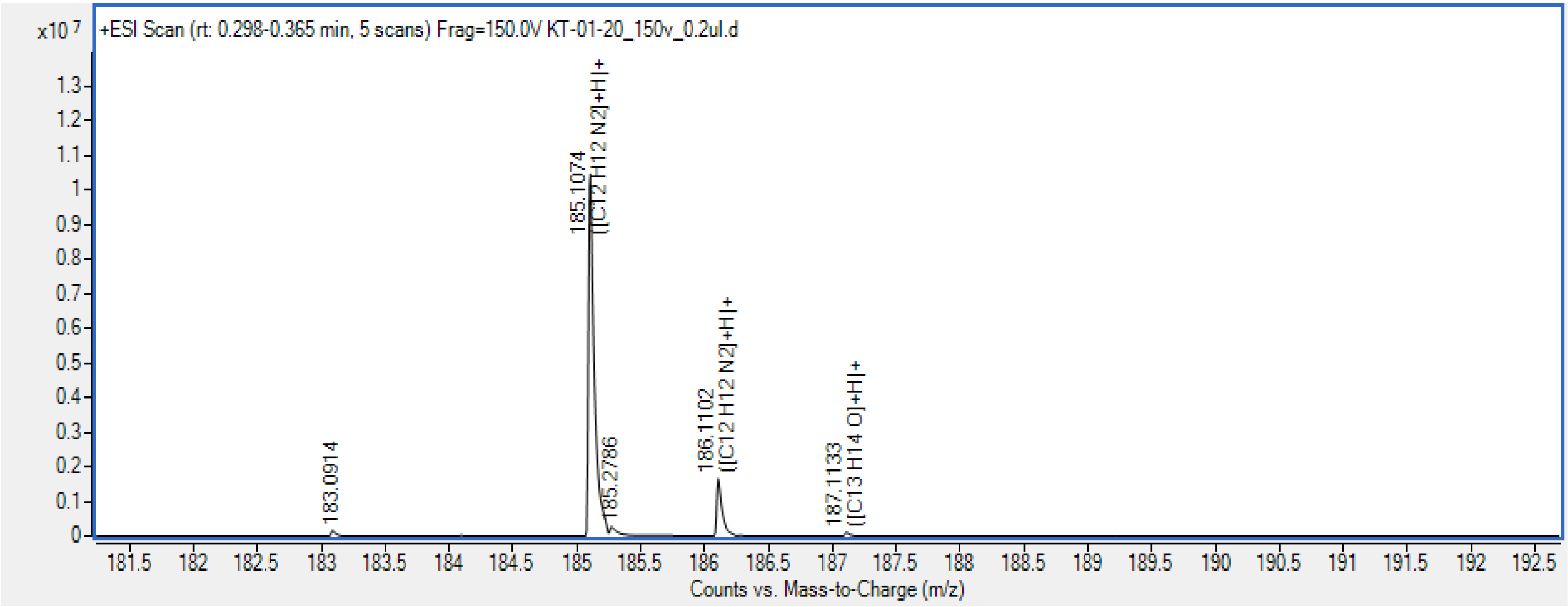

Formula,	Species,	m/z,	Score,	Diff (abs. ppm),	Mass
C12 H12 N2,	(M+H)+,	185.1074,	86.32,	0.04,	184.1001

3-methyl-2H-benzo[g]indazole (P6): ^{1}H-NMR ($CDCl_3$)

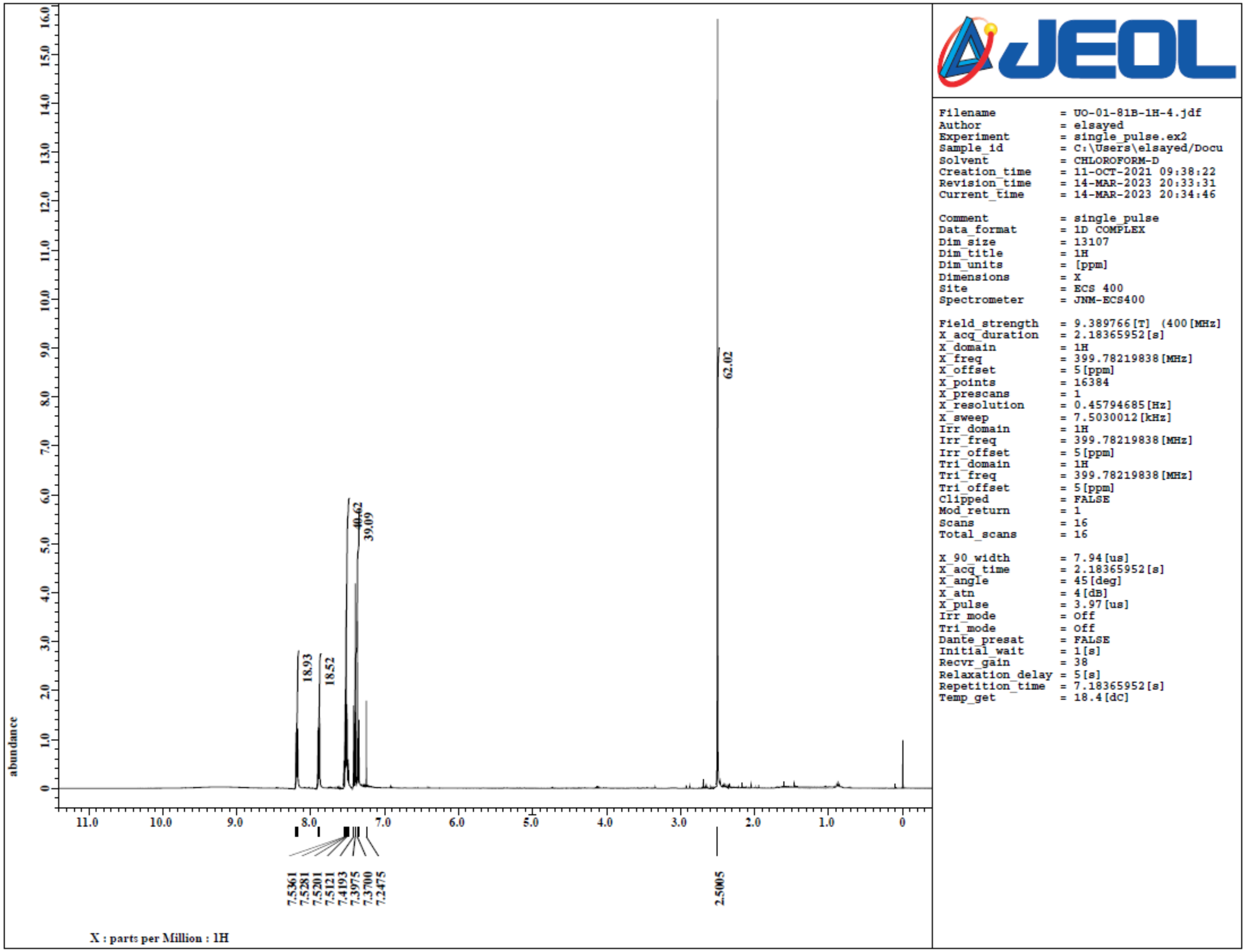

3-methyl-2H-benzo[g]indazole (P6): ^{1}H-NMR ($CDCl_3$) _ Expanded Aromatic Region

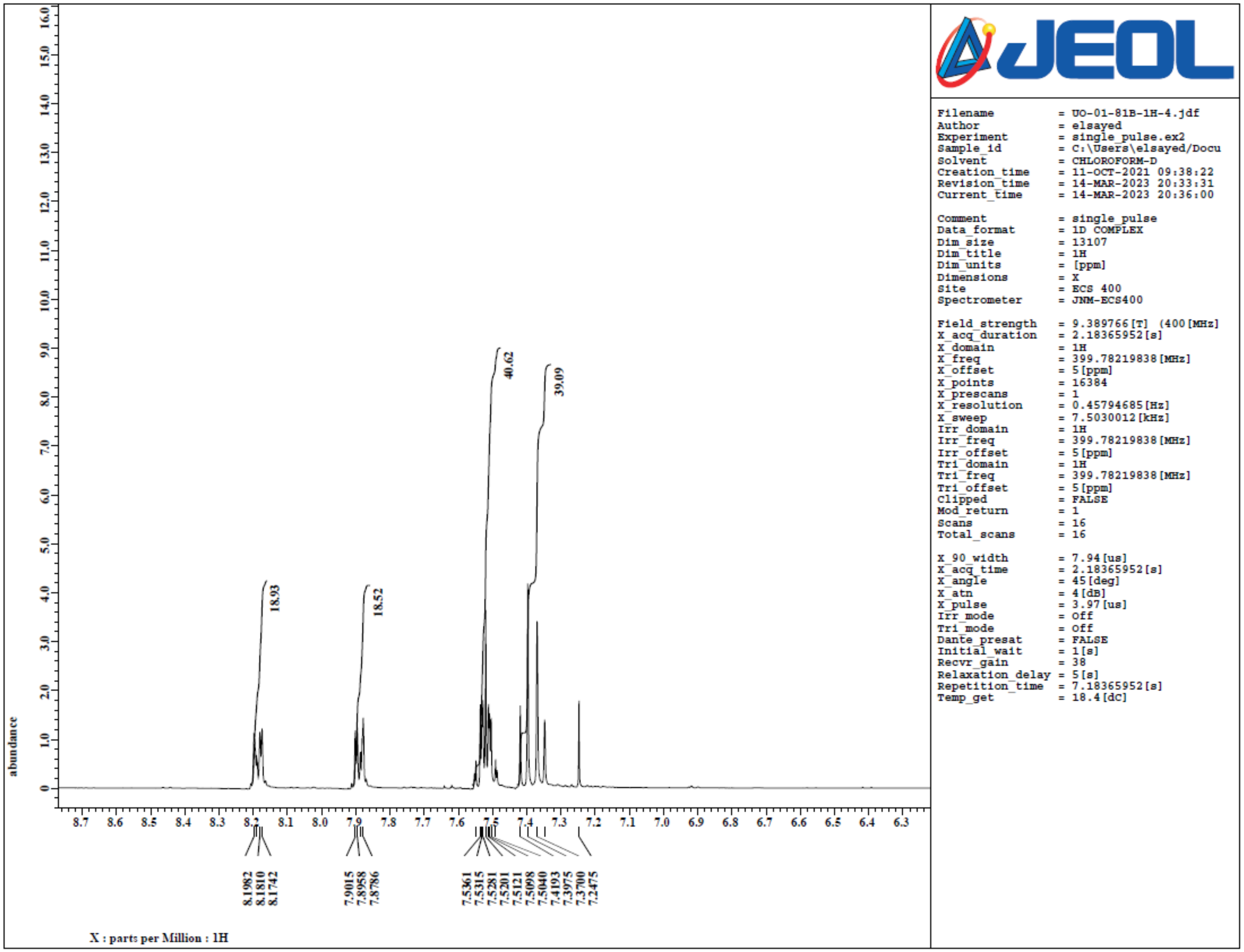

3-methyl-2H-benzo[g]indazole (P6): ^{13}C-NMR ($CDCl_3$)

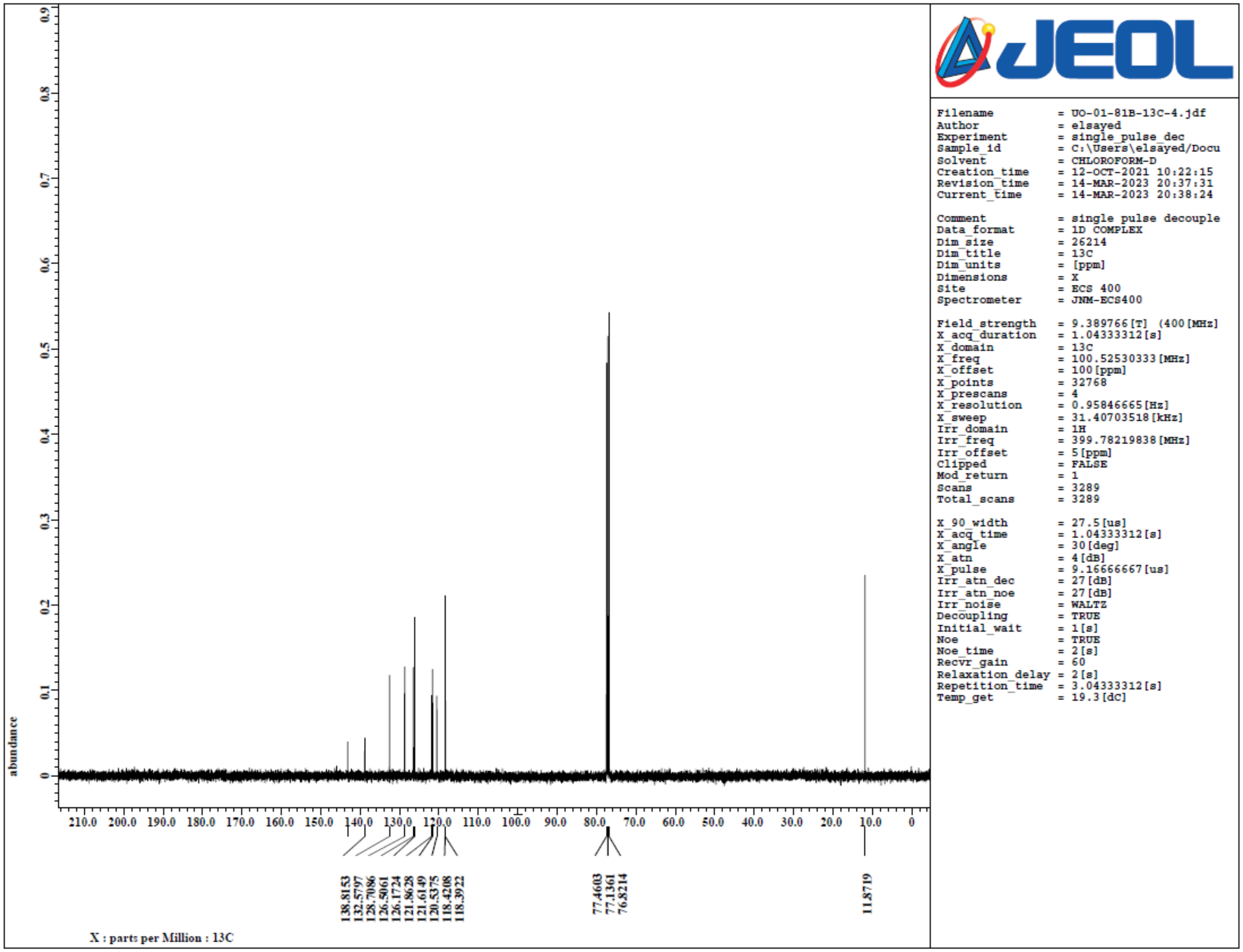

3-methyl-2H-benzo[g]indazole (P6): ^{13}C-NMR ($CDCl_3$) _ Expanded Aromatic Region

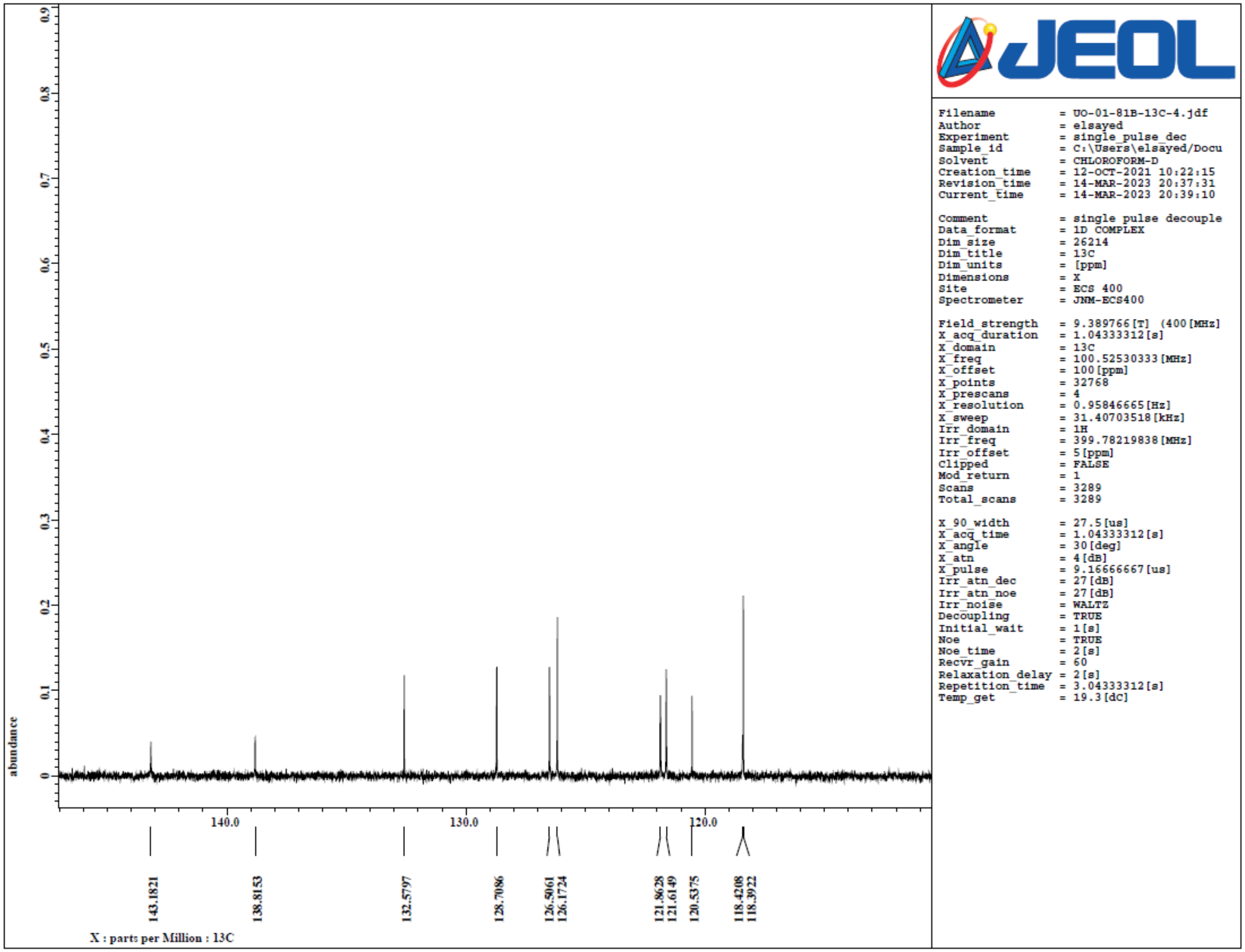

3-methyl-2H-benzo[g]indazole (P6): ESI-MS

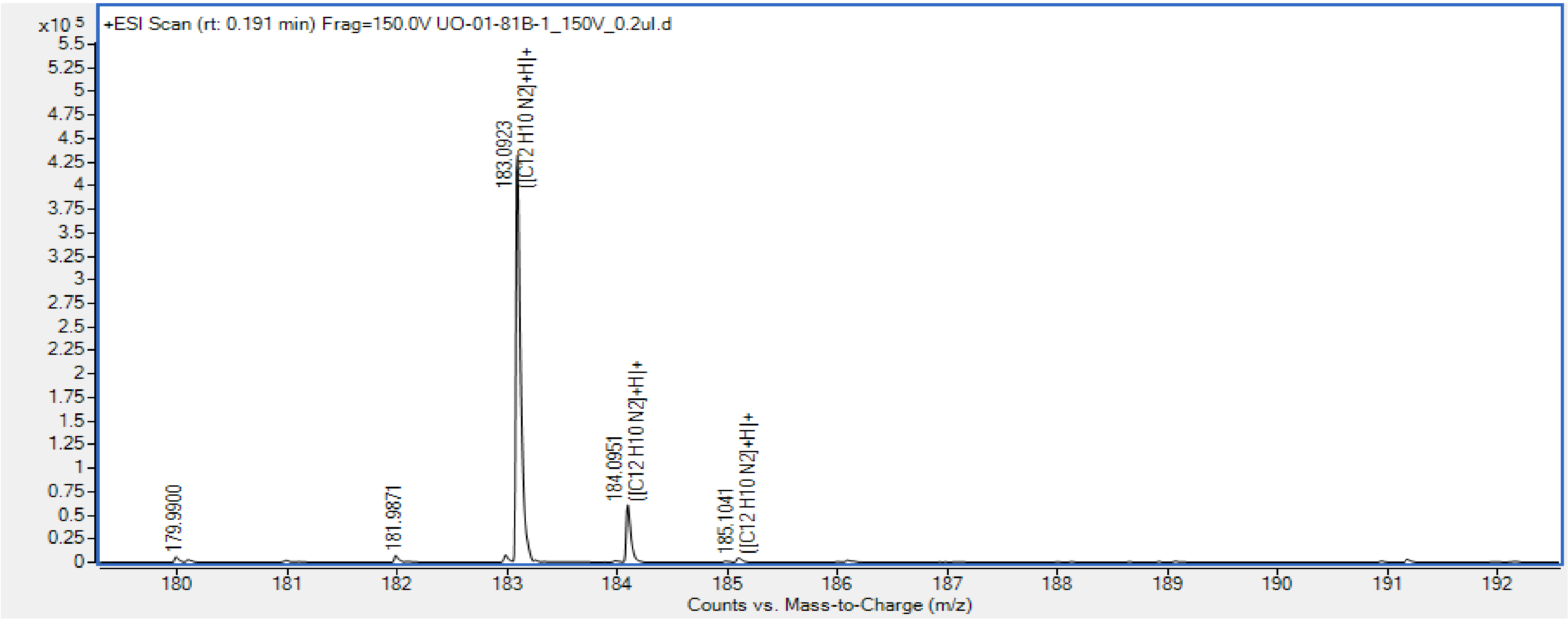

Formula,	Species,	m/z,	Diff (abs. ppm),	Score,	Mass
C12 H10 N2,	(M+H)+,	183.0923,	3.55,	97.29,	182.085

4-ethyl-3,5-dimethyl-1-phenyl-1H-pyrazole (P7): ^{1}H-NMR ($CDCl_3$)

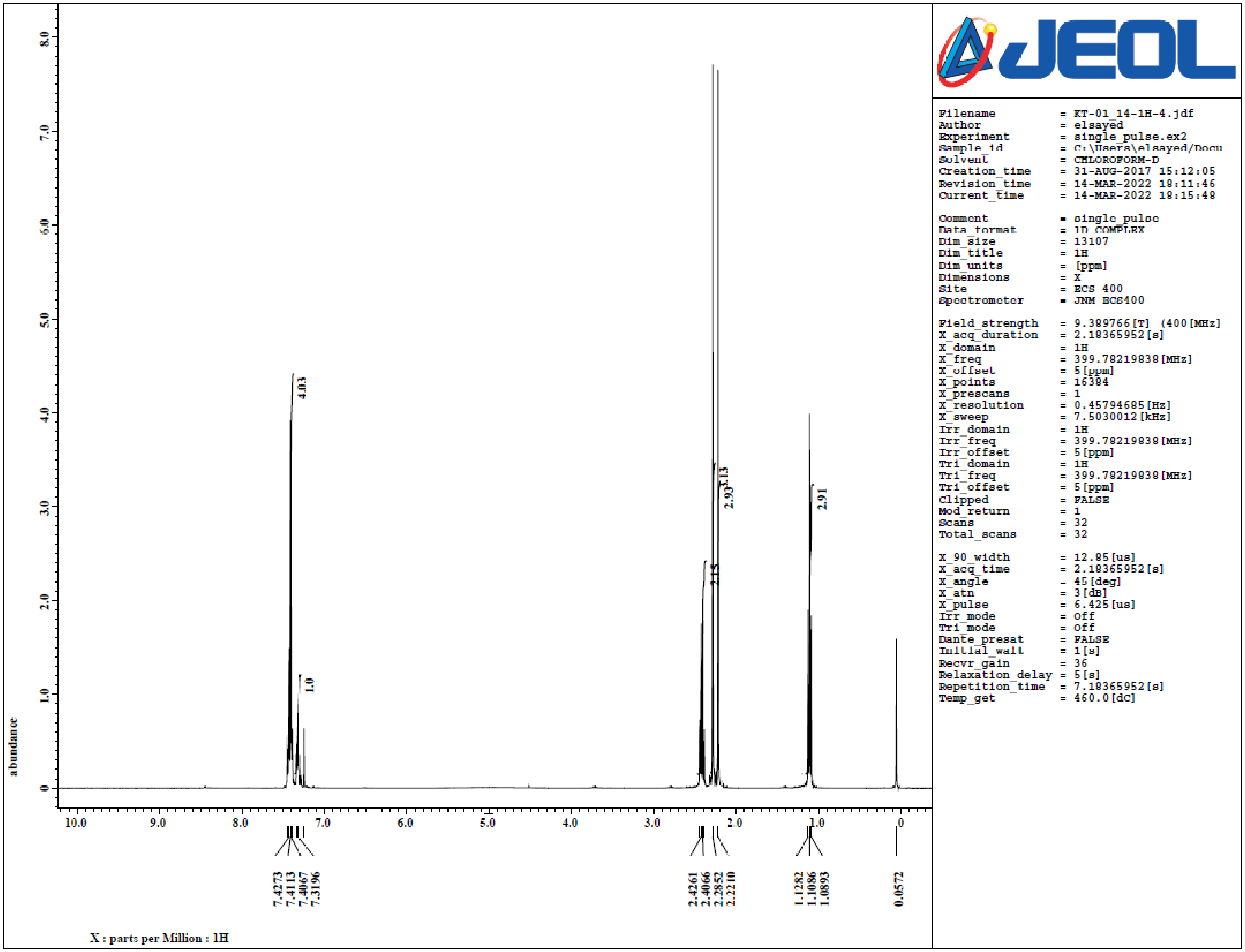

4-ethyl-3,5-dimethyl-1-phenyl-1H-pyrazole (P7): ^{1}H-NMR ($CDCl_3$) _ Expanded Aromatic Region

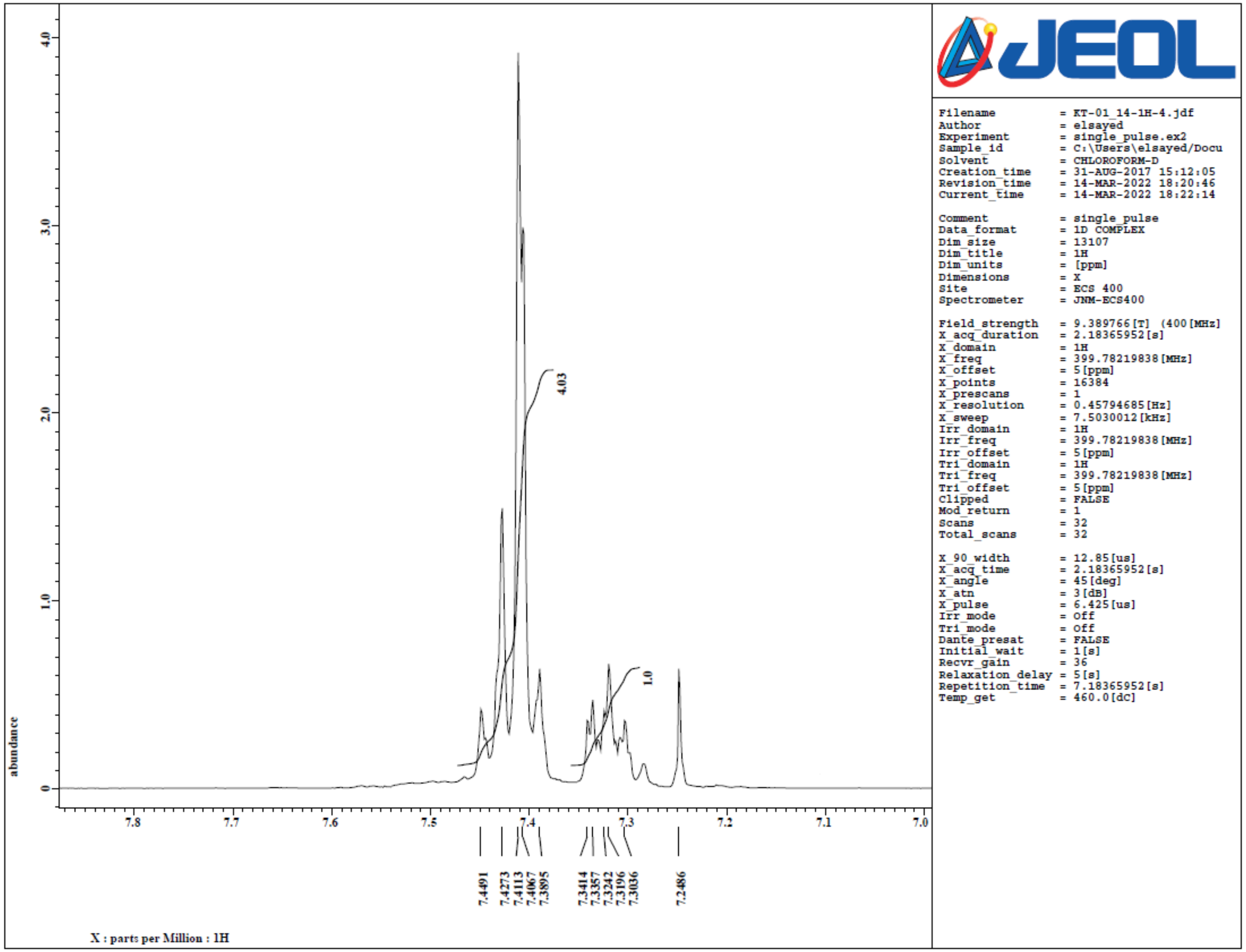


```
Filename           = KT-01_14-1H-4.jdf
Author             = elsayed
Experiment         = single_pulse.ex2
Sample_id          = C:\Users\elsayed/Docu
Solvent            = CHLOROFORM-D
Creation_time      = 31-AUG-2017 15:12:05
Revision_time      = 14-MAR-2022 18:20:46
Current_time       = 14-MAR-2022 18:22:14

Comment            = single_pulse
Data_format        = 1D COMPLEX
Dim_size           = 13107
Dim_title          = 1H
Dim_units          = [ppm]
Dimensions         = X
Site               = ECS 400
Spectrometer       = JNM-ECS400

Field_strength     = 9.389766[T]  (400[MHz]
X_acq_duration     = 2.18365952[s]
X_domain           = 1H
X_freq             = 399.78219838[MHz]
X_offset           = 5[ppm]
X_points           = 16384
X_prescans         = 1
X_resolution       = 0.45794685[Hz]
X_sweep            = 7.5030012[kHz]
Irr_domain         = 1H
Irr_freq           = 399.78219838[MHz]
Irr_offset         = 5[ppm]
Tri_domain         = 1H
Tri_freq           = 399.78219838[MHz]
Tri_offset         = 5[ppm]
Clipped            = FALSE
Mod_return         = 1
Scans              = 32
Total_scans        = 32

X_90_width         = 12.85[us]
X_acq_time         = 2.18365952[s]
X_angle            = 45[deg]
X_atn              = 3[dB]
X_pulse            = 6.425[us]
Irr_mode           = Off
Tri_mode           = Off
Dante_presat       = FALSE
Initial_wait       = 1[s]
Recvr_gain         = 36
Relaxation_delay   = 5[s]
Repetition_time    = 7.18365952[s]
Temp_get           = 460.0[dC]
```

4-ethyl-3,5-dimethyl-1-phenyl-1H-pyrazole (P7): ^{13}C-NMR ($CDCl_3$)

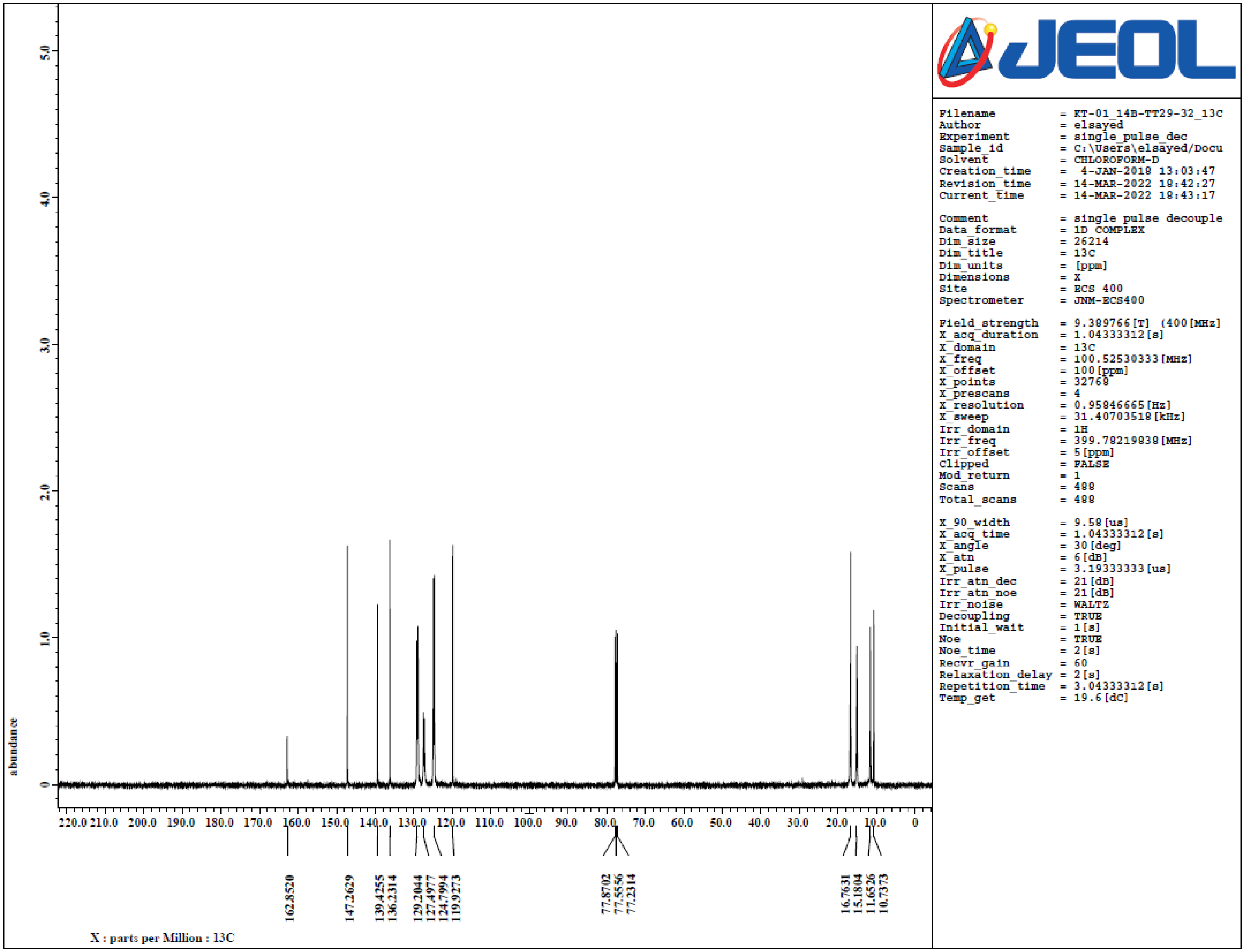

4-ethyl-3,5-dimethyl-1-phenyl-1H-pyrazole (P7): ESI-MS

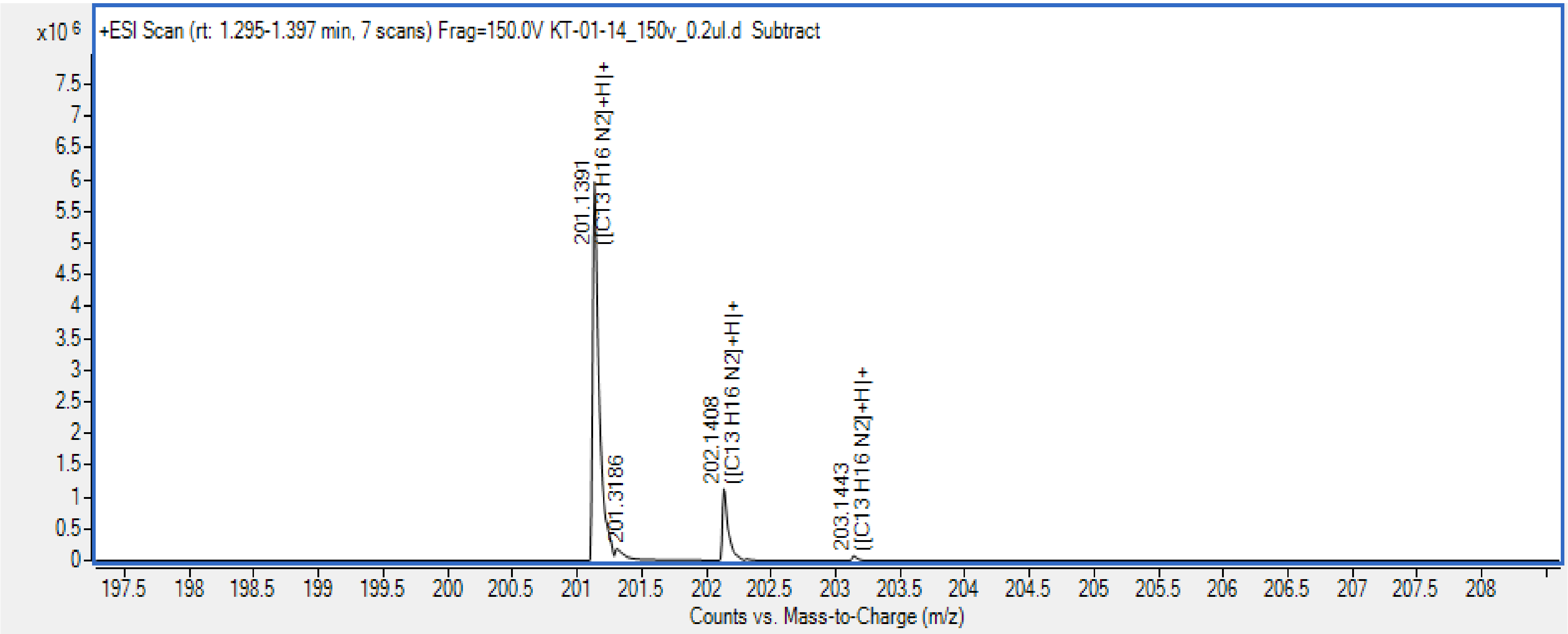

Formula,	Species,	m/z,	Score,	Diff (abs. ppm),	Mass
C13 H16 N2,	(M+H)+,	201.1391,	92.34,	1.02,	200.1316

3-methyl-1-phenyl-1,4,5,6-tetrahydrocyclopenta[c]pyrazole (P8): ^{1}H-NMR ($CDCl_3$)

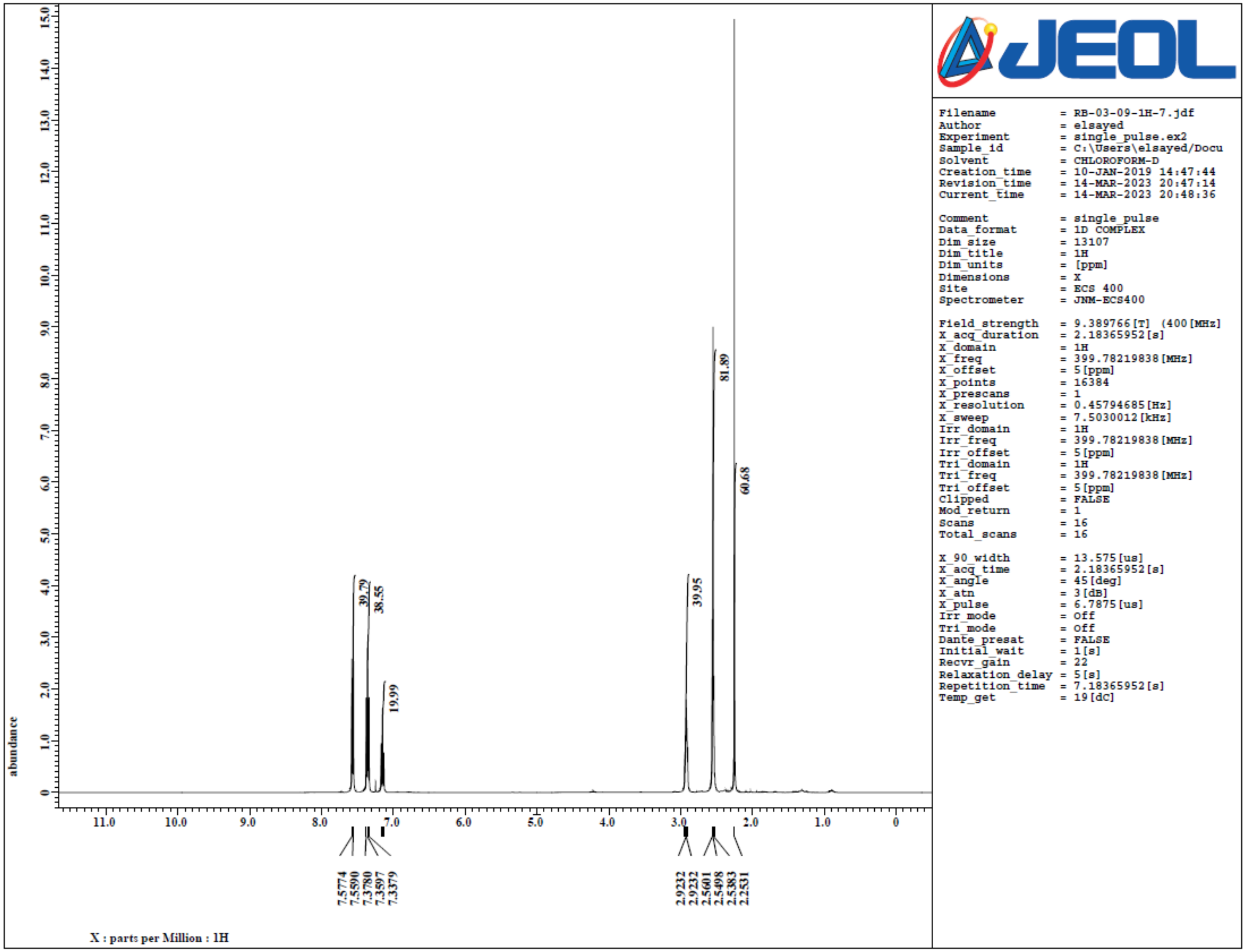

3-methyl-1-phenyl-1,4,5,6-tetrahydrocyclopenta[c]pyrazole (P8): ^{1}H-NMR ($CDCl_3$) _ Expanded Aliphatic Region

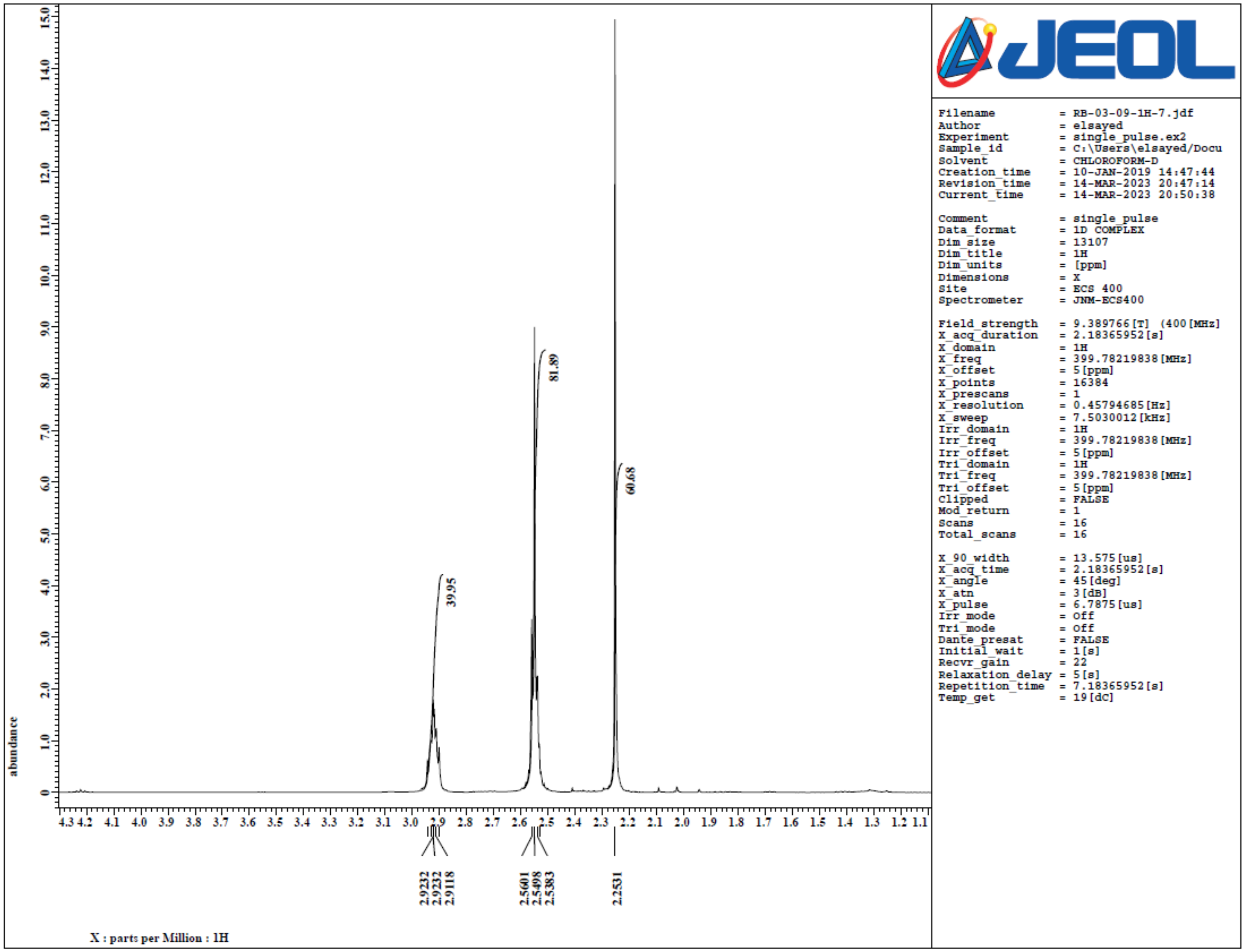

3-methyl-1-phenyl-1,4,5,6-tetrahydrocyclopenta[c]pyrazole (P8): ^{1}H-NMR ($CDCl_3$) _ Expanded Aromatic Region

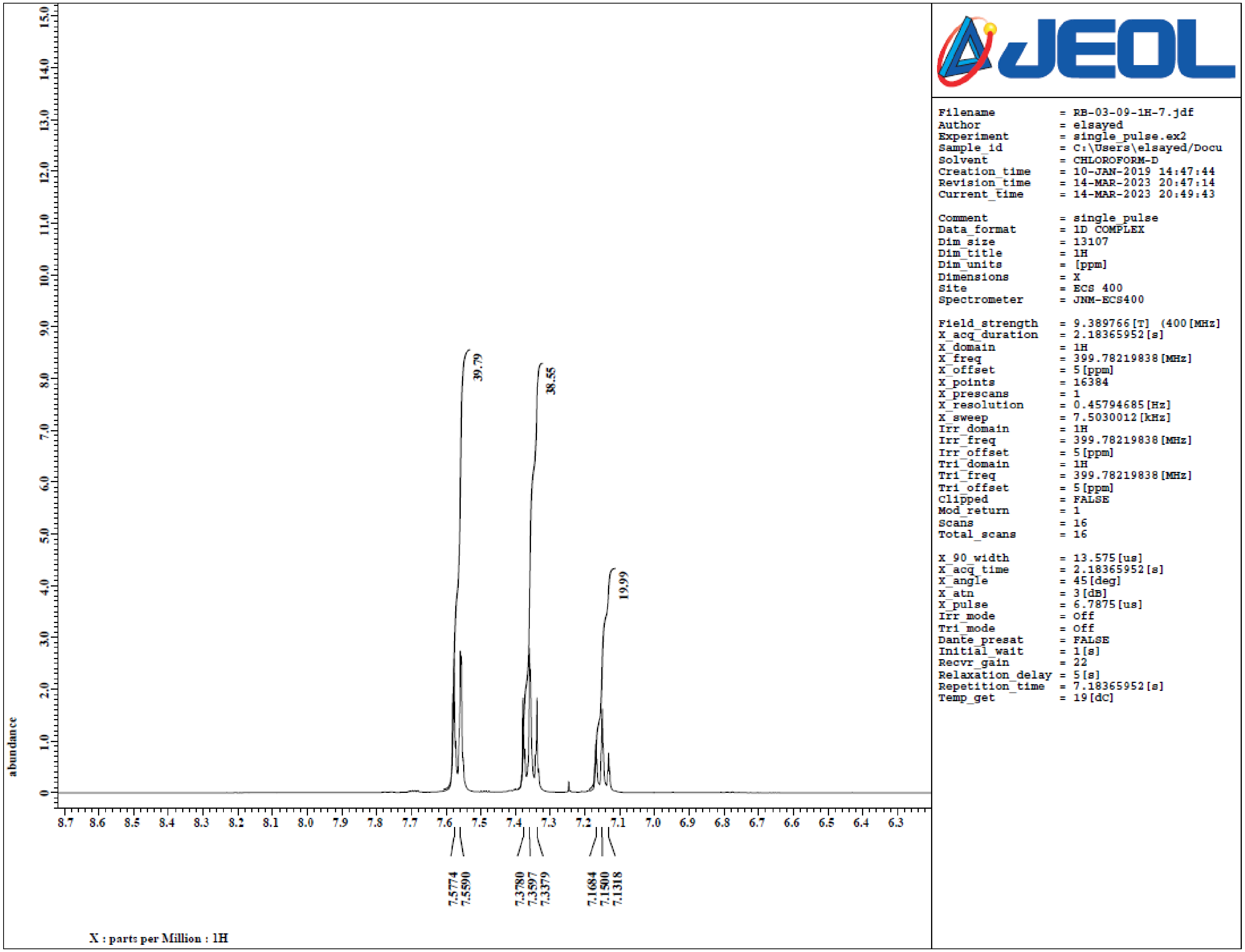

3-methyl-1-phenyl-1,4,5,6-tetrahydrocyclopenta[c]pyrazole (P8): ^{13}C-NMR ($CDCl_3$)

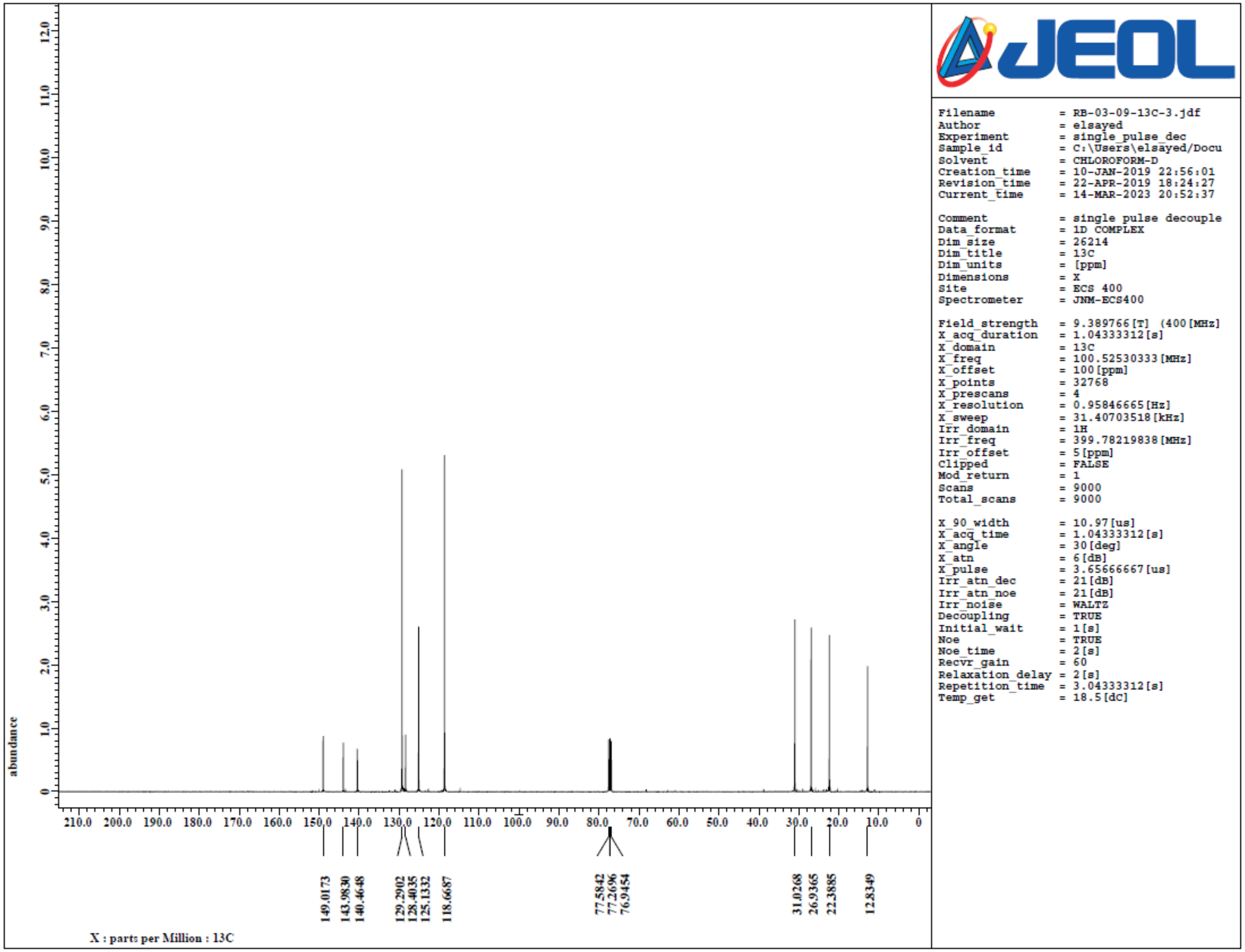

3-methyl-1-phenyl-1,4,5,6-tetrahydrocyclopenta[c]pyrazole (P8): ESI-MS

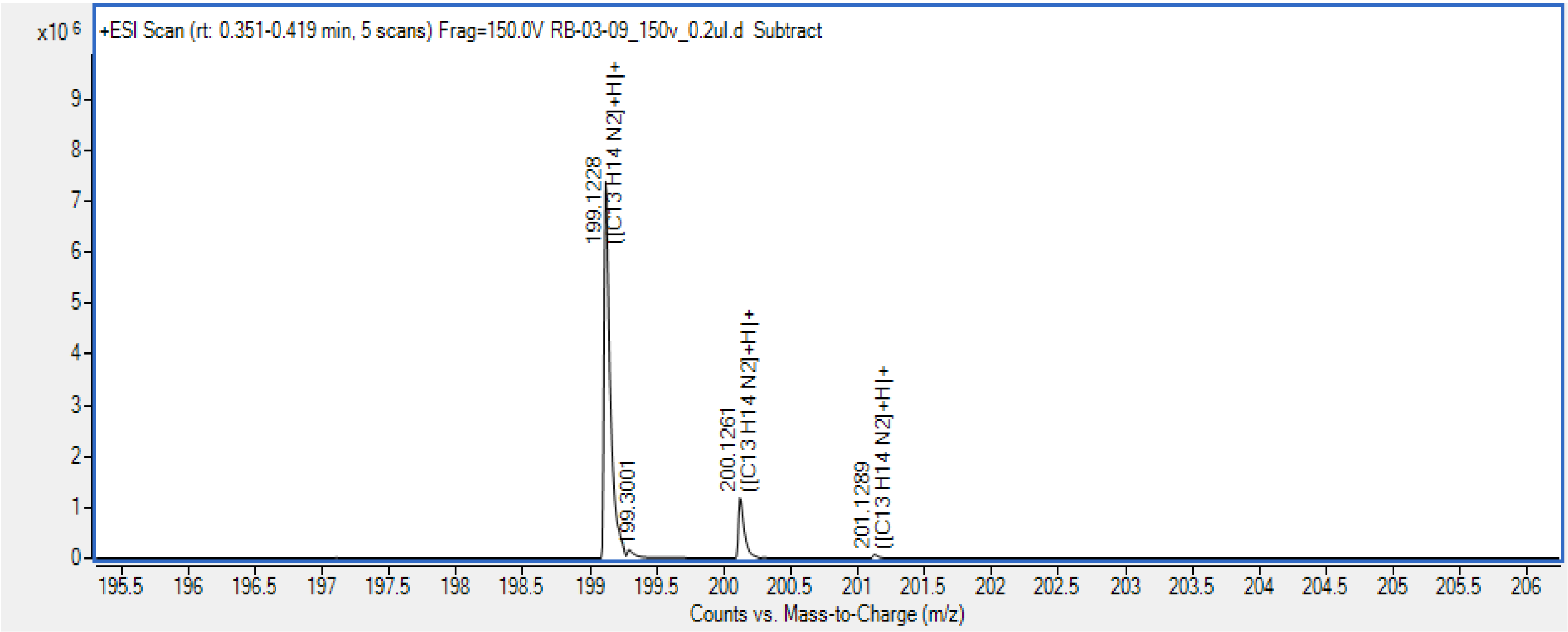

Formula,	Species,	m/z,	Score,	Diff (abs. ppm),	Mass
C13 H14 N2,	(M+H)+,	199.1228,	99.52,	0.57,	198.1156

3-methyl-2-phenyl-4,5-dihydro-2H-benzo[g]indazole (P9): ^{1}H-NMR ($CDCl_3$)

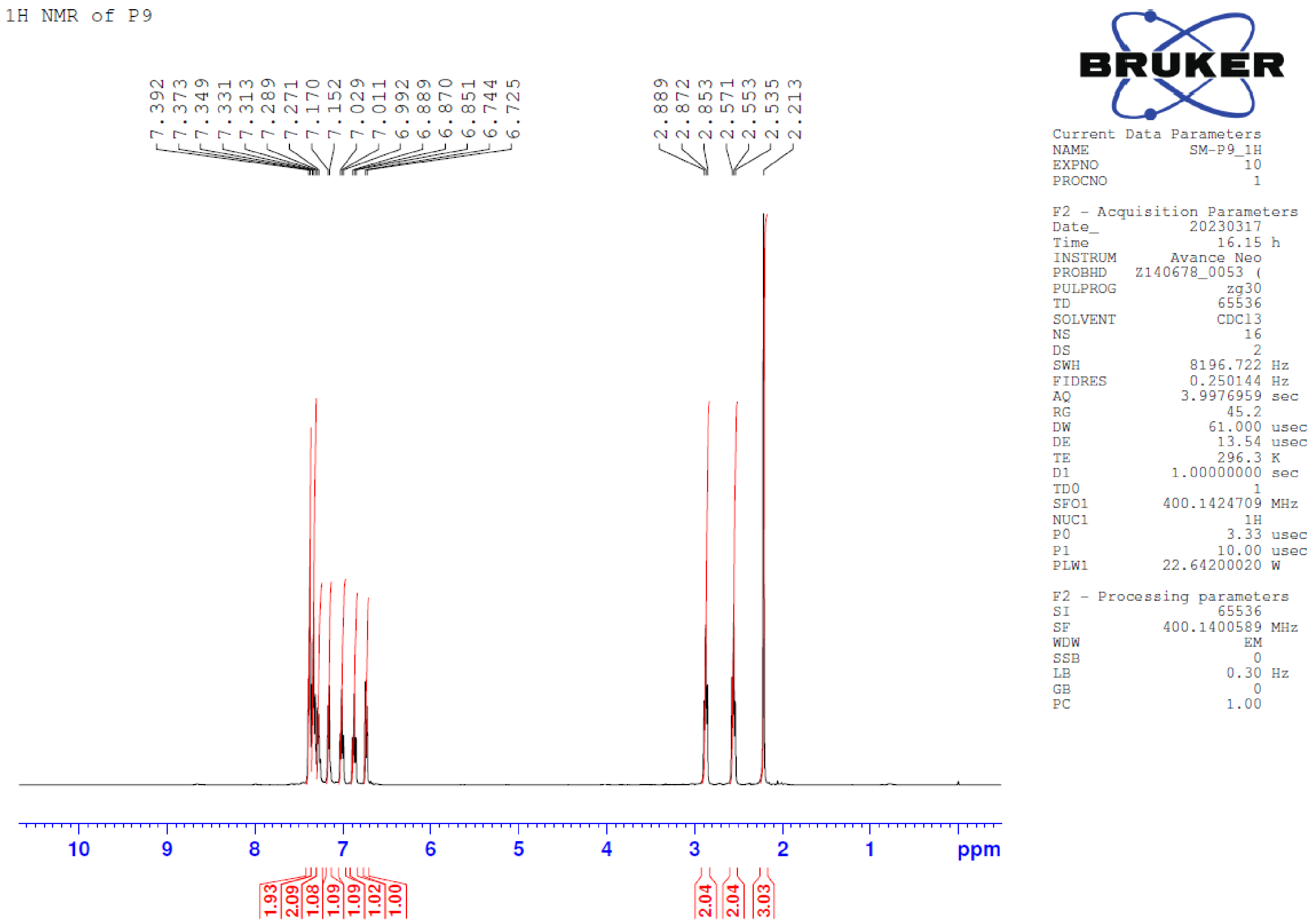

3-methyl-2-phenyl-4,5-dihydro-2H-benzo[g]indazole (P9): ^{1}H-NMR ($CDCl_3$) _ Expanded Aromatic Region

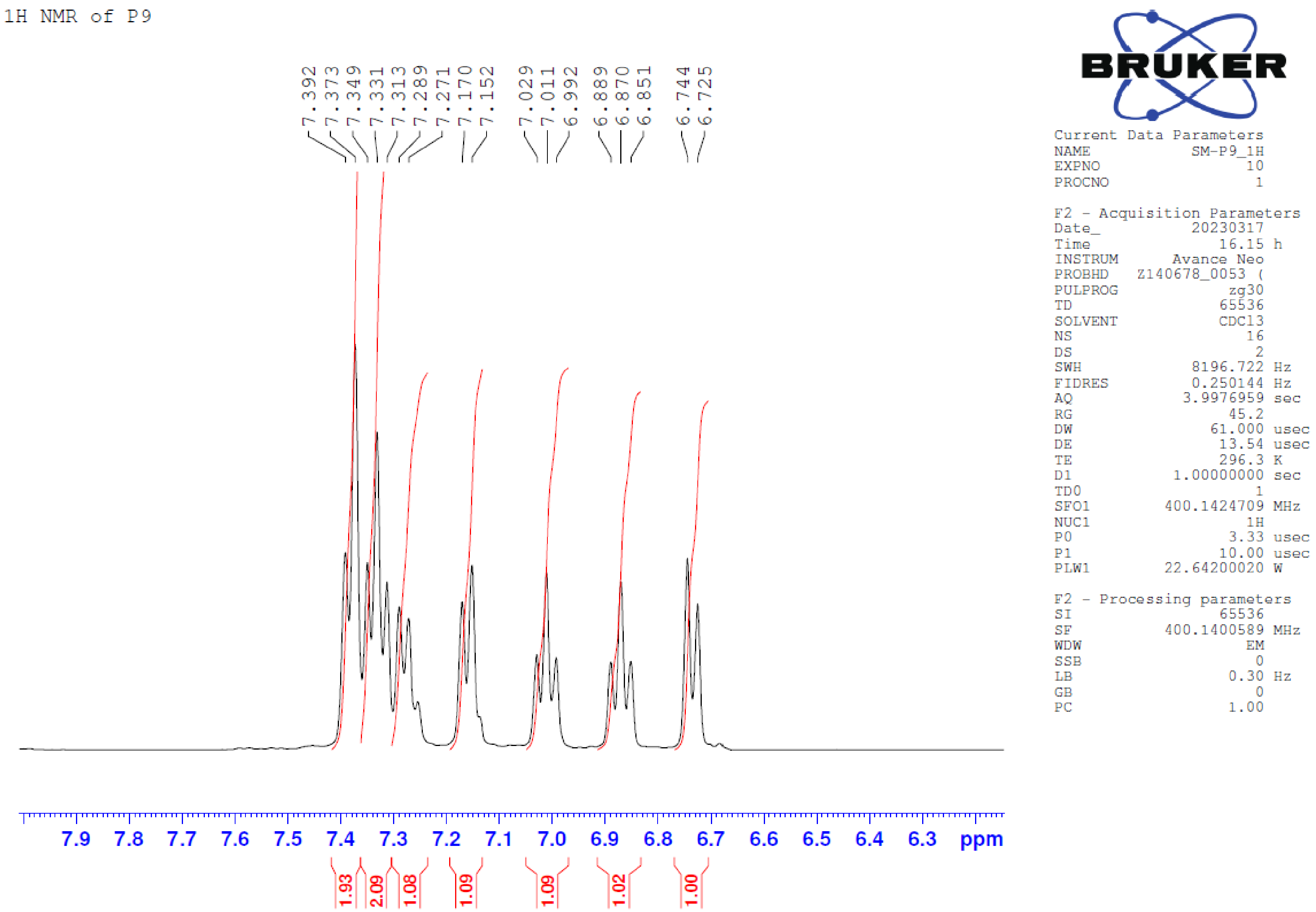

3-methyl-2-phenyl-4,5-dihydro-2H-benzo[g]indazole (P9): ^{13}C-NMR ($CDCl_3$)

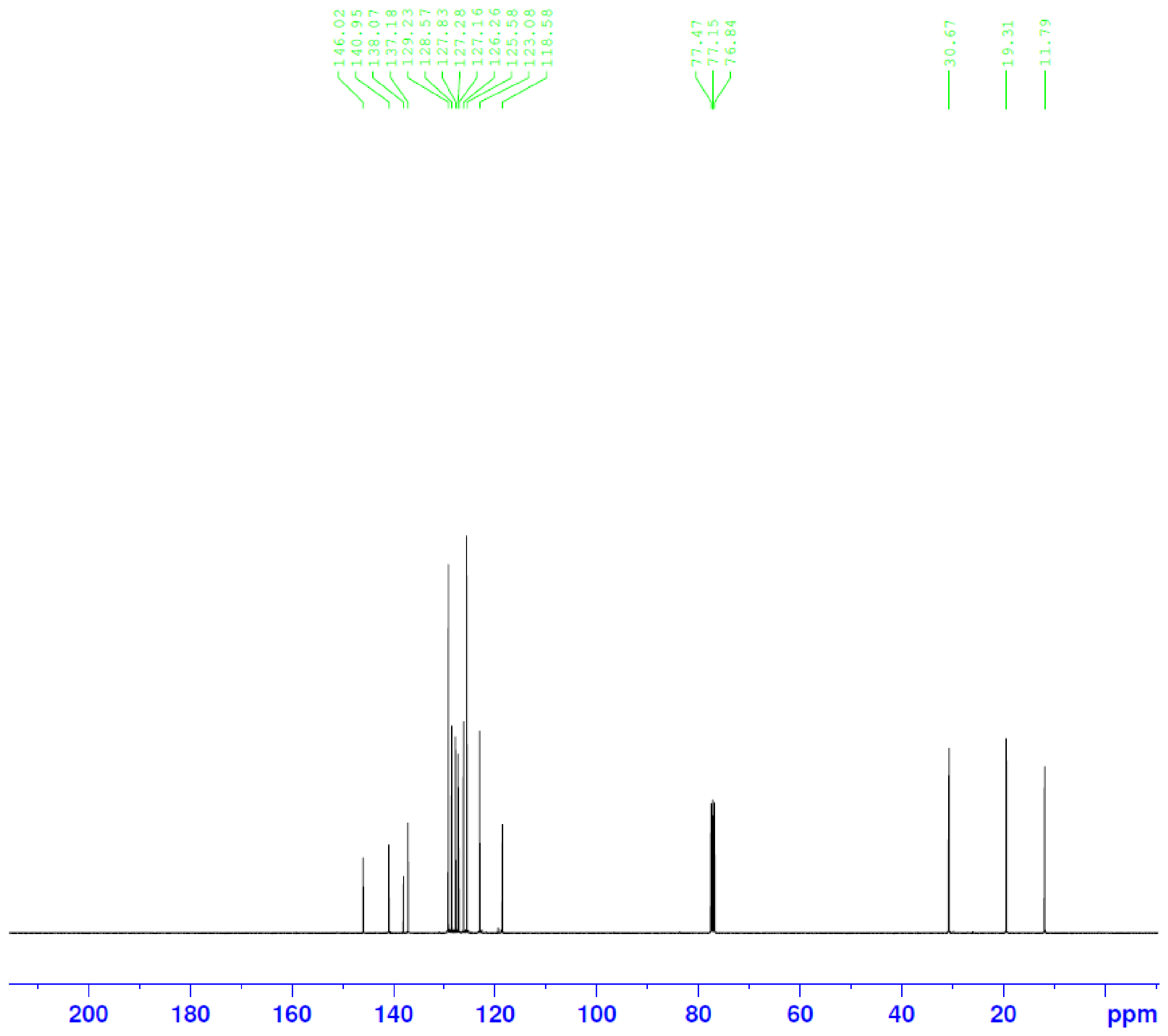


```
Current Data Parameters
NAME        SM-P-9_1Hand13C
EXPNO                    11
PROCNO                    1

F2 - Acquisition Parameters
Date_              20230318
Time                   1.22 h
INSTRUM          Avance Neo
PROBHD       Z140678_0053 (
PULPROG             zgpg30
TD                   65536
SOLVENT              CDCl3
NS                    4096
DS                       4
SWH              23809.523 Hz
FIDRES            0.726609 Hz
AQ               1.3762560 sec
RG                      16
DW                  21.000 usec
DE                    6.50 usec
TE                   296.6 K
D1              2.00000000 sec
D11             0.03000000 sec
TD0                      1
SFO1           100.6253446 MHz
NUC1                   13C
P0                    3.33 usec
P1                   10.00 usec
PLW1           77.80000305 W
SFO2           400.1416006 MHz
NUC2                    1H
CPDPRG[2           waltz65
PCPD2                90.00 usec
PLW2           22.64200020 W
PLW12           0.27952999 W
PLW13           0.14060000 W

F2 - Processing parameters
SI                   32768
SF             100.6152830 MHz
WDW                     EM
SSB                      0
LB                    1.00 Hz
GB                       0
PC                    1.40
```

3-methyl-2-phenyl-4,5-dihydro-2H-benzo[g]indazole (P9): ^{13}C-NMR ($CDCl_3$) _ Expanded Aromatic Region

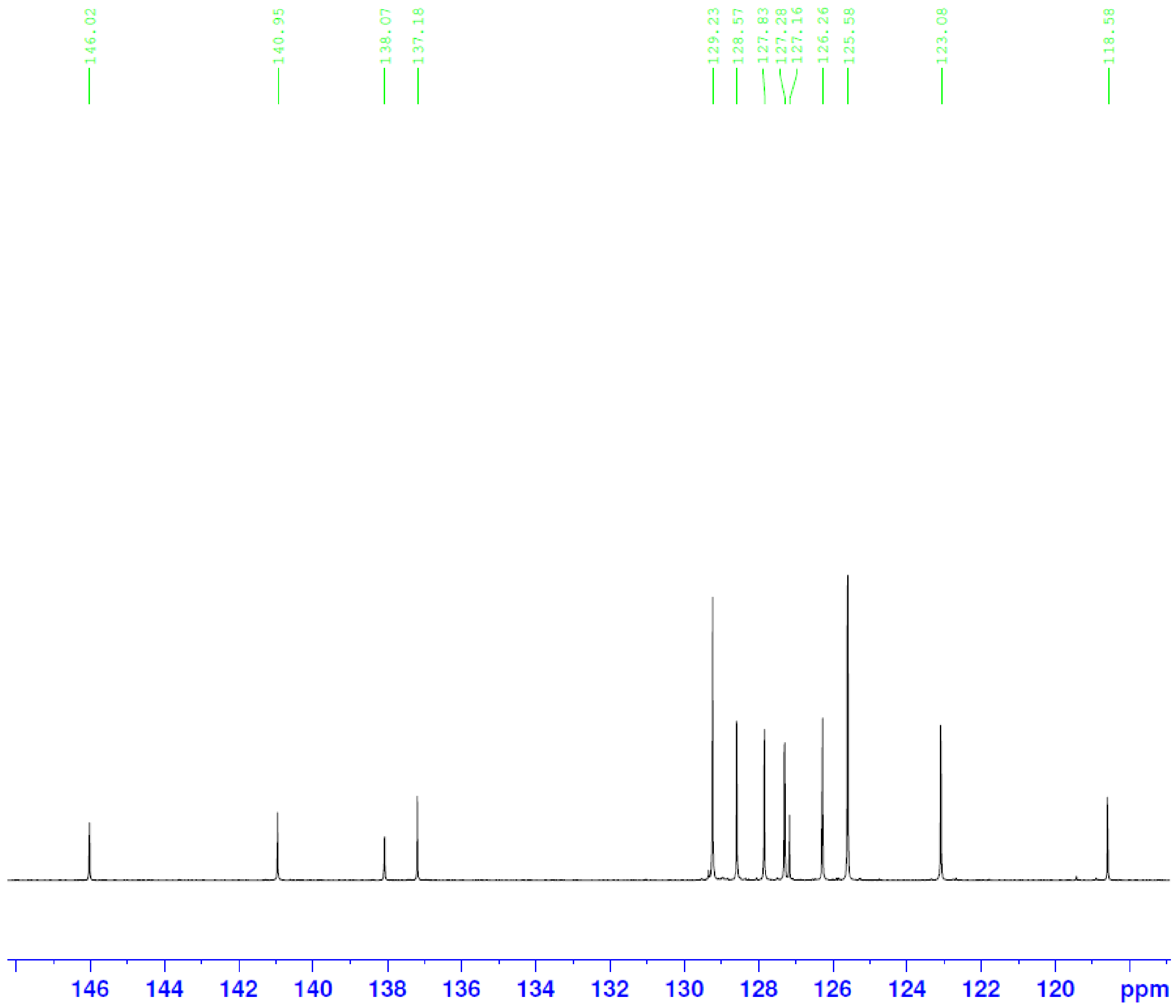


```
Current Data Parameters
NAME       SM-P-9_1Hand13C
EXPNO                   11
PROCNO                   1

F2 - Acquisition Parameters
Date_             20230318
Time                  1.22 h
INSTRUM          Avance Neo
PROBHD       Z140678_0053 (
PULPROG             zgpg30
TD                   65536
SOLVENT              CDCl3
NS                    4096
DS                       4
SWH              23809.523 Hz
FIDRES            0.726609 Hz
AQ               1.3762560 sec
RG                      16
DW                  21.000 usec
DE                    6.50 usec
TE                   296.6 K
D1              2.00000000 sec
D11             0.03000000 sec
TD0                      1
SFO1           100.6253446 MHz
NUC1                   13C
P0                    3.33 usec
P1                   10.00 usec
PLW1           77.80000305 W
SFO2           400.1416006 MHz
NUC2                    1H
CPDPRG[2           waltz65
PCPD2                90.00 usec
PLW2           22.64200020 W
PLW12           0.27952999 W
PLW13           0.14060000 W

F2 - Processing parameters
SI                   32768
SF             100.6152830 MHz
WDW                     EM
SSB                      0
LB                    1.00 Hz
GB                       0
PC                    1.40
```

3-methyl-2-phenyl-4,5-dihydro-2H-benzo[g]indazole (P9): ESI-MS

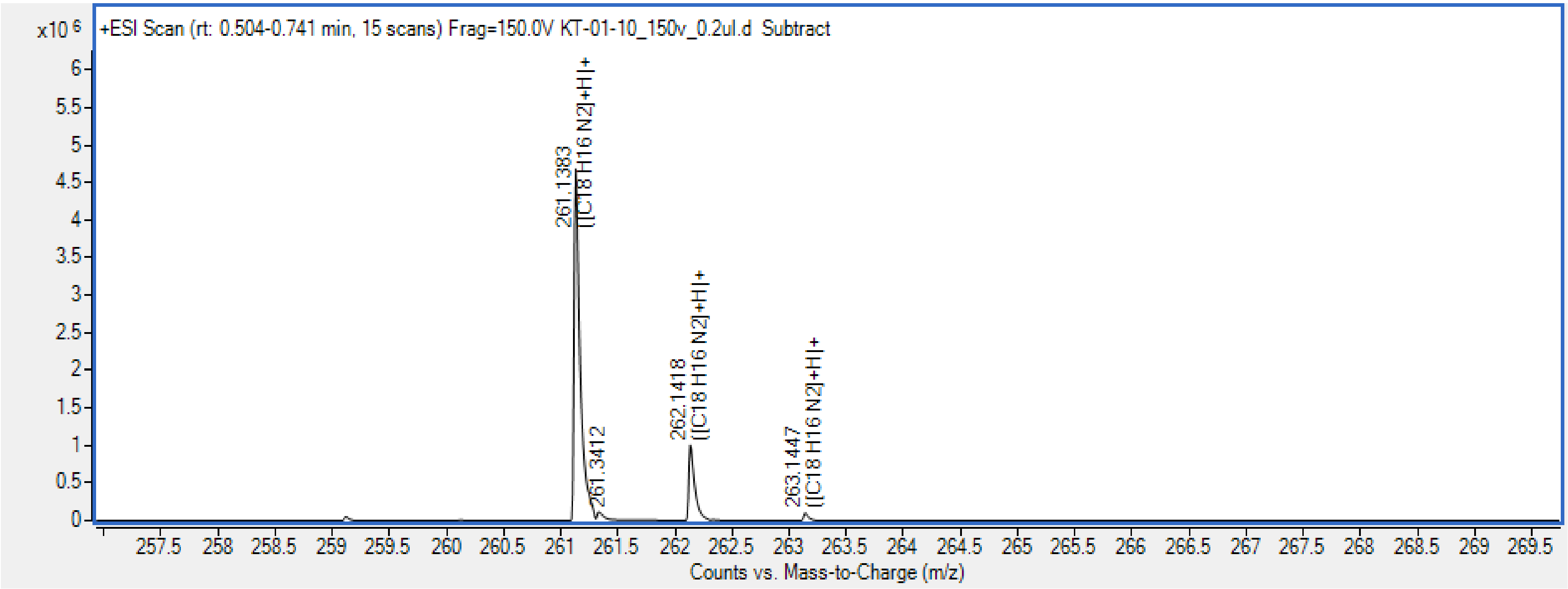

Formula,	Species,	m/z,	Score,	Diff (abs. ppm),	Mass
C18 H16 N2,	(M+H)+,	261.1383,	98.93,	1.11,	260.1311

Ethyl 3,5-dimethyl-1-phenyl-1H-pyrazole-4-carboxylate (P10): ^{1}H-NMR ($CDCl_3$)

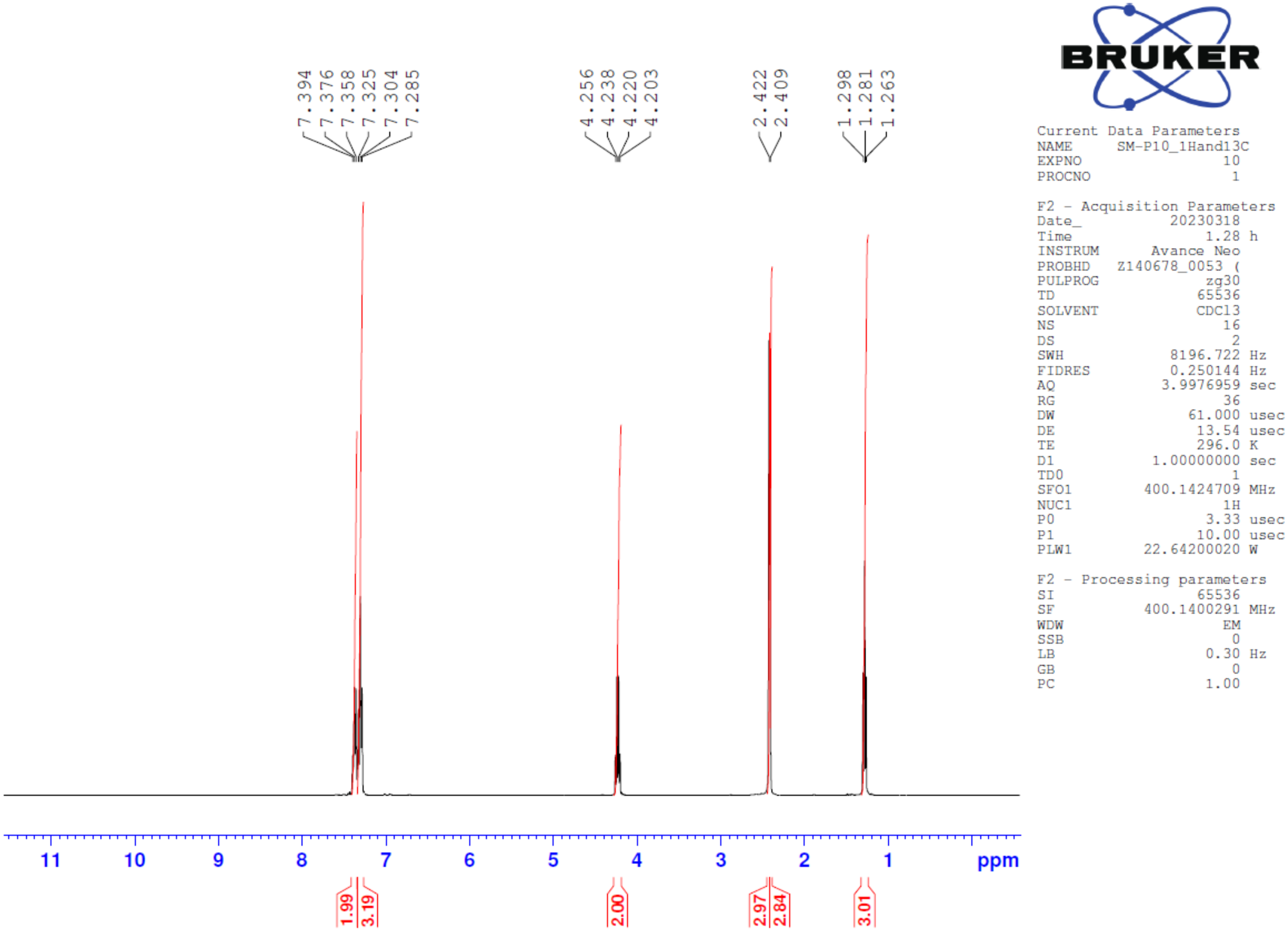

Ethyl 3,5-dimethyl-1-phenyl-1H-pyrazole-4-carboxylate (P10): ^{1}H-NMR ($CDCl_3$) _ Expanded Aromatic Region

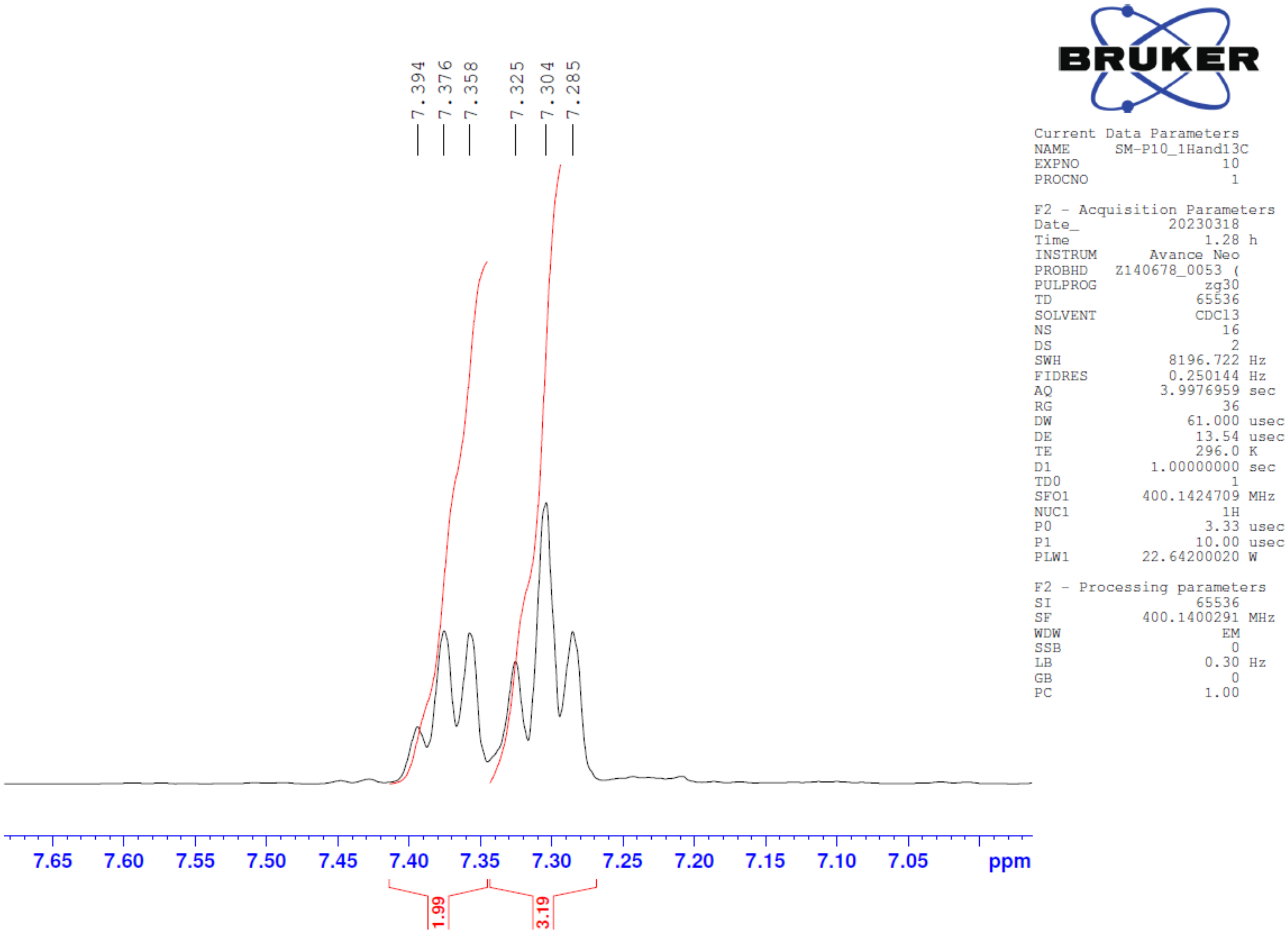

Ethyl 3,5-dimethyl-1-phenyl-1H-pyrazole-4-carboxylate (P10): ^{13}C-NMR ($CDCl_3$)

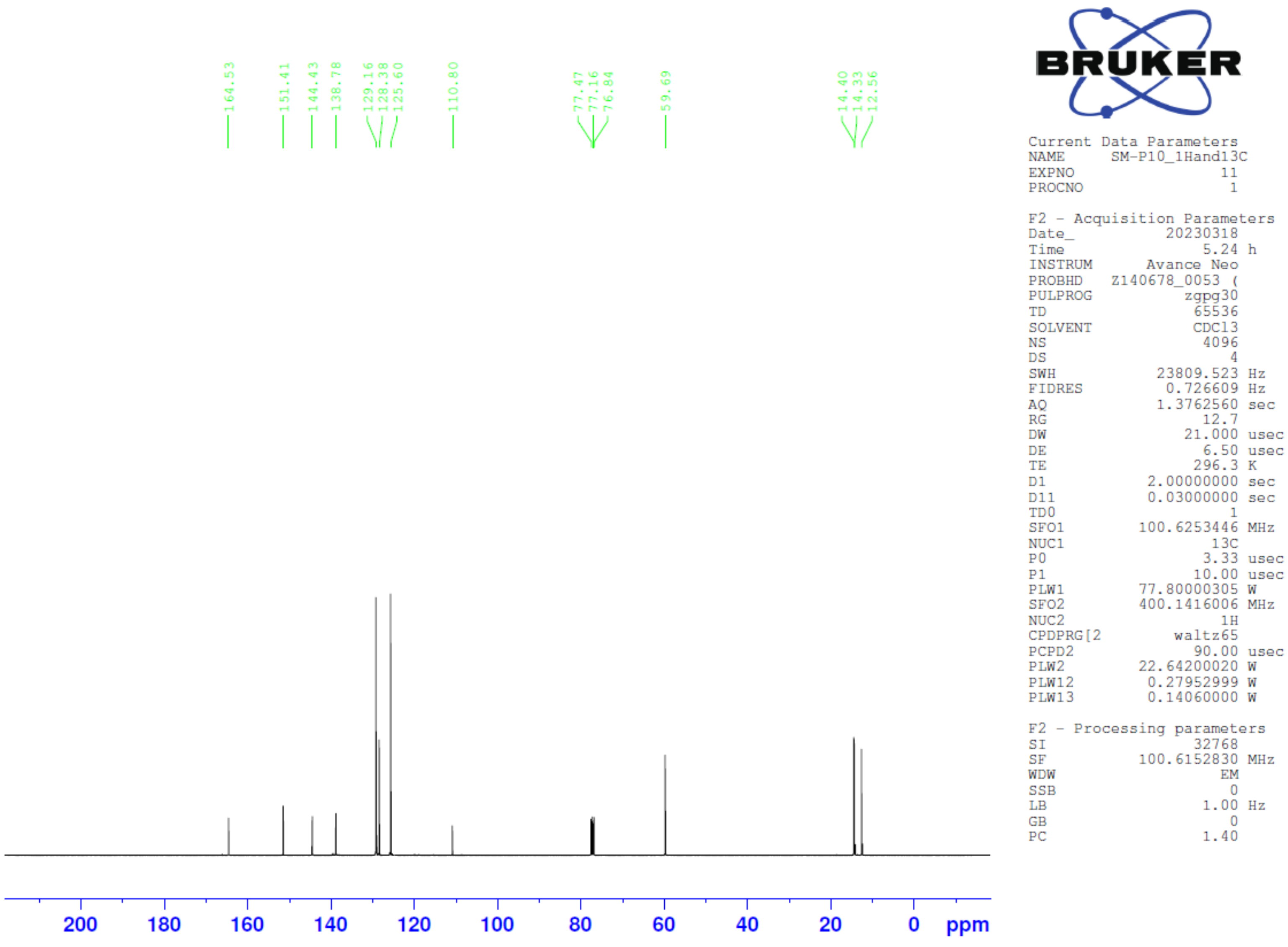

Ethyl 3,5-dimethyl-1-phenyl-1H-pyrazole-4-carboxylate (P10): ^{13}C-NMR ($CDCl_3$) _ Expanded Aliphatic Region

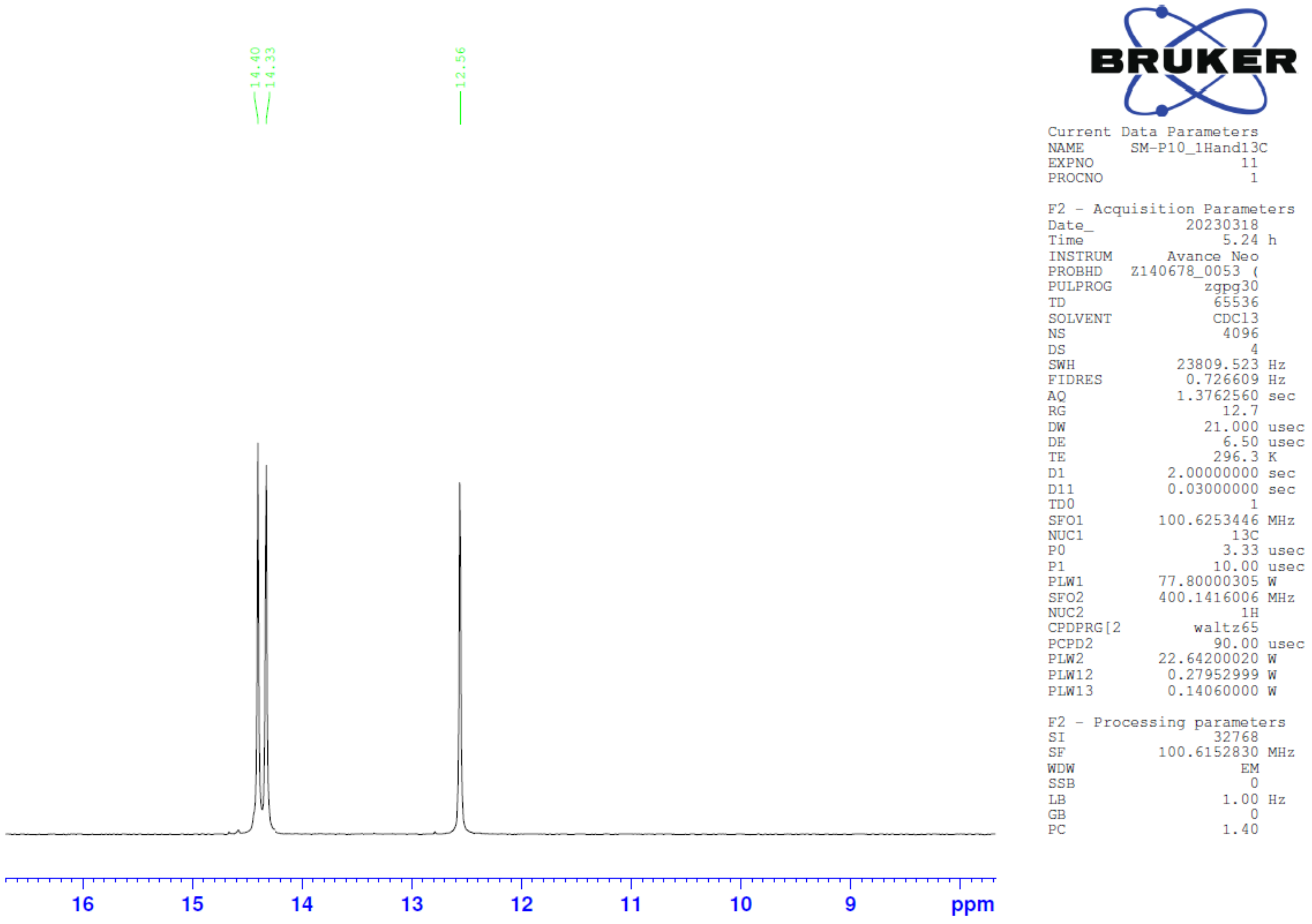

Ethyl 3,5-dimethyl-1-phenyl-1H-pyrazole-4-carboxylate (P10): ESI-MS

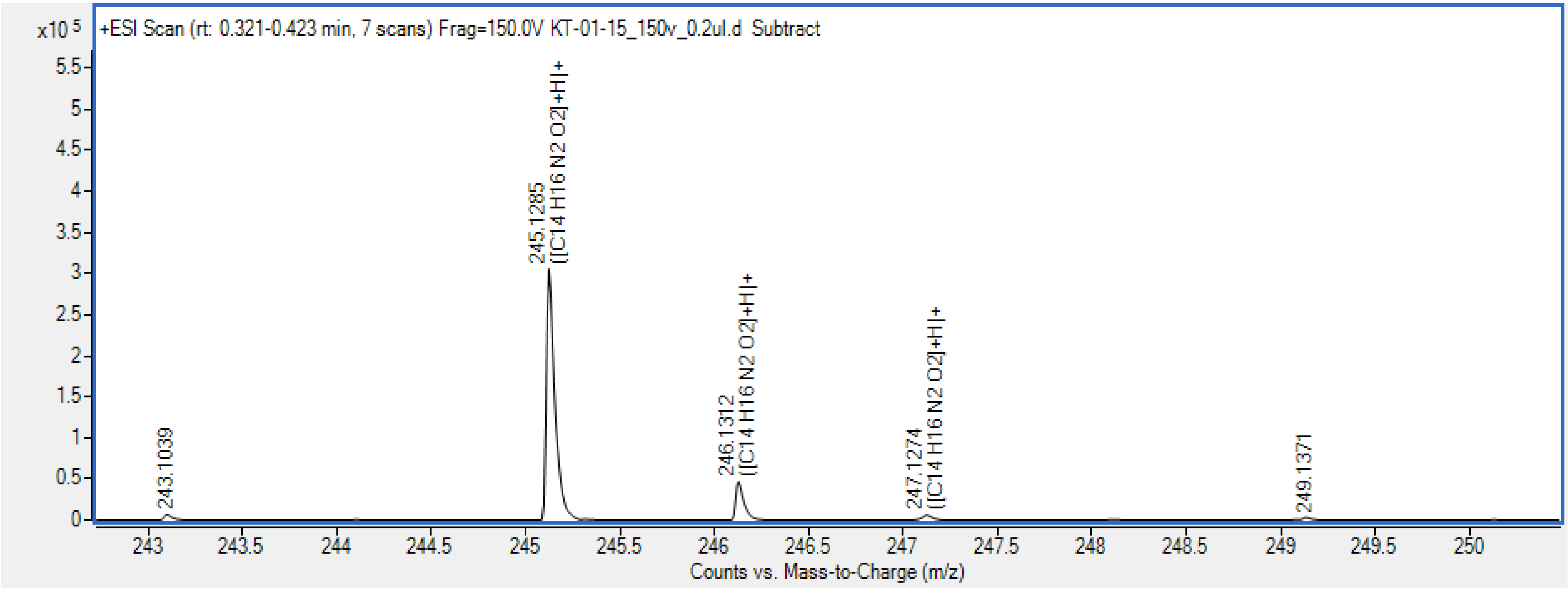

Formula,	Species,	m/z,	Score,	Diff (abs. ppm),	Mass
C14 H16 N2 O2,	(M+H)+,	245.1285,	97.86,	0.62,	244.121

5-methyl-3-phenylisoxazole (P11): ^{1}H-NMR ($CDCl_3$)

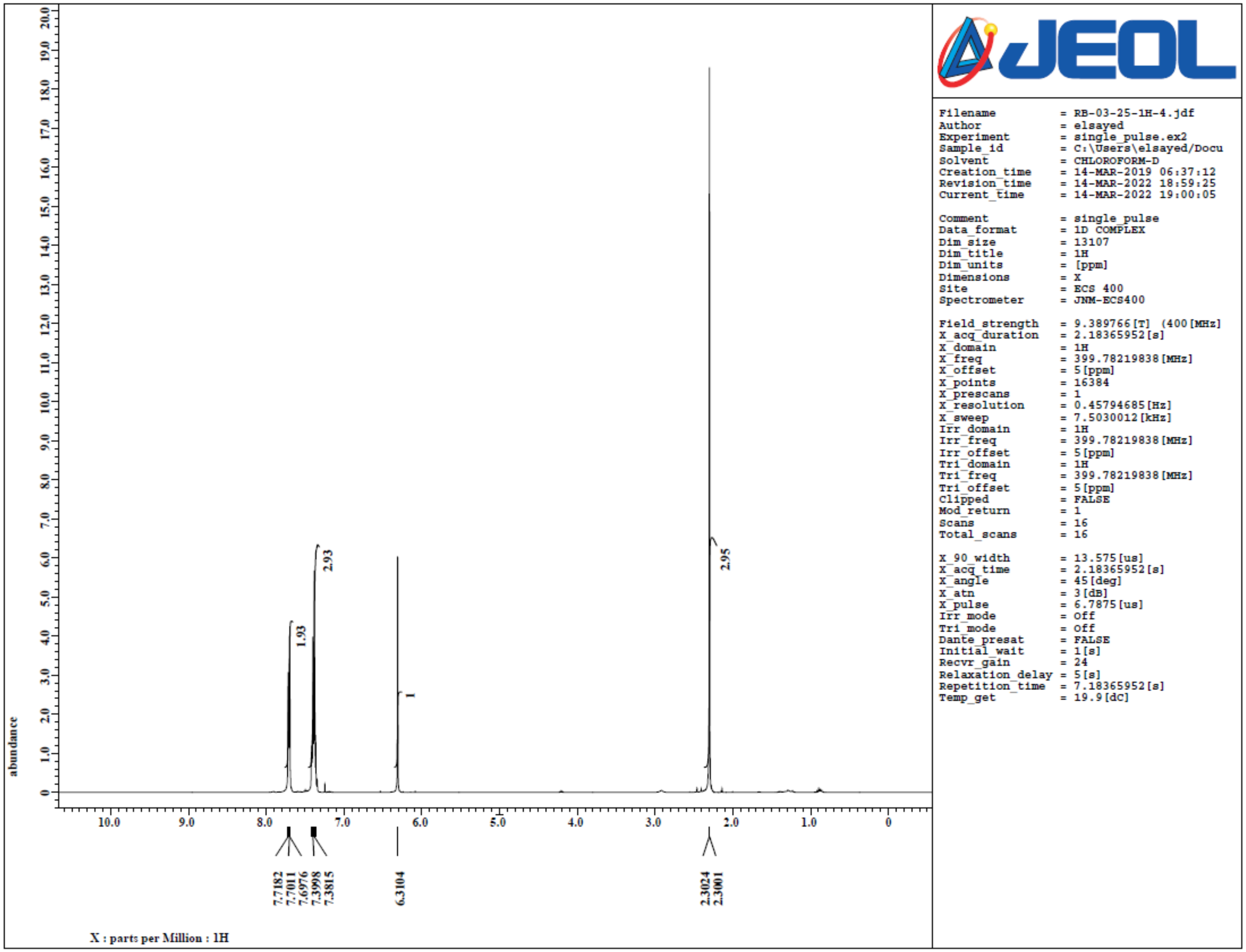

5-methyl-3-phenylisoxazole (P11): ^{1}H-NMR ($CDCl_3$) _ Aromatic Region Expanded

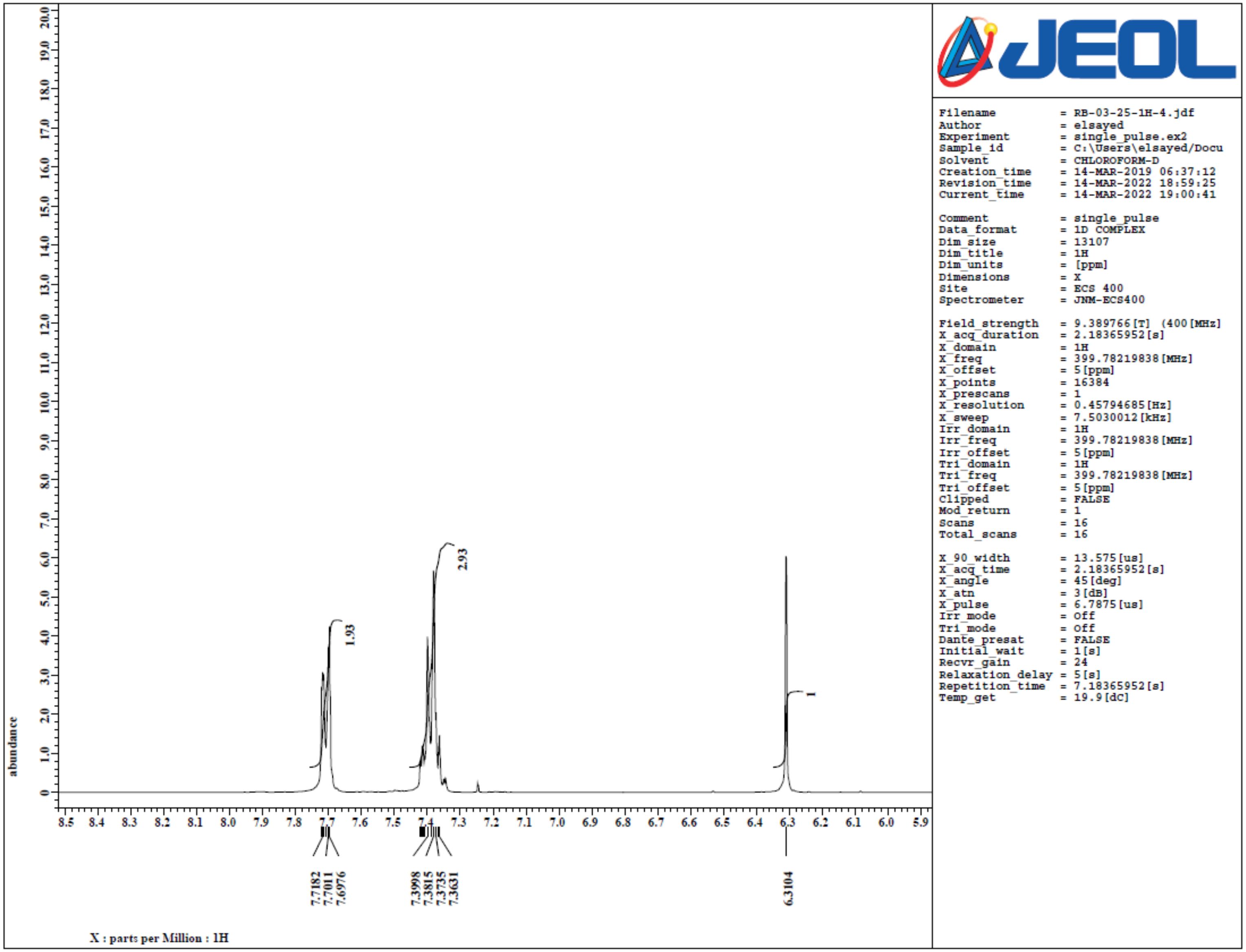

5-methyl-3-phenylisoxazole (P11): ^{13}C-NMR ($CDCl_3$)

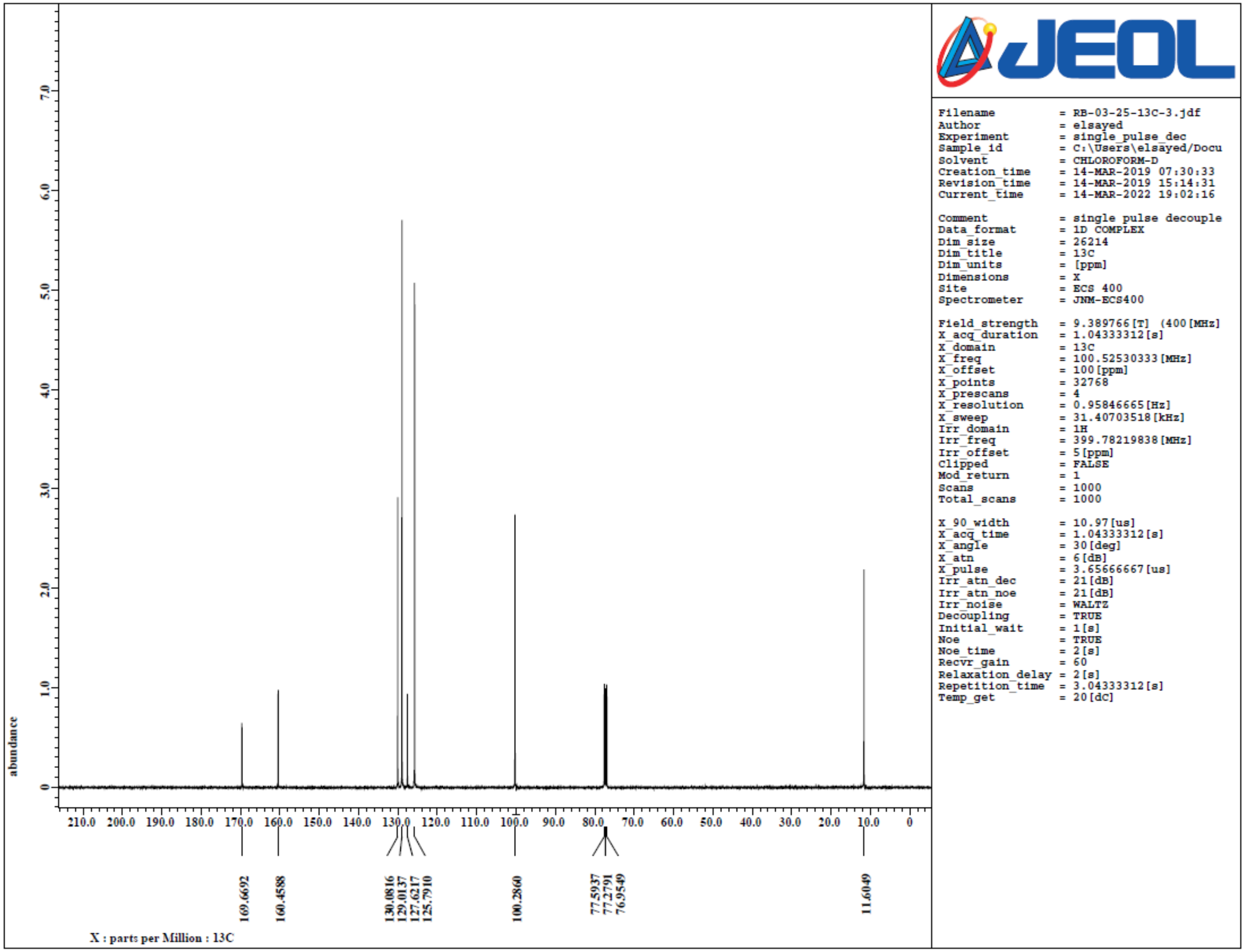

5-methyl-3-phenylisoxazole (P11): ESI-MS

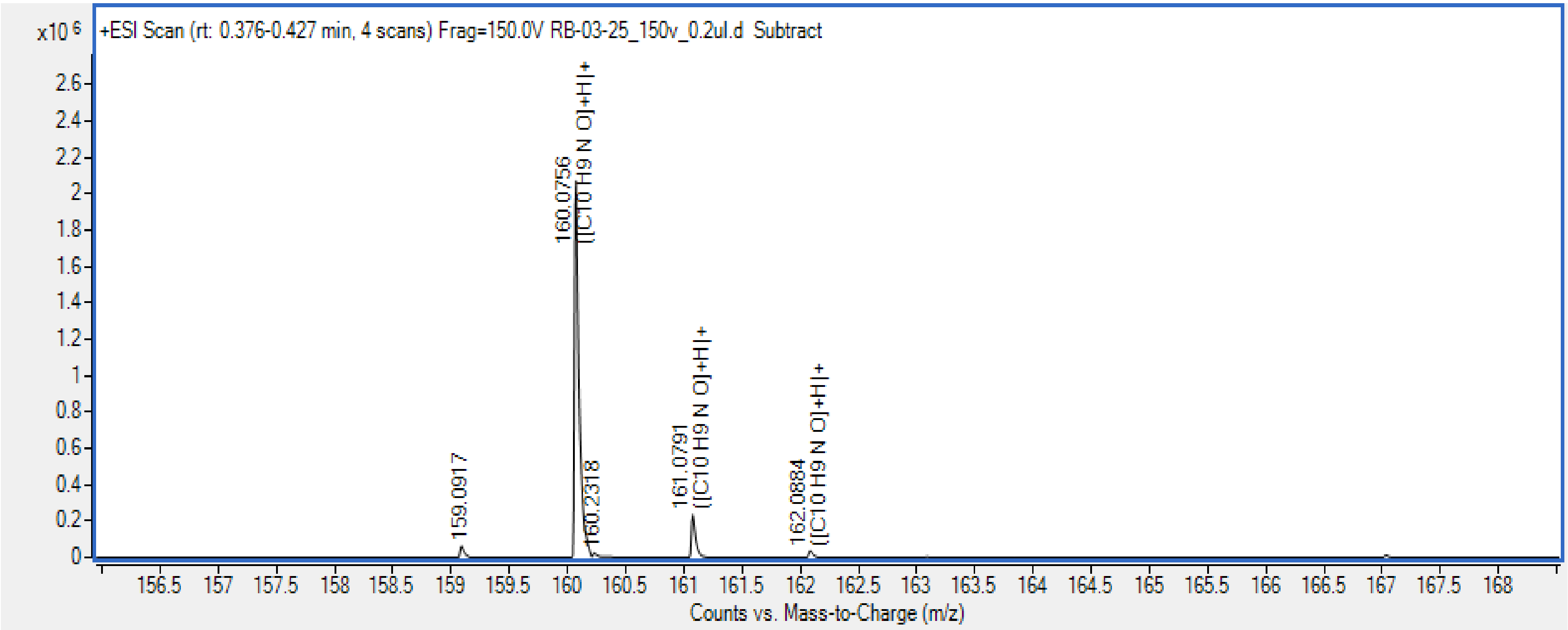

Formula,	Species,	m/z,	Score,	Diff (abs. ppm),	Mass
C10 H9 N O,	(M+H)+,	160.0756,	97.86,	0.38,	159.0685

3-methyl-4,5-dihydronaphtho[1,2-c]isoxazole (P12): ^{1}H-NMR ($CDCl_3$)

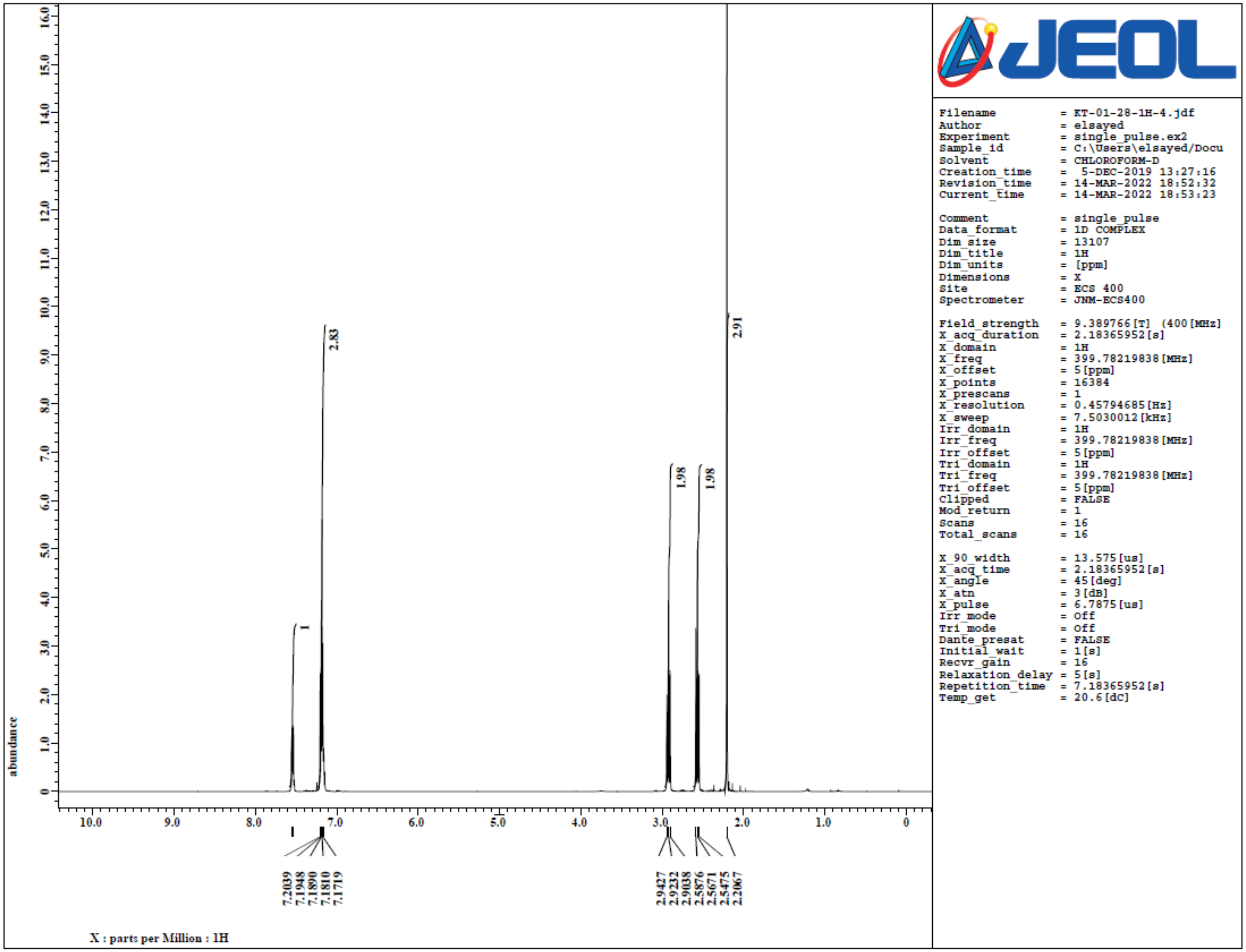

3-methyl-4,5-dihydronaphtho[1,2-c]isoxazole (P12): ^{1}H-NMR ($CDCl_3$) _ Expanded Aromatic Region

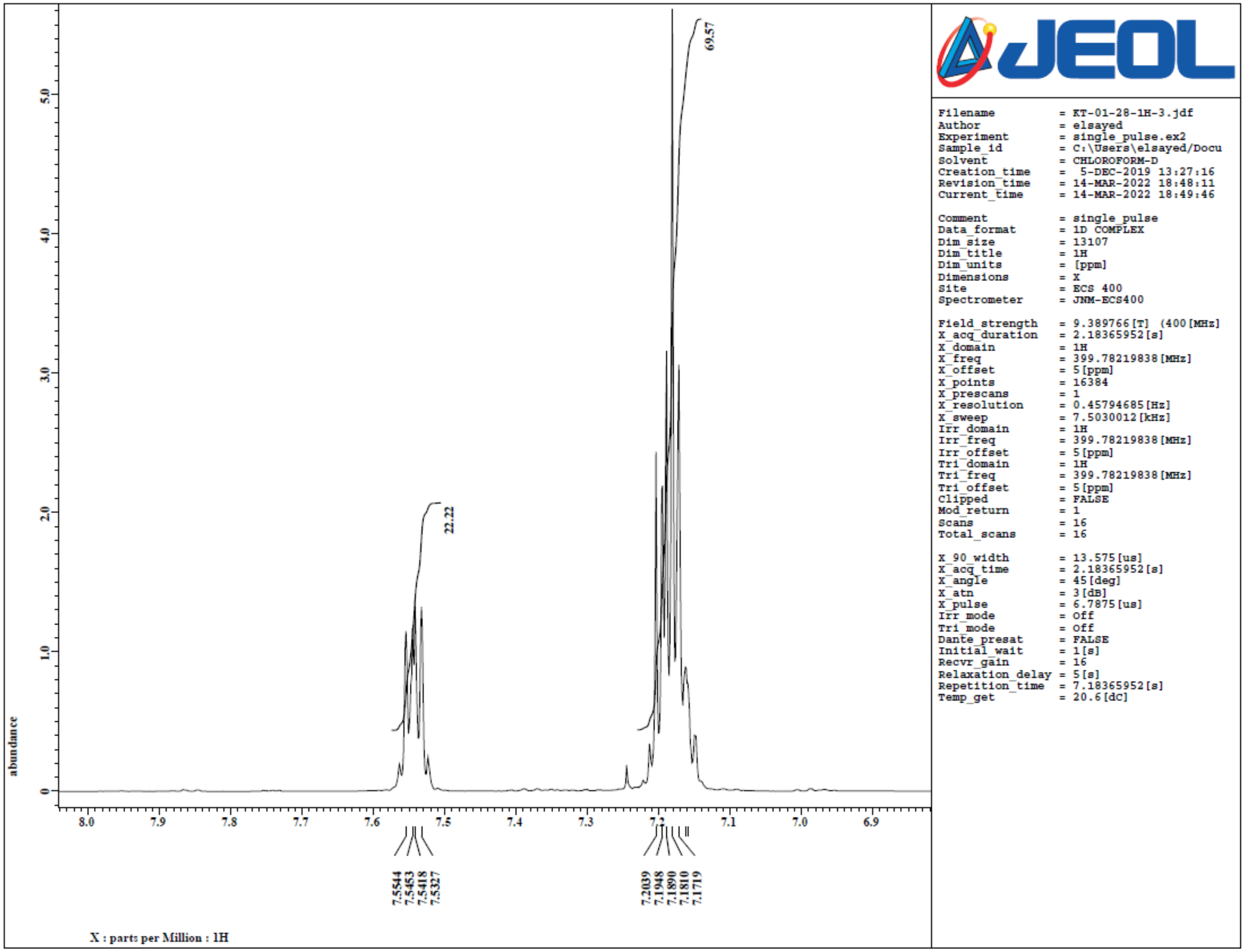

3-methyl-4,5-dihydronaphtho[1,2-c]isoxazole (P12): ^{13}C-NMR ($CDCl_3$)

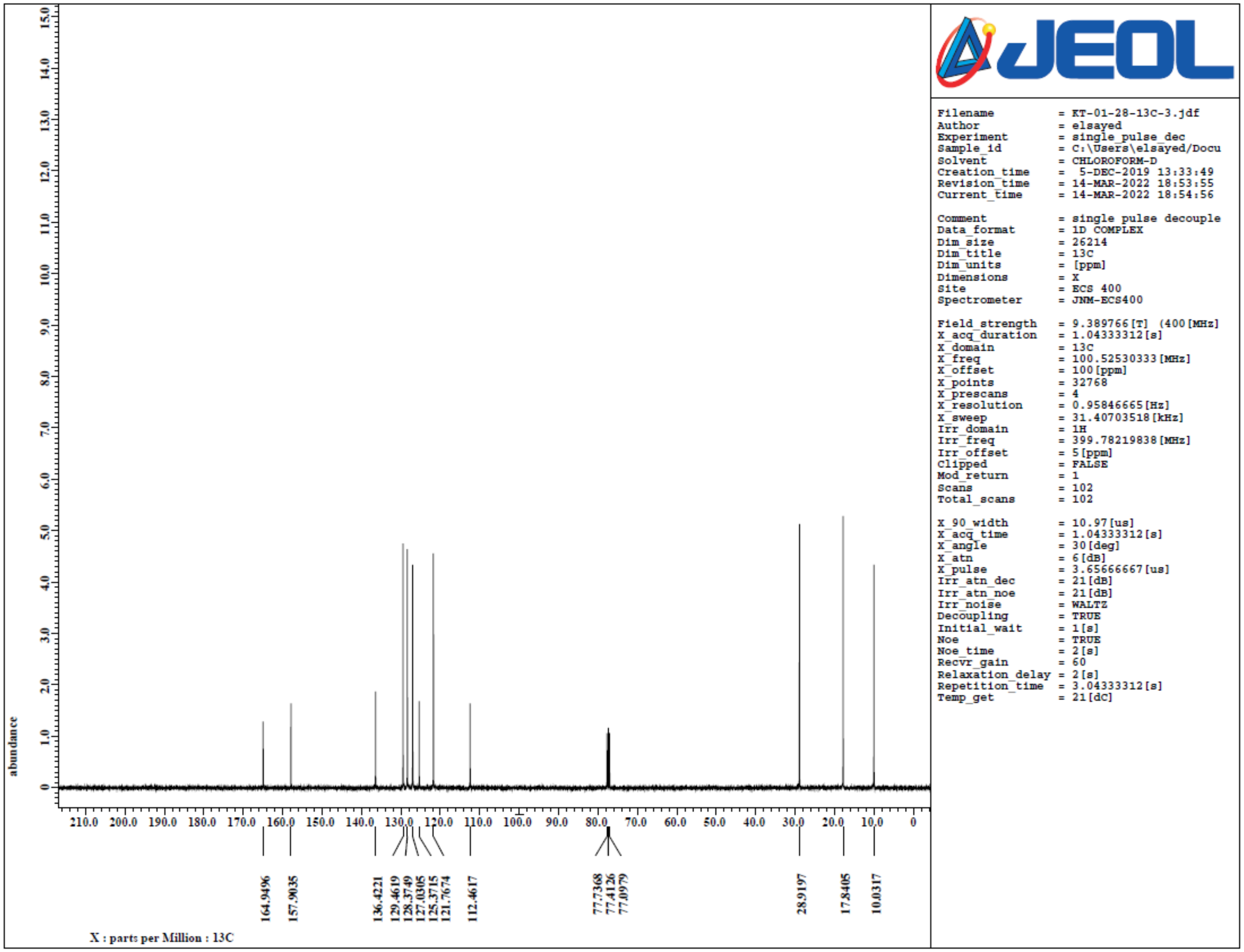

3-methyl-4,5-dihydronaphtho[1,2-c]isoxazole (P12): ESI-MS

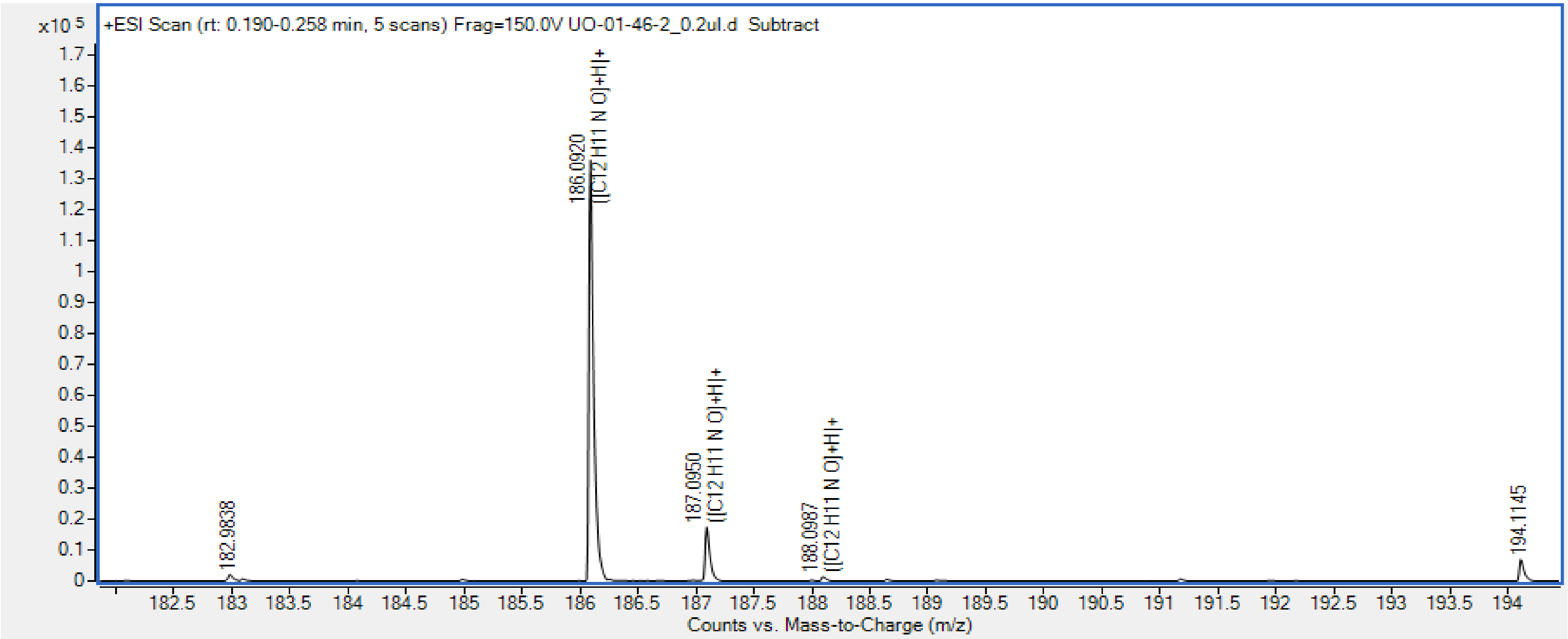

Formula,	Species,	m/z,	Diff (abs. ppm),	Score,	Mass
C12 H11 N O,	(M+H)+,	186.0920,	3.59,	97.51,	185.0847

1,3,5-triphenyl-1H-pyrazole (P13): ^{1}H-NMR ($CDCl_3$)

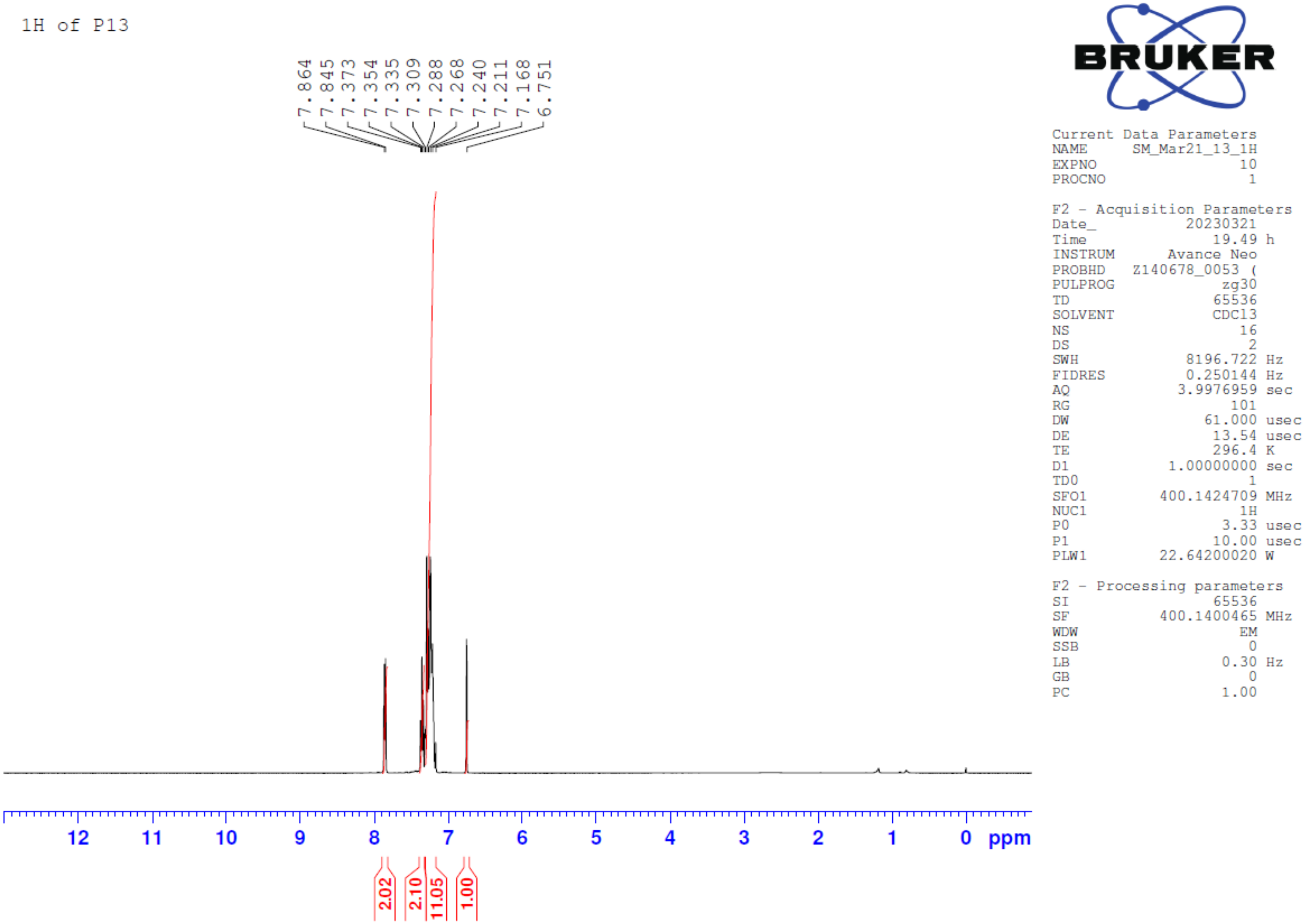

1,3,5-triphenyl-1H-pyrazole (P13): ^{1}H-NMR ($CDCl_3$) _ Expanded Aromatic Region

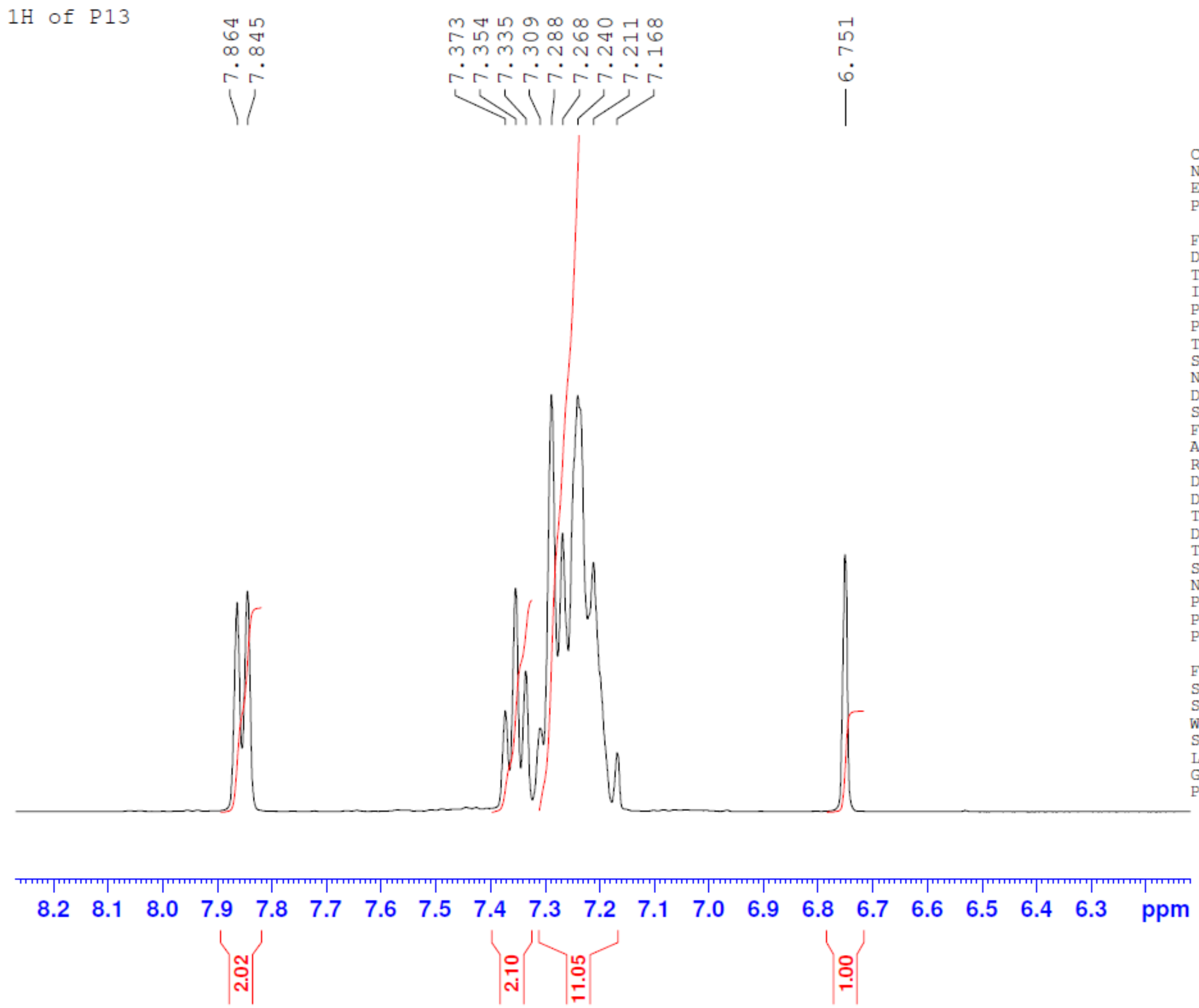

Current Data Parameters
NAME SM_Mar21_13_1H
EXPNO 10
PROCNO 1

F2 - Acquisition Parameters
Date_ 20230321
Time 19.49 h
INSTRUM Avance Neo
PROBHD Z140678_0053 (
PULPROG zg30
TD 65536
SOLVENT CDCl3
NS 16
DS 2
SWH 8196.722 Hz
FIDRES 0.250144 Hz
AQ 3.9976959 sec
RG 101
DW 61.000 usec
DE 13.54 usec
TE 296.4 K
D1 1.00000000 sec
TD0 1
SFO1 400.1424709 MHz
NUC1 1H
P0 3.33 usec
P1 10.00 usec
PLW1 22.64200020 W

F2 - Processing parameters
SI 65536
SF 400.1400465 MHz
WDW EM
SSB 0
LB 0.30 Hz
GB 0
PC 1.00

1,3,5-triphenyl-1H-pyrazole (P13): ^{13}C-NMR ($CDCl_3$)

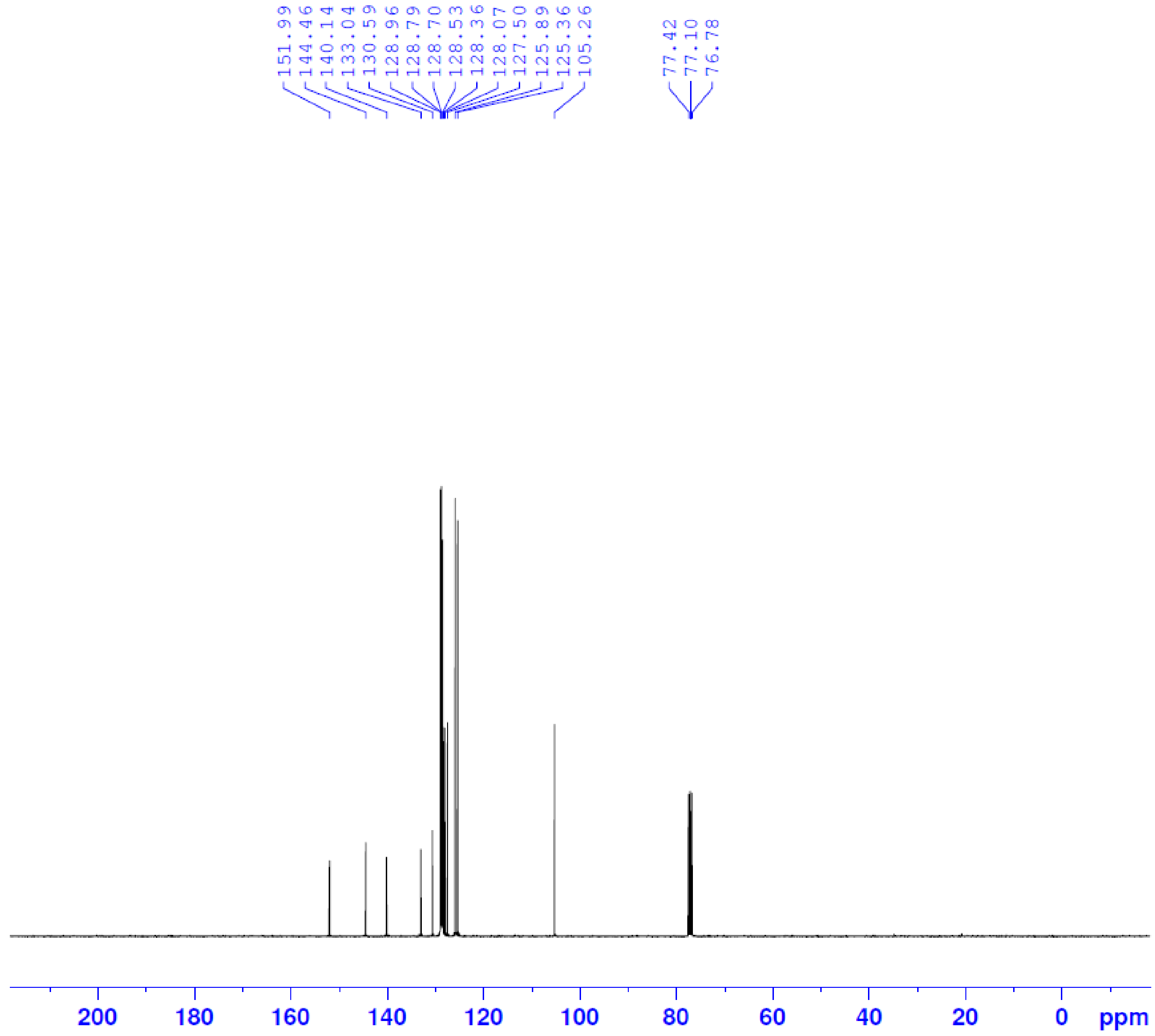

BRUKER

```
Current Data Parameters
NAME      SM_Mar21_P131Hand13C
EXPNO                   11
PROCNO                   1

F2 - Acquisition Parameters
Date_             20230321
Time                 19.42 h
INSTRUM         Avance Neo
PROBHD   Z140678_0053 (
PULPROG            zgpg30
TD                  65536
SOLVENT             CDCl3
NS                   4096
DS                      4
SWH             23809.523 Hz
FIDRES           0.726609 Hz
AQ              1.3762560 sec
RG                   12.7
DW                 21.000 usec
DE                   6.50 usec
TE                  296.9 K
D1             2.00000000 sec
D11            0.03000000 sec
TD0                     1
SFO1          100.6253446 MHz
NUC1                  13C
P0                   3.33 usec
P1                  10.00 usec
PLW1          77.80000305 W
SFO2          400.1416006 MHz
NUC2                   1H
CPDPRG[2          waltz65
PCPD2               90.00 usec
PLW2          22.64200020 W
PLW12          0.27952999 W
PLW13          0.14060000 W

F2 - Processing parameters
SI                  32768
SF            100.6152830 MHz
WDW                    EM
SSB                     0
LB                   1.00 Hz
GB                      0
PC                   1.40
```

1,3,5-triphenyl-1H-pyrazole (P13): ^{13}C-NMR ($CDCl_3$) _ Expanded Aromatic Region

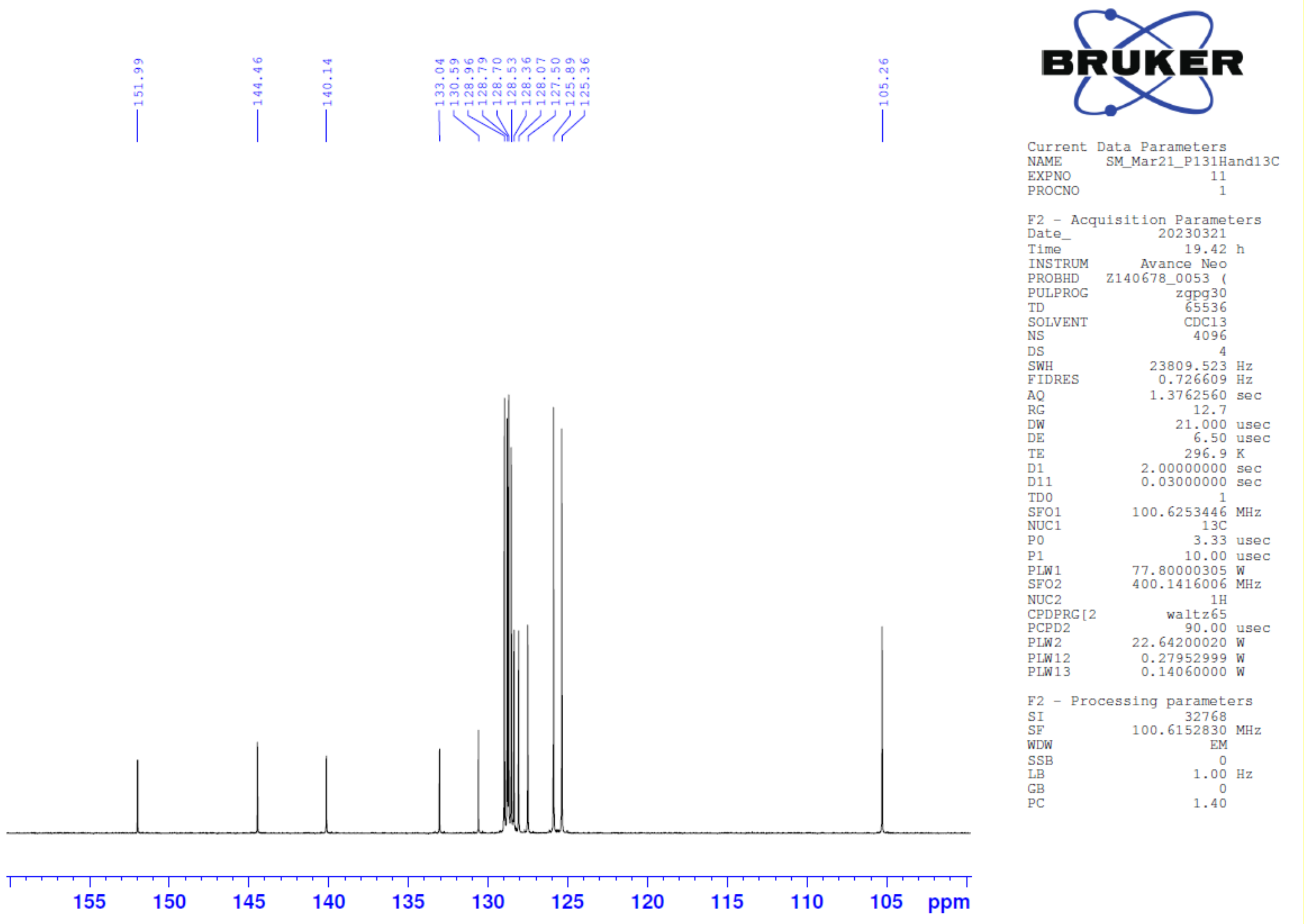

1,3,5-triphenyl-1H-pyrazole (P13): GC-MS

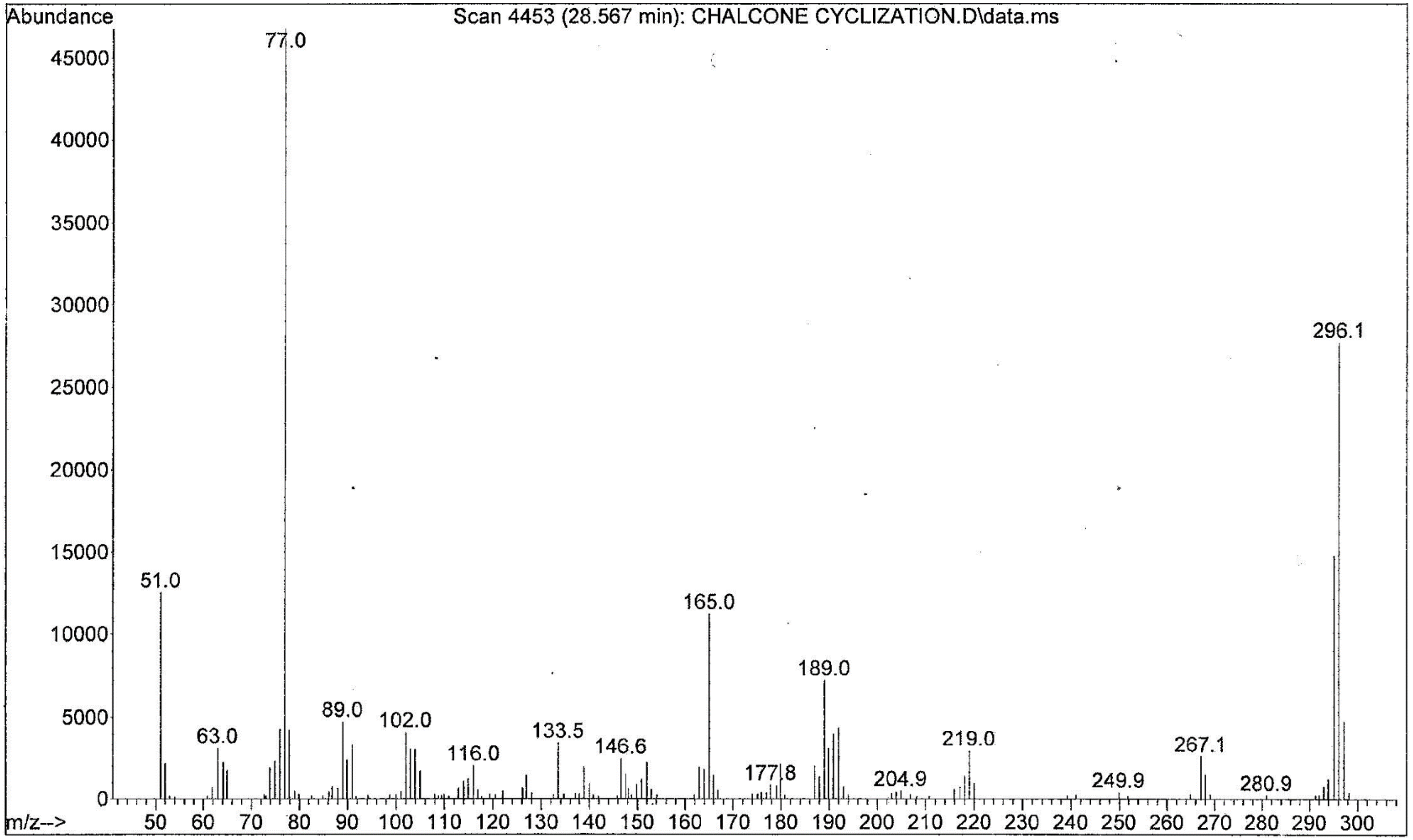

3-methyl-1,5-diphenyl-1H-pyrazole (P14): ^{1}H-NMR ($CDCl_3$)

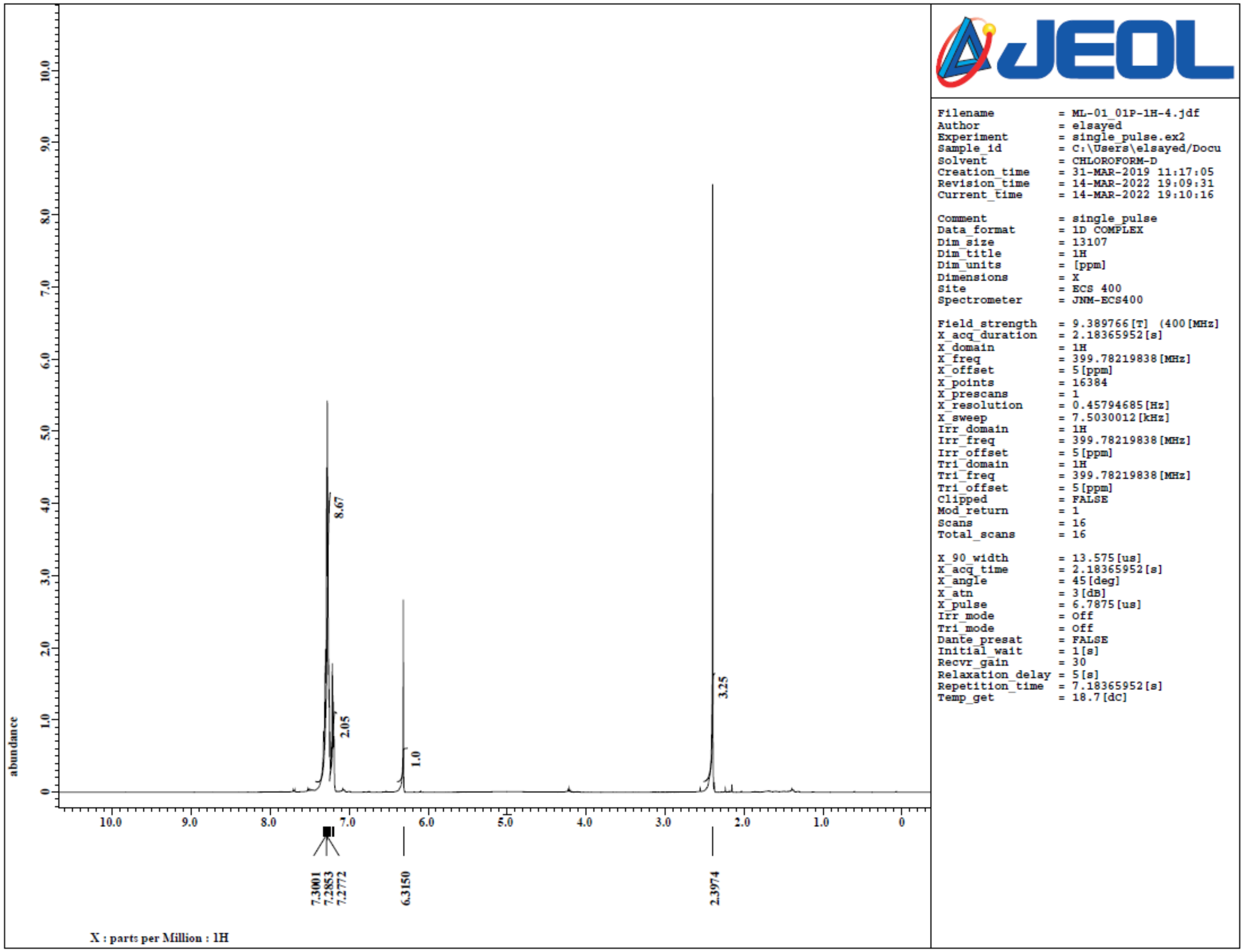

Filename	= ML-01_01P-1H-4.jdf
Author	= elsayed
Experiment	= single_pulse.ex2
Sample_id	= C:\Users\elsayed/Docu
Solvent	= CHLOROFORM-D
Creation_time	= 31-MAR-2019 11:17:05
Revision_time	= 14-MAR-2022 19:09:31
Current_time	= 14-MAR-2022 19:10:16
Comment	= single_pulse
Data_format	= 1D COMPLEX
Dim_size	= 13107
Dim_title	= 1H
Dim_units	= [ppm]
Dimensions	= X
Site	= ECS 400
Spectrometer	= JNM-ECS400
Field_strength	= 9.389766[T] (400[MHz]
X_acq_duration	= 2.18365952[s]
X_domain	= 1H
X_freq	= 399.78219838[MHz]
X_offset	= 5[ppm]
X_points	= 16384
X_prescans	= 1
X_resolution	= 0.45794685[Hz]
X_sweep	= 7.5030012[kHz]
Irr_domain	= 1H
Irr_freq	= 399.78219838[MHz]
Irr_offset	= 5[ppm]
Tri_domain	= 1H
Tri_freq	= 399.78219838[MHz]
Tri_offset	= 5[ppm]
Clipped	= FALSE
Mod_return	= 1
Scans	= 16
Total_scans	= 16
X_90_width	= 13.575[us]
X_acq_time	= 2.18365952[s]
X_angle	= 45[deg]
X_atn	= 3[dB]
X_pulse	= 6.7875[us]
Irr_mode	= Off
Tri_mode	= Off
Dante_presat	= FALSE
Initial_wait	= 1[s]
Recvr_gain	= 30
Relaxation_delay	= 5[s]
Repetition_time	= 7.18365952[s]
Temp_get	= 18.7[dC]

3-methyl-1,5-diphenyl-1H-pyrazole (P14): ^{1}H-NMR ($CDCl_3$) _ Expanded Aromatic Region

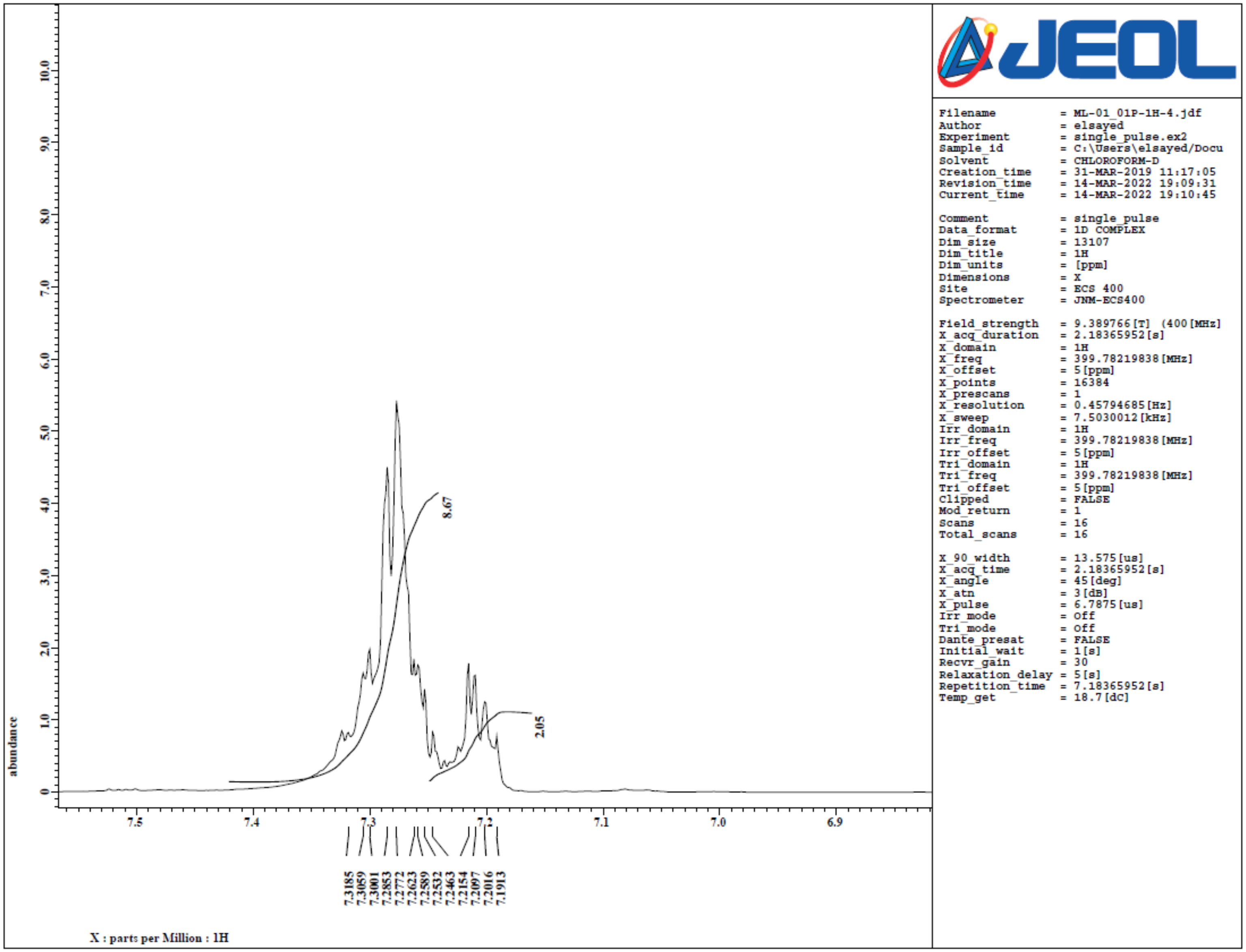

3-methyl-1,5-diphenyl-1H-pyrazole (P14): ^{13}C-NMR ($CDCl_3$)

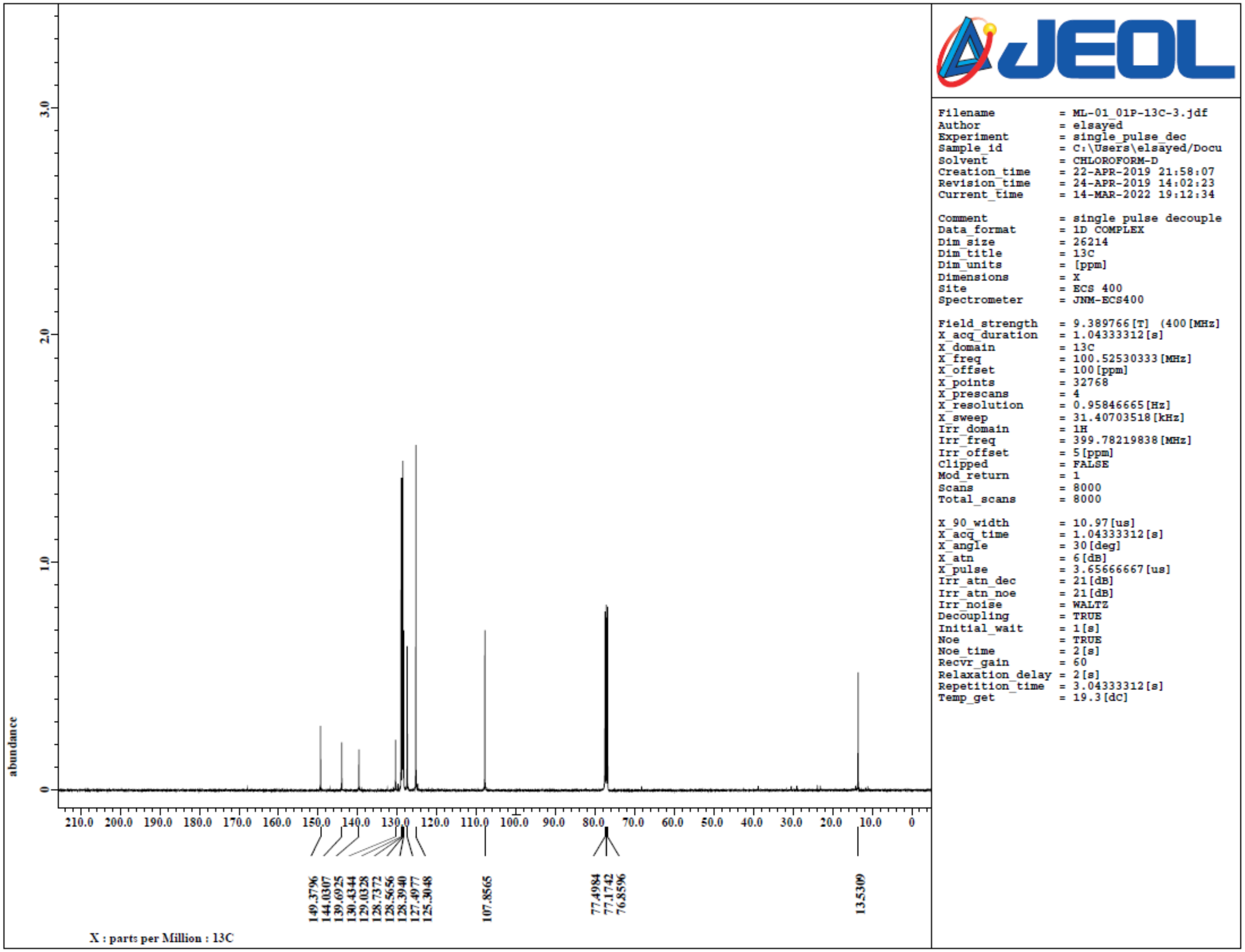

3-methyl-1,5-diphenyl-1H-pyrazole (P14): ^{13}C-NMR ($CDCl_3$) _ Expanded Aromatic Region

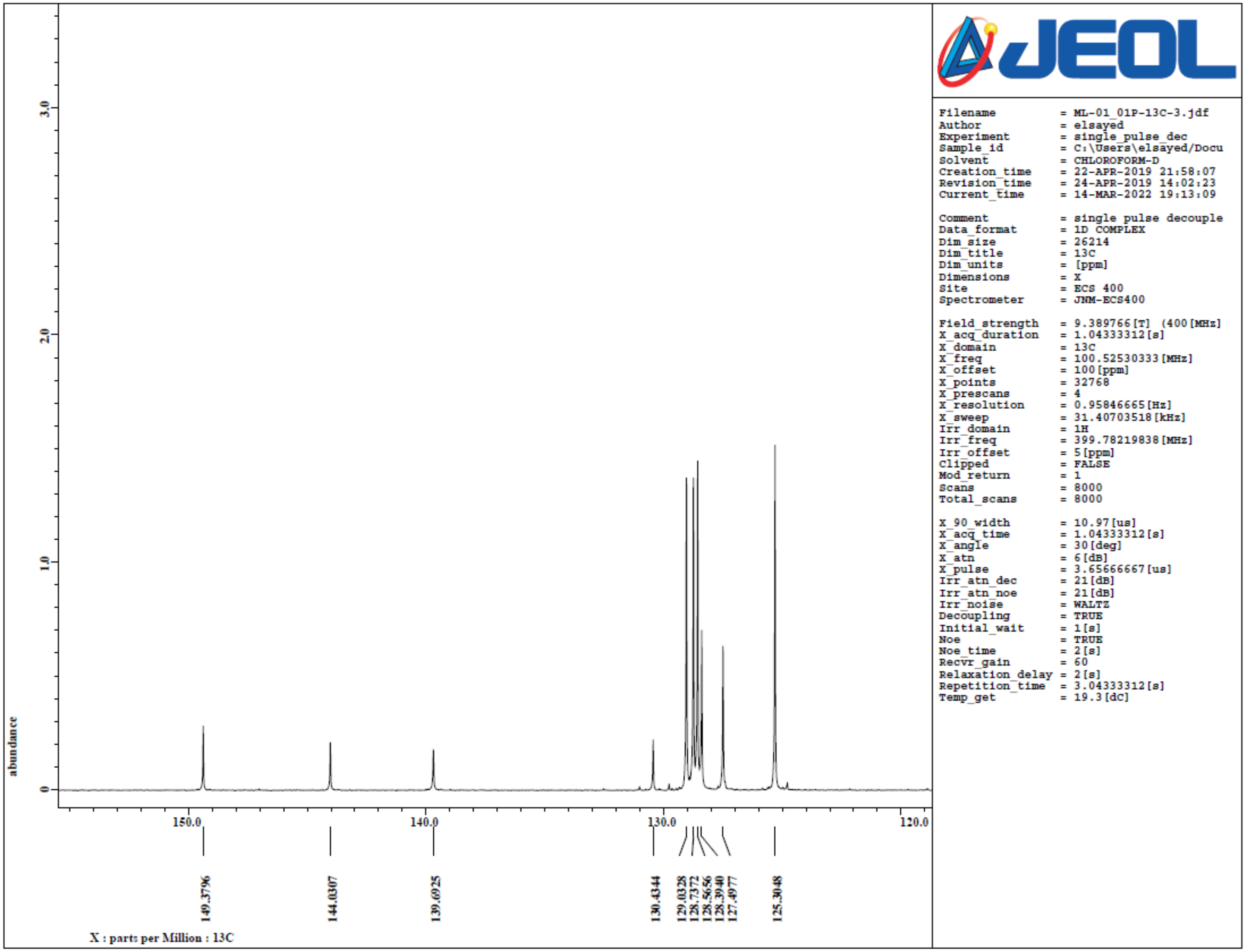

3-methyl-1,5-diphenyl-1H-pyrazole (P14): ESI-MS

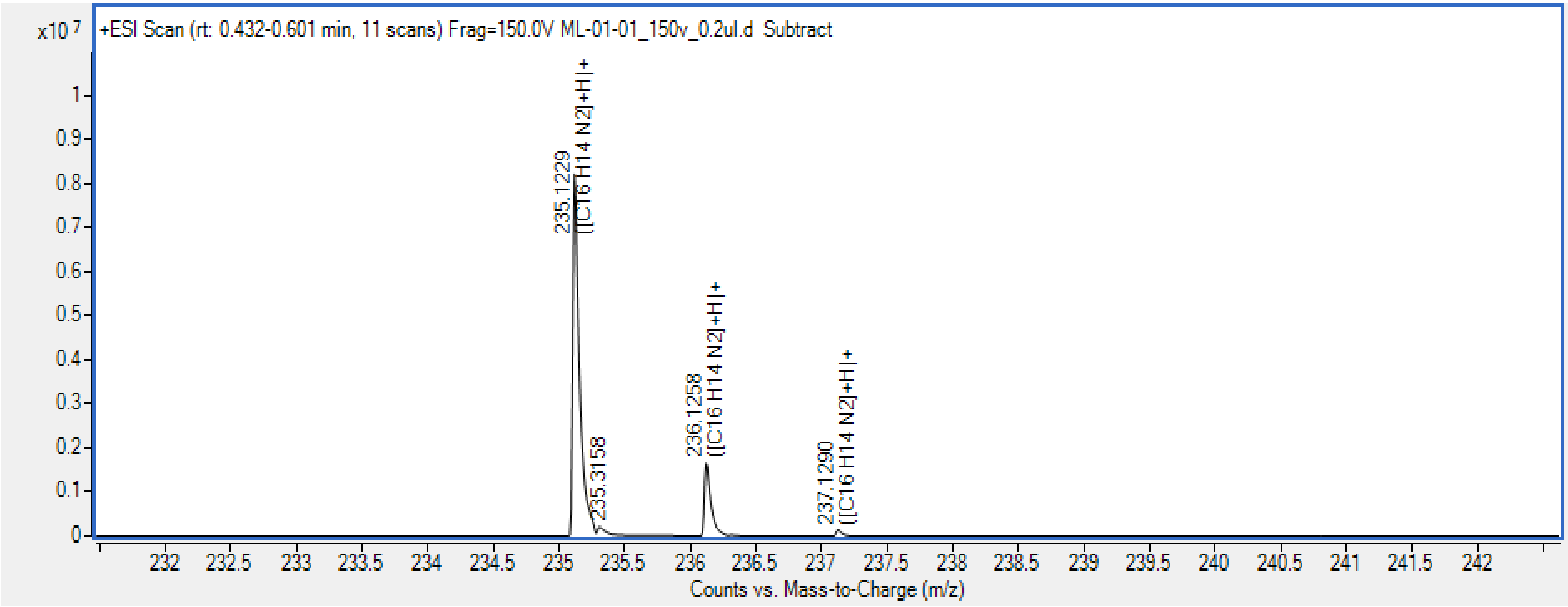

Formula,	Species,	m/z,	Score,	Diff (abs. ppm),	Mass
C16 H14 N2,	(M+H)+,	235.1229,	98.87,	0.54,	234.1156

1,5-diphenyl-3-(trifluoromethyl)-1H-pyrazole (P15): ^{1}H-NMR ($CDCl_3$)

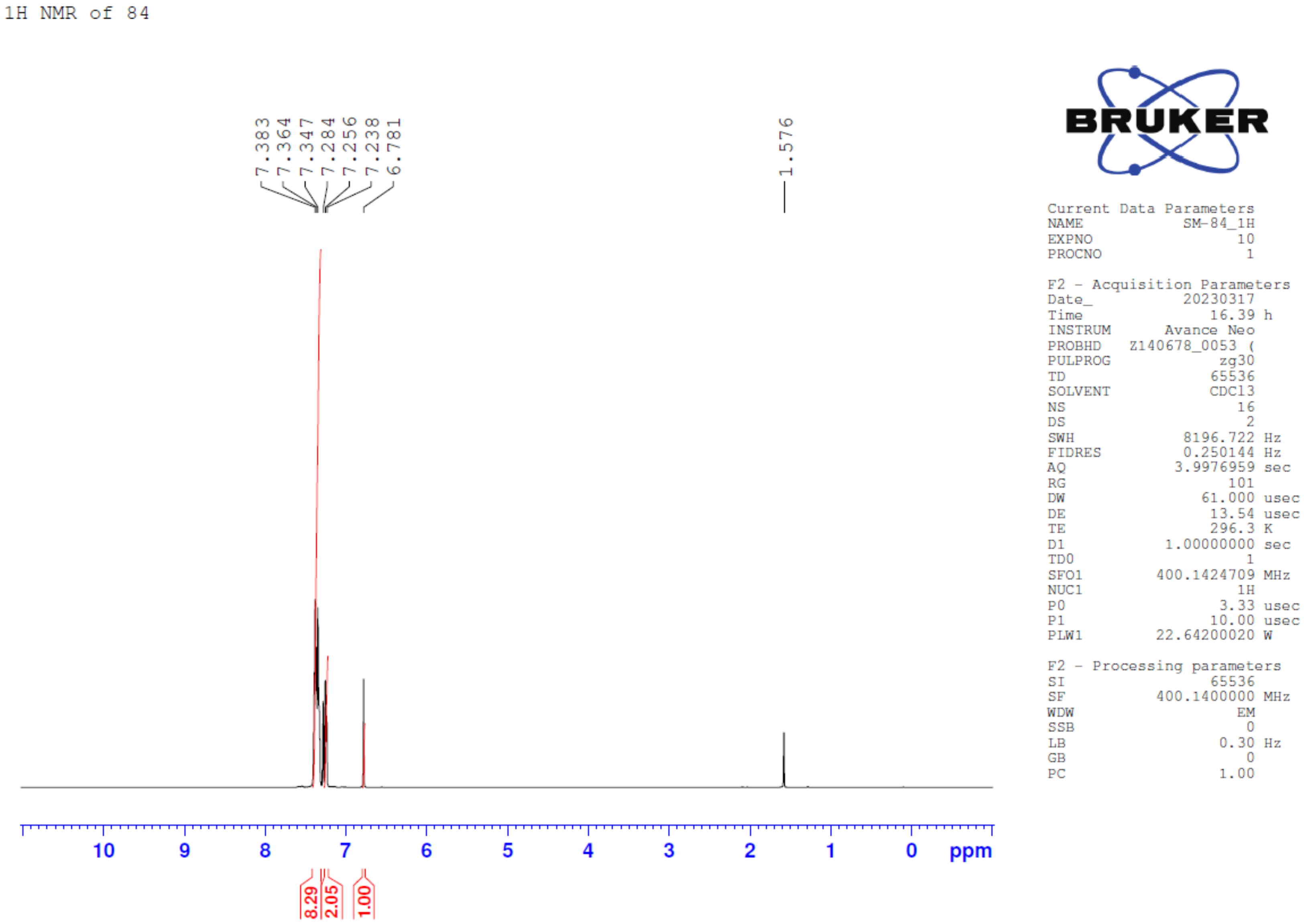

1,5-diphenyl-3-(trifluoromethyl)-1H-pyrazole (P15): ^{1}H-NMR ($CDCl_3$) _ Expanded Aromatic Region

1H NMR of 84

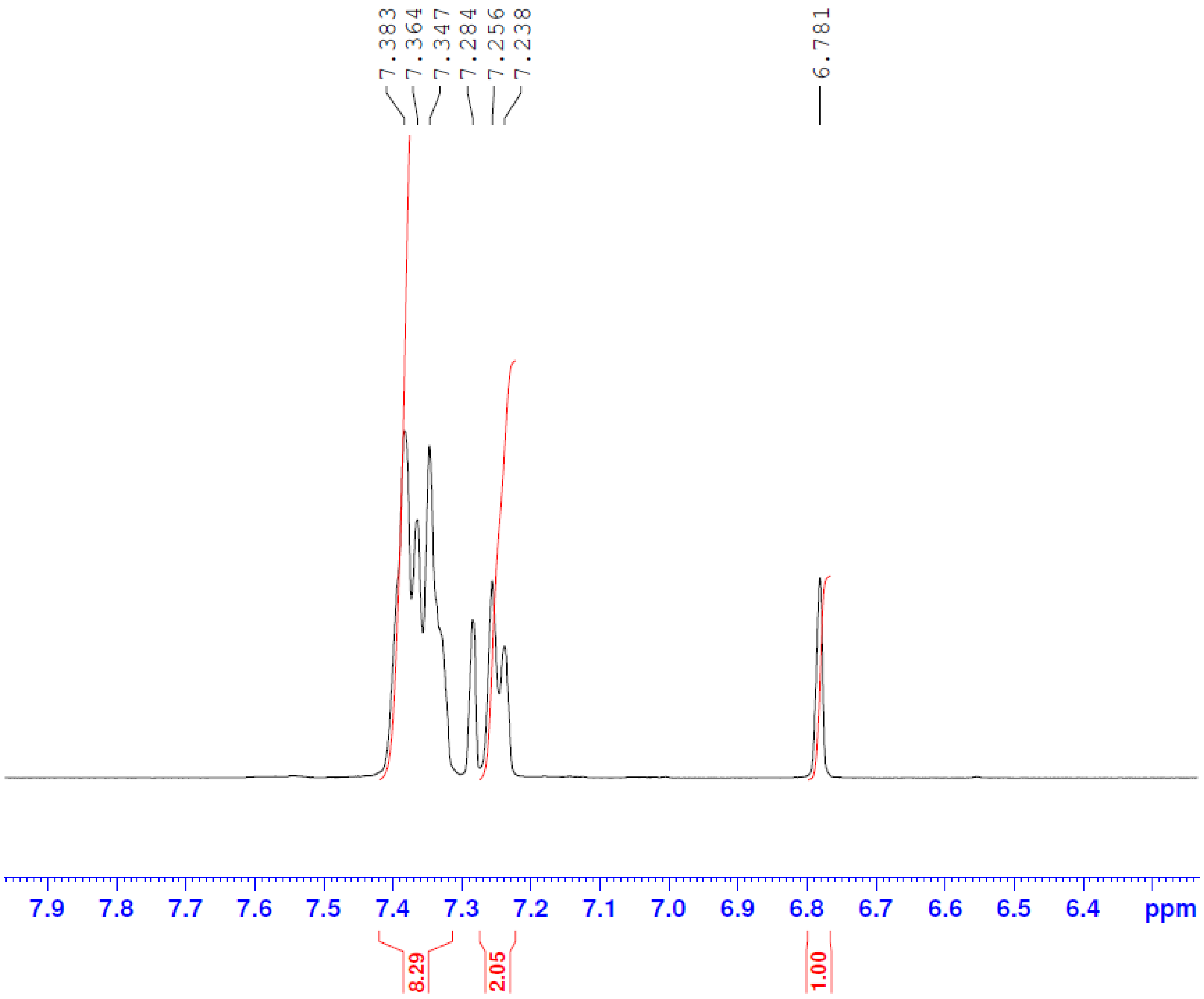

Current Data Parameters
NAME SM-84_1H
EXPNO 10
PROCNO 1

F2 - Acquisition Parameters
Date_ 20230317
Time 16.39 h
INSTRUM Avance Neo
PROBHD Z140678_0053 (
PULPROG zg30
TD 65536
SOLVENT CDCl3
NS 16
DS 2
SWH 8196.722 Hz
FIDRES 0.250144 Hz
AQ 3.9976959 sec
RG 101
DW 61.000 usec
DE 13.54 usec
TE 296.3 K
D1 1.00000000 sec
TD0 1
SFO1 400.1424709 MHz
NUC1 1H
P0 3.33 usec
P1 10.00 usec
PLW1 22.64200020 W

F2 - Processing parameters
SI 65536
SF 400.1400000 MHz
WDW EM
SSB 0
LB 0.30 Hz
GB 0
PC 1.00

1,5-diphenyl-3-(trifluoromethyl)-1H-pyrazole (P15): ^{13}C-NMR ($CDCl_3$)

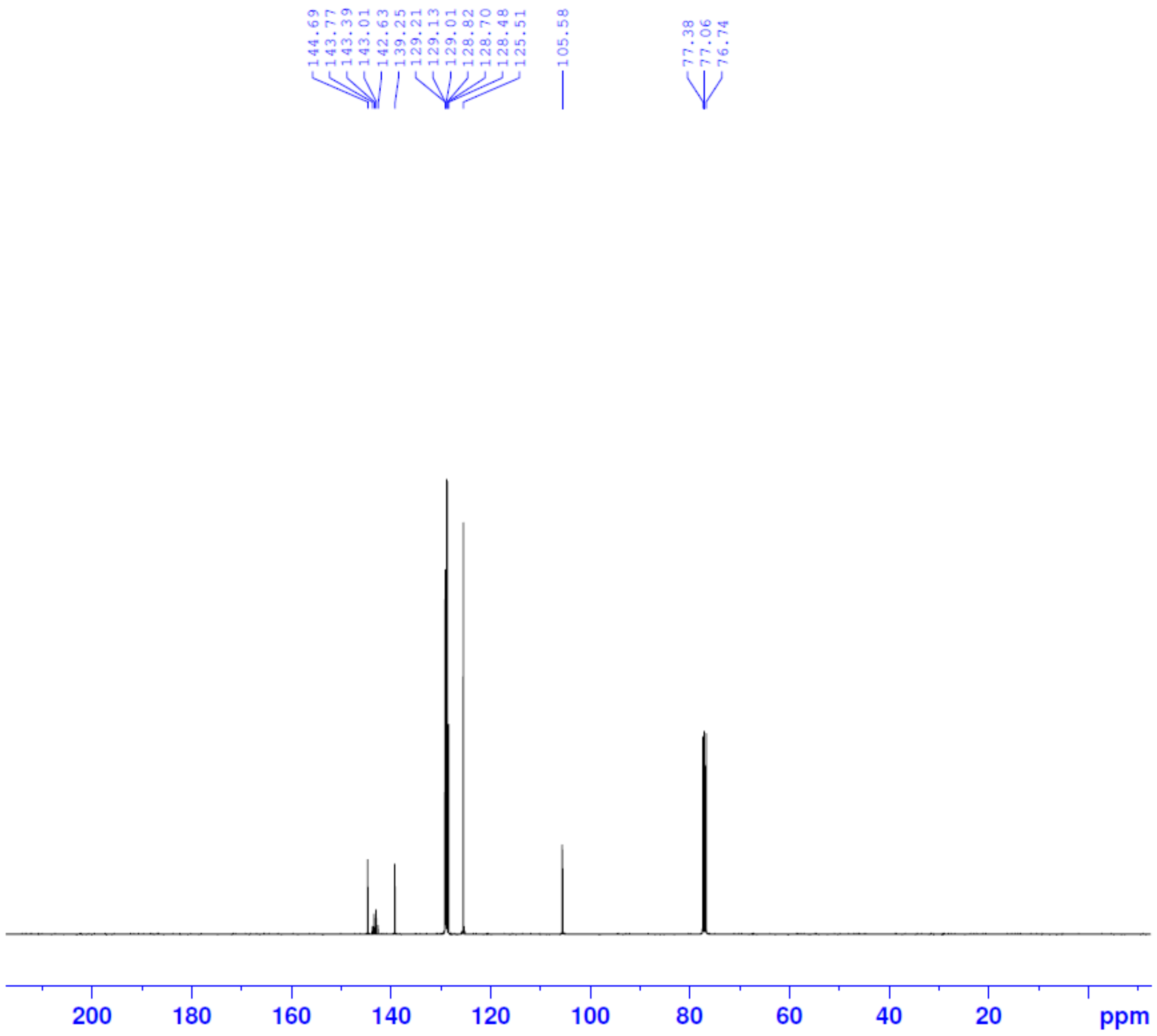


```
Current Data Parameters
NAME      SM-84_1Hand13C
EXPNO                 11
PROCNO                 1

F2 - Acquisition Parameters
Date_           20230318
Time               13.27 h
INSTRUM       Avance Neo
PROBHD    Z140678_0053 (
PULPROG          zgpg30
TD                 65536
SOLVENT            CDCl3
NS                  4096
DS                     4
SWH            23809.523 Hz
FIDRES          0.726609 Hz
AQ             1.3762560 sec
RG                    16
DW                21.000 usec
DE                  6.50 usec
TE                 296.2 K
D1            2.00000000 sec
D11           0.03000000 sec
TD0                    1
SFO1         100.6253446 MHz
NUC1                 13C
P0                  3.33 usec
P1                 10.00 usec
PLW1         77.80000305 W
SFO2         400.1416006 MHz
NUC2                  1H
CPDPRG[2         waltz65
PCPD2              90.00 usec
PLW2         22.64200020 W
PLW12         0.27952999 W
PLW13         0.14060000 W

F2 - Processing parameters
SI                 32768
SF           100.6152830 MHz
WDW                   EM
SSB                    0
LB                  1.00 Hz
GB                     0
PC                  1.40
```

1,5-diphenyl-3-(trifluoromethyl)-1H-pyrazole (P15): ^{13}C-NMR ($CDCl_3$) _ Expanded Aromatic Region

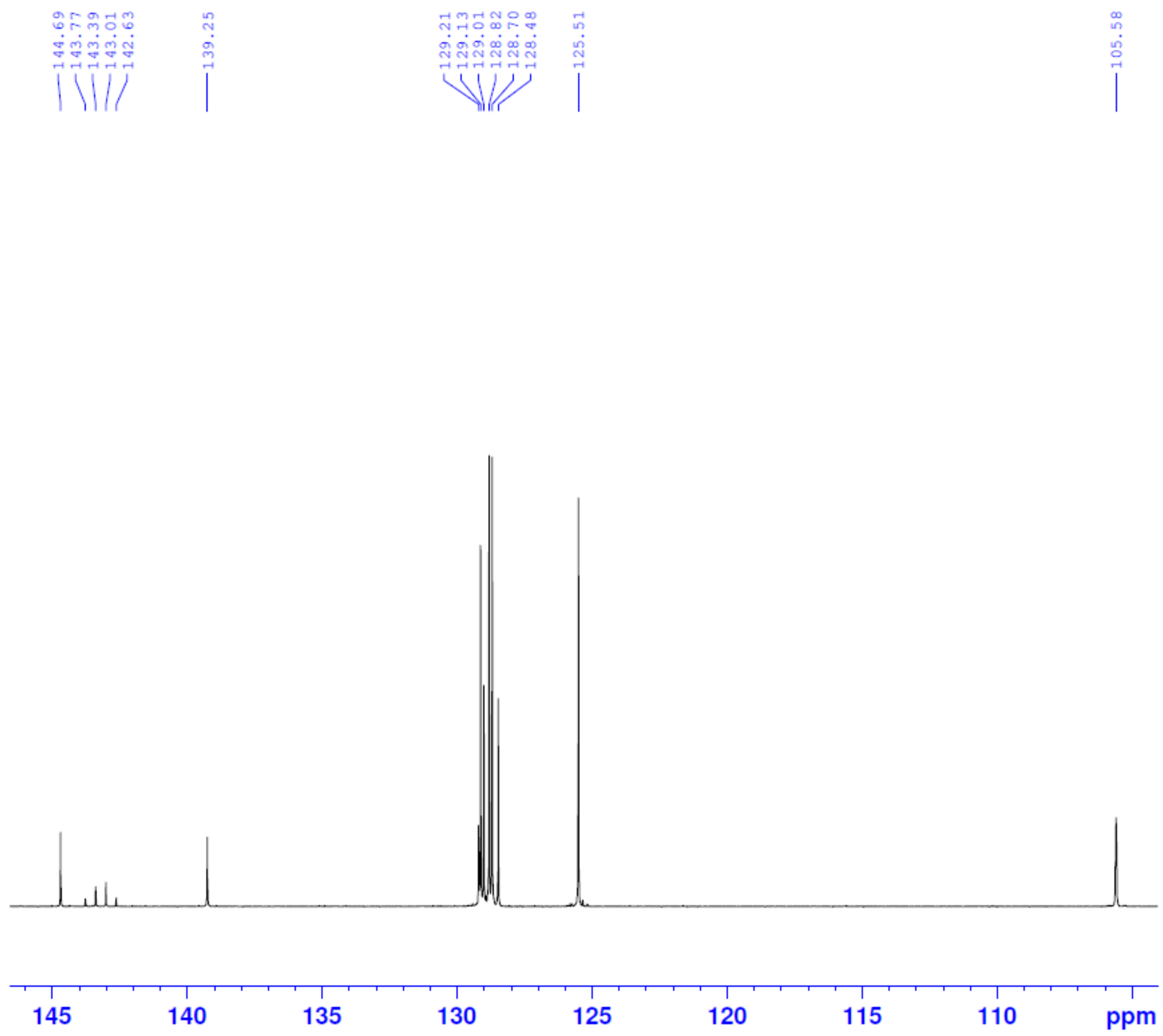

BRUKER

Current Data Parameters
NAME SM-84_1Hand13C
EXPNO 11
PROCNO 1

F2 - Acquisition Parameters
Date_ 20230318
Time 13.27 h
INSTRUM Avance Neo
PROBHD Z140678_0053 (
PULPROG zgpg30
TD 65536
SOLVENT CDCl3
NS 4096
DS 4
SWH 23809.523 Hz
FIDRES 0.726609 Hz
AQ 1.3762560 sec
RG 16
DW 21.000 usec
DE 6.50 usec
TE 296.2 K
D1 2.00000000 sec
D11 0.03000000 sec
TD0 1
SFO1 100.6253446 MHz
NUC1 13C
P0 3.33 usec
P1 10.00 usec
PLW1 77.80000305 W
SFO2 400.1416006 MHz
NUC2 1H
CPDPRG[2 waltz65
PCPD2 90.00 usec
PLW2 22.64200020 W
PLW12 0.27952999 W
PLW13 0.14060000 W

F2 - Processing parameters
SI 32768
SF 100.6152830 MHz
WDW EM
SSB 0
LB 1.00 Hz
GB 0
PC 1.40

1,5-diphenyl-3-(trifluoromethyl)-1H-pyrazole (P15): ESI-MS

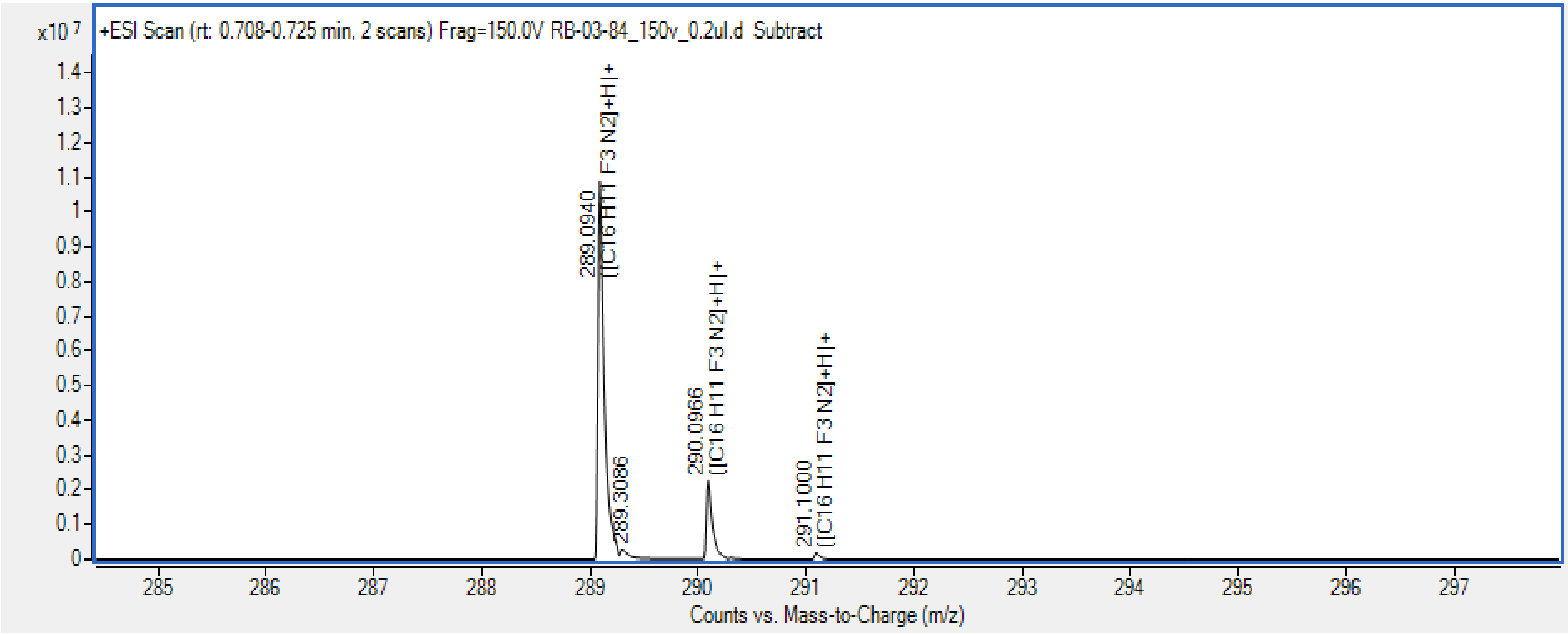

Formula,	Species,	m/z,	Score,	Diff (abs. ppm),	Mass
C16 H11 F3 N2,	(M+H)+,	289.0940,	95.7,	2.66,	288.0867

1-(4-bromophenyl)-3-methyl-5-phenyl-1H-pyrazole (P16): ^{1}H-NMR ($CDCl_3$)

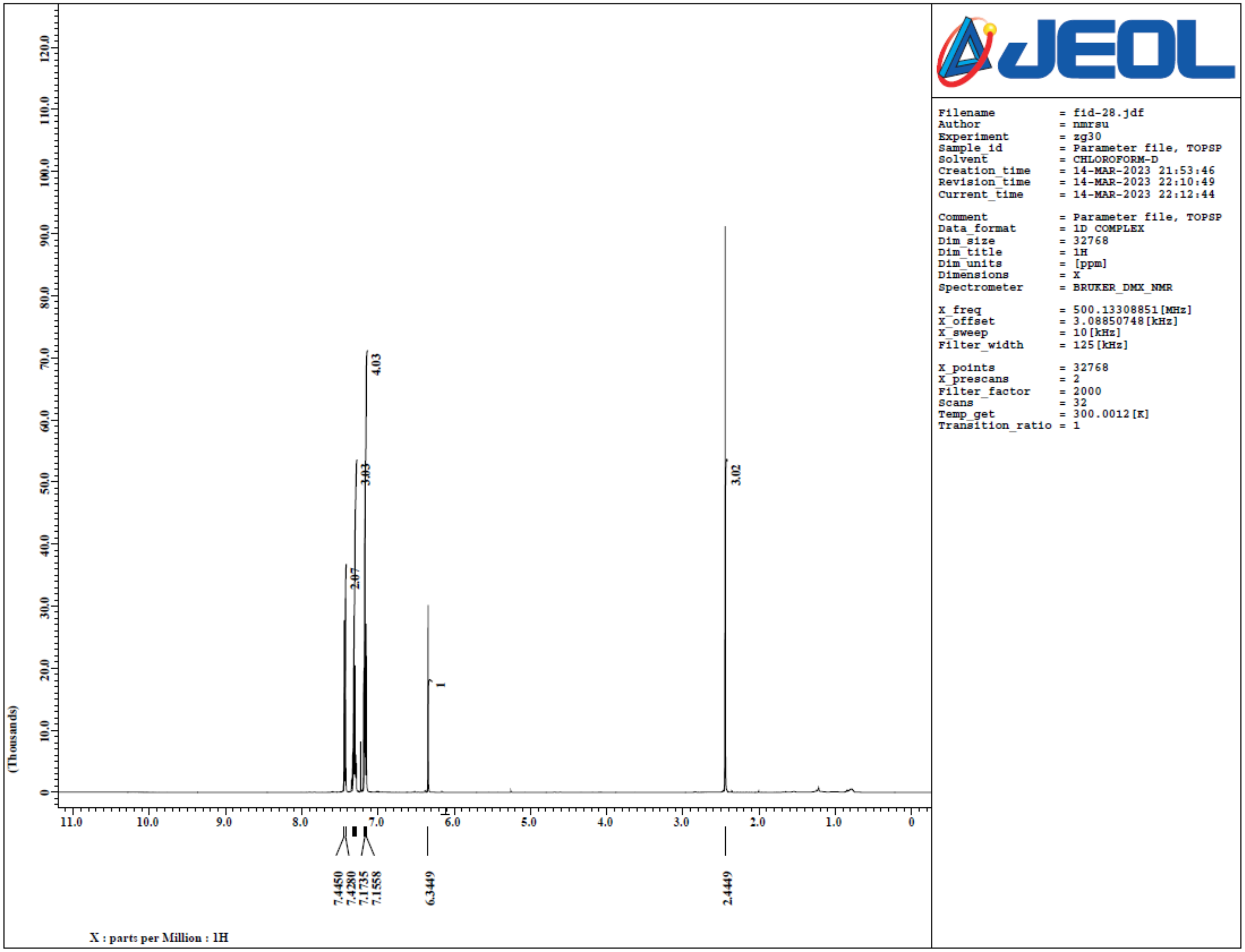

1-(4-bromophenyl)-3-methyl-5-phenyl-1H-pyrazole (P16): ^{1}H-NMR ($CDCl_3$) _ Expanded Aromatic Region

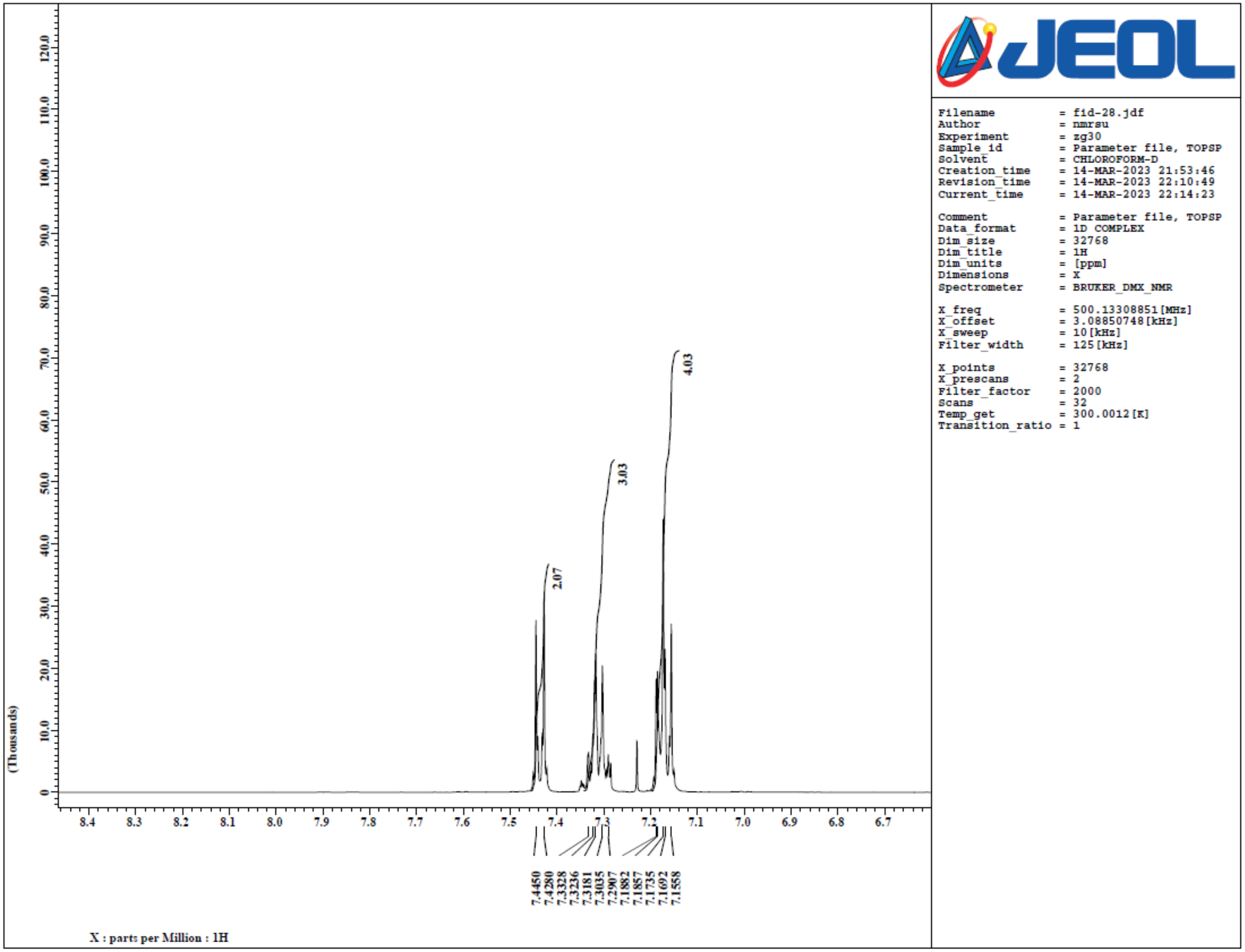

1-(4-bromophenyl)-3-methyl-5-phenyl-1H-pyrazole (P16): ^{13}C-NMR ($CDCl_3$)

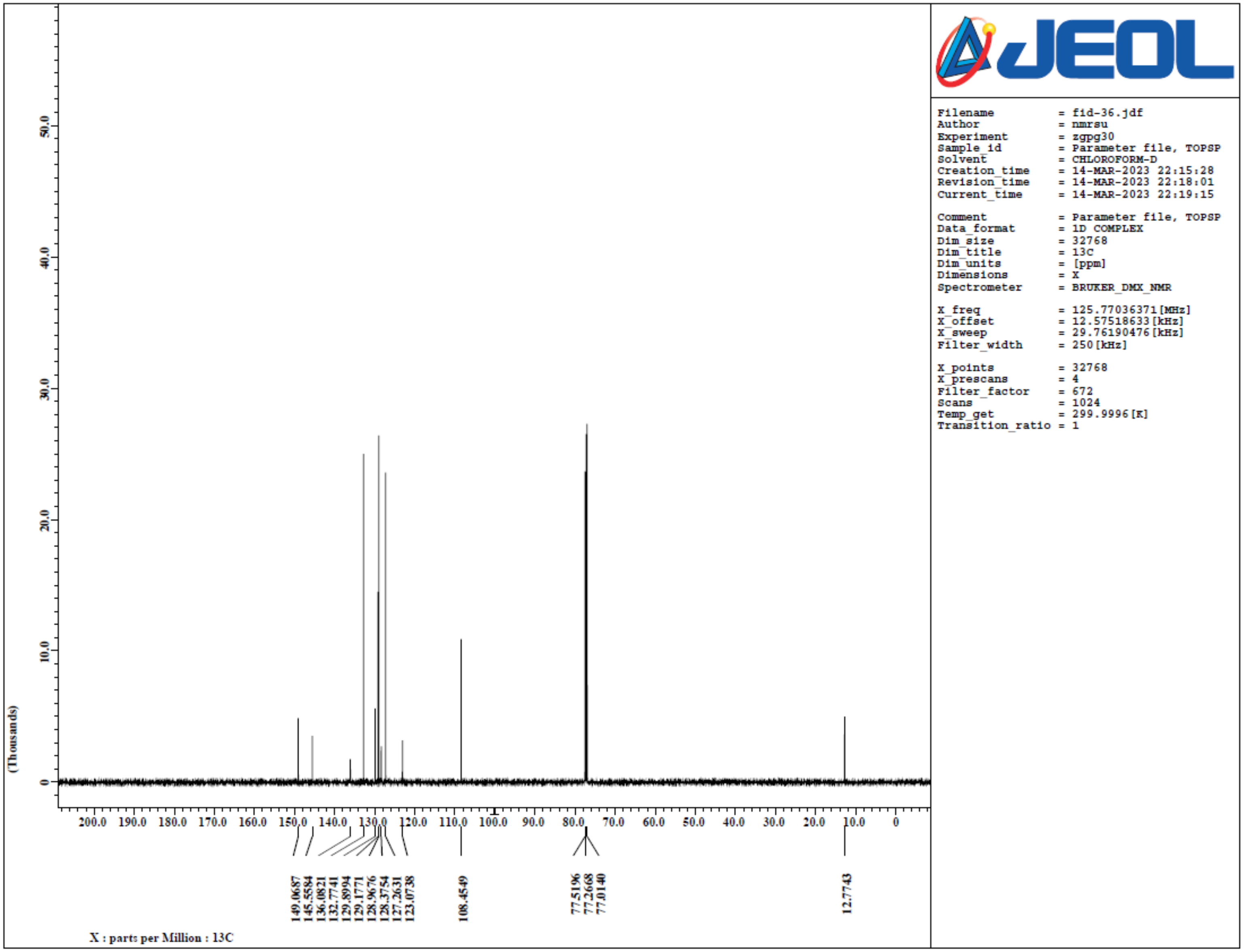

1-(4-bromophenyl)-3-methyl-5-phenyl-1H-pyrazole (P16): ^{13}C-NMR ($CDCl_3$) _ Expanded Aromatic Region

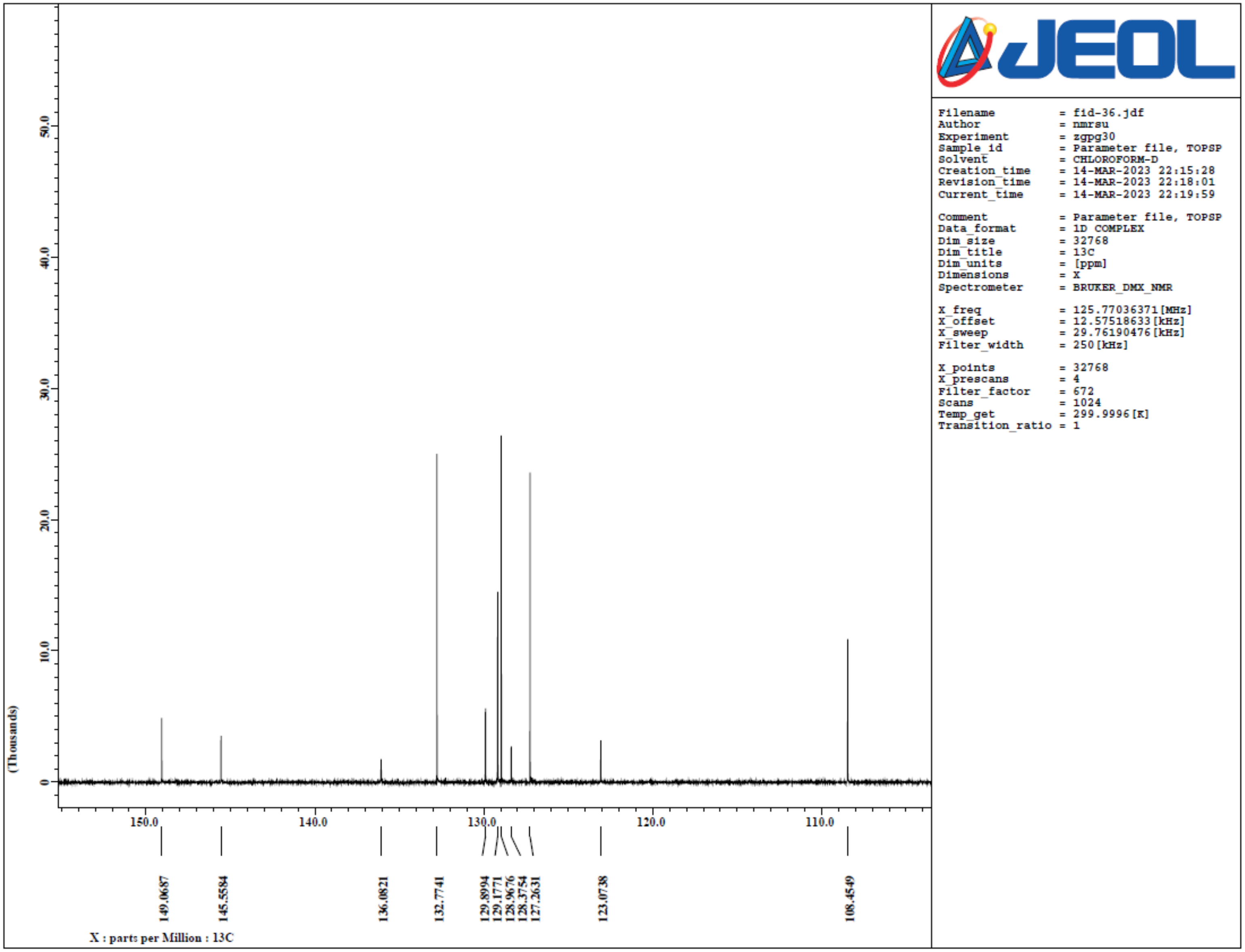

1-(4-bromophenyl)-3-methyl-5-phenyl-1H-pyrazole (P16): ESI-MS

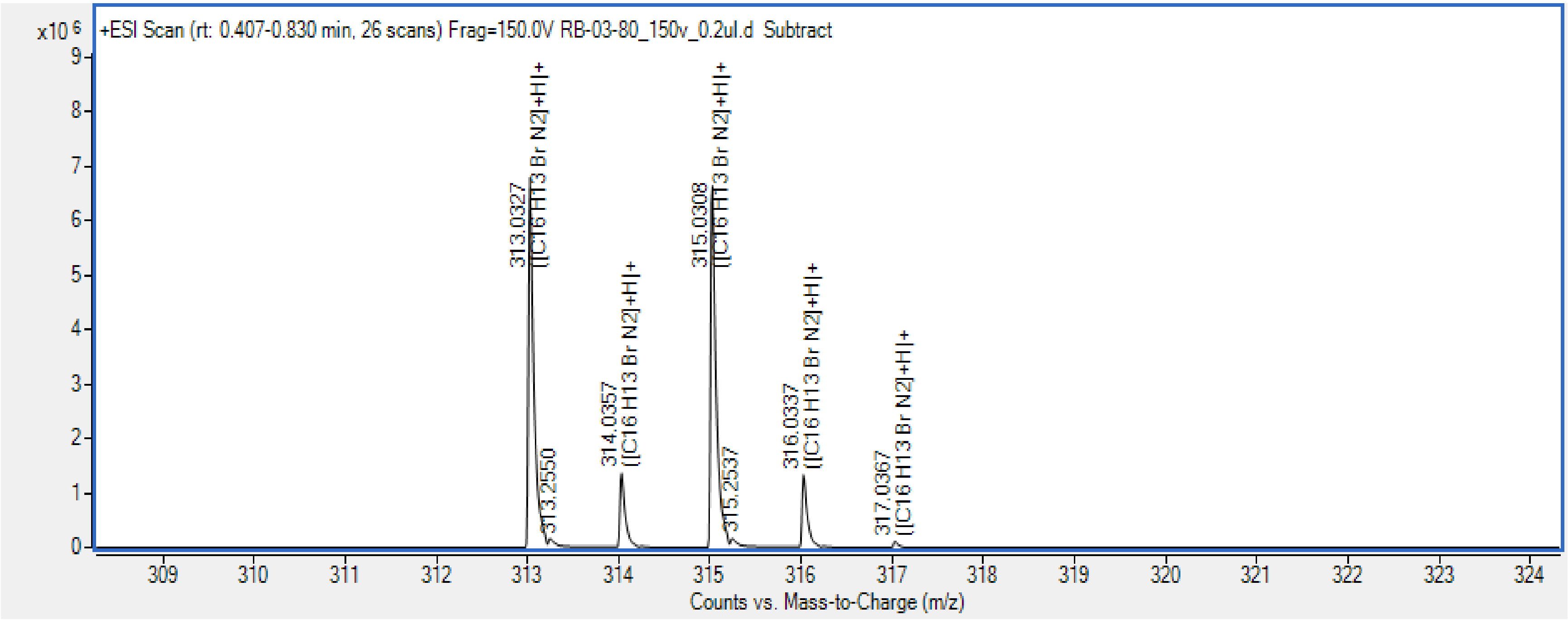

Formula,	Species,	m/z,	Score,	Diff (abs. ppm),	Mass
C16 H13 Br N2,	(M+H)+,	313.0327,	96.99,	2.58,	312.0254

1-(4-chlorophenyl)-3-methyl-5-phenyl-1H-pyrazole (P17): ^{1}H-NMR ($CDCl_3$)

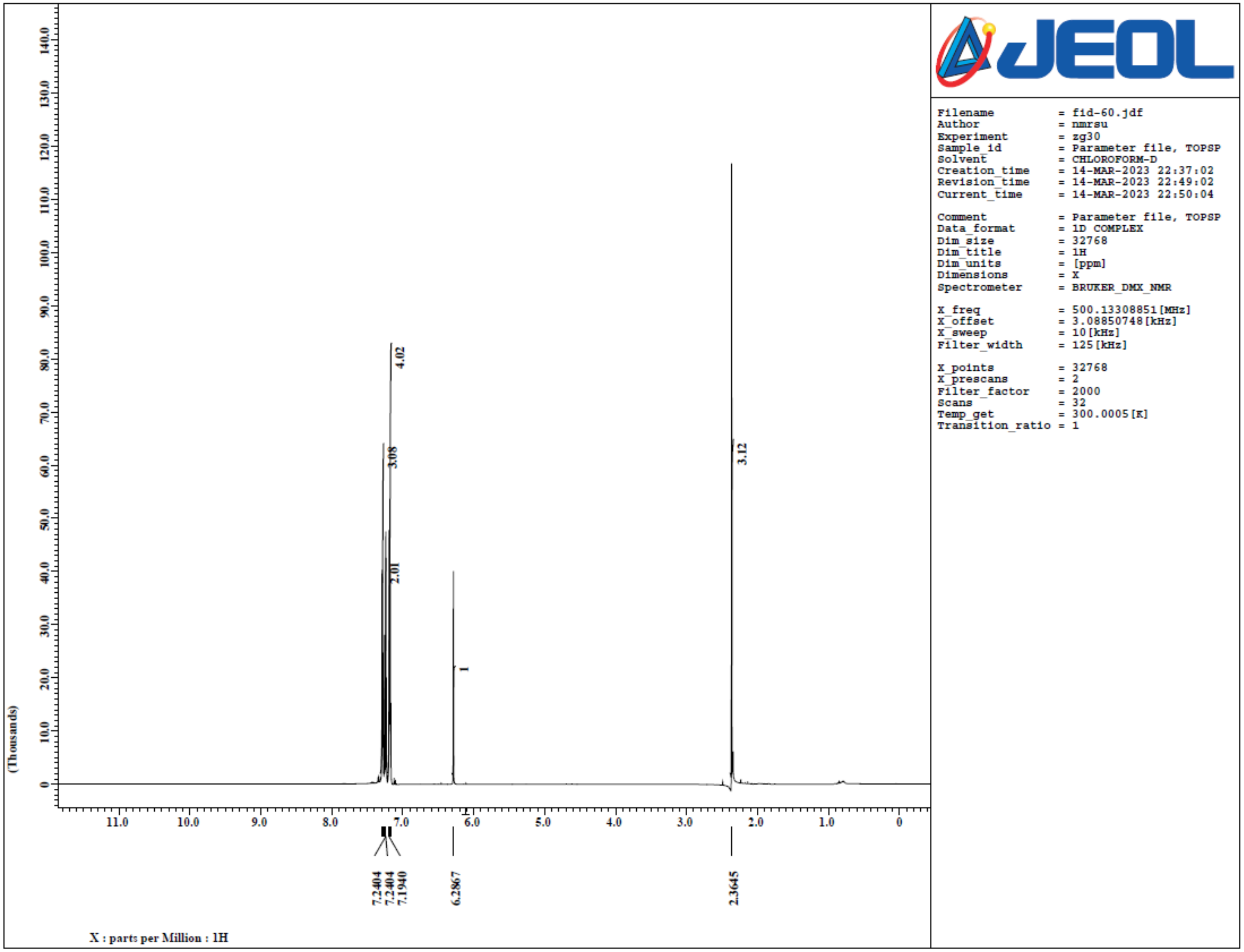

1-(4-chlorophenyl)-3-methyl-5-phenyl-1H-pyrazole (P17): ^{1}H-NMR ($CDCl_3$) _ Expanded Aromatic Region

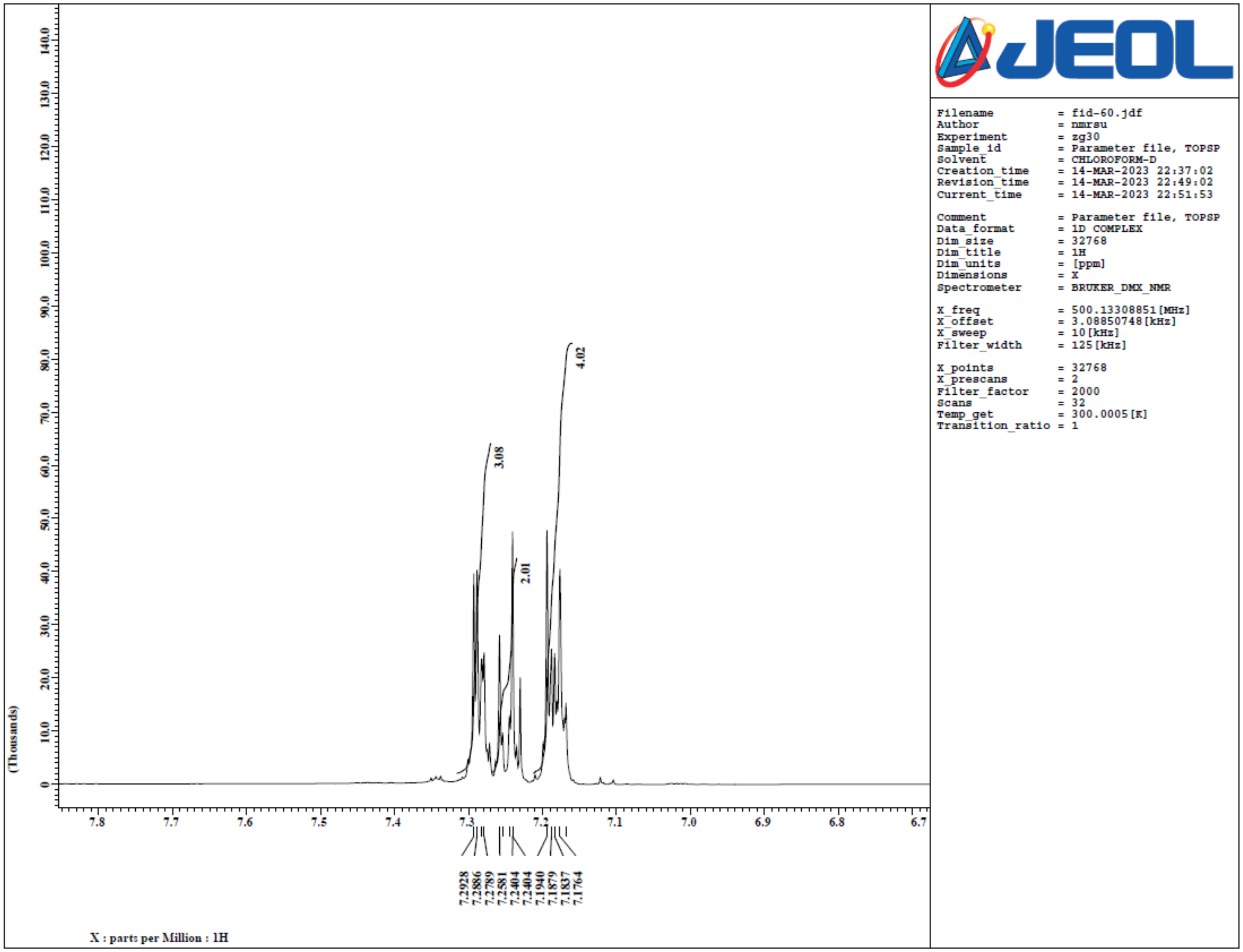

1-(4-chlorophenyl)-3-methyl-5-phenyl-1H-pyrazole (P17): ^{13}C-NMR ($CDCl_3$)

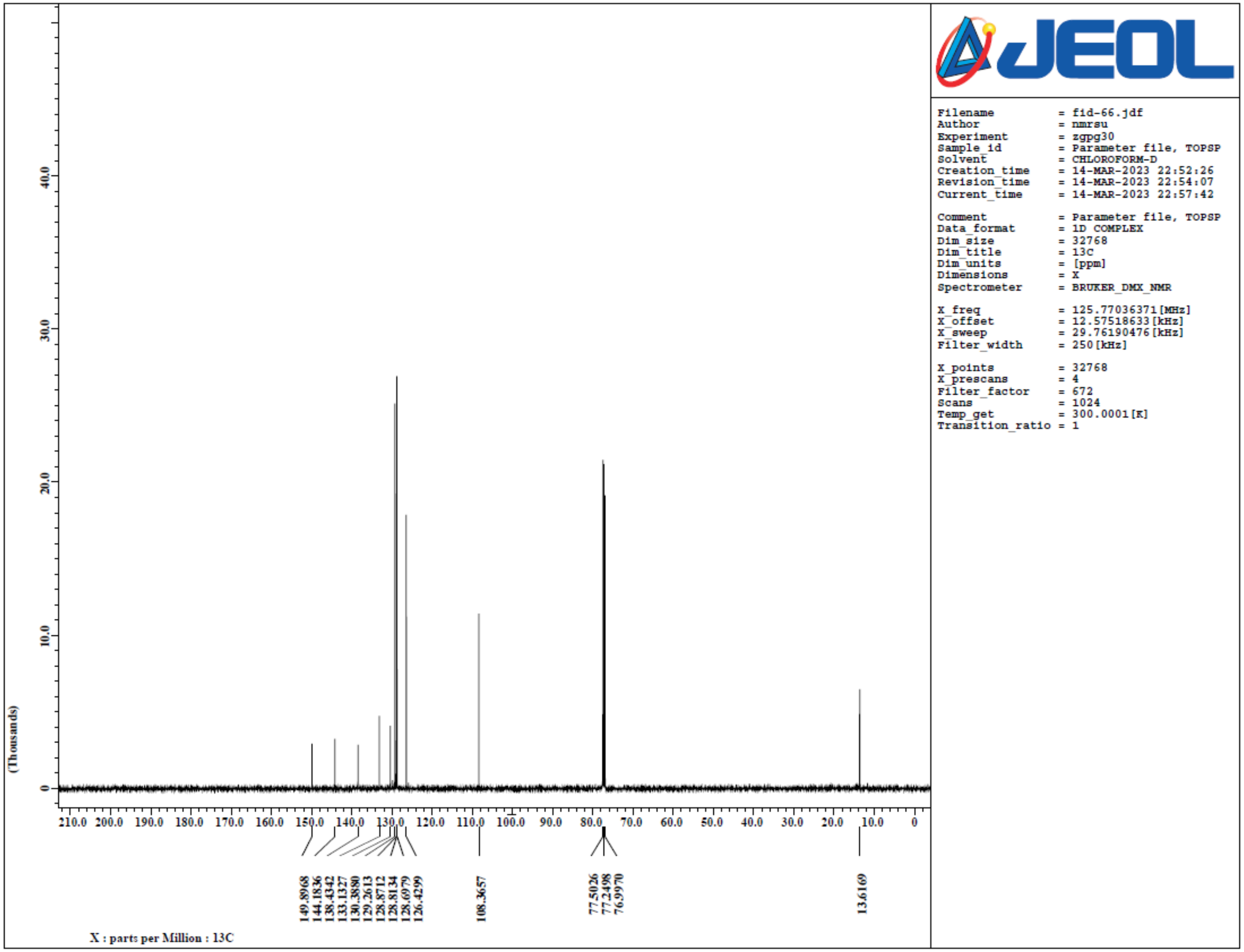

1-(4-chlorophenyl)-3-methyl-5-phenyl-1H-pyrazole (P17): ^{13}C-NMR ($CDCl_3$) _ Expanded Aromatic Region

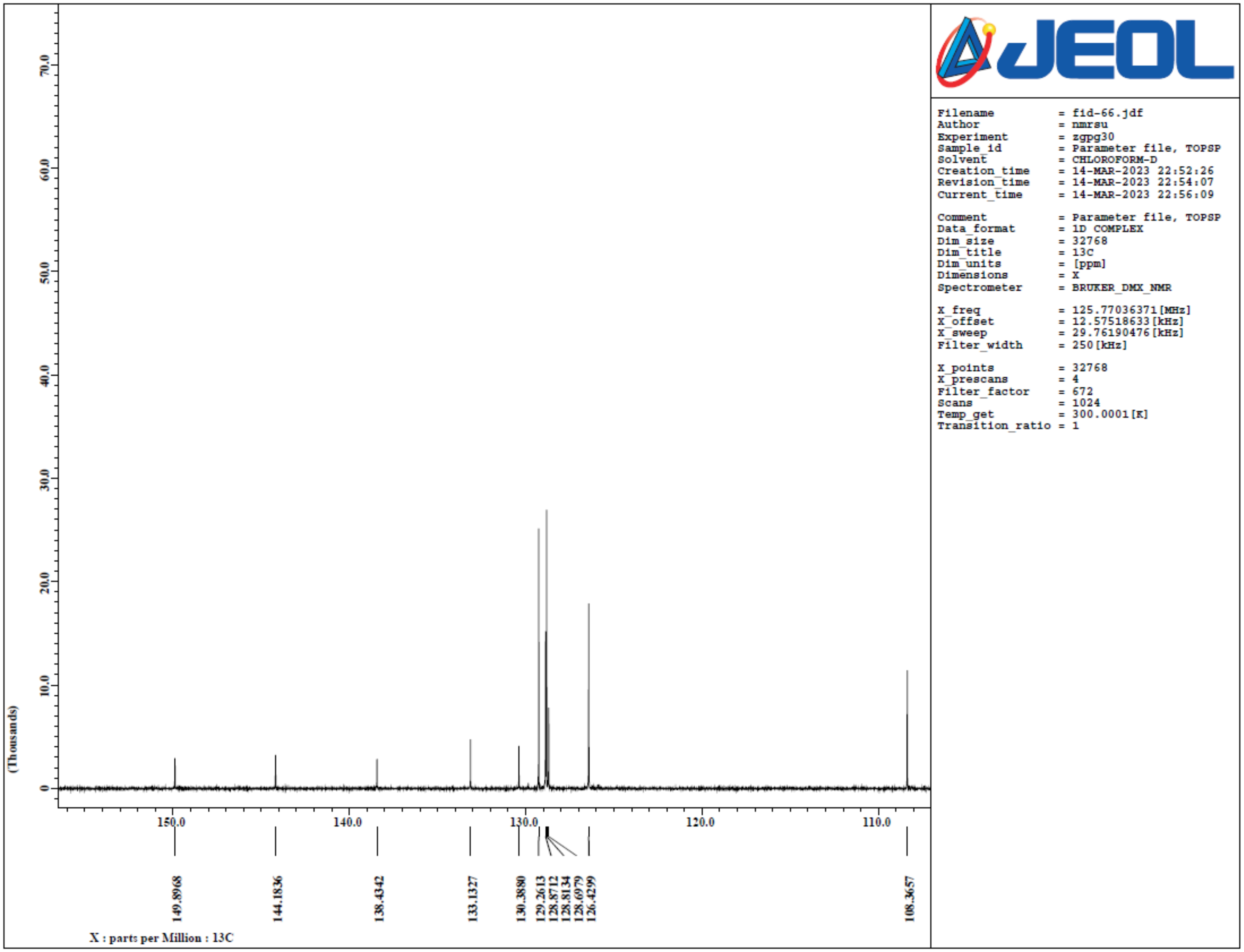

1-(4-chlorophenyl)-3-methyl-5-phenyl-1H-pyrazole (P17): ESI-MS

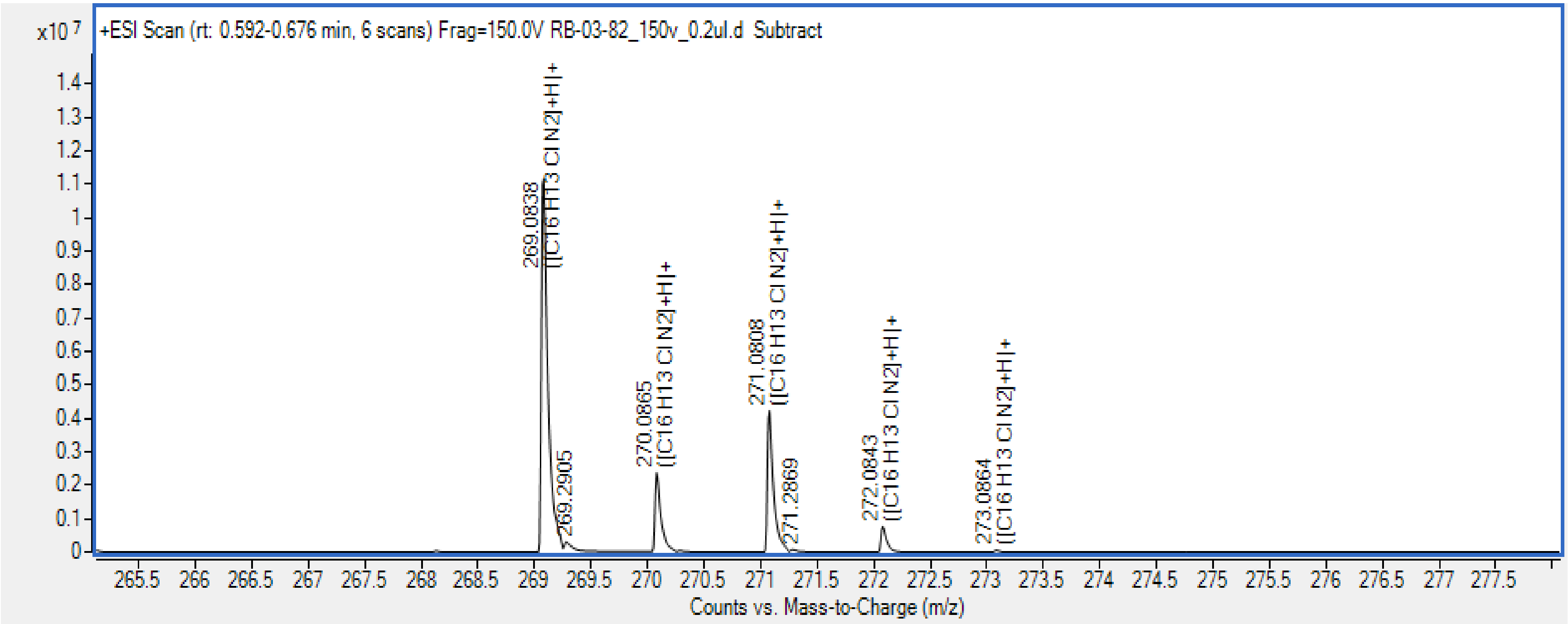

Formula,	Species,	m/z,	Score,	Diff (abs. ppm),	Mass
C16 H13 Cl N2,	(M+H)+,	269.0838,	97.12,	1.28,	268.0764

1-(4-methoxyphenyl)-3-methyl-5-phenyl-1H-pyrazole (P18): ^{1}H-NMR ($CDCl_3$)

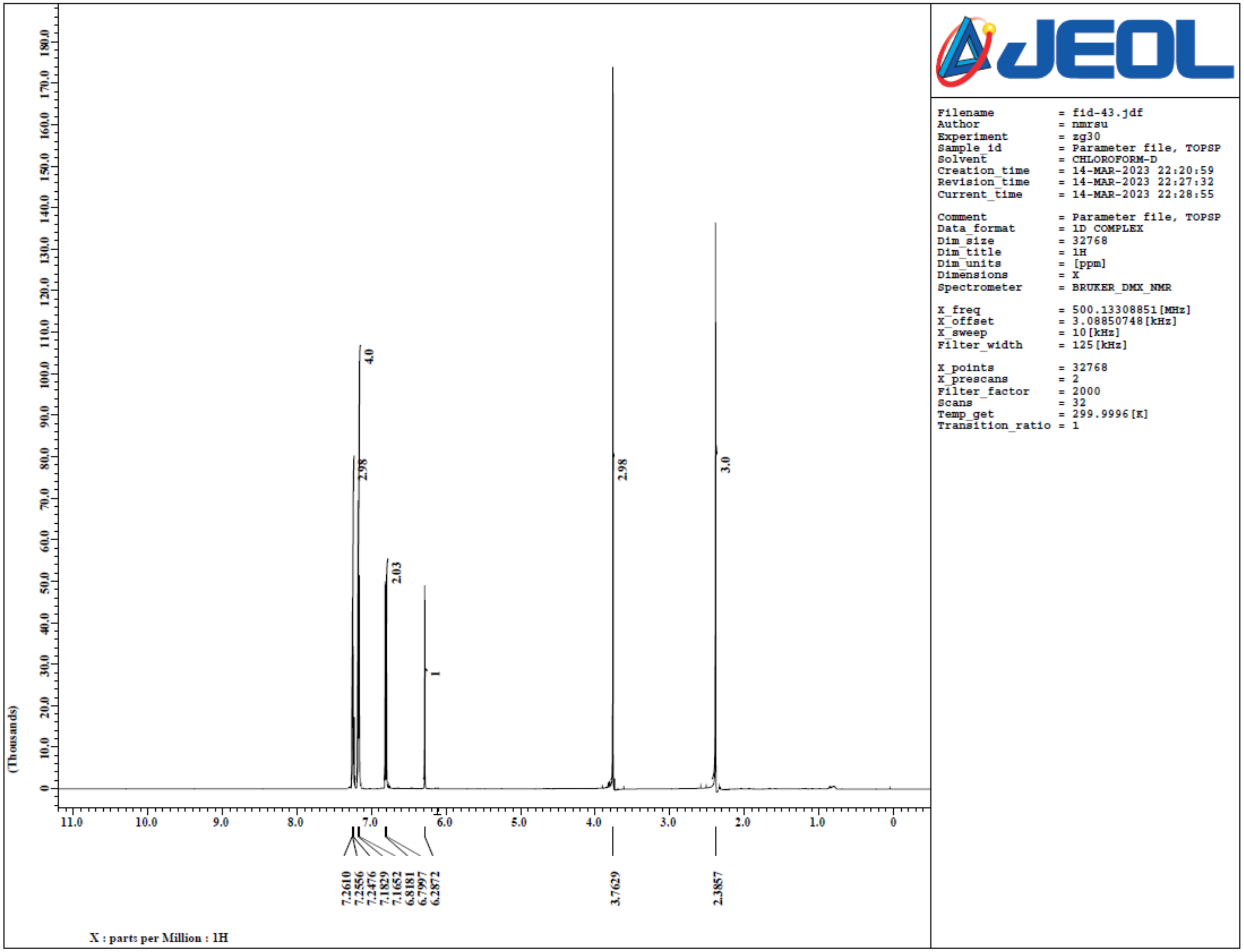

1-(4-methoxyphenyl)-3-methyl-5-phenyl-1H-pyrazole (P18): ^{1}H-NMR ($CDCl_3$) _ Expanded Aromatic Region

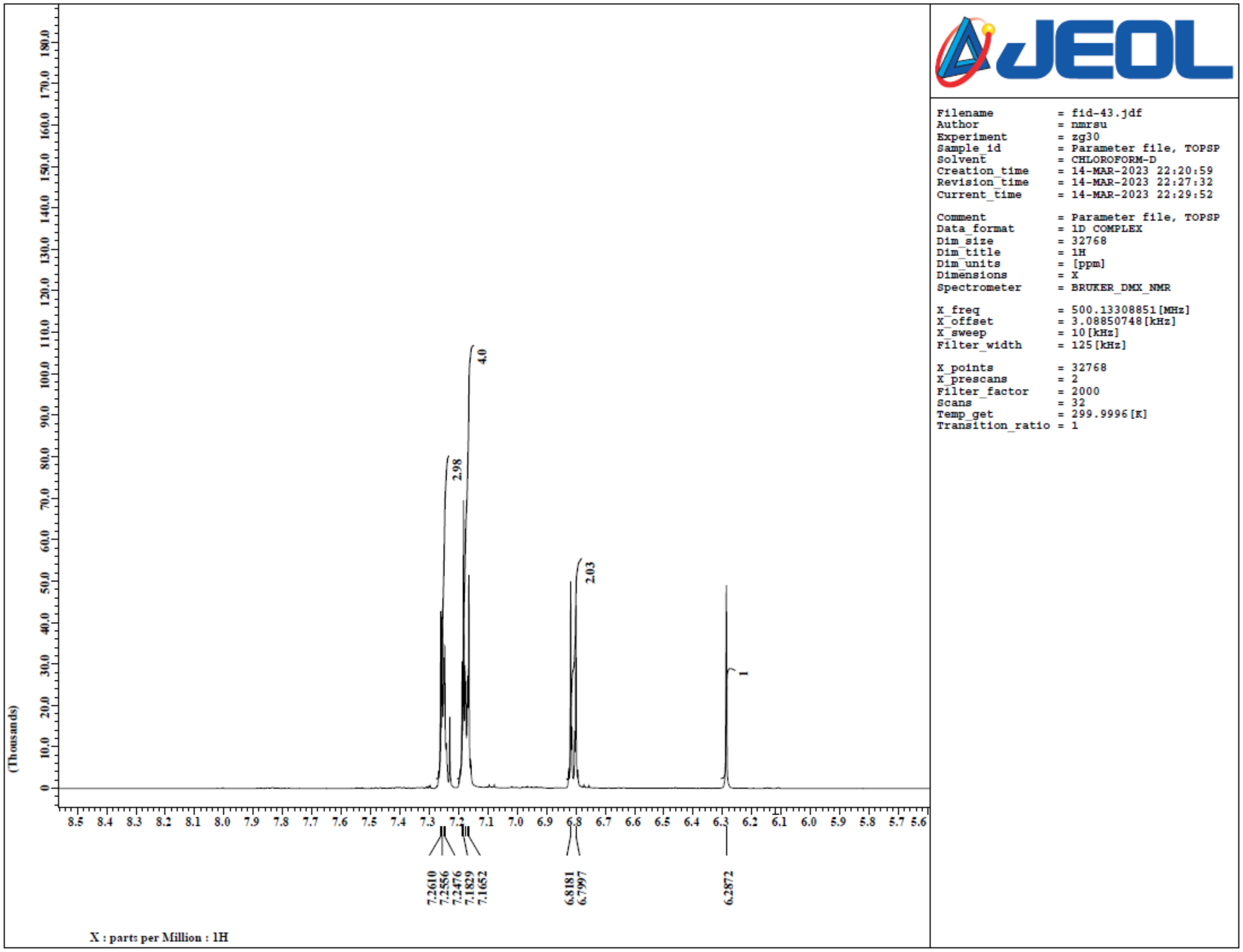

Filename = fid-43.jdf
Author = nmrsu
Experiment = zg30
Sample_id = Parameter file, TOPSP
Solvent = CHLOROFORM-D
Creation_time = 14-MAR-2023 22:20:59
Revision_time = 14-MAR-2023 22:27:32
Current_time = 14-MAR-2023 22:29:52

Comment = Parameter file, TOPSP
Data_format = 1D COMPLEX
Dim_size = 32768
Dim_title = 1H
Dim_units = [ppm]
Dimensions = X
Spectrometer = BRUKER_DMX_NMR

X_freq = 500.13308851[MHz]
X_offset = 3.08850748[kHz]
X_sweep = 10[kHz]
Filter_width = 125[kHz]

X_points = 32768
X_prescans = 2
Filter_factor = 2000
Scans = 32
Temp_get = 299.9996[K]
Transition_ratio = 1

1-(4-methoxyphenyl)-3-methyl-5-phenyl-1H-pyrazole (P18): ^{13}C-NMR ($CDCl_3$)

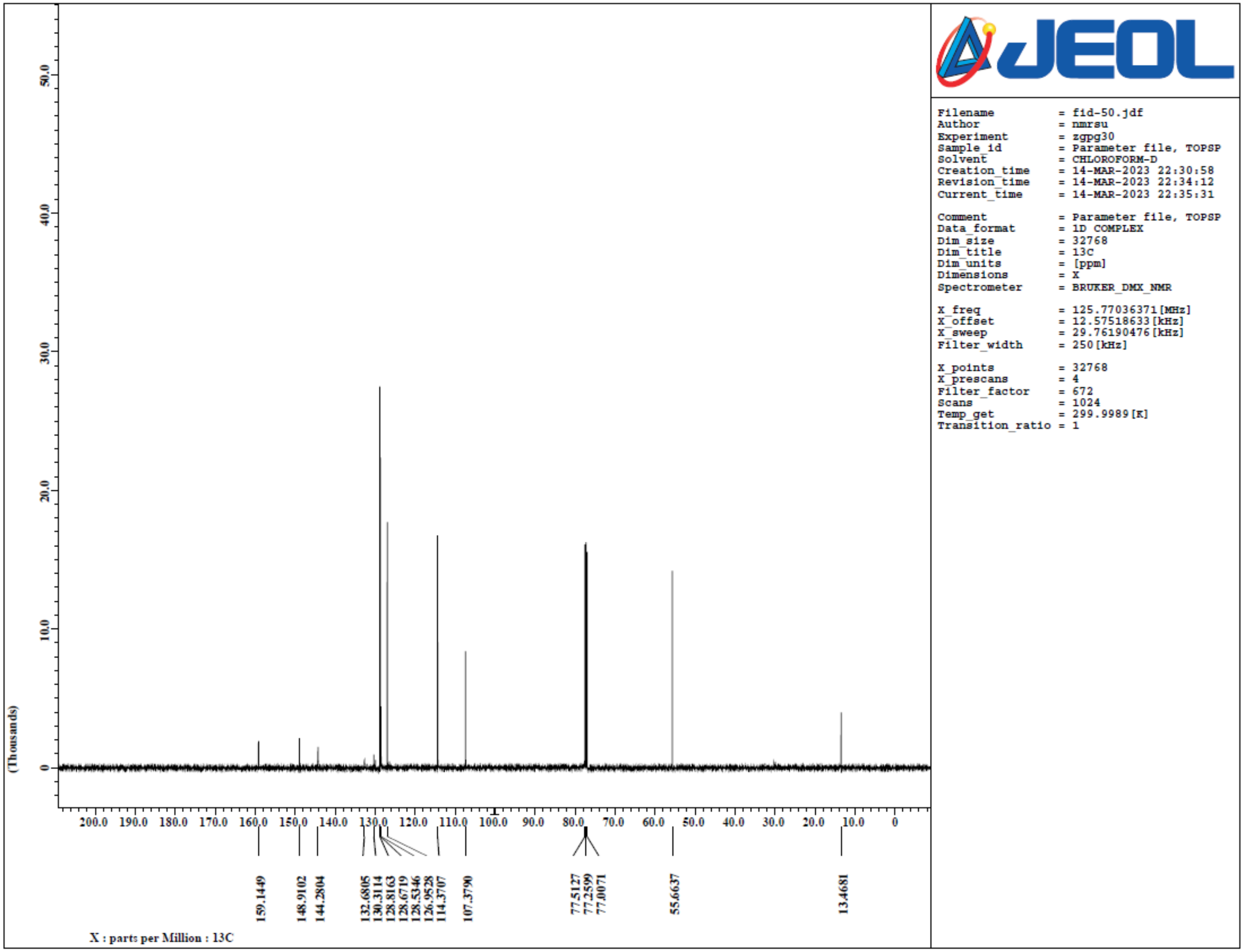

1-(4-methoxyphenyl)-3-methyl-5-phenyl-1H-pyrazole (P18): ESI-MS

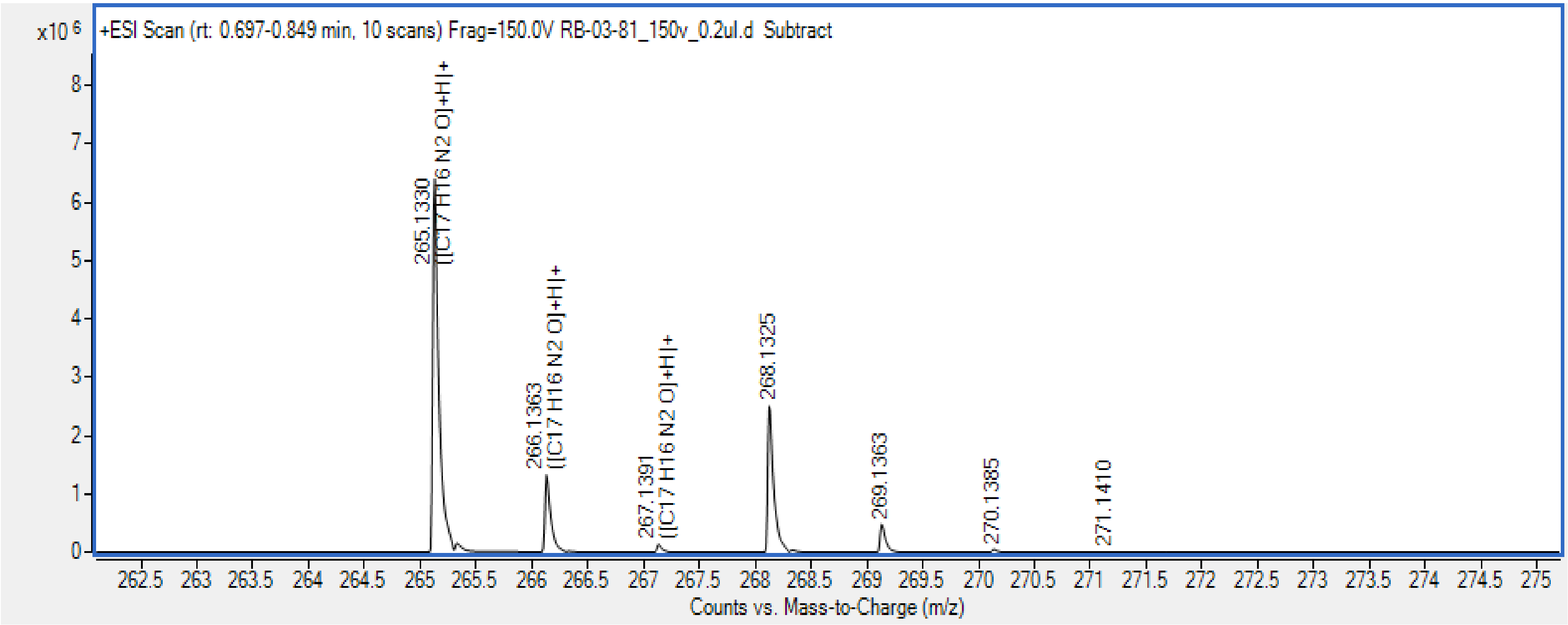

Formula,	Species,	m/z,	Score,	Diff (abs. ppm),	Mass
C17 H16 N2 O,	(M+H)+,	265.1330,	98.3,	1.94,	264.1258

4-(3-methyl-5-phenyl-1H-pyrazol-1-yl)benzenesulfonamide (P19): ^{1}H-NMR ($CDCl_3$)

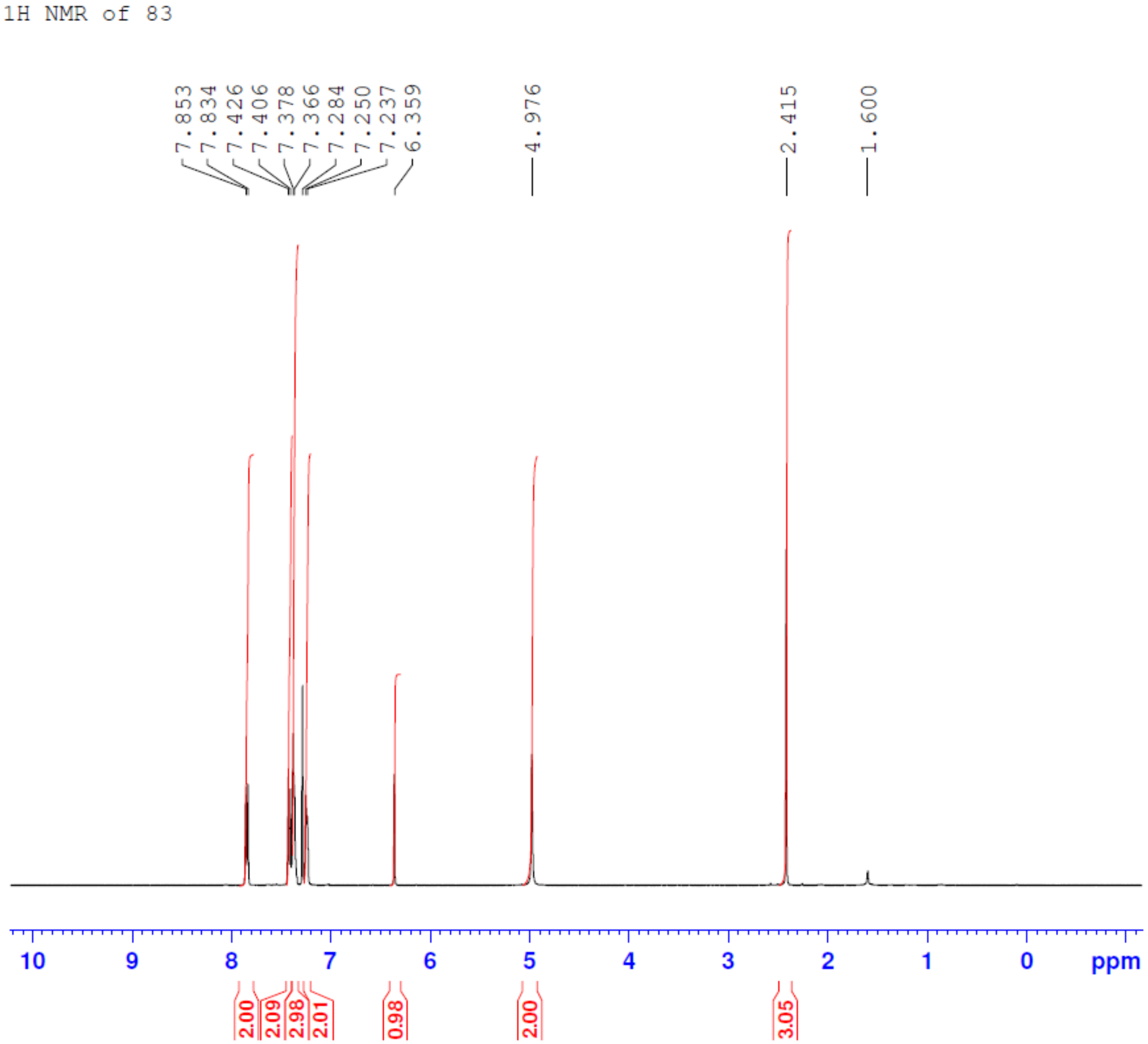


```
Current Data Parameters
NAME             SM-83_1H
EXPNO                  10
PROCNO                  1

F2 - Acquisition Parameters
Date_            20230317
Time                16.44 h
INSTRUM         Avance Neo
PROBHD       Z140678_0053 (
PULPROG              zg30
TD                  65536
SOLVENT             CDCl3
NS                     16
DS                      2
SWH              8196.722 Hz
FIDRES           0.250144 Hz
AQ              3.9976959 sec
RG                    101
DW                 61.000 usec
DE                  13.54 usec
TE                  296.3 K
D1             1.00000000 sec
TD0                     1
SFO1          400.1424709 MHz
NUC1                   1H
P0                   3.33 usec
P1                  10.00 usec
PLW1          22.64200020 W

F2 - Processing parameters
SI                  65536
SF            400.1400000 MHz
WDW                    EM
SSB                     0
LB                   0.30 Hz
GB                      0
PC                   1.00
```

4-(3-methyl-5-phenyl-1H-pyrazol-1-yl)benzenesulfonamide (P19): ^{1}H-NMR ($CDCl_3$) _ Expanded Aromatic Region

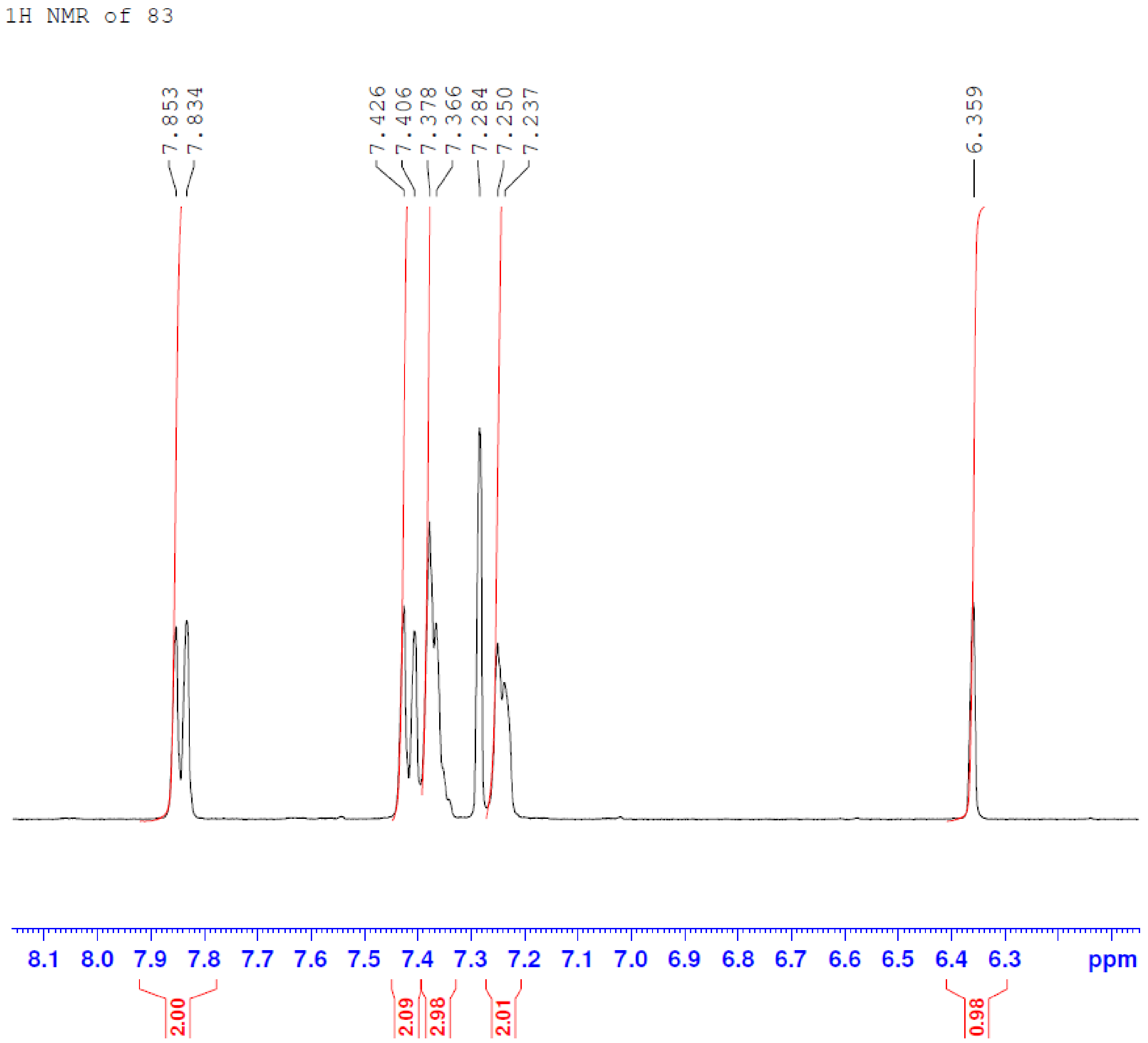


```
Current Data Parameters
NAME              SM-83_1H
EXPNO                   10
PROCNO                   1

F2 - Acquisition Parameters
Date_             20230317
Time                 16.44 h
INSTRUM          Avance Neo
PROBHD   Z140678_0053 (
PULPROG               zg30
TD                   65536
SOLVENT              CDCl3
NS                      16
DS                       2
SWH               8196.722 Hz
FIDRES            0.250144 Hz
AQ               3.9976959 sec
RG                     101
DW                  61.000 usec
DE                   13.54 usec
TE                   296.3 K
D1              1.00000000 sec
TD0                      1
SFO1           400.1424709 MHz
NUC1                    1H
P0                    3.33 usec
P1                   10.00 usec
PLW1           22.64200020 W

F2 - Processing parameters
SI                   65536
SF             400.1400000 MHz
WDW                     EM
SSB                      0
LB                    0.30 Hz
GB                       0
PC                    1.00
```

4-(3-methyl-5-phenyl-1H-pyrazol-1-yl)benzenesulfonamide (P19): ^{13}C-NMR ($CDCl_3$)

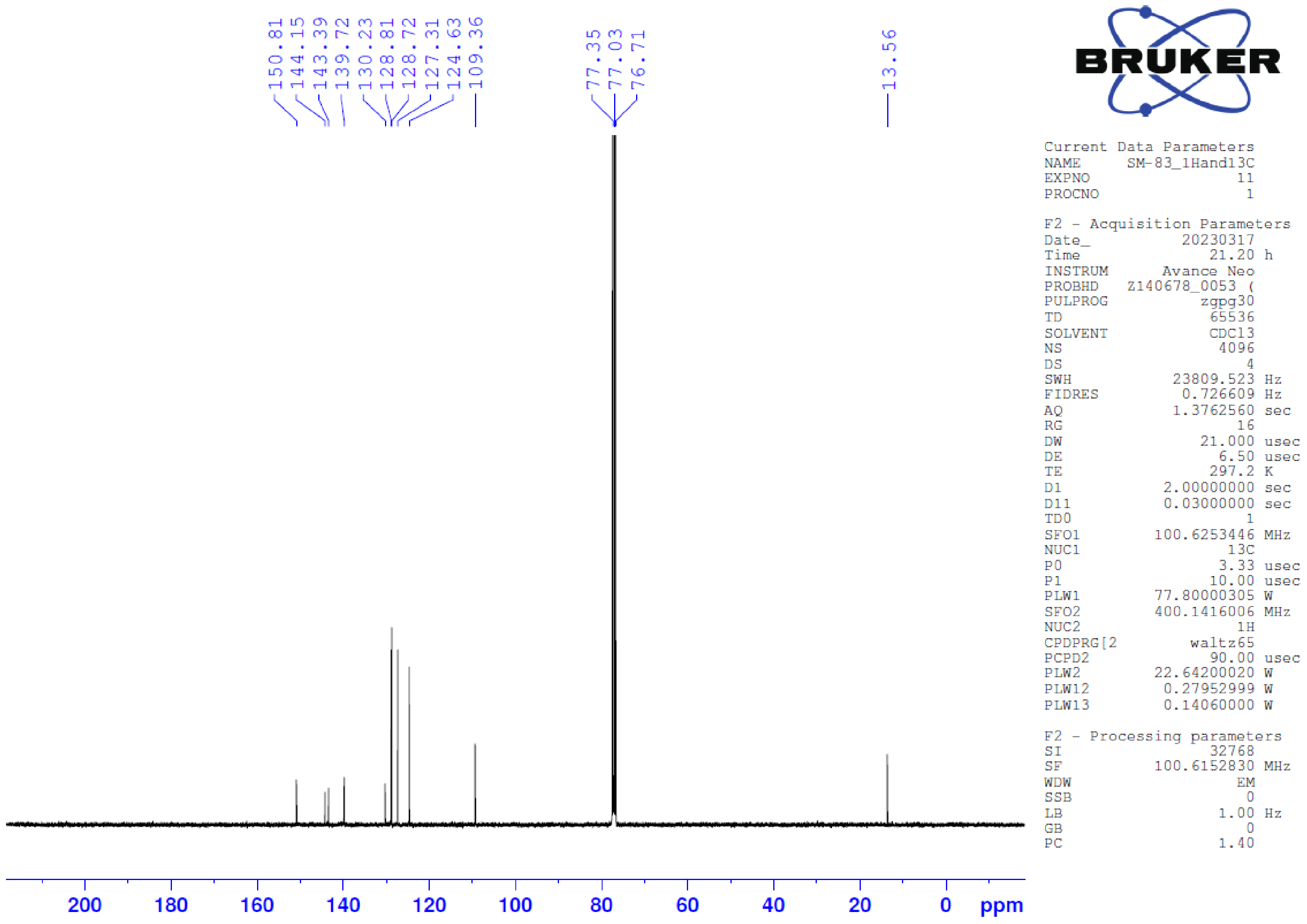

4-(3-methyl-5-phenyl-1H-pyrazol-1-yl)benzenesulfonamide (P19): ^{13}C-NMR ($CDCl_3$) _ Expanded Aromatic Region

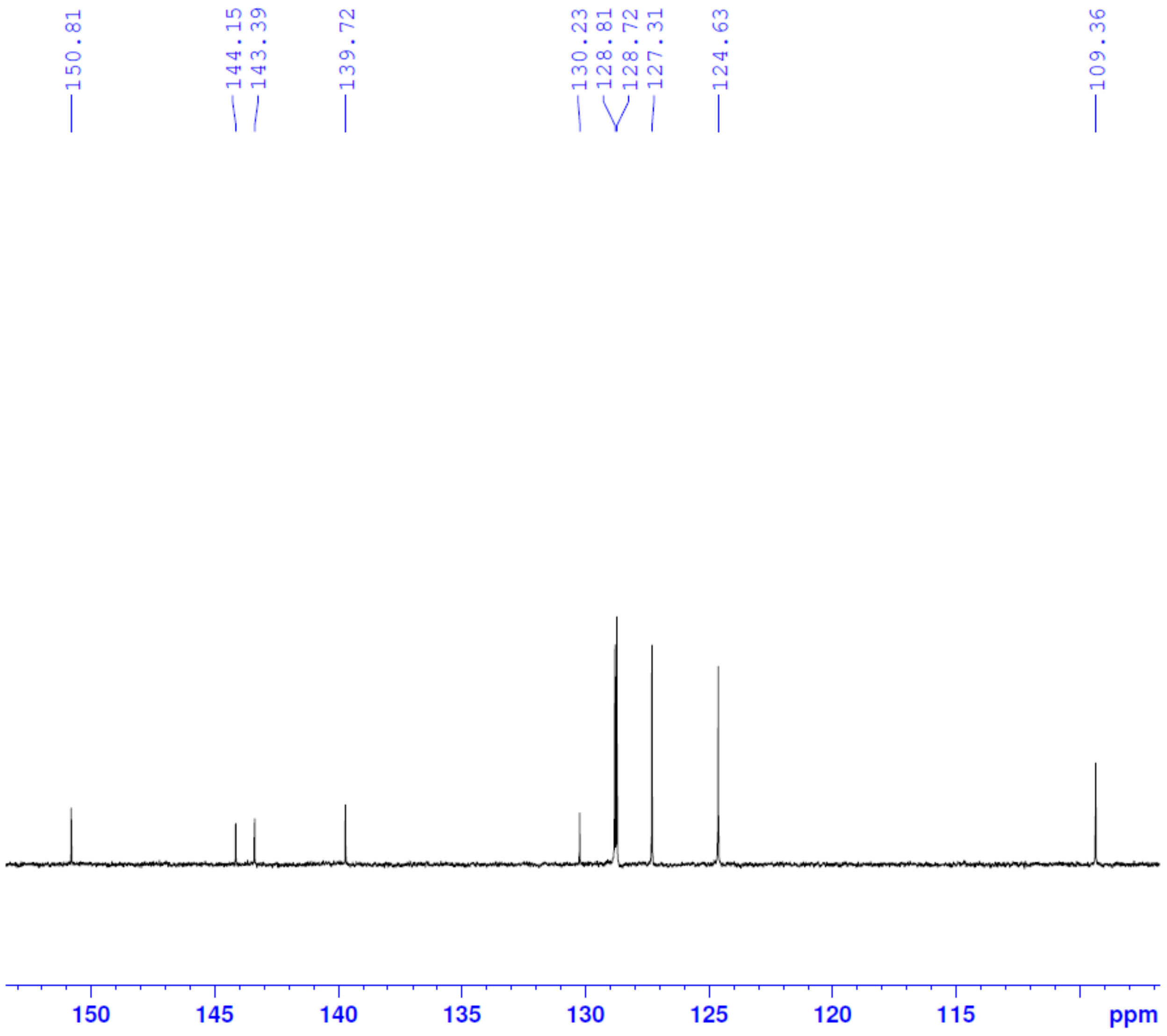

Current Data Parameters
NAME SM-83_1Hand13C
EXPNO 11
PROCNO 1

F2 - Acquisition Parameters
Date_ 20230317
Time 21.20 h
INSTRUM Avance Neo
PROBHD Z140678_0053 (
PULPROG zgpg30
TD 65536
SOLVENT CDCl3
NS 4096
DS 4
SWH 23809.523 Hz
FIDRES 0.726609 Hz
AQ 1.3762560 sec
RG 16
DW 21.000 usec
DE 6.50 usec
TE 297.2 K
D1 2.00000000 sec
D11 0.03000000 sec
TD0 1
SFO1 100.6253446 MHz
NUC1 13C
P0 3.33 usec
P1 10.00 usec
PLW1 77.80000305 W
SFO2 400.1416006 MHz
NUC2 1H
CPDPRG[2 waltz65
PCPD2 90.00 usec
PLW2 22.64200020 W
PLW12 0.27952999 W
PLW13 0.14060000 W

F2 - Processing parameters
SI 32768
SF 100.6152830 MHz
WDW EM
SSB 0
LB 1.00 Hz
GB 0
PC 1.40

4-(3-methyl-5-phenyl-1H-pyrazol-1-yl)benzenesulfonamide (P19): ESI-MS

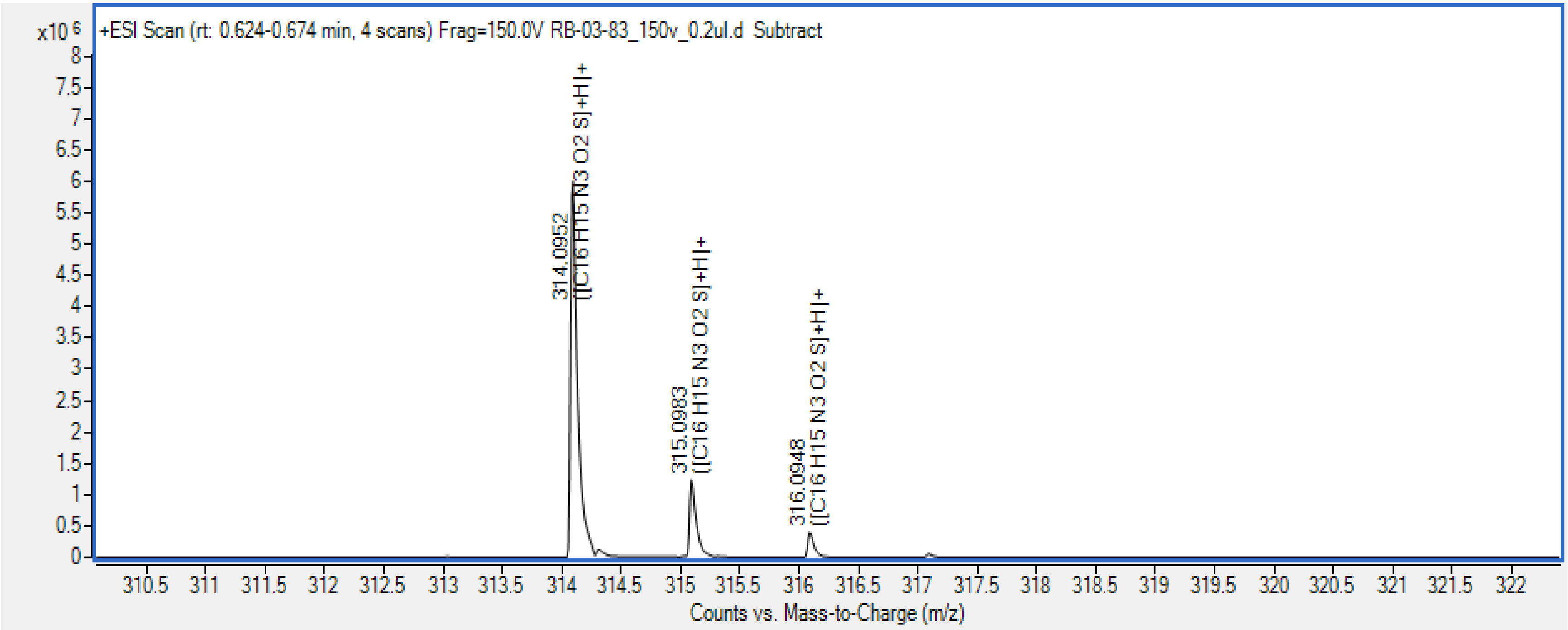

Formula,	Species,	m/z,	Score,	Diff (abs. ppm),	Mass
C16 H15 N3 O2 S,	(M+H)+,	314.0952,	98.14,	1.61,	313.088
